# Comparative effectiveness of moxibustion-based combination therapies for lumbar disc herniation: a systematic review and network meta-analysis of 50 randomized trials

**DOI:** 10.3389/fneur.2026.1809677

**Published:** 2026-05-14

**Authors:** Dongmin Du, Shurong Wang, Shuran Wang, Han Sun, Yi Shan

**Affiliations:** 1 Heilongjiang University of Chinese Medicine, Harbin, China; 2 Heilongjiang University of Chinese Medicine Affiliated Second Hospital, Harbin, China

**Keywords:** acupuncture, functional recovery, lumbar disc herniation, moxibustion, network meta-analysis, pain relief, traction, traditional Chinese medicine

## Abstract

**Objective:**

Moxibustion, a widely utilized external therapeutic technique in traditional Chinese medicine, has become an integral part of non-surgical management for lumbar disc herniation (LDH). This study seeks to perform a systematic review and network meta-analysis (NMA) to evaluate and compare the efficacy of different moxibustion-based combination therapies in the treatment of LDH.

**Methods:**

This study employs NMA to evaluate randomized controlled trials (RCTs) published up to January 14, 2026. A systematic search of the literature was conducted across multiple databases, including PubMed, EMBASE, the Cochrane Library, Web of Science, and Chinese databases such as CNKI and Wanfang Medical Database. The interventions assessed include moxibustion alone, as well as combinations of moxibustion with acupuncture (ACU), tuina (TUINA), lumbar traction (REHAB), traditional Chinese medicine therapy (TCM), and conventional intervention (CT). The primary outcomes include the Visual Analog Scale (VAS) score for pain, the Oswestry Disability Index (ODI) score for functional improvement, and the Japanese Orthopedic Association (JOA) score for clinical symptoms. Statistical analysis was conducted using Stata 17.0 MP, with a random-effects model applied to calculate mean differences (MD) and risk ratios (RR). The quality of evidence was assessed using the GRADE framework.

**Results:**

A total of 50 studies involving 4,399 patients were included in the analysis. The results suggest that, for the JOA score, low to moderate-quality evidence indicates that MOXI+CT (MD = 11.93, 95% CI: 8.88 to 14.98) and MOXI+TUINA (MD = 7.81, 95% CI: 4.4 to 11.23) significantly improved the JOA score compared to CT. For the ODI score, low to moderate-quality evidence indicates that MOXI+ACU (MD = −10.11, 95% CI: −11.92 to −8.3) and MOXI+TUINA (MD = −6.52, 95% CI: −8.58 to −4.46) significantly reduced the ODI score compared to CT. In the case of the VAS score, very low to low-quality evidence suggests that MOXI+ACU (MD = −1.99, 95% CI: −3.06 to −0.92) and MOXI+TUINA (MD = −2.2, 95% CI: −3.4 to −0.99) significantly reduced the VAS score compared to CT. Regarding the cure rate, moderate-quality evidence shows that MOXI+TUINA (RR = 2.45, 95% CI: 1.55 to 3.86) and MOXI+TCM (RR = 1.75, 95% CI: 1.31 to 2.33) significantly improved the cure rate compared to CT.

**Conclusion:**

Moderate to very low evidence indicates that the combination of TUINA+MOXI significantly reduces pain, enhances functional recovery, and promotes overall rehabilitation in patients with LDH. These results suggest that combining moxibustion with other therapeutic approaches may provide an effective non-surgical alternative for the management of LDH.

## 1 Introduction

Lumbar disc herniation (LDH) is a prevalent etiology of low back and leg pain. Its pathophysiology is characterized by the protrusion of the nucleus pulposus, typically resulting from intervertebral disc degeneration or injury, which compresses the nerve roots. This compression induces a spectrum of clinical symptoms, including pain, numbness, and functional impairments, all of which profoundly affect patients' quality of life (1). Most patients follow a benign natural course, with symptoms and functional limitations improving following conservative treatment, Non-surgical interventions should be regarded as the first-line approach (2). Therefore, clinical management should prioritize pain relief, functional improvement, optimization of conservative treatment strategies, and the reduction of unnecessary invasive procedures and long-term reliance on medication.

Over 85% of patients experience symptomatic relief over time with non-surgical management, which may encompass bed rest, traction, manual therapy, acupuncture, and nonsteroidal anti-inflammatory drugs (3). Nevertheless, the efficacy of non-surgical treatments is constrained by factors such as individual characteristics, patient preferences, adherence challenges, potential side effects, and economic costs (4). Consequently, both patients and clinicians generally prefer a multimodal, conservative treatment approach. However, there is a notable absence of systematic and clear comparative evidence regarding the optimal combinations of interventions and the most advantageous treatment strategies.

Moxibustion, a traditional Chinese medicine external therapy, is extensively utilized in clinical practice for the non-surgical treatment of LDH (5). In traditional Chinese medicine, LDH is commonly categorized as “lumbago”. Its development is frequently attributed to factors such as constitutional weakness and the invasion of external pathogens. The underlying pathophysiology is primarily characterized by stagnation of qi and blood stasis, or by the obstruction of the meridians due to cold and dampness, alongside deficiencies in the liver and kidneys. These deficiencies impair the nourishment of the muscles and bones, leading to meridian blockages and subsequent pain due to impaired circulation (6). Moxibustion exerts its therapeutic effects chiefly through thermal stimulation, which triggers vascular and other physiological changes at specific sites on the body's surface. In the context of traditional Chinese medicine, moxibustion is thought to harmonize the flow of qi and blood, unblock the meridians, and restore proper circulation, thereby alleviating pain and mitigating inflammatory responses (7). Acupuncture improves blood circulation and oxygen delivery, facilitating the restoration of neural function. Additionally, it regulates the body's endogenous pain suppression systems and associated neuroendocrine pathways, thereby providing analgesic effects and enhancing lumbar spine function (8). Tuina may reduce pain and improve function through mechanisms including the relaxation of muscles and tendons, enhancement of local blood circulation, restoration of spinal biomechanical balance, and attenuation of inflammatory responses (9). The traditional Chinese medicine formula exerts its therapeutic effects by “activating blood circulation, resolving blood stasis, unblocking the meridians, alleviating pain, Tonifying Qi, and strengthening the kidneys”. Its contemporary mechanisms include the inhibition of inflammatory cytokine release, enhancement of microcirculation in the nerve roots, reduction of oxidative stress, and promotion of nerve regeneration (10). Lumbar traction alleviates mechanical compression on the nerve roots by inducing vertebral separation, which increases the diameter of the intervertebral foramen and enhances local blood circulation (11). Therefore, the ‘thermal stimulation' provided by moxibustion may synergize with treatments such as acupuncture, tuina, lumbar traction, and traditional Chinese medicine, thereby enhancing overall therapeutic efficacy.

Traditional meta-analyses have predominantly examined the comparative efficacy of moxibustion alone vs. its combination with acupuncture, tuina, electroacupuncture, and other therapeutic modalities in the treatment of LDH. These analyses suggest that certain combination therapies may offer superior outcomes compared to standard monotherapies (12). However, the absence of comparative studies on different moxibustion combination therapies has left the most effective adjunctive treatment unresolved. This study seeks to systematically evaluate and rank the efficacy of various treatment protocols, including moxibustion combined with acupuncture, tuina, traction, traditional Chinese medicine, and conventional therapies.

Due to variations in the materials used and the specific procedural details of moxibustion, different techniques may demonstrate distinct characteristics in terms of stimulus intensity, method of application, and overall procedure (13). This study will systematically assess the advantages of warm acupuncture, thermosensitive moxibustion, fire-cupping moxibustion, and traditional moxibustion techniques, including ginger moxibustion, moxibustion with other substances, Taiyi Shen moxa rolls, and conventional moxibustion.

This study aims to systematically compare the efficacy of various moxibustion combination therapies for LDH using network meta-analysis (NMA), offering evidence from two complementary perspectives. First, at the effect level, moxibustion will be treated as a single intervention to evaluate its relative efficacy and rank within the broader network of combination therapies. Second, from a technical standpoint, moxibustion will be categorized into warm acupuncture, fire-cupping moxibustion, thermosensitive moxibustion, and traditional moxibustion, enabling a detailed comparison of the relative advantages and potential sources of variation among different moxibustion-based treatment regimens.

The primary outcomes include pain, as measured by the Visual Analog Scale (VAS) score, and functional improvement, as assessed by the Oswestry Disability Index (ODI) score and the Japanese Orthopedic Association (JOA) score. Additionally, treatment rankings and an evaluation of the certainty of the evidence will be presented, providing an evidence-based framework to inform clinical decision-making regarding combination therapies and guide future research directions.

## 2 Materials and methods

This NMA was performed following the guidelines outlined in the Preferred Reporting Items for Systematic Reviews and Meta-Analyses (PRISMA) extension for network meta-analyses (Supplementary Table S1) (14). To uphold transparency, reliability, and innovation, the protocol for this study has been registered with the Prospective Register of Systematic Reviews (CRD420261299978).

### 2.1 Data sources and search strategy

This study conducted a thorough search across multiple databases, including PubMed, EMBASE, Cochrane Library, Web of Science, CNKI, and Wanfang Medical Database. The search terms included “Intervertebral Disc Displacement,” “Disc Displacement, Intervertebral,” “Intervertebral Disk Displacement,” “Disk, Protruded,” “Moxibustion,” “Heat-sensitive Moxibustion,” “Thunder-fire Moxibustion,” “Warm-needling Moxibustion,” and “Randomized Controlled Trial” (Supplementary Table S2). The search encompassed all publications from the inception of each database up to January 14, 2026, utilizing both free-text and subject heading terms, without any language restrictions.

### 2.2 Selection criteria

Inclusion criteria: 1. Randomized controlled trials (RCTs) that include patients diagnosed with LDH based on either traditional Chinese medicine or Western medical diagnostic criteria. Diagnosis was made in accordance with the “Clinical Practice Guideline for Diagnosis and Treatment of Lumbar Disc Herniation” (15), with confirmation through MRI or CT imaging. Diagnosis was made in accordance with the “Clinical Practice Guidelines of Traditional Chinese Medicine Rehabilitation for Low Back Pain (Herniated Lumbar Disc)” (16), with primary symptoms including radiating pain or numbness in the lower limbs, accompanied by restricted movement of the lumbar spine. 2. RCTs assessing moxibustion, either alone or in combination with adjunctive therapies, were included. Moxibustion techniques were categorized into four types: warm needling (WN), heat-sensitive moxibustion (HM), thunder-fire moxibustion (TF), and traditional moxibustion (CM). Traditional moxibustion includes methods such as ginger-based moxibustion, moxibustion using barrier materials, Taiyi Shen moxa rolls, and general moxibustion. For clarity, these moxibustion techniques were collectively referred to as MOXI. Adjunctive therapies included acupuncture, electroacupuncture, ear acupuncture, tuina, massage, lumbar traction, and traditional Chinese medicine treatments, such as herbal fumigation, cupping therapy, and others. These were classified as follows: acupuncture, electroacupuncture, and ear acupuncture as ACU. Tuina, massage, and manual soft-tissue as TUINA. Lumbar traction as REHAB and traditional Chinese medicine therapies, including oral herbal medicine, fumigation, medicinal poultices, fumigation washes, and cupping, as TCM. 3. RCTs comparing conventional treatments for LDH were included. Conventional treatment, encompassing Western medicine, multimodal therapy, and nursing education, was classified as CT. 4. RCTs are required to report at least one of the following outcome measures: VAS score was used to assess the intensity of overall pain, as well as pain in the lower back and legs, with scores ranging from 0 to 10, where 10 indicates the most severe pain (17). If the VAS score is reported on a 0–100 mm scale, the score is divided by 10 and converted to a 0–10 scale for consistent analysis (18). ODI score is used to assess the degree of functional impairment, with scores ranging from 0 to 100, where higher scores reflect greater disability (19). JOA score evaluates the patient's clinical symptoms and signs, with a total score ranging from 0 to 29, where higher scores indicate less severe symptoms (20). Cure rate: Cure is defined as a straight leg raise exceeding 70° (21) or a JOA improvement of 75% or more (22). Adverse events: any untoward reactions occurring during the randomized controlled trial.

Exclusion criteria included: 1. RCTs with undefined or ambiguous outcome measures. 2. Systematic reviews or case reports.

Before including RCTs, we performed an initial screening based on titles and abstracts. Each included trial was independently verified by two reviewers to ensure that the data reflected the most recent publications.

### 2.3 Data extraction and quality assessment

Data from RCTs were independently extracted by the researchers in accordance with the Preferred Reporting Items for Systematic Reviews and Meta-Analysis guidelines. Any discrepancies were resolved through discussion with the second author. For each study, the following information was extracted: first author, publication year, sample size, patient age, gender and regional distribution, follow-up duration, and the intervention protocols for both the experimental and control groups. For continuous outcomes, the mean values and standard deviations (SDs) reported at the conclusion of the treatment period were prioritized. In cases where the SD was not directly provided, it was estimated using the methods outlined in the Cochrane Handbook, with standard error (SE), 95% confidence interval (CI), t-value, or *P*-value used to compute the SD. Where necessary, the SD was approximated from interquartile ranges (IQR) or range data. For dichotomous outcomes, the number of events and the total sample size in each group were extracted.

This study evaluated the quality of the included trials using the ROB2 tool, which is grounded in the evidence-based principles of systematic reviews and meta-analyses. This tool assesses potential sources of bias across five key domains: randomization process, deviations from the intended interventions, missing outcome data, outcome measurement, and selective reporting. For each domain, as well as for the overall risk of bias, studies were categorized as low risk, some concerns or high risk (23).

### 2.4 Statistical analysis

This study utilized Stata 17.0 MP to perform a NMA. For continuous outcomes with consistent scales and units across studies, mean differences (MD) and their corresponding 95% CIs were calculated. For dichotomous outcomes, risk ratios (RR) and their 95% CIs were estimated. In multi-arm trials, pairwise comparisons were derived using the augment format, retaining within-study correlation structures to prevent the underestimation of SEs due to the repeated use of control groups. For dichotomous outcomes, continuity correction (r = r + 0.5, n = n+1) was applied to all treatment arms of studies with zero events or zero non-events, in order to avoid infinite RR estimates. The primary analysis was conducted using a random-effects model under the consistency assumption, with between-study variance (τ^2^) estimated via restricted maximum likelihood (REML). When closed loops were present in the network, global inconsistency tests were employed to evaluate overall consistency, complemented by local inconsistency assessments using the node-splitting method. A *p*-value of < 0.05 was considered indicative of potential inconsistency. The loop inconsistency factor (IF) was also calculated to evaluate closed-loop consistency; if the 95% CI for the IF contained 0, this suggested no statistical evidence of inconsistency between direct and indirect evidence. A network plot was generated to visually represent the geometry of the network. In the plot, node size was proportional to the total sample size for each treatment, and the thickness of connecting lines reflected the number of studies comparing each intervention pair. To rank the interventions, multiple ranking metrics were used, including the cumulative ranking curve area (SUCRA), the probability of the best treatment (PreBest), and the average rank, in order to enhance the robustness and interpretability of the results. Publication bias and small-sample effects were assessed through comparison-adjusted funnel plots. Sensitivity analysis was performed using a leave-one-out approach, where each study was sequentially excluded, and the random-effects consistency model was re-estimated to compare the direction and magnitude of pooled effects. Additionally, univariate network meta-regression was conducted to explore the impact of study-level covariates on treatment effects, with regression coefficients, 95% CIs, and Wald test *p*-values reported. A *p*-value of < 0.05 was considered evidence of a statistically significant modifying effect of the covariate.

### 2.5 Evidence grading

This study evaluates the certainty of evidence in the results of the NMA using the GRADE framework and CINeMA (Confidence in NMA). RCTs are initially rated as “high certainty.” Evidence certainty is then assessed across six domains: risk of bias within studies, indirectness, imprecision, heterogeneity, inconsistency, and risk of bias between studies (publication bias/small-study effects). Risk of bias within studies is assessed using the RoB 2.0 tool, applied to each domain. The CINeMA contribution matrix is subsequently used to weight and aggregate bias according to its impact on the network estimates at the comparison level. Indirectness is assessed based on the assumptions of transitivity and exchangeability, with predefined potential effect modifiers, including baseline severity, intervention intensity, and follow-up duration. Consistency between direct and indirect evidence is evaluated in terms of the population, intervention, comparator, and outcome measures. Imprecision is determined using predefined minimal clinically important differences (MIDs): a mean difference (MD) of 2 for continuous outcomes (VAS score) (24); MD of 13 for continuous outcomes (JOA score) (20); and MD of 10 for continuous outcomes (ODI score) (25). This is combined with the 95% CIs to determine whether they cross the null effect line and exceed these thresholds. Heterogeneity is primarily assessed using τ^2^ from the random effects model and the position of the prediction interval relative to the MID. For networks with closed loops, inconsistency is assessed using CINeMA's built-in direct–indirect consistency evaluation, incorporating methods such as node-splitting, SIDE or design-by-treatment. Risk of bias between studies is evaluated by examining trial registration, gray literature retrieval, and considering small-study effects indicated by the comparison-adjusted funnel plot. Each domain is rated as no concern, some concern, or serious concern, with downgrading according to GRADE principles: one level downgrade for some concern and two levels for serious concern. Ultimately, the certainty of the evidence is classified as high, moderate, low, or very low.

## 3 Result

### 3.1 Selection of literature and study characteristics

**Figure 1:**
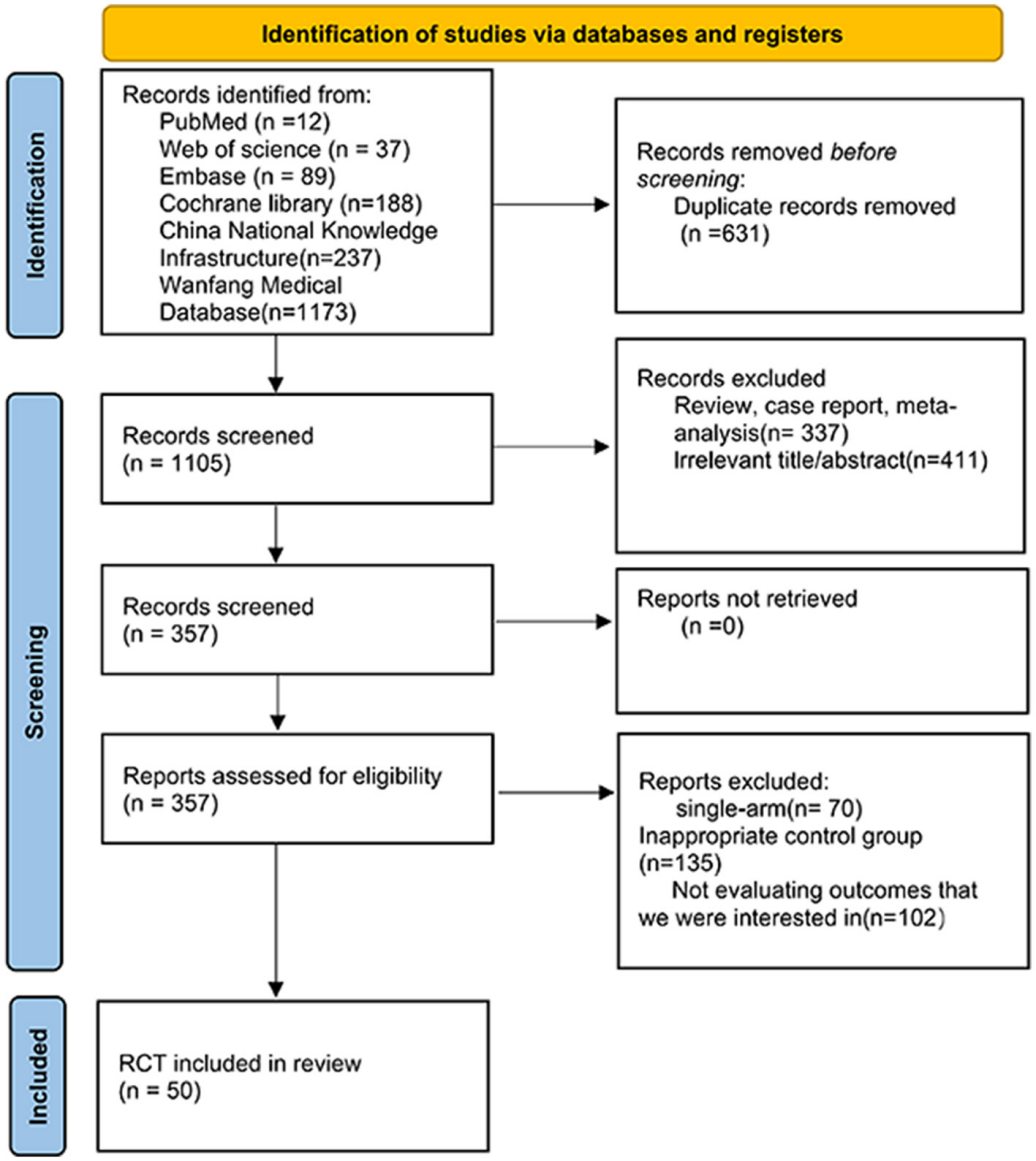
Flow diagram of the study selection process.

In the initial literature search, 1,736 records were retrieved from the database. After screening the abstracts to remove duplicates and irrelevant articles, 357 studies were identified for full-text review. Ultimately, 50 studies met the inclusion and exclusion criteria (Figure 1) (Supplementary Table S3). This analysis included a total of 4,399 patients who received one of the following 10 interventions: MOXI, ACU, TUINA, REHAB, TCM, CT, MOXI, and ACU (ACU+MOXI), CT and MOXI (CT+MOXI), TCM and MOXI (TCM+MOXI), and TUINA and MOXI (TUINA+MOXI). In the subgroup analyses, moxibustion combined regimen was further classified into four types: TF combined regimen, WN combined regimen, HM combined regimen, and CM combined regimen. The intervention durations varied as follows: Moxibustion (3–28 days), Acupuncture (10–30 days), Tuina (23–30 days), Traction (14–28 days), Traditional Chinese Medicine (14–28 days), Conventional Treatment (3–30 days), Moxibustion and Acupuncture (20–40 days), Conventional Treatment and Moxibustion (14–28 days), Traditional Chinese Medicine and Moxibustion (10–30 days), and Tuina and Moxibustion (14–28 days). The majority of data were collected from centers in China. The sample size was moderate, with a predominant focus on middle-aged and elderly patients (average age between 45 and 60 years), and a male-dominated sex ratio. Follow-up durations varied across studies, with common periods ranging from 1 to 12 weeks. Detailed information on all included studies is presented in Supplementary Tables S4, S5.

### 3.2 Evaluation of literature quality

The RoB2 tool was employed to evaluate the risk of bias in 50 RCTs included in this analysis. Of these, two studies were rated as low risk, 29 were classified as having some concerns, and 19 were deemed high risk, indicating a moderate overall quality of the evidence. In the randomization process domain, numerous studies failed to report the method used to generate random sequences, raising concerns about 47 studies. Regarding deviations from intended interventions, 48 studies were assessed as having some concerns due to limitations in blinding procedures for traditional Chinese medicine interventions. Similarly, in the domain of missing outcome data, uncertainty arose from the lack of clear reporting on participant dropouts or withdrawals, with 43 studies rated as having some concerns. In terms of outcome measurement, the use of subjective outcome measures in several studies led to 29 studies being classified as having some concerns. Finally, in the selective reporting domain, a number of studies did not adequately mitigate the risk of selective reporting, with 48 studies being assessed as having some concerns (Supplementary Figure S1).

### 3.3 Consistency

**Figure 2:**
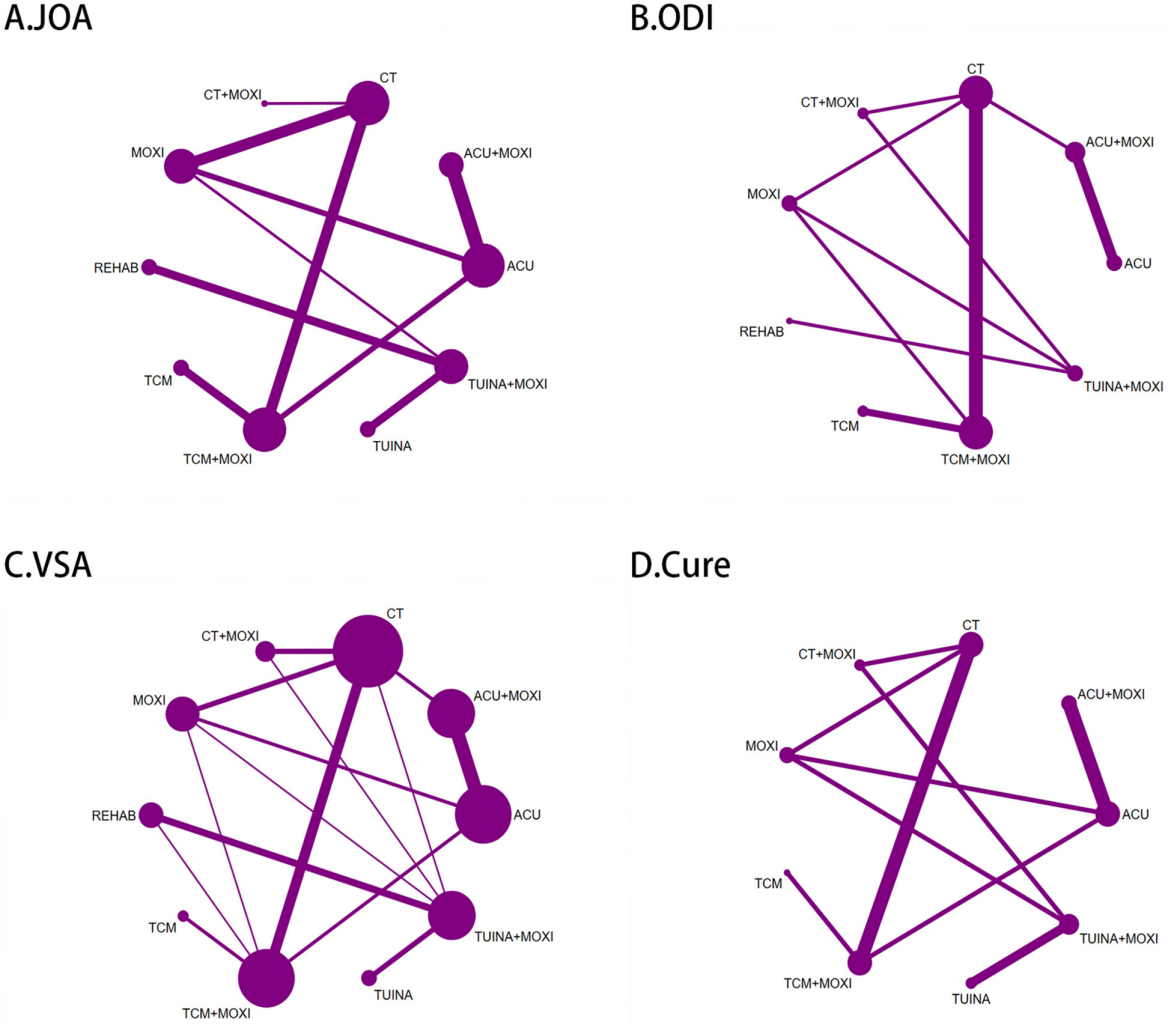
Network plot of interventions included in the primary analysis of patients with LDH. **(A)** Network plot of interventions included in the primary analysis for JOA score in patients with LDH. **(B)** Network plot of interventions included in the primary analysis for ODI score in patients with LDH. **(C)** Network plot of interventions included in the primary analysis for VAS score in patients with LDH. **(D)** Network plot of interventions included in the primary analysis for cure rate in patients with LDH.

In the primary analysis, the network of outcomes—comprising ODI score group, VAS score group, JOA score group, and cure rate group—was characterized by closed loops (Figure 2). A global inconsistency test was conducted, with results indicating *P*-values greater than 0.05, signifying the absence of significant global inconsistency (Supplementary Table S6). To assess local inconsistency, the node-splitting method was applied, and the *P*-values for all comparison nodes were similarly greater than 0.05 (Supplementary Tables S7–S10). Furthermore, a loop inconsistency analysis was performed to evaluate the alignment between direct and indirect evidence. The findings revealed that the credible intervals for the loop IFs consistently spanned zero, indicating strong overall consistency within the network model (Supplementary Tables S11–S14). Therefore, under the assumption of consistency, a consistency model was adopted for the NMA of the primary outcomes.

#### 3.3.1 JOA score

**Figure 3:**
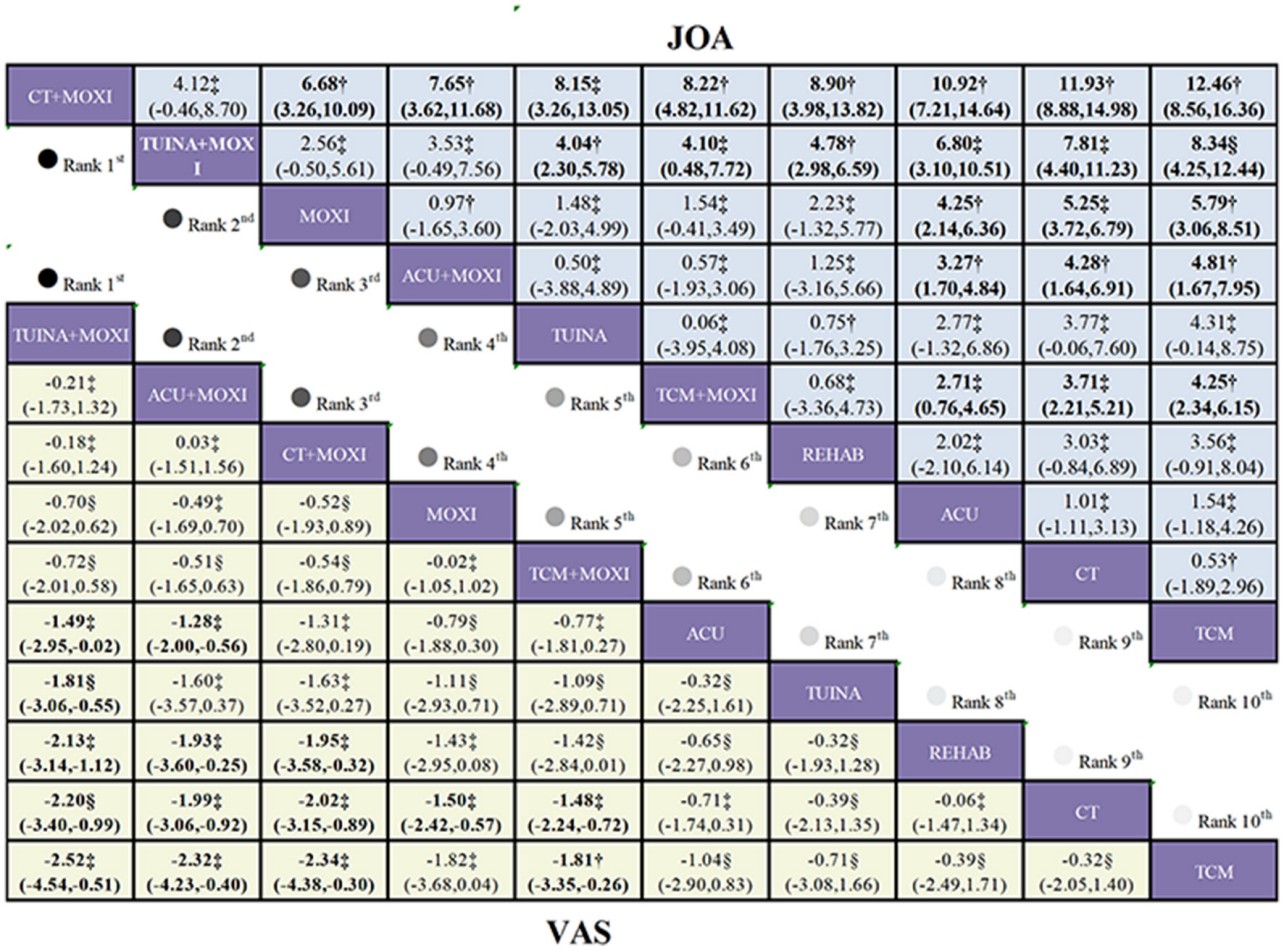
League table of treatment comparisons in the primary analysis of LDH. League table of the NMA for the comparative effectiveness of moxibustion-based combination therapies for lumbar disc herniation (ranked according to SUCRA values; outcomes: VAS score and JOA score). Interventions are ordered according to their SUCRA rankings (from highest to lowest probability of being the best treatment). Comparisons should be read from left to right. Comparative estimates are presented at the intersection between the treatment defined by the column and the treatment defined by the row. Continuous outcomes (VAS score and JOA score) are reported as MDs with corresponding 95% CIs. Lower-left triangle (VAS score): MD < 0 favors the treatment defined by the column (greater reduction in pain intensity), Upper-right triangle (JOA score): MD >0 favors the treatment defined by the column (greater improvement in functional status). Bold numbers indicate statistically significant differences (95% CI does not cross 0). CT serves as the reference comparator within the network; all relative effects should be interpreted according to the treatments specified by the corresponding row and column. Certainty of the evidence for the two primary outcomes, assessed using the GRADE approach, is indicated as follows: *, high certainty of evidence; †, moderate certainty of evidence; ‡, low certainty of evidence; §, very low certainty of evidence.

A total of 28 studies were included in the analysis, assessing the impact of 10 interventions on lumbar function and clinical outcomes in 2,356 patients with LDH. Moderate-quality evidence (Figure 3) indicates that, compared to CT, CT+MOXI (MD = 11.93, 95% CI 8.88 to 14.98) and ACU+MOXI (MD = 4.28, 95% CI 1.64 to 6.91) significantly improved the JOA score. Low-quality evidence suggests that, relative to CT, TCM+MOXI (MD = 3.71, 95% CI 2.21 to 5.21), MOXI (MD = 5.25, 95% CI 3.72 to 6.79), and TUINA+MOXI (MD = 7.81, 95% CI 4.4 to 11.23) also significantly increased the JOA score.

Based on the SUCRA results, MOXI+CT emerged as the top intervention, with a score of 99.6%, followed by TUINA+MOXI at 88.1%, reflecting their relative superiority in overall efficacy. However, probability analysis indicates that MOXI+CT is most likely to be the most effective intervention, with a highest probability of 96.1%. Therefore, MOXI+CT may represent the optimal treatment choice. Furthermore, the average ranking analysis reinforces the relative advantages of MOXI+CT and TUINA+MOXI, with MOXI ranking third with the lowest average rank of 3.6 (Supplementary Table S15; Supplementary Figure S2).

#### 3.3.2 ODI score

**Figure 4:**
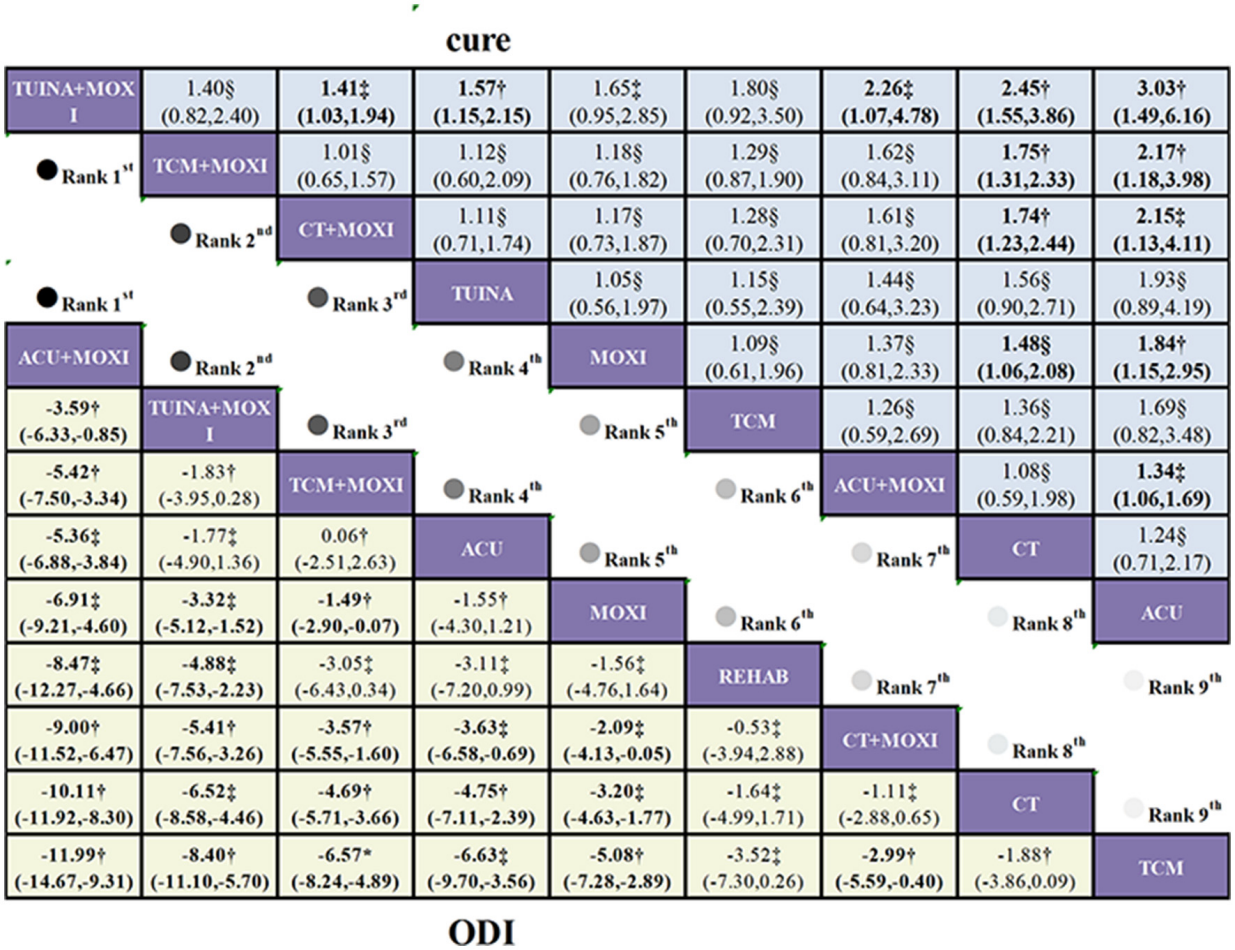
League table of treatment comparisons in the primary analysis of LDH. League table of the NMA for the comparative effectiveness of moxibustion-based combination therapies for lumbar disc herniation (ranked according to SUCRA values; outcomes: ODI score and cure rate). Interventions are ordered according to their SUCRA rankings (from highest to lowest probability of being the best treatment). Comparisons should be read from left to right. Comparative estimates are presented at the intersection between the treatment defined by the column and the treatment defined by the row. Continuous outcomes (ODI score) are reported as MDs, and dichotomous outcomes (cure rate) are reported as risk ratios (RRs), each with corresponding 95% CIs. Lower-left triangle (ODI score): MD < 0 favors the treatment defined by the column (greater improvement in functional disability).Upper-right triangle (cure rate): RR > 1 favors the treatment defined by the column. Bold numbers indicate statistically significant differences (95% CI does not cross 0 for MD or 1 for RR). CT serves as the reference comparator within the network; all relative effects are interpreted according to the treatments specified by the corresponding row and column. Certainty of the evidence for the two primary outcomes, assessed using the GRADE approach. *High certainty of evidence; ^†^moderate certainty of evidence; ^‡^low certainty of evidence; ^§^very low certainty of evidence.

This study aggregates data from 16 trials examining 9 distinct interventions to compare functional improvements in 1,384 patients with LDH. Moderate-quality evidence (Figure 4) shows that, compared to CT, ACU+MOXI (MD = −10.11, 95% CI: −11.92 to −8.3), TCM+MOXI (MD = −4.69, 95% CI: −5.17 to −3.66), and ACU (MD = −4.75, 95% CI: −7.11 to −2.39) all lead to significant reductions in ODI scores. Additionally, lower-quality evidence suggests that, in comparison to CT, TUINA+MOXI (MD = −6.52, 95% CI: −8.58 to −4.46) and MOXI (MD = −3.2, 95% CI: −4.63 to −1.77) also result in significant improvements in ODI scores.

According to the SUCRA results, ACU+MOXI leads the rankings with a score of 99.9%, followed by TUINA+MOXI at 85.2%, highlighting their relative superiority in overall efficacy. Probabilistic analysis further indicates that ACU+MOXI is the most likely to be the most effective intervention, with a highest probability of 99.6%. Thus, ACU+MOXI may be the most optimal treatment option. Additionally, the average rank analysis reinforces the relative advantages of ACU+MOXI and TUINA+MOXI, with ACU achieving the lowest average rank of 3.6, positioning it third (Supplementary Table S16; Supplementary Figure S3).

#### 3.3.3 VAS scores

This study integrates data from 40 trials encompassing 10 distinct interventions, evaluating the efficacy of these treatments in alleviating low back and leg pain in 3,620 patients with LDH. Low-quality evidence (Figure 3) suggests that, compared to CT, ACU+MOXI (MD = −1.99, 95% CI: −3.06 to −0.92), TCM+MOXI (MD = −1.48, 95% CI: −2.24 to −0.72), and CT+MOXI (MD = −2.02, 95% CI: −3.15 to −0.89) all lead to significant reductions in VAS scores. Additionally, very low-quality evidence indicates that, in comparison to CT, TUINA+MOXI (MD = −2.2, 95% CI: −3.4 to −0.99) also results in significant VAS score reductions.

The SUCRA results indicate that TUINA+MOXI leads with a score of 87.6%, followed by ACU+MOXI at 82.6%, demonstrating their relative superiority in overall efficacy. However, probabilistic analysis suggests that TUINA+MOXI is the most likely to be the most effective intervention, with the highest probability of 41.2%, closely followed by ACU+MOXI and CT+MOXI. Consequently, ACU+MOXI may represent the more optimal treatment option. Additionally, the average rank analysis further reinforces the relative advantage of TUINA+MOXI, with both ACU+MOXI and CT+MOXI achieving the lowest average rank of 2.6, tied for third (Supplementary Table S17; Supplementary Figure S4).

#### 3.3.4 Cure rate

This study aggregates data from 15 trials evaluating 9 distinct interventions, encompassing a total of 1,206 patients with LDH, of whom 567 were cured, yielding an approximate cure rate of 47%. Moderate-quality evidence (Figure 4) demonstrates that, compared to CT, TUINA+MOXI (RR = 2.45, 95% CI: 1.55 to 3.86), TCM+MOXI (RR = 1.75, 95% CI: 1.31 to 2.33), and CT+MOXI (RR = 1.44, 95% CI: 1.23 to 2.44) all significantly enhance cure rates. Additionally, very low-quality evidence suggests that, in comparison to CT, MOXI (RR = 1.48, 95% CI: 1.06 to 2.08) also significantly improves cure rates.

According to the SUCRA results, TUINA+MOXI ranks highest with a score of 97.2%, followed by TCM+MOXI at 72.7%, demonstrating their relative superiority in overall efficacy. However, probabilistic analysis suggests that TUINA+MOXI is most likely to be the most effective intervention, with the highest probability of 85.9%. Therefore, TUINA+MOXI may represent the optimal treatment option. Additionally, the average rank analysis further supports the relative advantage of TUINA+MOXI over TCM+MOXI, with CT+MOXI achieving the lowest average rank of 3.4, tied for third (Supplementary Table S18; Supplementary Figure S5).

#### 3.3.5 Adverse events

Although 50 RCTs were included in this review, only two reported adverse events. In one study, one patient experienced mild abdominal cramping, two developed skin redness and swelling, and two developed rash. Another study reported one case of skin allergy. Given the limited availability of safety data, a systematic NMA of adverse events was not feasible.

### 3.4 Subgroup analysis

**Figure 5:**
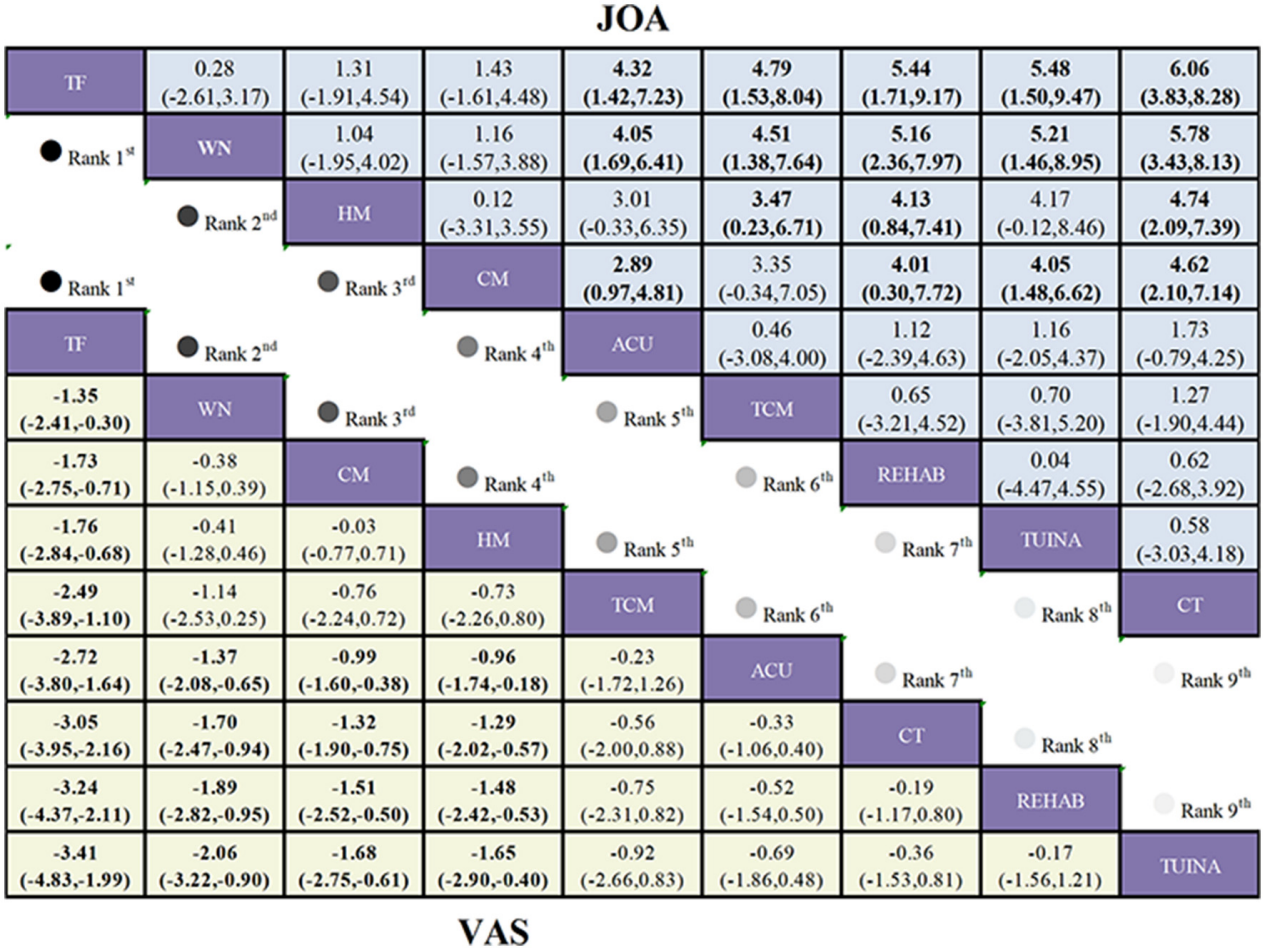
League table of treatment comparisons in the subgroup analysis of LDH. League table of the NMA for the comparative effectiveness of moxibustion-based combination therapies for lumbar disc herniation (ranked according to SUCRA values; outcomes: VAS score and JOA score). Interventions are ordered according to their SUCRA rankings (from highest to lowest probability of being the best treatment). Comparisons should be read from left to right. Comparative estimates are presented at the intersection between the treatment defined by the column and the treatment defined by the row. Continuous outcomes (VAS score and JOA score) are reported as MDs with corresponding 95% CIs. Lower-left triangle (VAS score): MD < 0 favors the treatment defined by the column (greater reduction in pain intensity), Upper-right triangle (JOA score): MD >0 favors the treatment defined by the column (greater improvement in functional status). Bold numbers indicate statistically significant differences (95% CI does not cross 0). CT serves as the reference comparator within the network; all relative effects should be interpreted according to the treatments specified by the corresponding row and column. Certainty of the evidence for the two primary outcomes, assessed using the GRADE approach.

**Figure 6:**
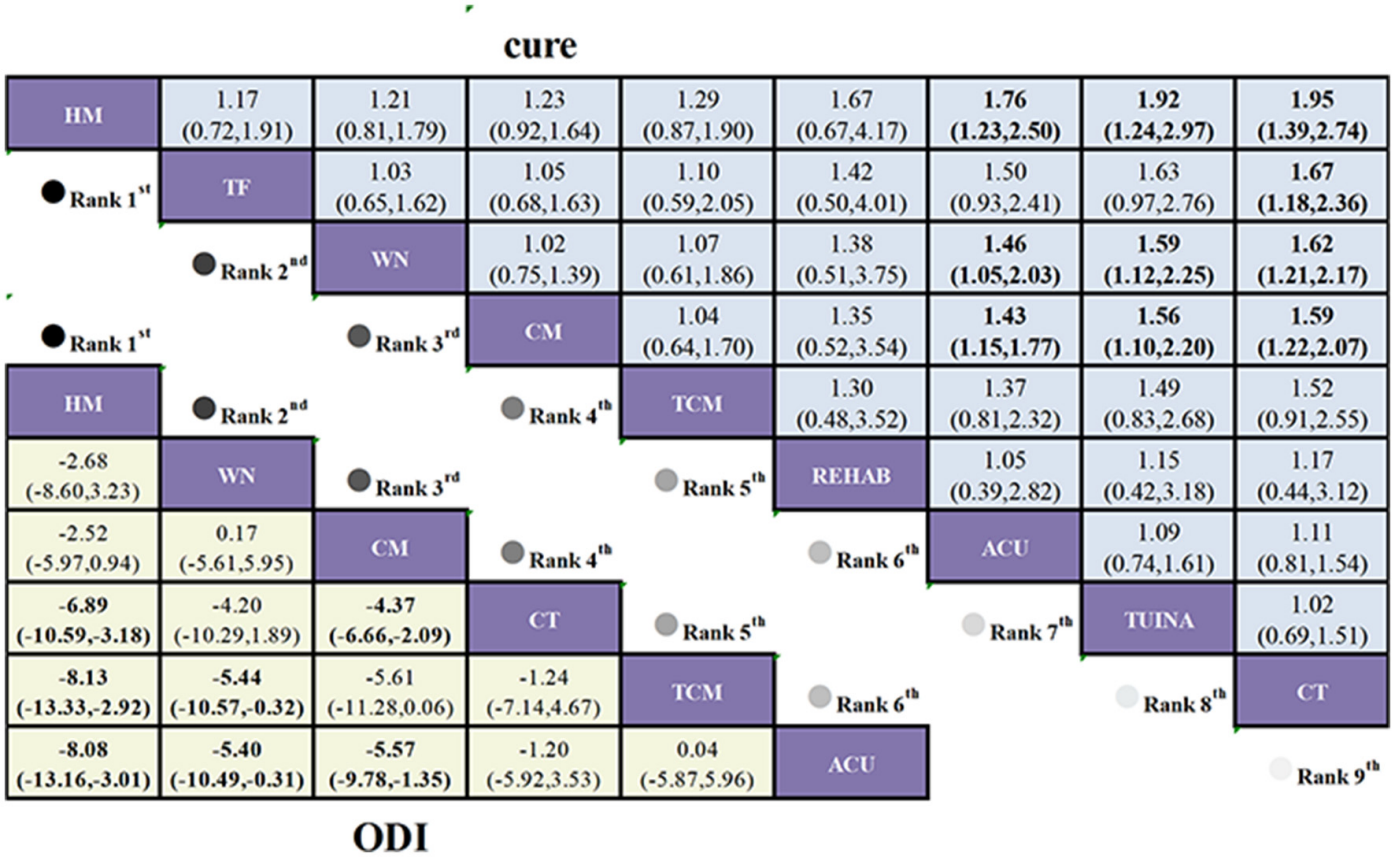
League table of treatment comparisons in the subgroup analysis of lumbar disc herniation. League table of the NMA for the comparative effectiveness of moxibustion-based combination therapies for lumbar disc herniation (ranked according to SUCRA values; outcomes: ODI score and cure rate). Interventions are ordered according to their SUCRA rankings (from highest to lowest probability of being the best treatment). Comparisons should be read from left to right. Comparative estimates are presented at the intersection between the treatment defined by the column and the treatment defined by the row. Continuous outcomes (ODI score) are reported as MDs, and dichotomous outcomes (cure rate) are reported as risk ratios (RRs), each with corresponding 95% CIs. Lower-left triangle (ODI score): MD < 0 favors the treatment defined by the column (greater improvement in functional disability).Upper-right triangle (cure rate): RR > 1 favors the treatment defined by the column. Bold numbers indicate statistically significant differences (95% CI does not cross 0 for MD or 1 for RR). CT serves as the reference comparator within the network; all relative effects are interpreted according to the treatments specified by the corresponding row and column. Certainty of the evidence for the two primary outcomes, assessed using the GRADE approach.

To determine the efficacy ranking of specific combination regimens, combination therapies were further stratified into conventional moxibustion-based, warm needling moxibustion-based, thunder-fire moxibustion-based, and heat-sensitive moxibustion-based regimens, followed by subgroup analyses. In the subgroup analyses of ODI score group, VAS score group, JOA score group, and cure rate group, closed loops were identified within the networks of these outcomes (Supplementary Figures S6–S9). Global inconsistency tests, node splitting methods, and loop inconsistency assessments all indicated satisfactory consistency (Supplementary Tables S19–S31; Supplementary Figures S10–S13). In the ODI score analysis, compared to CT, HM combined regimen (MD = −6.89, 95% CI: −10.53 to −3.18) and CM combined regimen (MD = −4.37, 95% CI: −6.66 to −2.09) significantly reduced ODI scores. In the JOA score analysis, compared to CT, TF combined regimen (MD = 6.06, 95% CI: 3.83 to 8.28), WN combined regimen (MD = 5.78, 95% CI: 3.43 to 8.13), and HM combined regimen (MD = 4.74, 95% CI: 2.09 to 7.39) significantly increased JOA scores. In the VAS score analysis, compared to CT, TF combined regimen (MD = −3.05, 95% CI: −3.19 to −2.16), WN combined regimen (MD = −1.70, 95% CI: −2.47 to −0.94), and CM combined regimen (MD = −1.32, 95% CI: −1.90 to −0.75) significantly reduced VAS scores. Regarding cure rates, compared to CT, HM combined regimen (RR = 1.95, 95% CI: 1.39 to 2.74), TF combined regimen (RR = 1.67, 95% CI: 1.18 to 2.36), and WN combined regimen (RR = 1.62, 95% CI: 1.21 to 2.17) significantly increased cure rates (Figures 5, 6).

### 3.5 Meta-regression, sensitivity analysis, and publication bias

The distribution of key baseline characteristics was well-balanced across treatment groups, with no significant differences identified. In both the primary and subgroup analyses, meta-regression revealed that factors such as average intervention duration, age, and disease duration had no substantial effect on treatment outcomes, thereby supporting the validity of the transitivity assumption (Supplementary Tables S32–S49).

This study conducted a leave-one-out sensitivity analysis to assess the influence of individual studies on the estimated network effects. The findings demonstrate that the relative effect direction of all moxibustion combination protocols vs. CT remained consistent, regardless of which study was excluded. The variability in effect size magnitudes was minimal, with 95% CIs showing substantial overlap. Additionally, the statistical significance of the results remained unchanged, further indicating that the primary analysis is robust (Supplementary Tables S50–S57).

Funnel plots were generated for each outcome measure to evaluate the potential for small sample effects and publication bias. The analysis revealed that the scatter distribution of the plots was nearly symmetrical, with no discernible systematic distortion or extreme outliers, indicating a minimal risk of publication bias (Supplementary Figures S14–S21).

### 3.6 Evidence grading

The certainty of the network estimates was assessed using the GRADE framework, with additional evaluation via CINeMA (Supplementary Tables S58–S61). Across the four network comparisons for all outcomes, the distribution of evidence certainty was as follows: one comparison with high certainty, 43 comparisons with moderate certainty, 74 comparisons with low certainty, and 44 comparisons with very low certainty. Comparisons with higher certainty were generally supported by multiple direct head-to-head trials, with CIs not crossing the threshold of no effect or the MID. In contrast, comparisons with lower or very low certainty were primarily influenced by sparse networks or reliance on indirect evidence, and were associated with wide CIs or potential small sample effects.

## 4 Discussion

### 4.1 Main findings

This study employs a NMA to systematically integrate both direct and indirect evidence, assessing the relative efficacy and hierarchical ranking of moxibustion combined with various adjunctive therapies for treating LDH. The results offer a robust evidence-based foundation for optimizing the integration of traditional Chinese medicine interventions in clinical practice.

This systematic review evaluated 50 RCTs, encompassing 4,399 patients with LDH and 10 distinct intervention types. In terms of pain relief, robust evidence, albeit with low to very low certainty, suggests that TUINA+MOXI and ACU+MOXI are among the most effective therapies for alleviating pain in these patients, as reflected in VAS scores, when compared to CT. Regarding functional recovery, evidence of low to moderate certainty suggests that, compared to CT, both ACU+MOXI and TUINA+MOXI are among the most effective therapies for improving function in patients with LDH, as assessed by the ODI score. Similarly, low to moderate certainty evidence indicates that, when compared to CT, both CT+MOXI and TUINA+MOXI are among the most effective interventions for enhancing functional recovery, as reflected in the JOA score. With regard to patient recovery, moderate-certainty evidence suggests that, compared to CT, both TUINA+MOXI and TCM+MOXI are among the most effective treatments for promoting recovery in patients with LDH. Consequently, in terms of pain relief, functional recovery, and overall patient recovery, TUINA+MOXI and ACU+MOXI are among the most effective therapeutic options for LDH. In the subgroup analysis, both the TF combined regimen and the WN combined regimen are among the most effective treatments for alleviating pain in patients with LDH, as evidenced by VAS scores, when compared to CT. Regarding functional recovery, the ODI is a patient-reported measure of lumbar pain disability, primarily assessing the degree of limitation in daily activities (26); In contrast, the JOA score is a comprehensive clinical evaluation tool, emphasizing the physician's assessment of the patient's symptoms, signs, and overall functional status (27). Thus, in the subgroup analysis of functional recovery, the JOA score was selected as the primary outcome measure. Compared to CT, both the TF combined regimen and the WN combined regimen emerge as some of the most effective treatments for enhancing functional recovery in patients with LDH. In terms of patient recovery, both the HM combined regimen and the WN combined regimen are among the most effective treatments for achieving recovery in patients with LDH, as compared to CT. Therefore, considering pain relief, functional recovery, and overall patient recovery, TF and WN within the moxibustion combined regimens stand out as some of the most effective therapeutic options for treating LDH.

### 4.2 Results analysis

In the primary analysis of this NMA study, TUINA+MOXI was identified as one of the most effective interventions, yielding the most favorable overall therapeutic outcomes. Moxibustion's thermal stimulation activates receptors at acupoints, improves local microcirculation, and induces the expression of heat shock proteins, contributing to analgesia and tissue protection. The near-infrared radiation released during combustion may also modulate neurohumoral regulation through both thermal and non-thermal biological effects. Additionally, mugwort leaves and their combustion products contain a range of compounds with antioxidant and anti-inflammatory properties. Therefore, moxibustion likely exerts its therapeutic effects through a synergistic mechanism involving thermal effects, radiation effects, and pharmacological actions (28). Tuina, a traditional Chinese therapy, operates through various mechanisms, including the modulation of muscle tone, enhancement of blood circulation, pain relief, and joint mobilization (29). While tuina is particularly effective in relieving pain and improving mobility via mechanical and neural pathways, its capacity to systematically regulate inflammatory mediators may be limited (30). In contrast, moxibustion has demonstrated the ability to downregulate levels of several pro-inflammatory cytokines (31). Tuina and moxibustion exhibit a complementary relationship in their therapeutic effects. Tuina primarily targets biomechanical adjustments and functional improvement, whereas moxibustion focuses on thermal anti-inflammatory effects, pain relief, and the regulation of microcirculation. Together, these treatments may theoretically form a continuous intervention pathway—“mechanical adjustment, inflammation control, and functional restoration”—which could potentially enhance synergistic effects in pain relief and functional recovery. However, these mechanisms remain speculative and require further investigation. As expected, TUINA+MOXI consistently shows beneficial effects across functional recovery, pain relief, and overall patient recovery. ACU+MOXI have shown positive effects. ACU encompasses acupuncture, electroacupuncture, and auricular acupuncture. The analgesic effects of acupuncture and electroacupuncture are primarily mediated through the activation of the central endogenous opioid system (32), Electroacupuncture produces a synergistic anti-inflammatory effect through multiple targets (33), while auricular acupuncture may indirectly affect pain perception, stress responses, and associated symptoms by regulating vagal nerve activity and autonomic nervous system balance (34). When combined with MOXI, ACU produces analgesic and regulatory effects through the nervous system, while moxibustion offers pain relief, anti-inflammatory, and antioxidant benefits through its thermal, radiative, and pharmacological mechanisms. Thus, ACU+MOXI is theoretically expected to provide a more comprehensive effect on pain relief and the improvement of associated symptoms. However, these mechanisms remain largely speculative and require further investigation. As anticipated, ACU+MOXI consistently shows a stable advantage in functional recovery, pain relief, and overall recovery outcomes.

In the subgroup analysis of this NMA study, TF is shown to be an effective treatment for LDH. Its therapeutic mechanisms are primarily attributed to both thermal and infrared radiation effects. The infrared radiation generated during combustion penetrates soft tissues, promoting local blood circulation and facilitating the removal of inflammatory pain-mediating substances, which ultimately contributes to its analgesic action (35). Thunder-fire moxibustion has demonstrated the ability to reduce the release of inflammatory cytokines and mitigate the inflammatory stimulation of nerve roots, thereby exerting both anti-inflammatory and tissue repair-promoting effects. Furthermore, through its synergistic actions of warming the meridians, dispersing cold, and enhancing blood circulation, TF thunder-fire moxibustion improves local microcirculation. This, in turn, aids in relieving muscle spasms and tissue edema, leading to improved lumbar spine function (36). As a result, thunder-fire moxibustion produces superior therapeutic outcomes. WN or acupuncture can alleviate pain by inhibiting central sensory input, while concurrently harnessing the immunomodulatory effects of moxibustion heat and infrared radiation to reduce serum levels of inflammatory cytokines. Furthermore, it promotes circulation and improves blood flow, thereby exerting a synergistic effect through multiple mechanisms (37). Consequently, warm acupuncture is anticipated to produce more favorable outcomes. As expected, the subgroup analysis supports that both the HM combined regimen and the WN combined regimen are effective therapeutic modalities for the treatment of LDH.

### 4.3 Implications for clinical practice and research

The results of this study underscore the promising potential of moxibustion combined therapy in the management of LDH. These findings suggest that its therapeutic benefits may not be limited to adjunctive care, but could establish it as a central intervention strategy with considerable promise for broader clinical implementation and adoption. While current clinical guidelines predominantly emphasize pharmacological treatments, traction, or isolated physical therapy interventions, there is a notable lack of systematic evaluations of moxibustion-based combination therapies. This study, however, leverages NMA to provide robust evidence through both direct and indirect comparisons, establishing a more hierarchical, evidence-driven framework for optimizing treatment strategies in LDH. Given its exceptional efficacy in pain relief, functional recovery, and patient recovery rates, along with a high level of acceptance of TUINA, TUINA+MOXI is particularly well-suited for incorporation into primary care settings and community health programs. Moreover, the effects of different combination therapies on clinical outcomes may vary, underscoring the importance of personalized intervention strategies. Among these combinations, TUINA+MOXI or ACU+MOXI may be prioritized for pain management in acute cases, where pain is severe, daily activities are significantly restricted, or there is a desire to reduce dependence on analgesics. This recommendation is based on the relative benefits observed with these combinations in terms of VAS scores from the network comparison; however, due to limitations in study quality and heterogeneity, the evidence level is classified as very low or low. Treatment decisions should involve discussions with patients regarding pain intensity, previous treatment responses, time commitment, and acceptable treatment frequency, with the understanding that the efficacy of these approaches still requires validation in higher-quality studies. In subacute and chronic stages, where the primary goals of rehabilitation are to restore functions such as walking, bending, and tolerance for prolonged sitting, TUINA+MOXI demonstrates advantages in both ODI and JOA scores and should thus be considered. Given the low level of evidence, it is essential to perform dynamic assessments and make necessary adjustments during treatment. In clinical scenarios aimed at improving overall outcomes, both TUINA+MOXI and TCM+MOXI represent viable options, with concurrent reference to VAS, ODI, and JOA score scales.

### 4.4 Comparison with previous studies

This study diverges from the approach of Wang Yang and colleagues in terms of intervention selection (12). Wang Yang and colleagues performed a subgroup analysis based on whether moxibustion was combined with other treatment modalities. However, their comparison was largely confined to pairwise comparisons between moxibustion and a single control, which precluded direct comparisons among various combination therapies. This limitation made it difficult to determine which combination therapy was superior. In contrast, our study integrates multiple moxibustion-based combination therapies within a unified network framework, allowing for direct comparisons of the relative efficacy of different “moxibustion + adjunctive” treatment regimens. Additionally, we conducted subgroup analyses based on specific moxibustion techniques, comparing Lei huo moxibustion, warm acupuncture, thermosensitive moxibustion, and traditional moxibustion as distinct nodes. This approach enabled us to explore the potential advantages of each moxibustion method within combination therapies and to provide more nuanced evidence to inform clinical decision-making. This study incorporates a broader array of outcome measures, including ODI, JOA, VAS scores, and the rate of cure. In contrast to the approach taken by Wang Yang and colleagues, our study includes the ODI score, offering a more comprehensive view of functional assessment. While the therapeutic efficacy reported by Wang Yang et al. reflects treatment outcomes, it primarily focuses on the degree of symptom improvement, without clearly distinguishing between complete recovery and partial improvement. In contrast, our study explicitly defines the criteria for “cure,” thereby enhancing the clinical applicability of the findings. Regarding “effect size and certainty,” we not only conducted SUCRA rankings but also applied the GRADE classification, enabling a clearer delineation of the level of evidence.

### 4.5 Strengths and limitations

This study followed stringent methodological guidelines. The ROB2 tool was utilized to evaluate the risk of bias in the included studies, while the GRADE framework was applied to assess the certainty of the evidence. Subgroup analyses were performed based on various moxibustion techniques to identify potential differences between them. Furthermore, sensitivity analyses and network meta-regression were conducted to evaluate the robustness of the primary findings.

Nonetheless, several limitations should be acknowledged. Given the nature of moxibustion, blinding of both participants and researchers is often impractical. As a result, a considerable proportion of the included studies were classified as having some bias (58%) or high risk (36%), which could potentially affect the reliability of the effect estimates—especially in comparisons based on a limited number of studies. Although no evident funnel plot asymmetry was observed, the small number of studies in most comparisons restricts the ability to fully exclude publication bias. Second, only two studies reported adverse events, providing insufficient safety data for NMA; adverse events were therefore described narratively. Furthermore, follow-up duration was inconsistent across studies and generally limited to 1 to 12 weeks. Given the relatively high recurrence rate of LDH, these findings reflect only short-term therapeutic effects, with medium- and long-term efficacy remaining unclear. Finally, since this research focused on traditional Chinese therapeutic methods with participants exclusively from China, the generalizability of the findings to other countries remains uncertain and warrants further investigation.

## 5 Conclusion

This large-scale NMA of RCTs provides very low to moderate evidence supporting the effectiveness of TUINA+MOXI in treating patients with LDH. The intervention demonstrates significant benefits in pain relief, functional recovery, and patient cure rates. However, as all included studies were conducted in China, future research should prioritize large-scale, high-quality RCTs with standardized intervention protocols to confirm and expand upon these findings, during long-term follow-up after treatment completion.

## Data Availability

The original contributions presented in the study are included in the article/Supplementary material, further inquiries can be directed to the corresponding author.
