# Supplementary material for: Comparative effectiveness of moxibustion-based combination therapies for lumbar disc herniation: a systematic review and network meta-analysis of 50 randomized trials
Source: Front Neurol. 2026 May 14;17:1809677. doi: 10.3389/fneur.2026.1809677 (PMC13215928; doi:10.3389/fneur.2026.1809677)
Supplement: Supplementary file 1 [file Table_1.docx]

Table S1 PRISMA NMA Checklist of Items to Include When Reporting a Systematic Review Involving a Network Meta-analysis

| Section/Topic | Item # | Checklist Item | Reported on Page # |
| --- | --- | --- | --- |
| TITLE |  |  |  |
| Title | 1 | Identify the report as a systematic review *incorporating*  anetwork meta-analysis (or related form of meta-analysis). |  |
|  |  |  |  |
| ABSTRACT |  |  |  |
| Structured summary | 2 | Provide a structured summary including, as applicable:  **Background:** main objectives  **Methods:** data sources; study eligibility criteria, participants, and interventions; study appraisal; and *synthesis methods, such as network meta-analysis.*  **Results:** number of studies and participants identified; summary estimates with corresponding confidence/credible intervals; treatment rankings may also be discussed. Authors may choose to summarize pairwise comparisons against a chosen treatment included in their analyses for brevity.  **Discussion/Conclusions:** limitations; conclusions and implications of findings.  **Other:** systematic review registration number with registry name. |  |
|  |  |  |  |
| INTRODUCTION |  |  |  |
| Rationale | 3 | Describe the rationale for the review in the context of what is already known*, including mention of why a network meta-analysis has been conducted.* |  |
| Objectives | 4 | Provide an explicit statement of questions being addressed, with reference to participants, interventions, comparisons, outcomes, and study design (PICOS). |  |
|  |  |  |  |
| METHODS |  |  |  |
| Protocol and registration | 5 | Indicate whether a review protocol exists and if and where it can be accessed (e.g., Web address); and, if available, provide registration information, including registration number. |  |
| Eligibility criteria | 6 | Specify study characteristics (e.g., PICOS, length of follow-up) and report characteristics (e.g., years considered, language, publication status) used as criteria for eligibility, giving rationale. *Clearly describe eligible treatments included in the treatment network, and note whether any have been clustered or merged into the same node (with justification).* |  |
| Information sources | 7 | Describe all information sources (e.g., databases with dates of coverage, contact with study authors to identify additional studies) in the search and date last searched. |  |
| Search | 8 | Present full electronic search strategy for at least one database, including any limits used, such that it could be repeated. |  |
| Study selection | 9 | State the process for selecting studies (i.e., screening, eligibility, included in systematic review, and, if applicable, included in the meta-analysis). |  |
| Data collection process | 10 | Describe method of data extraction from reports (e.g., piloted forms, independently, in duplicate) and any processes for obtaining and confirming data from investigators. |  |
| Data items | 11 | List and define all variables for which data were sought (e.g., PICOS, funding sources) and any assumptions and simplifications made. |  |
| Geometry of the network | S1 | Describe methods used to explore the geometry of the treatment network under study and potential biases related to it. This should include how the evidence base has been graphically summarized for presentation, and what characteristics were compiled and used to describe the evidence base to readers. |  |
| Risk of bias within individual studies | 12 | Describe methods used for assessing risk of bias of individual studies (including specification of whether this was done at the study or outcome level), and how this information is to be used in any data synthesis. |  |
| Summary measures | 13 | State the principal summary measures (e.g., risk ratio, difference in means). Also describe the use of additional summary measures assessed, such as treatment rankings and surface under the cumulative ranking curve (SUCRA) values, as well as modified approaches used to present summary findings from meta-analyses. |  |
| Planned methods of analysis | 14 | Describe the methods of handling data and combining results of studies for each network meta-analysis. This should include, but not be limited to:   - Handling of multi-arm trials; - Selection of variance structure; - Selection of prior distributions in Bayesian analyses; and - Assessment of model fit. |  |
| Assessment of Inconsistency | S2 | Describe the statistical methods used to evaluate the agreement of direct and indirect evidence in the treatment network(s) studied. Describe efforts taken to address its presence when found. |  |
| Risk of bias across studies | 15 | Specify any assessment of risk of bias that may affect the cumulative evidence (e.g., publication bias, selective reporting within studies). |  |
| Additional analyses | 16 | Describe methods of additional analyses if done, indicating which were pre-specified. This may include, but not be limited to, the following:   - Sensitivity or subgroup analyses; - Meta-regression analyses; - Alternative formulations of the treatment network; and - Use of alternative prior distributions for Bayesian analyses (if applicable). |  |
| RESULTS† |  |  |  |
| Study selection | 17 | Give numbers of studies screened, assessed for eligibility, and included in the review, with reasons for exclusions at each stage, ideally with a flow diagram. |  |
| Presentation of network structure | S3 | Provide a network graph of the included studies to enable visualization of the geometry of the treatment network. |  |
| Summary of network geometry | S4 | Provide a brief overview of characteristics of the treatment network. This may include commentary on the abundance of trials and randomized patients for the different interventions and pairwise comparisons in the network, gaps of evidence in the treatment network, and potential biases reflected by the network structure. |  |
| Study characteristics | 18 | For each study, present characteristics for which data were extracted (e.g., study size, PICOS, follow-up period) and provide the citations. |  |
| Risk of bias within studies | 19 | Present data on risk of bias of each study and, if available, any outcome level assessment. |  |
| Results of individual studies | 20 | For all outcomes considered (benefits or harms), present, for each study: 1) simple summary data for each intervention group, and 2) effect estimates and confidence intervals. *Modified approaches may be needed to deal with information from larger networks.* |  |
| Synthesis of results | 21 | Present results of each meta-analysis done, including confidence/credible intervals. In larger networks, authors may focus on comparisons versus a particular comparator (e.g. placebo or standard care), with full findings presented in an appendix. League tables and forest plots may be considered to summarize pairwise comparisons. If additional summary measures were explored (such as treatment rankings), these should also be presented. |  |
| Exploration for inconsistency | S5 | Describe results from investigations of inconsistency. This may include such information as measures of model fit to compare consistency and inconsistency models, *P* values from statistical tests, or summary of inconsistency estimates from different parts of the treatment network. |  |
| Risk of bias across studies | 22 | Present results of any assessment of risk of bias across studies for the evidence base being studied. |  |
| Results of additional analyses | 23 | Give results of additional analyses, if done (e.g., sensitivity or subgroup analyses, meta-regression analyses*, alternative network geometries studied, alternative choice of prior distributions for Bayesian analyses,* and so forth). |  |
|  |  |  |  |
| DISCUSSION |  |  |  |
| Summary of evidence | 24 | Summarize the main findings, including the strength of evidence for each main outcome; consider their relevance to key groups (e.g., healthcare providers, users, and policy-makers). |  |
| Limitations | 25 | Discuss limitations at study and outcome level (e.g., risk of bias), and at review level (e.g., incomplete retrieval of identified research, reporting bias). *Comment on the validity of the assumptions, such as transitivity and consistency. Comment on any concerns regarding network geometry (e.g., avoidance of certain comparisons).* |  |
| Conclusions | 26 | Provide a general interpretation of the results in the context of other evidence, and implications for future research. |  |
|  |  |  |  |
| FUNDING |  |  |  |
| Funding | 27 | Describe sources of funding for the systematic review and other support (e.g., supply of data); role of funders for the systematic review. This should also include information regarding whether funding has been received from manufacturers of treatments in the network and/or whether some of the authors are content experts with professional conflicts of interest that could affect use of treatments in the network. | NA |

PICOS = population, intervention, comparators, outcomes, study design.

* Text in italics indicate S wording specific to reporting of network meta-analyses that has been added to guidance from the PRISMA statement.

† Authors may wish to plan for use of appendices to present all relevant information in full detail for items in this section.

| **Table S2.Literature Search Strategy** | |
| --- | --- |
| **Pubmed** | 1 "Intervertebral Disc Displacement"[Mesh] Most Recent "Intervertebral Disc Displacement"[MeSH Terms] 21,413  2 ((((((((((((((((((((((((((((((((((((((((((((((((((((((((((((((("Intervertebral Disc Displacement"[Mesh]) OR (Intervertebral Disc Displacement[Title/Abstract])) OR (Disc Displacement, Intervertebral[Title/Abstract])) OR (Intervertebral Disc Displacements[Title/Abstract])) OR (Protruded Disc[Title/Abstract])) OR (Disc, Protruded[Title/Abstract])) OR (Discs, Protruded[Title/Abstract])) OR (Protruded Discs[Title/Abstract])) OR (Protruded Disk[Title/Abstract])) OR (Disk, Protruded[Title/Abstract])) OR (Protruded Disks[Title/Abstract])) OR (Intervertebral Disk Displacement[Title/Abstract])) OR (Disk Displacement, Intervertebral[Title/Abstract])) OR (Intervertebral Disk Displacements[Title/Abstract])) OR (Disk, Herniated[Title/Abstract])) OR (Herniated Disks[Title/Abstract])) OR (Slipped Disk[Title/Abstract])) OR (Disk, Slipped[Title/Abstract])) OR (Slipped Disks[Title/Abstract])) OR (Disk Prolapse[Title/Abstract])) OR (Disk Prolapses[Title/Abstract])) OR (Prolapse, Disk[Title/Abstract])) OR (Prolapses, Disk[Title/Abstract])) OR (Prolapsed Disk[Title/Abstract])) OR (Disk, Prolapsed[Title/Abstract])) OR (Prolapsed Disks[Title/Abstract])) OR (Herniated Disc[Title/Abstract])) OR (Herniated Discs[Title/Abstract])) OR (Disc, Herniated[Title/Abstract])) OR (Herniated Disk[Title/Abstract])) OR (Slipped Disc[Title/Abstract])) OR (Disc, Slipped[Title/Abstract])) OR (Slipped Discs[Title/Abstract])) OR (Prolapsed Disc[Title/Abstract])) OR (Disc, Prolapsed[Title/Abstract])) OR (Prolapsed Discs[Title/Abstract])) OR (Disc Herniation[Title/Abstract])) OR (Disc Herniations[Title/Abstract])) OR (Herniation, Disc[Title/Abstract])) OR (Intervertebral Disc Herniation[Title/Abstract])) OR (Herniation, Intervertebral Disc[Title/Abstract])) OR (Intervertebral Disc Herniations[Title/Abstract])) OR (Intervertebral Disk Herniation[Title/Abstract])) OR (Herniation, Intervertebral Disk[Title/Abstract])) OR (Intervertebral Disk Herniations[Title/Abstract])) OR (Disk Herniation[Title/Abstract])) OR (Disk Herniations[Title/Abstract])) OR (Herniation, Disk[Title/Abstract])) OR (Intervertebral Disk Protrusion[Title/Abstract])) OR (Disk Protrusion, Intervertebral[Title/Abstract])) OR (Intervertebral Disk Protrusions[Title/Abstract])) OR (Protrusion, Intervertebral Disk[Title/Abstract])) OR (Protrusions, Intervertebral Disk[Title/Abstract])) OR (Intervertebral Disc Protrusion[Title/Abstract])) OR (Disc Protrusion, Intervertebral[Title/Abstract])) OR (Disc Protrusions, Intervertebral[Title/Abstract])) OR (Intervertebral Disc Protrusions[Title/Abstract])) OR (Protrusion, Intervertebral Disc[Title/Abstract])) OR (Disc Protrusion[Title/Abstract])) OR (Disc Protrusions[Title/Abstract])) OR (Protrusion, Disc[Title/Abstract])) OR (Disk Protrusion[Title/Abstract])) OR (Disk Protrusions[Title/Abstract])) OR (Protrusion, Disk[Title/Abstract]) "Intervertebral Disc Displacement"[MeSH Terms] OR "Intervertebral Disc Displacement"[Title/Abstract] OR "disc displacement intervertebral"[Title/Abstract] OR "intervertebral disc displacements"[Title/Abstract] OR "protruded disc"[Title/Abstract] OR "disc protruded"[Title/Abstract] OR "discs protruded"[Title/Abstract] OR "protruded discs"[Title/Abstract] OR "protruded disk"[Title/Abstract] OR "disk protruded"[Title/Abstract] OR "protruded disks"[Title/Abstract] OR "intervertebral disk displacement"[Title/Abstract] OR ("Disk"[All Fields] AND "displacement intervertebral"[Title/Abstract]) OR "intervertebral disk displacements"[Title/Abstract] OR "disk herniated"[Title/Abstract] OR "herniated disks"[Title/Abstract] OR "slipped disk"[Title/Abstract] OR ("Disk"[All Fields] AND "Slipped"[Title/Abstract]) OR "slipped disks"[Title/Abstract] OR "disk prolapse"[Title/Abstract] OR "disk prolapses"[Title/Abstract] OR (("Prolapse"[MeSH Terms] OR "Prolapse"[All Fields] OR "Prolapses"[All Fields] OR "Prolapsed"[All Fields] OR "prolapsing"[All Fields]) AND "Disk"[Title/Abstract]) OR (("Prolapse"[MeSH Terms] OR "Prolapse"[All Fields] OR "Prolapses"[All Fields] OR "Prolapsed"[All Fields] OR "prolapsing"[All Fields]) AND "Disk"[Title/Abstract]) OR "prolapsed disk"[Title/Abstract] OR "disk prolapsed"[Title/Abstract] OR "prolapsed disks"[Title/Abstract] OR "herniated disc"[Title/Abstract] OR "herniated discs"[Title/Abstract] OR "disc herniated"[Title/Abstract] OR "herniated disk"[Title/Abstract] OR "slipped disc"[Title/Abstract] OR "disc slipped"[Title/Abstract] OR "slipped discs"[Title/Abstract] OR "prolapsed disc"[Title/Abstract] OR "disc prolapsed"[Title/Abstract] OR "prolapsed discs"[Title/Abstract] OR "disc herniation"[Title/Abstract] OR "disc herniations"[Title/Abstract] OR "herniation disc"[Title/Abstract] OR "intervertebral disc herniation"[Title/Abstract] OR "herniation intervertebral disc"[Title/Abstract] OR "intervertebral disc herniations"[Title/Abstract] OR "intervertebral disk herniation"[Title/Abstract] OR "herniation intervertebral disk"[Title/Abstract] OR "intervertebral disk herniations"[Title/Abstract] OR "disk herniation"[Title/Abstract] OR "disk herniations"[Title/Abstract] OR "herniation disk"[Title/Abstract] OR "intervertebral disk protrusion"[Title/Abstract] OR ("Disk"[All Fields] AND "protrusion intervertebral"[Title/Abstract]) OR "intervertebral disk protrusions"[Title/Abstract] OR "protrusion intervertebral disk"[Title/Abstract] OR (("Protrusion"[All Fields] OR "Protrusions"[All Fields] OR "protrusive"[All Fields] OR "protrusiveness"[All Fields]) AND "intervertebral disk"[Title/Abstract]) OR "intervertebral disc protrusion"[Title/Abstract] OR ("Disc"[All Fields] AND "protrusion intervertebral"[Title/Abstract]) OR (("Disc"[All Fields] AND ("Protrusion"[All Fields] OR "Protrusions"[All Fields] OR "protrusive"[All Fields] OR "protrusiveness"[All Fields])) AND "Intervertebral"[Title/Abstract]) OR "intervertebral disc protrusions"[Title/Abstract] OR (("Protrusion"[All Fields] OR "Protrusions"[All Fields] OR "protrusive"[All Fields] OR "protrusiveness"[All Fields]) AND "intervertebral disc"[Title/Abstract]) OR "disc protrusion"[Title/Abstract] OR "disc protrusions"[Title/Abstract] OR "protrusion disc"[Title/Abstract] OR "disk protrusion"[Title/Abstract] OR "disk protrusions"[Title/Abstract] OR (("Protrusion"[All Fields] OR "Protrusions"[All Fields] OR "protrusive"[All Fields] OR "protrusiveness"[All Fields]) AND "Disk"[Title/Abstract]) 28,150  3 ( "Moxibustion"[Mesh] OR moxibustion[tiab] OR moxa[tiab] OR "moxa stick"[tiab] OR mugwort[tiab] OR aijiu[tiab] OR "ai jiu"[tiab] OR "Ai Jiu"[tiab] ) OR ( ("mild moxibustion"[tiab] OR "gentle moxibustion"[tiab] OR "suspended moxibustion"[tiab] OR "suspension moxibustion"[tiab] OR "moxa stick"[tiab] OR "moxa-stick"[tiab] OR "stick moxibustion"[tiab] OR "moxa stick moxibustion"[tiab] OR xuanjiu[tiab] OR "xuan jiu"[tiab] OR wenhejiu[tiab] OR "wen he jiu"[tiab] ) OR ("thunder-fire moxibustion"[tiab] OR "thunder fire moxibustion"[tiab] OR "thunder-fire"[tiab] OR leihuo[tiab] OR "lei huo"[tiab] OR "lei-huo"[tiab] ) OR ("heat-sensitive moxibustion"[tiab] OR "heat sensitive moxibustion"[tiab] OR "heat-sensitized moxibustion"[tiab] OR "heat-sensitive acupoint"[tiab] OR "heat sensitive acupoint"[tiab] OR reminjiu[tiab] OR "re min jiu"[tiab] OR "remin jiu"[tiab] ) OR ("indirect moxibustion"[tiab] OR "partitioned moxibustion"[tiab] OR "insulated moxibustion"[tiab] OR "separated moxibustion"[tiab] OR "ginger-separated moxibustion"[tiab] OR "ginger partitioned moxibustion"[tiab] OR "ginger moxibustion"[tiab] OR "salt-separated moxibustion"[tiab] OR "salt partitioned moxibustion"[tiab] OR "salt moxibustion"[tiab] OR gejiangjiu[tiab] OR "ge jiang jiu"[tiab] OR geyanjiu[tiab] OR "ge yan jiu"[tiab] OR "geyan jiu"[tiab] ) OR ("warm-needling moxibustion"[tiab] OR "warm needling moxibustion"[tiab] OR "warm needle moxibustion"[tiab] OR "warm-needling"[tiab] OR "warm needling"[tiab] OR "warm needle"[tiab] OR "warming needle"[tiab] OR wenzhen[tiab] OR "wen zhen"[tiab] OR wenzhenjiu[tiab] OR "wen zhen jiu"[tiab] OR (("Acupuncture Therapy"[Mesh] OR acupuncture[tiab]) AND (moxibustion[tiab] OR moxa[tiab])) ) ) "Moxibustion"[MeSH Terms] OR "Moxibustion"[Title/Abstract] OR "moxa"[Title/Abstract] OR "moxa-stick"[Title/Abstract] OR "mugwort"[Title/Abstract] OR "Ai Jiu"[Title/Abstract] OR "Ai Jiu"[Title/Abstract] OR ("mild moxibustion"[Title/Abstract] OR "suspended moxibustion"[Title/Abstract] OR "suspension moxibustion"[Title/Abstract] OR "moxa-stick"[Title/Abstract] OR "moxa-stick"[Title/Abstract] OR "stick moxibustion"[Title/Abstract] OR "moxa stick moxibustion"[Title/Abstract] OR ("thunder-fire moxibustion"[Title/Abstract] OR "thunder-fire moxibustion"[Title/Abstract] OR "thunder-fire"[Title/Abstract] OR "leihuo"[Title/Abstract] OR "lei-huo"[Title/Abstract] OR "lei-huo"[Title/Abstract]) OR ("heat-sensitive moxibustion"[Title/Abstract] OR "heat-sensitive moxibustion"[Title/Abstract] OR "heat-sensitive acupoint"[Title/Abstract] OR "heat-sensitive acupoint"[Title/Abstract]) OR ("indirect moxibustion"[Title/Abstract] OR "partitioned moxibustion"[Title/Abstract] OR "insulated moxibustion"[Title/Abstract] OR "separated moxibustion"[Title/Abstract] OR "ginger-separated moxibustion"[Title/Abstract] OR "ginger partitioned moxibustion"[Title/Abstract] OR "ginger moxibustion"[Title/Abstract] OR "salt-separated moxibustion"[Title/Abstract] OR "salt partitioned moxibustion"[Title/Abstract] OR "salt moxibustion"[Title/Abstract]) OR ("warm-needling moxibustion"[Title/Abstract] OR "warm-needling moxibustion"[Title/Abstract] OR "warm needle moxibustion"[Title/Abstract] OR "warm-needling"[Title/Abstract] OR "warm-needling"[Title/Abstract] OR "warm needle"[Title/Abstract] OR "warming needle"[Title/Abstract] OR "wenzhen"[Title/Abstract] OR "wen zhen"[Title/Abstract] OR "wenzhenjiu"[Title/Abstract] OR (("Acupuncture Therapy"[MeSH Terms] OR "acupuncture"[Title/Abstract]) AND ("Moxibustion"[Title/Abstract] OR "moxa"[Title/Abstract])))) 5,633  4 (((((((((((((((((((((((((((((((((((((((((((((((((((((((((((((((("Intervertebral Disc Displacement"[Mesh]) OR (Intervertebral Disc Displacement[Title/Abstract])) OR (Disc Displacement, Intervertebral[Title/Abstract])) OR (Intervertebral Disc Displacements[Title/Abstract])) OR (Protruded Disc[Title/Abstract])) OR (Disc, Protruded[Title/Abstract])) OR (Discs, Protruded[Title/Abstract])) OR (Protruded Discs[Title/Abstract])) OR (Protruded Disk[Title/Abstract])) OR (Disk, Protruded[Title/Abstract])) OR (Protruded Disks[Title/Abstract])) OR (Intervertebral Disk Displacement[Title/Abstract])) OR (Disk Displacement, Intervertebral[Title/Abstract])) OR (Intervertebral Disk Displacements[Title/Abstract])) OR (Disk, Herniated[Title/Abstract])) OR (Herniated Disks[Title/Abstract])) OR (Slipped Disk[Title/Abstract])) OR (Disk, Slipped[Title/Abstract])) OR (Slipped Disks[Title/Abstract])) OR (Disk Prolapse[Title/Abstract])) OR (Disk Prolapses[Title/Abstract])) OR (Prolapse, Disk[Title/Abstract])) OR (Prolapses, Disk[Title/Abstract])) OR (Prolapsed Disk[Title/Abstract])) OR (Disk, Prolapsed[Title/Abstract])) OR (Prolapsed Disks[Title/Abstract])) OR (Herniated Disc[Title/Abstract])) OR (Herniated Discs[Title/Abstract])) OR (Disc, Herniated[Title/Abstract])) OR (Herniated Disk[Title/Abstract])) OR (Slipped Disc[Title/Abstract])) OR (Disc, Slipped[Title/Abstract])) OR (Slipped Discs[Title/Abstract])) OR (Prolapsed Disc[Title/Abstract])) OR (Disc, Prolapsed[Title/Abstract])) OR (Prolapsed Discs[Title/Abstract])) OR (Disc Herniation[Title/Abstract])) OR (Disc Herniations[Title/Abstract])) OR (Herniation, Disc[Title/Abstract])) OR (Intervertebral Disc Herniation[Title/Abstract])) OR (Herniation, Intervertebral Disc[Title/Abstract])) OR (Intervertebral Disc Herniations[Title/Abstract])) OR (Intervertebral Disk Herniation[Title/Abstract])) OR (Herniation, Intervertebral Disk[Title/Abstract])) OR (Intervertebral Disk Herniations[Title/Abstract])) OR (Disk Herniation[Title/Abstract])) OR (Disk Herniations[Title/Abstract])) OR (Herniation, Disk[Title/Abstract])) OR (Intervertebral Disk Protrusion[Title/Abstract])) OR (Disk Protrusion, Intervertebral[Title/Abstract])) OR (Intervertebral Disk Protrusions[Title/Abstract])) OR (Protrusion, Intervertebral Disk[Title/Abstract])) OR (Protrusions, Intervertebral Disk[Title/Abstract])) OR (Intervertebral Disc Protrusion[Title/Abstract])) OR (Disc Protrusion, Intervertebral[Title/Abstract])) OR (Disc Protrusions, Intervertebral[Title/Abstract])) OR (Intervertebral Disc Protrusions[Title/Abstract])) OR (Protrusion, Intervertebral Disc[Title/Abstract])) OR (Disc Protrusion[Title/Abstract])) OR (Disc Protrusions[Title/Abstract])) OR (Protrusion, Disc[Title/Abstract])) OR (Disk Protrusion[Title/Abstract])) OR (Disk Protrusions[Title/Abstract])) OR (Protrusion, Disk[Title/Abstract])) AND (( "Moxibustion"[Mesh] OR moxibustion[tiab] OR moxa[tiab] OR "moxa stick"[tiab] OR mugwort[tiab] OR aijiu[tiab] OR "ai jiu"[tiab] OR "Ai Jiu"[tiab] ) OR ( ("mild moxibustion"[tiab] OR "gentle moxibustion"[tiab] OR "suspended moxibustion"[tiab] OR "suspension moxibustion"[tiab] OR "moxa stick"[tiab] OR "moxa-stick"[tiab] OR "stick moxibustion"[tiab] OR "moxa stick moxibustion"[tiab] OR xuanjiu[tiab] OR "xuan jiu"[tiab] OR wenhejiu[tiab] OR "wen he jiu"[tiab] ) OR ("thunder-fire moxibustion"[tiab] OR "thunder fire moxibustion"[tiab] OR "thunder-fire"[tiab] OR leihuo[tiab] OR "lei huo"[tiab] OR "lei-huo"[tiab] ) OR ("heat-sensitive moxibustion"[tiab] OR "heat sensitive moxibustion"[tiab] OR "heat-sensitized moxibustion"[tiab] OR "heat-sensitive acupoint"[tiab] OR "heat sensitive acupoint"[tiab] OR reminjiu[tiab] OR "re min jiu"[tiab] OR "remin jiu"[tiab] ) OR ("indirect moxibustion"[tiab] OR "partitioned moxibustion"[tiab] OR "insulated moxibustion"[tiab] OR "separated moxibustion"[tiab] OR "ginger-separated moxibustion"[tiab] OR "ginger partitioned moxibustion"[tiab] OR "ginger moxibustion"[tiab] OR "salt-separated moxibustion"[tiab] OR "salt partitioned moxibustion"[tiab] OR "salt moxibustion"[tiab] OR gejiangjiu[tiab] OR "ge jiang jiu"[tiab] OR geyanjiu[tiab] OR "ge yan jiu"[tiab] OR "geyan jiu"[tiab] ) OR ("warm-needling moxibustion"[tiab] OR "warm needling moxibustion"[tiab] OR "warm needle moxibustion"[tiab] OR "warm-needling"[tiab] OR "warm needling"[tiab] OR "warm needle"[tiab] OR "warming needle"[tiab] OR wenzhen[tiab] OR "wen zhen"[tiab] OR wenzhenjiu[tiab] OR "wen zhen jiu"[tiab] OR (("Acupuncture Therapy"[Mesh] OR acupuncture[tiab]) AND (moxibustion[tiab] OR moxa[tiab])) ) )) ("Intervertebral Disc Displacement"[MeSH Terms] OR "Intervertebral Disc Displacement"[Title/Abstract] OR "disc displacement intervertebral"[Title/Abstract] OR "intervertebral disc displacements"[Title/Abstract] OR "protruded disc"[Title/Abstract] OR "disc protruded"[Title/Abstract] OR "discs protruded"[Title/Abstract] OR "protruded discs"[Title/Abstract] OR "protruded disk"[Title/Abstract] OR "disk protruded"[Title/Abstract] OR "protruded disks"[Title/Abstract] OR "intervertebral disk displacement"[Title/Abstract] OR ("Disk"[All Fields] AND "displacement intervertebral"[Title/Abstract]) OR "intervertebral disk displacements"[Title/Abstract] OR "disk herniated"[Title/Abstract] OR "herniated disks"[Title/Abstract] OR "slipped disk"[Title/Abstract] OR ("Disk"[All Fields] AND "Slipped"[Title/Abstract]) OR "slipped disks"[Title/Abstract] OR "disk prolapse"[Title/Abstract] OR "disk prolapses"[Title/Abstract] OR (("Prolapse"[MeSH Terms] OR "Prolapse"[All Fields] OR "Prolapses"[All Fields] OR "Prolapsed"[All Fields] OR "prolapsing"[All Fields]) AND "Disk"[Title/Abstract]) OR (("Prolapse"[MeSH Terms] OR "Prolapse"[All Fields] OR "Prolapses"[All Fields] OR "Prolapsed"[All Fields] OR "prolapsing"[All Fields]) AND "Disk"[Title/Abstract]) OR "prolapsed disk"[Title/Abstract] OR "disk prolapsed"[Title/Abstract] OR "prolapsed disks"[Title/Abstract] OR "herniated disc"[Title/Abstract] OR "herniated discs"[Title/Abstract] OR "disc herniated"[Title/Abstract] OR "herniated disk"[Title/Abstract] OR "slipped disc"[Title/Abstract] OR "disc slipped"[Title/Abstract] OR "slipped discs"[Title/Abstract] OR "prolapsed disc"[Title/Abstract] OR "disc prolapsed"[Title/Abstract] OR "prolapsed discs"[Title/Abstract] OR "disc herniation"[Title/Abstract] OR "disc herniations"[Title/Abstract] OR "herniation disc"[Title/Abstract] OR "intervertebral disc herniation"[Title/Abstract] OR "herniation intervertebral disc"[Title/Abstract] OR "intervertebral disc herniations"[Title/Abstract] OR "intervertebral disk herniation"[Title/Abstract] OR "herniation intervertebral disk"[Title/Abstract] OR "intervertebral disk herniations"[Title/Abstract] OR "disk herniation"[Title/Abstract] OR "disk herniations"[Title/Abstract] OR "herniation disk"[Title/Abstract] OR "intervertebral disk protrusion"[Title/Abstract] OR ("Disk"[All Fields] AND "protrusion intervertebral"[Title/Abstract]) OR "intervertebral disk protrusions"[Title/Abstract] OR "protrusion intervertebral disk"[Title/Abstract] OR (("Protrusion"[All Fields] OR "Protrusions"[All Fields] OR "protrusive"[All Fields] OR "protrusiveness"[All Fields]) AND "intervertebral disk"[Title/Abstract]) OR "intervertebral disc protrusion"[Title/Abstract] OR ("Disc"[All Fields] AND "protrusion intervertebral"[Title/Abstract]) OR (("Disc"[All Fields] AND ("Protrusion"[All Fields] OR "Protrusions"[All Fields] OR "protrusive"[All Fields] OR "protrusiveness"[All Fields])) AND "Intervertebral"[Title/Abstract]) OR "intervertebral disc protrusions"[Title/Abstract] OR (("Protrusion"[All Fields] OR "Protrusions"[All Fields] OR "protrusive"[All Fields] OR "protrusiveness"[All Fields]) AND "intervertebral disc"[Title/Abstract]) OR "disc protrusion"[Title/Abstract] OR "disc protrusions"[Title/Abstract] OR "protrusion disc"[Title/Abstract] OR "disk protrusion"[Title/Abstract] OR "disk protrusions"[Title/Abstract] OR (("Protrusion"[All Fields] OR "Protrusions"[All Fields] OR "protrusive"[All Fields] OR "protrusiveness"[All Fields]) AND "Disk"[Title/Abstract])) AND ("Moxibustion"[MeSH Terms] OR "Moxibustion"[Title/Abstract] OR "moxa"[Title/Abstract] OR "moxa-stick"[Title/Abstract] OR "mugwort"[Title/Abstract] OR "Ai Jiu"[Title/Abstract] OR "Ai Jiu"[Title/Abstract] OR ("mild moxibustion"[Title/Abstract] OR "suspended moxibustion"[Title/Abstract] OR "suspension moxibustion"[Title/Abstract] OR "moxa-stick"[Title/Abstract] OR "moxa-stick"[Title/Abstract] OR "stick moxibustion"[Title/Abstract] OR "moxa stick moxibustion"[Title/Abstract] OR ("thunder-fire moxibustion"[Title/Abstract] OR "thunder-fire moxibustion"[Title/Abstract] OR "thunder-fire"[Title/Abstract] OR "leihuo"[Title/Abstract] OR "lei-huo"[Title/Abstract] OR "lei-huo"[Title/Abstract]) OR ("heat-sensitive moxibustion"[Title/Abstract] OR "heat-sensitive moxibustion"[Title/Abstract] OR "heat-sensitive acupoint"[Title/Abstract] OR "heat-sensitive acupoint"[Title/Abstract]) OR ("indirect moxibustion"[Title/Abstract] OR "partitioned moxibustion"[Title/Abstract] OR "insulated moxibustion"[Title/Abstract] OR "separated moxibustion"[Title/Abstract] OR "ginger-separated moxibustion"[Title/Abstract] OR "ginger partitioned moxibustion"[Title/Abstract] OR "ginger moxibustion"[Title/Abstract] OR "salt-separated moxibustion"[Title/Abstract] OR "salt partitioned moxibustion"[Title/Abstract] OR "salt moxibustion"[Title/Abstract]) OR ("warm-needling moxibustion"[Title/Abstract] OR "warm-needling moxibustion"[Title/Abstract] OR "warm needle moxibustion"[Title/Abstract] OR "warm-needling"[Title/Abstract] OR "warm-needling"[Title/Abstract] OR "warm needle"[Title/Abstract] OR "warming needle"[Title/Abstract] OR "wenzhen"[Title/Abstract] OR "wen zhen"[Title/Abstract] OR "wenzhenjiu"[Title/Abstract] OR (("Acupuncture Therapy"[MeSH Terms] OR "acupuncture"[Title/Abstract]) AND ("Moxibustion"[Title/Abstract] OR "moxa"[Title/Abstract]))))) 59  5 (((((((((((((((((((((((((((((((((((((((((((((((((((((((((((((((("Intervertebral Disc Displacement"[Mesh]) OR (Intervertebral Disc Displacement[Title/Abstract])) OR (Disc Displacement, Intervertebral[Title/Abstract])) OR (Intervertebral Disc Displacements[Title/Abstract])) OR (Protruded Disc[Title/Abstract])) OR (Disc, Protruded[Title/Abstract])) OR (Discs, Protruded[Title/Abstract])) OR (Protruded Discs[Title/Abstract])) OR (Protruded Disk[Title/Abstract])) OR (Disk, Protruded[Title/Abstract])) OR (Protruded Disks[Title/Abstract])) OR (Intervertebral Disk Displacement[Title/Abstract])) OR (Disk Displacement, Intervertebral[Title/Abstract])) OR (Intervertebral Disk Displacements[Title/Abstract])) OR (Disk, Herniated[Title/Abstract])) OR (Herniated Disks[Title/Abstract])) OR (Slipped Disk[Title/Abstract])) OR (Disk, Slipped[Title/Abstract])) OR (Slipped Disks[Title/Abstract])) OR (Disk Prolapse[Title/Abstract])) OR (Disk Prolapses[Title/Abstract])) OR (Prolapse, Disk[Title/Abstract])) OR (Prolapses, Disk[Title/Abstract])) OR (Prolapsed Disk[Title/Abstract])) OR (Disk, Prolapsed[Title/Abstract])) OR (Prolapsed Disks[Title/Abstract])) OR (Herniated Disc[Title/Abstract])) OR (Herniated Discs[Title/Abstract])) OR (Disc, Herniated[Title/Abstract])) OR (Herniated Disk[Title/Abstract])) OR (Slipped Disc[Title/Abstract])) OR (Disc, Slipped[Title/Abstract])) OR (Slipped Discs[Title/Abstract])) OR (Prolapsed Disc[Title/Abstract])) OR (Disc, Prolapsed[Title/Abstract])) OR (Prolapsed Discs[Title/Abstract])) OR (Disc Herniation[Title/Abstract])) OR (Disc Herniations[Title/Abstract])) OR (Herniation, Disc[Title/Abstract])) OR (Intervertebral Disc Herniation[Title/Abstract])) OR (Herniation, Intervertebral Disc[Title/Abstract])) OR (Intervertebral Disc Herniations[Title/Abstract])) OR (Intervertebral Disk Herniation[Title/Abstract])) OR (Herniation, Intervertebral Disk[Title/Abstract])) OR (Intervertebral Disk Herniations[Title/Abstract])) OR (Disk Herniation[Title/Abstract])) OR (Disk Herniations[Title/Abstract])) OR (Herniation, Disk[Title/Abstract])) OR (Intervertebral Disk Protrusion[Title/Abstract])) OR (Disk Protrusion, Intervertebral[Title/Abstract])) OR (Intervertebral Disk Protrusions[Title/Abstract])) OR (Protrusion, Intervertebral Disk[Title/Abstract])) OR (Protrusions, Intervertebral Disk[Title/Abstract])) OR (Intervertebral Disc Protrusion[Title/Abstract])) OR (Disc Protrusion, Intervertebral[Title/Abstract])) OR (Disc Protrusions, Intervertebral[Title/Abstract])) OR (Intervertebral Disc Protrusions[Title/Abstract])) OR (Protrusion, Intervertebral Disc[Title/Abstract])) OR (Disc Protrusion[Title/Abstract])) OR (Disc Protrusions[Title/Abstract])) OR (Protrusion, Disc[Title/Abstract])) OR (Disk Protrusion[Title/Abstract])) OR (Disk Protrusions[Title/Abstract])) OR (Protrusion, Disk[Title/Abstract])) AND (( "Moxibustion"[Mesh] OR moxibustion[tiab] OR moxa[tiab] OR "moxa stick"[tiab] OR mugwort[tiab] OR aijiu[tiab] OR "ai jiu"[tiab] OR "Ai Jiu"[tiab] ) OR ( ("mild moxibustion"[tiab] OR "gentle moxibustion"[tiab] OR "suspended moxibustion"[tiab] OR "suspension moxibustion"[tiab] OR "moxa stick"[tiab] OR "moxa-stick"[tiab] OR "stick moxibustion"[tiab] OR "moxa stick moxibustion"[tiab] OR xuanjiu[tiab] OR "xuan jiu"[tiab] OR wenhejiu[tiab] OR "wen he jiu"[tiab] ) OR ("thunder-fire moxibustion"[tiab] OR "thunder fire moxibustion"[tiab] OR "thunder-fire"[tiab] OR leihuo[tiab] OR "lei huo"[tiab] OR "lei-huo"[tiab] ) OR ("heat-sensitive moxibustion"[tiab] OR "heat sensitive moxibustion"[tiab] OR "heat-sensitized moxibustion"[tiab] OR "heat-sensitive acupoint"[tiab] OR "heat sensitive acupoint"[tiab] OR reminjiu[tiab] OR "re min jiu"[tiab] OR "remin jiu"[tiab] ) OR ("indirect moxibustion"[tiab] OR "partitioned moxibustion"[tiab] OR "insulated moxibustion"[tiab] OR "separated moxibustion"[tiab] OR "ginger-separated moxibustion"[tiab] OR "ginger partitioned moxibustion"[tiab] OR "ginger moxibustion"[tiab] OR "salt-separated moxibustion"[tiab] OR "salt partitioned moxibustion"[tiab] OR "salt moxibustion"[tiab] OR gejiangjiu[tiab] OR "ge jiang jiu"[tiab] OR geyanjiu[tiab] OR "ge yan jiu"[tiab] OR "geyan jiu"[tiab] ) OR ("warm-needling moxibustion"[tiab] OR "warm needling moxibustion"[tiab] OR "warm needle moxibustion"[tiab] OR "warm-needling"[tiab] OR "warm needling"[tiab] OR "warm needle"[tiab] OR "warming needle"[tiab] OR wenzhen[tiab] OR "wen zhen"[tiab] OR wenzhenjiu[tiab] OR "wen zhen jiu"[tiab] OR (("Acupuncture Therapy"[Mesh] OR acupuncture[tiab]) AND (moxibustion[tiab] OR moxa[tiab])) ) )) Randomized Controlled Trial (("Intervertebral Disc Displacement"[MeSH Terms] OR "Intervertebral Disc Displacement"[Title/Abstract] OR "disc displacement intervertebral"[Title/Abstract] OR "intervertebral disc displacements"[Title/Abstract] OR "protruded disc"[Title/Abstract] OR "disc protruded"[Title/Abstract] OR "discs protruded"[Title/Abstract] OR "protruded discs"[Title/Abstract] OR "protruded disk"[Title/Abstract] OR "disk protruded"[Title/Abstract] OR "protruded disks"[Title/Abstract] OR "intervertebral disk displacement"[Title/Abstract] OR ("Disk"[All Fields] AND "displacement intervertebral"[Title/Abstract]) OR "intervertebral disk displacements"[Title/Abstract] OR "disk herniated"[Title/Abstract] OR "herniated disks"[Title/Abstract] OR "slipped disk"[Title/Abstract] OR ("Disk"[All Fields] AND "Slipped"[Title/Abstract]) OR "slipped disks"[Title/Abstract] OR "disk prolapse"[Title/Abstract] OR "disk prolapses"[Title/Abstract] OR (("Prolapse"[MeSH Terms] OR "Prolapse"[All Fields] OR "Prolapses"[All Fields] OR "Prolapsed"[All Fields] OR "prolapsing"[All Fields]) AND "Disk"[Title/Abstract]) OR (("Prolapse"[MeSH Terms] OR "Prolapse"[All Fields] OR "Prolapses"[All Fields] OR "Prolapsed"[All Fields] OR "prolapsing"[All Fields]) AND "Disk"[Title/Abstract]) OR "prolapsed disk"[Title/Abstract] OR "disk prolapsed"[Title/Abstract] OR "prolapsed disks"[Title/Abstract] OR "herniated disc"[Title/Abstract] OR "herniated discs"[Title/Abstract] OR "disc herniated"[Title/Abstract] OR "herniated disk"[Title/Abstract] OR "slipped disc"[Title/Abstract] OR "disc slipped"[Title/Abstract] OR "slipped discs"[Title/Abstract] OR "prolapsed disc"[Title/Abstract] OR "disc prolapsed"[Title/Abstract] OR "prolapsed discs"[Title/Abstract] OR "disc herniation"[Title/Abstract] OR "disc herniations"[Title/Abstract] OR "herniation disc"[Title/Abstract] OR "intervertebral disc herniation"[Title/Abstract] OR "herniation intervertebral disc"[Title/Abstract] OR "intervertebral disc herniations"[Title/Abstract] OR "intervertebral disk herniation"[Title/Abstract] OR "herniation intervertebral disk"[Title/Abstract] OR "intervertebral disk herniations"[Title/Abstract] OR "disk herniation"[Title/Abstract] OR "disk herniations"[Title/Abstract] OR "herniation disk"[Title/Abstract] OR "intervertebral disk protrusion"[Title/Abstract] OR ("Disk"[All Fields] AND "protrusion intervertebral"[Title/Abstract]) OR "intervertebral disk protrusions"[Title/Abstract] OR "protrusion intervertebral disk"[Title/Abstract] OR (("Protrusion"[All Fields] OR "Protrusions"[All Fields] OR "protrusive"[All Fields] OR "protrusiveness"[All Fields]) AND "intervertebral disk"[Title/Abstract]) OR "intervertebral disc protrusion"[Title/Abstract] OR ("Disc"[All Fields] AND "protrusion intervertebral"[Title/Abstract]) OR (("Disc"[All Fields] AND ("Protrusion"[All Fields] OR "Protrusions"[All Fields] OR "protrusive"[All Fields] OR "protrusiveness"[All Fields])) AND "Intervertebral"[Title/Abstract]) OR "intervertebral disc protrusions"[Title/Abstract] OR (("Protrusion"[All Fields] OR "Protrusions"[All Fields] OR "protrusive"[All Fields] OR "protrusiveness"[All Fields]) AND "intervertebral disc"[Title/Abstract]) OR "disc protrusion"[Title/Abstract] OR "disc protrusions"[Title/Abstract] OR "protrusion disc"[Title/Abstract] OR "disk protrusion"[Title/Abstract] OR "disk protrusions"[Title/Abstract] OR (("Protrusion"[All Fields] OR "Protrusions"[All Fields] OR "protrusive"[All Fields] OR "protrusiveness"[All Fields]) AND "Disk"[Title/Abstract])) AND ("Moxibustion"[MeSH Terms] OR "Moxibustion"[Title/Abstract] OR "moxa"[Title/Abstract] OR "moxa-stick"[Title/Abstract] OR "mugwort"[Title/Abstract] OR "Ai Jiu"[Title/Abstract] OR "Ai Jiu"[Title/Abstract] OR ("mild moxibustion"[Title/Abstract] OR "suspended moxibustion"[Title/Abstract] OR "suspension moxibustion"[Title/Abstract] OR "moxa-stick"[Title/Abstract] OR "moxa-stick"[Title/Abstract] OR "stick moxibustion"[Title/Abstract] OR "moxa stick moxibustion"[Title/Abstract] OR ("thunder-fire moxibustion"[Title/Abstract] OR "thunder-fire moxibustion"[Title/Abstract] OR "thunder-fire"[Title/Abstract] OR "leihuo"[Title/Abstract] OR "lei-huo"[Title/Abstract] OR "lei-huo"[Title/Abstract]) OR ("heat-sensitive moxibustion"[Title/Abstract] OR "heat-sensitive moxibustion"[Title/Abstract] OR "heat-sensitive acupoint"[Title/Abstract] OR "heat-sensitive acupoint"[Title/Abstract]) OR ("indirect moxibustion"[Title/Abstract] OR "partitioned moxibustion"[Title/Abstract] OR "insulated moxibustion"[Title/Abstract] OR "separated moxibustion"[Title/Abstract] OR "ginger-separated moxibustion"[Title/Abstract] OR "ginger partitioned moxibustion"[Title/Abstract] OR "ginger moxibustion"[Title/Abstract] OR "salt-separated moxibustion"[Title/Abstract] OR "salt partitioned moxibustion"[Title/Abstract] OR "salt moxibustion"[Title/Abstract]) OR ("warm-needling moxibustion"[Title/Abstract] OR "warm-needling moxibustion"[Title/Abstract] OR "warm needle moxibustion"[Title/Abstract] OR "warm-needling"[Title/Abstract] OR "warm-needling"[Title/Abstract] OR "warm needle"[Title/Abstract] OR "warming needle"[Title/Abstract] OR "wenzhen"[Title/Abstract] OR "wen zhen"[Title/Abstract] OR "wenzhenjiu"[Title/Abstract] OR (("Acupuncture Therapy"[MeSH Terms] OR "acupuncture"[Title/Abstract]) AND ("Moxibustion"[Title/Abstract] OR "moxa"[Title/Abstract])))))) AND (randomizedcontrolledtrial[Filter]) 12 |
| **Web of Science** | 1 "TS=(""Intervertebral Disc Displacement"" OR ""Protruded Discs"" OR ""Disc Protrusion"" OR ""Intervertebral"" OR ""Intervertebral Disk Herniations"" OR ""Protrusion, Disk"" OR ""Intervertebral Disk Displacements"" OR ""Disk, Prolapsed"" OR ""Disk Prolapse"" OR ""Disc Herniation"" OR ""Protrusions, Intervertebral Disk"" OR ""Disc Protrusions, Intervertebral"" OR ""Intervertebral Disk Protrusions"" OR ""Herniation, Disk"" OR ""Protrusion, Intervertebral Disk"" OR ""Intervertebral Disc Protrusions"" OR ""Herniation, Intervertebral Disk"" OR ""Disc, Protruded"" OR ""Herniated Disks"" OR ""Discs, Protruded"" OR ""Intervertebral Disk Protrusion"" OR ""Herniated Discs"" OR ""Intervertebral Disk Herniation"" OR ""Intervertebral Disk Displacement"" OR ""Prolapsed Disks"" OR ""Disk Protrusion, Intervertebral"" OR ""Disk Herniation"" OR ""Prolapsed Discs"" OR ""Slipped Discs"" OR ""Intervertebral Disc Herniation"" OR ""Protrusion, Disc"" OR ""Disc, Prolapsed"" OR ""Disk Displacement, Intervertebral"" OR ""Herniation, Disc"" OR ""Prolapses, Disk"" OR ""Slipped Disk"" OR ""Slipped Disc"" OR ""Disc Protrusions"" OR ""Intervertebral Disc Protrusion"" OR ""Slipped Disks"" OR ""Disk Protrusion"" OR ""Prolapsed Disk"" OR ""Prolapse, Disk"" OR ""Disk, Protruded"" OR ""Protruded Disks"" OR ""Protrusion, Intervertebral Disc"" OR ""Disk Protrusions"" OR ""Protruded Disc"" OR ""Disk Prolapses"" OR ""Herniation, Intervertebral Disc"" OR ""Herniated Disk"" OR ""Intervertebral Disc Herniations"" OR ""Intervertebral Disc Displacements"" OR ""Disk Herniations"" OR ""Disc, Slipped"" OR ""Disc Protrusion"" OR ""Disc Herniations"" OR ""Herniated Disc"" OR ""Disk, Slipped"" OR ""Prolapsed Disc"" OR ""Disc Displacement, Intervertebral"" OR "" Disk, Herniated"" OR ""Disc, Herniated"" OR ""Protruded Disk"") " Web of Science Core Collection 40129  2 "TS=(""Moxibustion"" OR ""moxa"" OR ""moxa stick"" OR ""mugwort"" OR ""ai jiu"" OR ""aijiu"" OR ""Ai Jiu"" OR ""mild moxibustion"" OR ""gentle moxibustion"" OR ""suspended moxibustion"" OR ""suspension moxibustion"" OR ""moxa stick"" OR ""moxa-stick"" OR ""stick moxibustion"" OR ""moxa stick moxibustion"" OR ""xuanjiu"" OR ""xuan jiu"" OR "" wenhejiu"" OR ""wen he jiu"" OR ""thunder-fire moxibustion"" OR ""thunder fire moxibustion"" OR ""thunder-fire"" OR ""leihuo"" OR ""lei huo"" OR ""lei-huo"" OR ""heat-sensitive moxibustion"" OR ""heat sensitive moxibustion"" OR ""heat-sensitized moxibustion"" OR ""heat-sensitive acupoint"" OR ""heat sensitive acupoint"" OR ""reminjiu"" OR ""re min jiu"" OR ""remin jiu"" OR ""indirect moxibustion"" OR ""partitioned moxibustion"" OR ""insulated moxibustion"" OR ""separated moxibustion"" OR ""ginger-separated moxibustion"" OR ""ginger partitioned moxibustion"" OR ""ginger moxibustion"" OR ""salt-separated moxibustion"" OR ""salt partitioned moxibustion"" OR ""salt moxibustion"" OR ""gejiangjiu"" OR ""ge jiang jiu"" OR ""geyanjiu"" OR ""ge yan jiu"" OR ""geyan jiu"" OR ""warm-needling moxibustion"" OR ""warm needling moxibustion"" OR ""warm needle moxibustion"" OR ""warm-needling"" OR ""warm needling"" OR ""warm needle"" OR ""warming needle"" OR ""wenzhen"" OR ""wen zhen"" OR ""wenzhenjiu"" OR ""wen zhen jiu"") " Web of Science Core Collection 4567  3 "TS=(  random* OR randomi* OR randomly  OR placebo* OR sham  OR trial OR ""clinical trial"" OR ""controlled clinical trial""  OR ""double blind"" OR ""single blind"" OR ""triple blind""  OR crossover OR ""cross over"" OR ""parallel group""  OR cluster random*  OR RCT  ) " Web of Science Core Collection 4487818  4 "#3 AND #2 AND #1 " Web of Science Core Collection 37 |
| **Cochrane** | #1 MeSH descriptor: [Intervertebral Disc Displacement] explode all trees 1434  #2 "Protruded Discs" OR "Disc Protrusion" OR "Intervertebral" OR "Intervertebral Disk Herniations" OR "Protrusion, Disk" OR "Intervertebral Disk Displacements" OR "Disk, Prolapsed" OR "Disk Prolapse" OR "Disc Herniation" OR "Protrusions, Intervertebral Disk" OR "Disc Protrusions, Intervertebral" OR "Intervertebral Disk Protrusions" OR "Herniation, Disk" OR "Protrusion, Intervertebral Disk" OR "Intervertebral Disc Protrusions" OR "Herniation, Intervertebral Disk" OR "Disc, Protruded" OR "Herniated Disks" OR "Discs, Protruded" OR "Intervertebral Disk Protrusion" OR "Herniated Discs" OR "Intervertebral Disk Herniation" OR "Intervertebral Disk Displacement" OR "Prolapsed Disks" OR "Disk Protrusion, Intervertebral" OR "Disk Herniation" OR "Prolapsed Discs" OR "Slipped Discs" OR "Intervertebral Disc Herniation" OR "Protrusion, Disc" OR "Disc, Prolapsed" OR "Disk Displacement, Intervertebral" OR "Herniation, Disc" OR "Prolapses, Disk" OR "Slipped Disk" OR "Slipped Disc" OR "Disc Protrusions" OR "Intervertebral Disc Protrusion" OR "Slipped Disks" OR "Disk Protrusion" OR "Prolapsed Disk" OR "Prolapse, Disk" OR "Disk, Protruded" OR "Protruded Disks" OR "Protrusion, Intervertebral Disc" OR "Disk Protrusions" OR "Protruded Disc" OR "Disk Prolapses" OR "Herniation, Intervertebral Disc" OR "Herniated Disk" OR "Intervertebral Disc Herniations" OR "Intervertebral Disc Displacements" OR "Disk Herniations" OR "Disc, Slipped" OR "Disc Protrusion" OR "Disc Herniations" OR "Herniated Disc" OR "Disk, Slipped" OR "Prolapsed Disc" OR "Disc Displacement, Intervertebral" OR " Disk, Herniated" OR "Disc, Herniated" OR "Protruded Disk" 6152  #3 #1 OR #2 6152  #4 "Moxibustion" OR "moxa" OR "moxa stick" OR "mugwort" OR "ai jiu" OR "aijiu" OR "Ai Jiu" OR "mild moxibustion" OR "gentle moxibustion" OR "suspended moxibustion" OR "suspension moxibustion" OR "moxa stick" OR "moxa-stick" OR "stick moxibustion" OR "moxa stick moxibustion" OR "xuanjiu" OR "xuan jiu" OR " wenhejiu" OR "wen he jiu" OR "thunder-fire moxibustion" OR "thunder fire moxibustion" OR "thunder-fire" OR "leihuo" OR "lei huo" OR "lei-huo" OR "heat-sensitive moxibustion" OR "heat sensitive moxibustion" OR "heat-sensitized moxibustion" OR "heat-sensitive acupoint" OR "heat sensitive acupoint" OR "reminjiu" OR "re min jiu" OR "remin jiu" OR "indirect moxibustion" OR "partitioned moxibustion" OR "insulated moxibustion" OR "separated moxibustion" OR "ginger-separated moxibustion" OR "ginger partitioned moxibustion" OR "ginger moxibustion" OR "salt-separated moxibustion" OR "salt partitioned moxibustion" OR "salt moxibustion" OR "gejiangjiu" OR "ge jiang jiu" OR "geyanjiu" OR "ge yan jiu" OR "geyan jiu" OR "warm-needling moxibustion" OR "warm needling moxibustion" OR "warm needle moxibustion" OR "warm-needling" OR "warm needling" OR "warm needle" OR "warming needle" OR "wenzhen" OR "wen zhen" OR "wenzhenjiu" OR "wen zhen jiu" 7316  #5 "Acupuncture Therapy" AND "moxibustion" 1758  #6 "Acupuncture Therapy" AND "moxa" 58  #7 "acupuncture" AND "moxibustion" 6169  #8 "acupuncture" AND "moxa" 197  #9 #4 OR #5 OR #6 OR #7 OR #8 7316  #10 #3 AND #9 188 |
| **Embase** | #1 'intervertebral disk hernia'/exp OR 'disc hernia' OR 'disc herniation' OR 'disc prolapse' OR 'discal hernia' OR 'discal herniation' OR 'discus hernia' OR 'disk hernia' OR 'disk hernia disease' OR 'disk herniation' OR 'disk prolapse' OR 'disk protrusion' OR 'disk, herniated' OR 'hernia disci' OR 'hernia nuclei pulposi' OR 'herniated disc' OR 'herniated disk' OR 'herniated intervertebral disc' OR 'herniated intervertebral disk' OR 'herniated nucleus pulposus' OR 'herniated vertebral disc' OR 'herniated vertebral disk' OR 'intervertebral disc displacement' OR 'intervertebral disc hernia' OR 'intervertebral disc prolapse' OR 'intervertebral disc protrusion' OR 'intervertebral disk displacement' OR 'intervertebral disk herniation' OR 'intervertebral disk perforation' OR 'intervertebral disk prolapse' OR 'intervertebral disk protrusion' OR 'intervertebral disk rupture' OR 'intervertebral prolapse' OR 'invertebral disk hernia' OR 'nucleus pulposus hernia' OR 'rupture, intervertebral disk' OR 'slipped disc' OR 'slipped disk' OR 'slipped intervertebral disc' OR 'slipped vertebral disc' OR 'spinal disc prolapse' OR 'spinal disk disease' OR 'spinal disk hernia' OR 'vertebral disc hernia' OR 'vertebral disk hernia' OR 'intervertebral disk hernia' 38296  #2 'moxibustion'/exp OR 'moxibustion' OR 'moxa' OR 'mugwort' OR 'aijiu' OR 'ai jiu' OR 'mild moxibustion' OR 'gentle moxibustion' OR 'suspended moxibustion' OR 'suspension moxibustion' OR 'moxa stick' OR 'moxa-stick' OR 'stick moxibustion' OR 'moxa stick moxibustion' OR 'xuanjiu' OR 'xuan jiu' OR 'wenhejiu' OR 'wen he jiu' OR 'thunder-fire moxibustion' OR 'thunder fire moxibustion' OR 'thunder-fire' OR 'leihuo' OR 'lei huo' OR 'lei-huo' OR 'heat-sensitive moxibustion' OR 'heat sensitive moxibustion' OR 'reminjiu' OR 're min jiu' OR 'remin jiu' OR 'indirect moxibustion' OR 'partitioned moxibustion' OR 'insulated moxibustion' OR 'separated moxibustion' OR 'ginger-separated moxibustion' OR 'ginger partitioned moxibustion' OR 'ginger moxibustion' OR 'salt-separated moxibustion' OR 'salt partitioned moxibustion' OR 'salt moxibustion' OR 'gejiangjiu' OR 'ge jiang jiu' OR 'geyanjiu' OR 'ge yan jiu' OR 'geyan jiu' OR 'warm-needling moxibustion' OR 'warm needling moxibustion' OR 'warm needle moxibustion' OR 'warm-needling' OR 'warm needling' OR 'warm needle' OR 'warming needle' OR 'wenzhen' OR 'wen zhen' OR 'wenzhenjiu' OR 'wen zhen jiu' 23942  #3 #1 AND #2 268  #4 #3 AND 'randomized controlled trial'/de 89 |
| **China National Knowledge Infrastructure** | (SU = Lumbar Disc Herniation OR SU = Lumbar Intervertebral Disc Herniation OR SU = Intervertebral Disc Displacement OR SU = Lumbar Disc Protrusion OR SU = Lumbar Disc Bulge)  AND  (SU = Moxibustion OR SU = Mild Moxibustion OR SU = Suspended Moxibustion OR SU = Thunder-Fire Moxibustion  OR SU = Heat-Sensitive Moxibustion OR SU = Indirect Moxibustion OR SU = Ginger-Partitioned Moxibustion OR SU = Salt-Partitioned Moxibustion OR SU = Warm Needling Moxibustion)  AND  (SU = Randomized OR SU = Clinical Observation OR SU = Randomized Controlled Trial) 237 |
| **Wanfang Medical Database** | (Subject: (Lumbar Disc Herniation) OR Title/Keywords: (Lumbar Disc Herniation OR Lumbar Intervertebral Disc Herniation OR Intervertebral Disc Displacement OR Lumbar Disc Protrusion OR Lumbar Disc Bulge))  AND  (Subject: (Moxibustion) OR Title/Keywords: (Mild Moxibustion OR Suspended Moxibustion OR Thunder-Fire Moxibustion OR Heat-Sensitive Moxibustion OR Indirect Moxibustion OR Ginger-Partitioned Moxibustion OR Salt-Partitioned Moxibustion OR Warm Needling Moxibustion OR Moxibustion))  AND  (Subject: (Randomized Controlled Trial OR Randomized OR Clinical Observation)) 1173 |

| Table S3 References |
| --- |

1. Wang L, Xiong Y, Li J. Effect of Moxibustion,Cupping Combined with Auricular Acupuncture on Lumbar Mobility in Patients with Liver and Kidney Deficiency Type Lumbar Disc Herniation. *Journal of Clinical Nursing in Practice* (2024) 10(03):53–6. doi: 10.11997/nitcwm.202403014.

2. Zhang Q, Chen S, Ma B. Application Effect of Thunder Fire Moxibustion Combined with Traditional Chinese Medicine Fumigation on Patients with Lumbar Disc Herniation of Qi Stagnation and Blood Stasis Type. *Journal of Clinical Nursing in Practice* (2023) 9(11):49–52. doi: 10.11997/nitcwm.202311013.

3. Cai C, Gong Y, Dong D, Xue J, Zheng X, Zhong Z, et al. Combined Therapies of Modified Taiyi Miraculous Moxa Roll and Cupping for Patients with Lumbar Intervertebral Disc Herniation. *Evid Based Complement Alternat Med* (2018) 2018:6754730. Epub 20180328. doi: 10.1155/2018/6754730.

4. Xu M, Yang X, Liu S, Gu C. Observation of Thunder Fire Moxibustion Pendulum Method on the Acute Exacerbation of Lumbar Disc Herniation. *Journal of Emergency in Traditional Chinese Medicine* (2016) 25(03):482–4. doi: 10.3969/j.issn.1004-745X.2016.03.034.

5. Hu Q, Liu P, Li H. Effects of Cupping Therapy Combined with Moxibustion on Pain Severity and Lumbar Function in Patients with Lumbar Disc Herniation. *Chinese Journal of Medical Device* (2024) 37(05):137–9+43. doi: 10.3969/j.issn.1002-2376.2024.05.040.

6. Ding W, Xie D, Chen R, Jiao L, Li Q. A Randomized Controlled Trial on the Enhancing Effect of Deqi in Patients with Lumbar Disc Herniation through Heat-Sensitive Moxibustion of Governor Vessel Combined with the Manipulation Release of Meridians. *Journal of Jiangxi University of Traditional Chinese Medicine* (2025) 37(04):55–60. doi: 10.20140/j.2095-7785.2025.04.14.

7. Xu F. Heat-Sensitive Moxibustion Based on Syndrome Differentiation of Six Channels in the Treatment of Lumbar Disc Herniation. *Chinese Medicine Modern Distance Education of China* (2025) 23(11):121–3. doi: 10.3969/j.issn.1672-2779.2025.11.039.

8. Zhong Q, Du L, Li S, Wang Z. Clinical Effect of Thunder Fire Moxibustion Combined with Modified Shen-Tong Zhuyu Decoction on Lumbar Disc Herniation. *China Modern Medicine* (2024) 31(35):105–8. doi: 10.3969/j.issn.1674-4721.2024.35.024.

9. Liu W, Tan X, Zhong Z, Ye Q, Li J. Clinical Observation of Strong Partitioned Moxibustion Combined with Acupuncture in the Treatment of Lumbar Disc Herniation of the Spleen Deficiency and Dampness Excess Type. *Journal of Practical Traditional Chinese Medicine* (2024) 40(12):2529–31.

10. Wu Y, Ai C, Liu J, Zeng H, Wu C, Zhan H. Clinical Observation on Modified Shentong Zhuyu Decoction Combined with Heat-Sensitive Moxibustion in Treating Lumbar Disc Herniation of Qi Stagnation and Blood Stasis Type. *Drug Evaluation* (2025) 22(07):871–4. doi: 10.19939/j.cnki.1672-2809.2025.07.22.

11. Xu S, Li Y, Li Y, Yang W, Wu C. Observation on the Effect of Warm Acupuncture Combined with Cupping Therapy on Lumbar Intervertebraldisc Herniation. *Contemporary Medicine* (2019) 25(34):98–100. doi: 10.3969/j.issn.1009-4393.2019.34.040.

12. Zhou H, Deng X. The Influence of Thunder-Fire Moxibustion Combined with Lumbar Traction and Massage on Pain and Lower Limb Function in Patients with Lumbar Disc Herniation. *Henan Traditional Chinese Medicine* (2024) 44(04):600–3. doi: 10.16367/j.issn.1003-5028.2024.04.0113.

13. Yao D, Liu C, Wang Q, Li T, Yao C, Zhang Q, et al. Clinical Study of Limin Spinal Manipulation Combined with Heat-Sensitive Moxibustion in Treatment of Ldh. *Journal of Clinical Acupuncture and Moxibustion* (2024) 40(08):46–51. doi: 10.19917/j.cnki.1005-0779.024154.

14. Xiong Q, Cheng C, Liu F. Effects of Heat Sensitive Moxibustion Combined with Massage with Point Selection Along Channel on Lumbago Degree and Lumbar Function in Patients with Lumbar Disc Herniation. *Medical Innovation of China* (2024) 21(34):71–4. doi: 10.3969/j.issn.1674-4985.2024.34.017.

15. Chen W, Xu T, Xiao X, Zhong J, Wu L, Liang J, et al. Clinical Observation of Heat-Sensitive Moxibustion and Acupuncture in Treating Patients with Low Back Pain. *Journal of Liaoning University of Traditional Chinese Medicine* (2021) 23(02):181–4. doi: 10.13194/j.issn.1673-842x.2021.02.040.

16. Liang Y. Clinical Observation of 30 Cases of Lumbar Disc Herniation Treated with Moxibustion and Medicinal Ironing Therapy. *Chinese Journal of Ethnomedicine and Ethnopharmacy* (2014) 23(19):66–7.

17. Deng M, Qiu W, Guan Y. Clinical Observation of Lumbar Disc Herniation Treated with Moxibustion Combined with Traditional Chinese Medicinal Hot Compress Therapy. *Guangming Journal of Chinese Medicine* (2022) 37(11):1906–9. doi: 10.3969/j.issn.1003-8914.2022.11.006.

18. Li S. Clinical Observation of Lumbar Disc Herniation Treated with Abdominal Acupuncture Combined with Ginger-Separated Moxibustion. *Shenzhen Journal of Integrated Traditional Chinese and Western Medicine* (2015) 25(01):66–8. doi: 10.16458/j.cnki.1007-0893.2015.01.036.

19. Liu Y. Clinical Observation of Cold-Congealing and Blood-Stasis Type Lumbar Disc Herniation Treated with Thunder-Fire Moxibustion Combined with Electroacupuncture with Multiple-Needle Technique. *China's Naturopathy* (2022) 30(22):33–6. doi: 10.19621/j.cnki.11-3555/r.2022.2211.

20. Guo H. Clinical Observation of Lumbar Disc Herniation Treated with Heat-Sensitive Moxibustion Combined with Traditional Chinese Medicinal Damp-Heat Compress Therapy. *Journal of Practical Traditional Chinese Medicine* (2023) 39(02):393–4.

21. Lin M, Fang Y, Zeng S. Clinical Observation of Cold-Dampness Obstruction Type Lumbar Disc Herniation Treated with Warm-Needle Moxibustion. *China's Naturopathy* (2021) 29(15):32–5. doi: 10.19621/j.cnki.11-3555/r.2021.1512.

22. Wen Q. Clinical Observation of Cold-Dampness Type Lumbar Disc Herniation Treated with Warm-Needle Moxibustion. *China's Naturopathy* (2023) 31(11):68–71. doi: 10.19621/j.cnki.11-3555/r.2023.1121.

23. Xie W, Shi J, Zhang X. Clinical Observation of Lumbar Disc Herniation Treated with Acupuncture Combined with Heat-Sensitive Moxibustion. *Journal of Practical Traditional Chinese Medicine* (2023) 39(11):2245–7.

24. Zhang J, Jiao J, Zhang S. Warm-Needle Moxibustion Combined with Duhuo Jisheng Decoction for Lumbar Disc Herniation with Qi Stagnation and Blood Stasis Complicated by Liver and Kidney Deficiency Syndrome: A Randomized Controlled Trial. *Chinese Acupuncture & Moxibustion*:1–15. doi: 10.13703/j.0255-2930.20250228-0001.

25. Li J, Xiao L, Zeng J, Yang Y, Huang R, Yue L. Effects of Warm Acupuncture Combined with Chinese Herbal Medicine Soaking on Pain,Lumbar Function and Quality of Life in Patients with Lumbar Disc Herniation. *Liaoning Journal of Traditional Chinese Medicine* (2024) 51(11):164–7. doi: 10.13192/j.issn.1000-1719.2024.11.042.

26. Zhu B, Qi R, Shen Y, Xing Y, Ma Y. Clinical Observation of Warm-Needle Moxibustion for Lumbar Disc Herniation of Qi Stagnation and Blood Stasis Type and Cold-Dampness Obstruction Type. *China's Naturopathy* (2021) 29(01):46–9. doi: 10.19621/j.cnki.11-3555/r.2021.0119.

27. Yu A, Ju B, Wang Q, Liang J, Jiang S, Xu T, et al. Clinical Observation of 60 Cases of Pain Due to Lumbar Disc Herniation Treated with Moxibustion at Lumbar Heat-Sensitive Acupoints. *Shanghai Journal of Traditional Chinese Medicine* (2017) 51(S1):102–4+14. doi: 10.16305/j.1007-1334.2017.S1.029.

28. Yin S. Clinical Observation of 44 Cases of Lumbar Disc Herniation Treated with Traditional Chinese Medicinal Fumigation Combined with Warm-Needle Moxibustion. *Hunan Journal of Traditional Chinese Medicine* (2022) 38(08):68–70. doi: 10.16808/j.cnki.issn1003-7705.2022.08.016.

29. Xu Z, Fang H, Shi Y, Ling Y. Clinical Study on Moxibustion Combined with Warming and Activating Meridian Massage in Treatment of Blood Stasis Vessel Obstruction Type of Prolapse of Lumbar Intervertebral Disc. *World Chinese Medicine* (2018) 13(1):170–4. doi: 10.3969/j.issn.1673-7202.2018.01.042.

30. Jiang Y. Study on the Nursing Effects of Moxibustion Combined with Traditional Chinese Medicinal Fumigation in Patients with Qi Stagnation and Blood Stasis Type Lumbar Disc Herniation. *Xinjiang Journal of Traditional Chinese Medicine* (2023) 41(5):75–7.

31. Feng M, Liu H. Clinical Effect Observation of Electroacupuncture Combined with Thermal Moxibustion in Treatment of Lumbar Disc Herniation. *Smart Healthcare* (2021) 7(30):54–6. doi: 10.19335/j.cnki.2096-1219.2021.30.019.

32. Wang G, Zhang X, Li C, Huang W. Observations on the Efficacy of Electroacupuncture Plus Indirect Moxibustion Treating Lumbar Intervertebral Disc Herniation. *Shanghai Journal of Acupuncture and Moxibustion* (2017) 36(12):1466–8. doi: 10.13460/j.issn.1005-0957.2017.12.1466.

33. Wang J. Auricular Acupuncture Combined with Indirect Moxibustion on Improving Lumbar Function in Patients with Lumbar Disc Herniation. *Chinese Medicine Modern Distance Education of China* (2025) 23(12):99–102. doi: 10.3969/j.issn.1672-2779.2025.12.032.

34. Sun J, Jia Z, Dong J, Xie C, Xu Z, Zhu X. Clinical Study on Treating Lumbar Interveterbral Disc Protrusion by Abdomen Needle Combined with Moxibustion. *Journal of Clinical Acupuncture and Moxibustion* (2009) 25(8):1–2. doi: 10.3969/j.issn.1005-0779.2009.08.001.

35. Sun J. Observation of the Therapeutic Effect of Ginger Separated Moxibustion Combined with Self-Made Traditional Chinese Medicine Formula on Lumbar Disc Herniation in 60 Cases. *Capital Medicine* (2025) 32(3):135–8. doi: 10.3969/j.issn.1005-8257.2025.03.046.

36. Xu C, Yu N, Lu J, Xiong S, Liu Y. Observations on Efficacy of Spreading Medicinal Moxibustion on Ginger Plus Massotherapy for Lumbar Intervertebral Disc Herniation. *Shanghai Journal of Acupuncture and Moxibustion* (2018) 37(9):1059–62. doi: 10.13460/j.issn.1005-0957.2018.09.1059.

37. Gao Y, Meng H. Effect of Sandwiched Moxibustion Combined with Traditional Chinese Medicine Hot Package on Functional Rehabilitation of Lumbar Spine in Patients with Lumbar Disc Herniation. *Acta Medicinae Sinica* (2021) 34(2):119–23. doi: 10.19296/j.cnki.1008-2409.2021-02-033.

38. Jiang Y, Wang H, Mou J. Effects of Mixed-Frequency Electroacupuncture Therapy Combined with Ginger Interposed Moxibustion on Patients with Lumbar Disc Herniation Complicated by Sciatica. *Clinical Misdiagnosis & Mistherapy* (2025) 38(18):103–7. doi: 10.3969/j.issn.1002-3429.2025.18.021.

39. Liu C, Lu M, Kang Z, Zhang T, Wang Z, Zhang Y, et al. Effects of Thunder-Fire Moxibustion at Huangshu (Bl52) on Pain Severity and Plasma Substance P Levels in Patients with Lumbar Disc Herniation. *Modern Medicine and Health Research* (2021) 5(09):138–40.

40. Yin Z. The Nursing Effect of Thunder Fire Moxibustion Combined with Traditional Chinese Medicine Fumigation on Patients with Blood Stasis and Qi Stagnation Type Lumbar Intervertebral Disc Herniation. *Capital Medicine* (2023) 30(20):139–41. doi: 10.3969/j.issn.1005-8257.2023.20.048.

41. Zhang A, Guo R, Liu Y. Application Effect of Heat Sensitive Moxibustion Combined with Acupoint Massage in the Nursing of Patients with Low Back and Leg Pain. *Henan Medical Research* (2023) 32(9):1697–701. doi: 10.3969/j.issn.1004-437X.2023.09.039.

42. Hua Y, Mo M, Dong L, Liu Q. Observation on the Therapeutic Effects of Heat-Sensitive Moxibustion Combined with Traditional Chinese Medicinal Fumigation and Washing in the Treatment of Lumbar Disc Herniation. *Journal of Guangxi University of Chinese Medicine* (2015) 18(3):28–30.

43. Wu Z. Observation on the Clinical Effects of Tuina Combined with Warm-Needle Moxibustion at Jiaji (Ex-B2) Points in the Treatment of Lumbar Disc Herniation. *Journal of Guangxi University of Chinese Medicine* (2017) 15(4):114–6.

44. Chen J. Efficacy of Tuina Manipulation Combined with Moxibustion on Patients with Lumbar Disc Herniation. *Journal of Clinical Acupuncture and Moxibustion* (2016) 32(1):21–3.

45. Wang H. Clinical Observation of Warm-Needle Moxibustion Combined with Tuina in the Treatment of Lumbar Disc Herniation. *Journal of Practical Traditional Chinese Medicine* (2020) 36(03):371–2.

46. Zhao H. Effects of Warm-Needle Moxibustion Combined with Acupoint Massage on Pain and Functional Recovery in Patients with Lumbar Disc Herniation. *Modern Diagnosis & Treatment* (2023) 34(12):1894–6.

47. Li X, Li L, Yuan M. Clinical Effect of Warm Acupuncture and Moxibustion for Treatment of Lumbar Disc Herniation. *Chinese and Foreign Medical Research* (2019) 17(25):152–4. doi: 10.14033/j.cnki.cfmr.2019.25.065.

48. Xu M. Clinical Observation of Warm-Needle Moxibustion Added at Lumbar Jiaji (Ex-B2) Points for the Treatment of Lumbar Disc Herniation and Its Effects on Pain. *Medical Diet and Health* (2019) (14):138.

49. Pan M, Sun L, Yao H. Acupuncture Combined with Ginger-Separated Moxibustion in Treatment of Lumbar Disc Herniation with Sciatica. *Liaoning Journal of Traditional Chinese Medicine* (2025) 52(3):166–9,后插4. doi: 10.13192/j.issn.1000-1719.2025.03.043.

50. Li J. Observation on the Clinical Effects of Traditional Chinese Medical Tuina Therapy Combined with Warm-Needle Moxibustion in the Treatment of Lumbar Disc Herniation. *Contemporary Medicine Forum* (2024) 22(18):99–101. doi: 10.3969/j.issn.2095-7629.2024.18.029.

|  |
| --- |

Table S4: Detailed characteristics of the included studies

| **No.** | **Disease** | **First author and year of publication** | **experimental group** | **Control Group** | **VAS score** | **JOA score** | **ODIscore** | **Country** |
| --- | --- | --- | --- | --- | --- | --- | --- | --- |
| 1 | LDH | Chunyue Cai 2018 | TCM+MOXI | ACU | 6.540±1.289/6.349 ± 1.143 | 13.83±1.54/13.71 ± 1.47 | NR | China |
| 2 | LDH | Xu Minmin 2016 | MOXI | CT | 6.96±1.52/6.83±1.58 | 10.44±1.55/10.51±1.41 | NR | China |
| 3 | LDH | Zhang Qun 2023 | TCM+MOXI | CT | NR | 10.13 ± 1.68/10.11 ± 1.71 | NR | China |
| 4 | LDH | Wang Liya 2024 | CT+MOXI | CT | 5.11±0.69/5.18±0.75 | NR | 42.26±4.37/42.19±4.59 | China |
| 5 | LDH | Hu Qinqin 2024 | TCM+MOXI | CT | 7.12±1.02/7.16±1.05 | 10.97±1.56/10.74±1.64 | 36.25±5.26/36.33±5.37 | China |
| 6 | LDH | Ding Wenjie 2025 | TUINA+MOXI | MOXI | 6.49±0.72/6.35±1.03 | NR | NR | China |
| 7 | LDH | Xu Fen 2025 | MOXI | CT | NR | 14.48±2.36/15.07±2.51 | 31.65±3.47/32.09±3.50 | China |
| 8 | LDH | Zhong Qunhua 2024 | CT+MOXI | CT | 8.24±0.60/ 8.41±0.78 | 9.96±1.24/10.18±1.32 | NR | China |
| 9 | LDH | Liu Wenlin 2024 | ACU+MOXI | ACU | 5.59±1.60/5.50±1.67 | 12.40±3.34/13.31±4.06 | 33.53±6.18/34.11±6.73 | China |
| 10 | LDH | Wu Yusheng 2025 | TCM+MOXI | CT | 6.40±1.30/6.53±1.25 | 14.43±3.76/14.73±4.31 | NR | China |
| 11 | LDH | Xu Shihai 2019 | TCM+MOXI | ACU | NR | 18.48±3.11/17.84±4.27 | NR | China |
| 12 | LDH | Yao Dong 2024 | TUINA+MOXI | CT+MOXI | 7.29±0.91/6.92±0.87 | NR | 28.74±5.97/28.61±6.27 | China |
| 13 | LDH | Chen Weiyi 2021 | ACU+MOXI | ACU+MOXI | 5.62±1.08/5.69±1.04 | NR | 20.52±6.12/20.38±6.09 | China |
| 14 | LDH | Liang Yuanyuan 2014 | TCM+MOXI | CT | 9.6±0.91/9.2±0.83 | 9.62±2.35/13.06±2.91 | NR | China |
| 15 | LDH | Deng Min 2022 | TCM+MOXI | CT | 5.45±1.50/5.33±1.68 | NR | 39.17±5.38/39.36±5.45 | China |
| 16 | LDH | Li Shengqiang 2015 | ACU+MOXI | ACU | 7.81±1.25/7.74±1.22 | NR | NR | China |
| 17 | LDH | Liu Yiyi 2022 | ACU+MOXI | ACU | NR | 11.9±2.4/11.5±2.7 | NR | China |
| 18 | LDH | Guo Hui 2023 | TCM+MOXI | TCM | NR | 13.65±2.11/14.05±2.06 | 69.32±5.87/68.85±5.49 | China |
| 19 | LDH | Wen Qi 2023 | MOXI | ACU | 5.54±1.56/5.89±1.79 | 13.95±3.75/14.61±4.12 | NR | China |
| 20 | LDH | Lin Mingqin 2021 | MOXI | CT | 6.56±2.38/6.81±2.94 | 13.39±3.36/13.69±3.19 | NR | China |
| 21 | LDH | Xie Wenhui 2023 | ACU+MOXI | CT | 6.78±1.13/6.93±0.88 | NR | NR | China |
| 22 | LDH | Zhang Jing 2026 | TCM+MOXI | TCM | 7.81±1.37/8.03±1.41 | 14.71±2.48/14.25±2.11 | 61.95±10.17/63.42±10.34 | China |
| 23 | LDH | Zhu Bin 2021 | MOXI | ACU | 8.05±0.77/7.95±0.72 | 6.31±2.05/6.62±2.11 | 35.14±3.86/34.83±3.71 | China |
| 24 | LDH | Yu Ansheng 2017 | ACU+MOXI | ACU | 6．17±1.64/6．33±1.9 | NR | NR | China |
| 25 | LDH | Yin Shaojian 2022 | TCM+MOXI | MOXI | 6.35±1.68/6.72±1.85 | NR | 6.96±2.18/7.57±2.32 | China |
| 26 | LDH | Xu Zhenkai 2018 | TUINA+MOXI | TUINA | 7.21±1.45/7.12±1.42 | 17.57±1.71/17.36±1.68 | NR | China |
| 27 | LDH | Jiang Yunqing 2023 | TCM+MOXI | CT | NR | NR | 36.12±5.14/36.18±5.26 | China |
| 28 | LDH | Feng Minhe 2021 | ACU+MOXI | ACU | 6.16±0.93/6.29±1.05 | NR | NR | China |
| 29 | LDH | Wang Guoshu 2017 | ACU+MOXI | ACU | NR | 10.90±3.67/10.50±3.18 | NR | China |
| 30 | LDH | Wang Jianying 2025 | ACU+MOXI | CT | 6.59±1.52/6.63±1.20 | NR | 35.96±5.26/35.30±5.30 | China |
| 31 | LDH | Sun Jian 2009 | ACU+MOXI | ACU | 6.46±1.48/6.37±1.54 | 11.33±3.17/11.76±3.29 | NR | China |
| 32 | LDH | Sun Jianbing 2025 | TCM+MOXI | CT | 5.79±0.21/5.80±0.43 | NR | NR | China |
| 33 | LDH | Xu Chenghua 2018 | TUINA+MOXI | TUINA | NR | 10.82±2.06/10.79±2.08 | NR | China |
| 34 | LDH | Gao Yuanguanyu 2021 | TCM+MOXI | CT | 5.34±1.63/5.41±1.54 | NR | 39.3±5.2/39.1±5.0 | China |
| 35 | LDH | Jiang Yuqin 2025 | ACU+MOXI | ACU | 6.62±0.79/6.58±0.74 | 15.04±3.17/14.85±3.26 | NR | China |
| 36 | LDH | Liu Chunyu 2021 | CT+MOXI | CT | 7.24±1.16/7.21±1.19 | NR | NR | China |
| 37 | LDH | Yin Zhao 2023 | TCM+MOXI | TCM | 7.98±1.02/7.89±0.98 | 16.12±3.56/16.32±3.20 | NR | China |
| 38 | LDH | Zhang Aili 2023 | TUINA+MOXI | CT | 7.19±0.46/7.21±0.49 | NR | NR | China |
| 39 | LDH | Hua Yuqi 2015 | TCM+MOXI | REHAB | 4.28±1.43/4.54±1.62 | NR | NR | China |
| 40 | LDH | Wu Zongxiang 2017 | TUINA+MOXI | TUINA | 7.93±1.3/7.90±1.30 | NR | NR | China |
| 41 | LDH | Chen Jing 2016 | TUINA+MOXI | TUINA | 7.4 ±1.1/7.3 ±1.4 | 13.6±2.1/13.4±2.5 | NR | China |
| 42 | LDH | Wang Hongfei 2020 | TUINA+MOXI | REHAB | 7.36±1.09/7.41±1.12 | 8.64±1.36/8.52±1.41 | NR | China |
| 43 | LDH | Zhao Huayun 2023 | TUINA+MOXI | REHAB | 7.18±1.01/7.20±1.03 | 16.09±2.16/16.05±2.14 | NR | China |
| 44 | LDH | Li Xiuxia 2019 | MOXI | CT | 7.51±1.14/7.46±1.18 | 10.23±3.33/10.16±3.16 | NR | China |
| 45 | LDH | Xu Mengxia 2019 | ACU+MOXI | ACU | 6.1±1.8/6.2±1.5 | NR | 25.3±3.8/25.9±4.2 | China |
| 46 | LDH | Pan Meng 2025 | ACU+MOXI | ACU | 5.84±0.95/5.51±0.98 | NR | 53.59±8.1/55.30±8.43 | China |
| 47 | LDH | Li Ju 2024 | TUINA+MOXI | MOXI | NR | 17.19±1.21/17.52±1.46 | 73.15±3.34/72.91±3.26 | China |
| 48 | LDH | Zhou Huaming 2024 | TUINA+MOXI | REHAB | 8.13±1.16/8.24±1.23 | NR | 15.18± 5.57/15.15±5.63 | China |
| 49 | LDH | Li Jia 2024 | TCM+MOXI | ACU | 5.58±0.76/5.69±0.84 | NR | NR | China |
| 50 | LDH | Xiong Qi 2024 | TUINA+MOXI | CT | 6.23±1.02/6.25±1.04 | 11.31±1.35/11.22±1.32 | NR | China |

Table S5 Detailed characteristics of the included studies

| **No.** | **Treatment Group Sample size** | **Control Group Sample size** | **Duration of disease** | **Age（mean±sd）** | **Sex (male/female)** | **Duration of intervention** | **Reasearch Ttpe** |
| --- | --- | --- | --- | --- | --- | --- | --- |
| 1 | 35 | 35 | NR | 53.26 ± 11.64/52.77 ± 11.89 | 41/29 | 20 | RCT |
| 2 | 60 | 60 | 3.20±0.50/3.10±0.50 | 43.10±5.30/42.70±5.30 | 76/44 | 3 | RCT |
| 3 | 52 | 52 | 5.45±1.07/5.39±1.18 | 46.96 ± 8.54 /47.13 ± 8.26 | 55/49 | 14 | RCT |
| 4 | 51 | 51 | 17.23±3.25/17.44±3.14 | 44.27±3.19/43.91±3.24 | 75/27 | 28 | RCT |
| 5 | 44 | 45 | 1.22±0.28/1.24 ±0.31 | 41.25±4.21/41.36±4.58 | 55/34 | 20 | RCT |
| 6 | 35 | 35 | 3/3 | 40.15±11.29/40.67±11.31 | 33/30 | 21 | RCT |
| 7 | 45 | 45 | NR | 62.98±5.30/62.36±5.19 | 48/42 | 28 | RCT |
| 8 | 35 | 35 | 10.95±1.08/10.98±1.05 | 61.37±2.1/61.42±2.1 | 41/29 | 14 | RCT |
| 9 | 32 | 36 | 1.32±0.39/1.25±0.23 | 49.02±6.88/48.65±8.3 | 38/30 | 14 | RCT |
| 10 | 30 | 30 | 1.03±0.05/1.04±0.07 | 54.47±11.04/54.27±10.96 | 32/28 | 14 | RCT |
| 11 | 45 | 45 | NR | 52.04±4.61/51.42±5.14 | 30/60 | 23 | RCT |
| 12 | 34 | 36 | 10/13 | 50.26 ± 1.85 | 30/40 | 14 | RCT |
| 13 | 30 | 30 | 1.12±0.80 | 41.83±10.80 | 30/30 | 28 | RCT |
| 14 | 30 | 30 | NR | 30-60 | 31/29 | 14 | RCT |
| 15 | 49 | 49 | 3.2２±0.4６/3.26±0.48 | 53.52±2.67/53.55 ± 2.64 | 63/45 | 14 | RCT |
| 16 | 39 | 36 | 0.39±0.07/0.34±0.07 | 57.64±10.75/58.82±10.84 | 42/33 | 30 | RCT |
| 17 | 30 | 30 | 1.00±0.14/1.01±0.14 | 48.97±5.27/49.22±5.50 | 18/42 | 10 | RCT |
| 18 | 45 | 45 | 4.63±1.02/5.03±1.10 | 55.71±5.32/54.95±5.68 | 40/50 | 28 | RCT |
| 19 | 40 | 40 | 1.52±0.46/1.45±0.41 | 38.26±8.15/39.65±8.59 | 47/43 | 14 | RCT |
| 20 | 30 | 30 | 1.14±0.35/1.08±0.39 | 38.26±8.15/39.65±8.59 | 35/25 | 18 | RCT |
| 21 | 30 | 30 | 1.79±0.40/1.79±0.39 | 49.02±6.23/48.83±5.92 | 34/26 | 30 | RCT |
| 22 | 50 | 50 | 1.97±0.52/2.06±0.33 | 46±4/46±5 | 62/38 | 28 | RCT |
| 23 | 42 | 42 | NR | 52.88±11.03/52.00±13.16 | 44/40 | 14 | RCT |
| 24 | 30 | 30 | NR | 47.7 ±13.27 | NR | 21 | RCT |
| 25 | 44 | 44 | 1.16±0.11/1.13±0.05 | 46.35 ± 8.76/45.05±7.41 | 53/35 | 30 | RCT |
| 26 | 80 | 80 | 0.53 ± 0.12 | 41.43±1.81/41.03±1.67 | 98/62 | 30 | RCT |
| 27 | 44 | 44 | 1.14±0.43/1.12±0.46 | 52.32±3.18/51.94±3.23 | 54/34 | 30 | RCT |
| 28 | 44 | 44 | 8.58±2.18/8.17±2.58 | 45.27±1.36/45.38±1.45 | 49/39 | 20 | RCT |
| 29 | 30 | 30 | 1.24±0.36/1.31±0.21 | 47±13/47±13 | 28/32 | 40 | RCT |
| 30 | 33 | 33 | NR | 54.99±10.99/55.89±10.89 | 35/31 | 14 | RCT |
| 31 | 45 | 45 | NR | 44.10±10.28/43.36±11.43 | 48/42 | 28 | RCT |
| 32 | 60 | 60 | NR | 44.5±10.4/46.5±10.5 | 81/39 | 14 | RCT |
| 33 | 33 | 32 | 3.25±0.72/3.23±0.69 | 50±4/49±4 | 30/35 | 21 | RCT |
| 34 | 43 | 43 | 1.30±0.27/1.28±0.26 | 35.4±3.4/35.2± 3.3 | 48/38 | 28 | RCT |
| 35 | 30 | 30 | 2.52±0.51/2.47±0.56 | 52.85±6.47/54.74±5.69 | 29/31 | 28 | RCT |
| 36 | 30 | 30 | NR | 52.42±3.17/52.38±3.25 | 41/19 | 28 | RCT |
| 37 | 38 | 38 | 4.23±1.02/4.32±1.10 | 45.22±3.47/45.17±3.51 | 42/34 | 14 | RCT |
| 38 | 40 | 40 | NR | 51.29±6.68/51.82±6.7 | 49/31 | 28 | RCT |
| 39 | 42 | 42 | 0.87±0.26/0.96±0.3 | 43.53±4.32/44.64±3.86 | 43/41 | 14 | RCT |
| 40 | 53 | 53 | 0.86±0.12/0.76±0.12 | 49.7±5.5/48.9±4.8 | 58/48 | 23 | RCT |
| 41 | 50 | 50 | 2.3±1.4/２.1±1 | 45.1±8.9/43.7±9.2 | 57/43 | 28 | RCT |
| 42 | 50 | 50 | 2.03±0.4/2.18±0.4 | 50.86±2.73/51.17±2.25 | 53/47 | 20 | RCT |
| 43 | 49 | 49 | 0.80±0.10/0.80±0.10 | 40.58±3.21/40.63±3.19 | 51/47 | 28 | RCT |
| 44 | 49 | 49 | 3.14±0.41/3.16±0.44 | 59.71±4.20/59.68±4.16 | 47/51 | 10 | RCT |
| 45 | 50 | 50 | 5.8±1.83/5.8±1.83 | 44.2±5.38/45.1±5.69 | 60/40 | 24 | RCT |
| 46 | 54 | 53 | 0.82 ± 0.18/0.87 ± 0.23 | 61.7±7.4/60.0±6.6 | 37/70 | 28 | RCT |
| 47 | 33 | 33 | 2.34±0.51/2.18±0.36 | 52.85±1.42/52.69±1.16 | 41/25 | 21 | RCT |
| 48 | 38 | 38 | 4±0.4/4±0.3 | 45.38±3.59/44.38±2.98 | 39/37 | 7 | RCT |
| 49 | 150 | 150 | 2.62±0.78/2.58±3.78 | 57.16±7.21/56.32±6.45 | 155/145 | 10 | RCT |
| 50 | 44 | 44 | 2.04±0.39/2.11±0.41 | 48.52±4.19/48.48±4.15 | 48/40 | 28 | RCT |

**Table of P-values for Global Inconsistency Tests of Each Outcome in the Main Analysis of LDH Patients (Table S6)**

| **Outcome** | **P value (Global Inconsistency Test)** |
| --- | --- |
| Primary analysis:ODI outcome group | 0.9947 |
| Primary analysis:VAS outcome group | 0.4991 |
| Primary analysis:JOA outcome group | 0.319 |
| Primary analysis:cured group | 0.1984 |

Node-splitting analysis in the primary analysis

**Node-splitting analysis based on the ODI score group in the primary analysis of patients with LDH (Table S7)**

| **Side** | **Coef.** | **Std.Err.** | **Coef.** | **Std.Err.** | **Coef.** | **Std.Err.** | **P>z** | **tau** |
| --- | --- | --- | --- | --- | --- | --- | --- | --- |
| ACU VS ACU+MOXI | -5.359263 | 0.7785186 | -5.69901 | 11.74181 | 0.3397467 | 11.76756 | 0.977 | 0.9188049 |
| ACU+MOXI VS CT | 10.11002 | 0.9268328 | 9.231986 | 16.55652 | 0.8780352 | 16.58267 | 0.958 | 0.918654 |
| CT VS CT+MOXI | -1.150001 | 1.133245 | -0.96081 | 2.12459 | -0.1891913 | 2.40793 | 0.937 | 1.049417 |
| CT VS MOXI | -3.090002 | 1.230206 | -3.274908 | 1.103345 | 0.1849063 | 1.652506 | 0.911 | 1.071113 |
| CT VS TCM+MOXI | -4.686284 | 0.6383762 | -4.595894 | 1.545742 | -0.0903897 | 1.672558 | 0.957 | 1.079875 |
| CT+MOXI VS TUINA+MOXI | -5.490008 | 1.558471 | -5.298043 | 1.836046 | -0.1919643 | 2.408299 | 0.936 | 1.049454 |
| MOXI VS TCM+MOXI | -1.440009 | 1.099815 | -1.534907 | 1.262411 | 0.0948982 | 1.674296 | 0.955 | 1.080139 |
| MOXI VS TUINA+MOXI | -3.280002 | 1.156412 | -3.47218 | 2.112287 | 0.1921777 | 2.408123 | 0.936 | 1.049452 |
| REHAB VS TUINA+MOXI | -4.88 | 1.34967 | -3.547268 | 272.9117 | -1.332732 | 272.915 | 0.996 | 0.9173273 |
| TCMT VS CM+MOXI | -6.568766 | 0.8542742 | 0.026239 | 159.1672 | -6.595005 | 159.169 | 0.967 | 0.9173797 |

**Node-splitting analysis based on the VAS score group in the primary analysis of patients with LDH (Table S8)**

| **Side** | **Coef.** | **Std. Err.** | **Coef.** | **Std. Err.** | **Coef.** | **Std. Err.** | **P>z** | **tau** |
| --- | --- | --- | --- | --- | --- | --- | --- | --- |
| ACU VS ACU+MOXI | -1.169567 | 0.3967051 | -2.010164 | 1.020286 | 0.8405964 | 1.094699 | 0.443 | 1.105651 |
| ACU VS MOXI | -1.507809 | 0.790465 | -0.1024856 | 0.7684884 | -1.405324 | 1.102453 | 0.202 | 1.08849 |
| ACU VSTCM+MOXI | -0.5317176 | 0.7917731 | -0.975403 | 0.7360342 | 0.4436854 | 1.081042 | 0.681 | 1.114237 |
| ACU+MOXI VS CT | 2.42661 | 0.7867238 | 1.57612 | 0.7676574 | 0.8504898 | 1.099202 | 0.439 | 1.105783 |
| CT VS CT+MOXI | -2.169768 | 0.6471842 | -1.364516 | 1.33447 | -0.8052523 | 1.483139 | 0.587 | 1.111612 |
| CT VS MOXI | -1.822983 | 0.6458142 | -1.112085 | 0.7009126 | -0.7108984 | 0.9530767 | 0.456 | 1.107156 |
| CT VS TCM+MOXI | -1.274976 | 0.4566741 | -2.021888 | 0.7409739 | 0.7469127 | 0.8703992 | 0.391 | 1.104172 |
| CT VS TUINA+MOXI | -1.109999 | 1.094873 | -2.686946 | 0.7358932 | 1.576947 | 1.319199 | 0.232 | 1.091018 |
| CT+MOXI VS TUINA+MOXI | -0.6500063 | 1.139142 | 0.1520986 | 0.9574193 | -0.8021048 | 1.488051 | 0.59 | 1.11172 |
| MOXI VS TCM+MOXI | -1.450003 | 1.109629 | 0.4226822 | 0.5844229 | -1.872685 | 1.254124 | 0.135 | 1.07878 |
| MOXI VS TUINA+MOXI | -1.669981 | 1.112855 | -0.1446527 | 0.8416275 | -1.525329 | 1.395274 | 0.274 | 1.095212 |
| REHAB VS TCM+MOXI | -1.72 | 1.150945 | -1.206166 | 0.9604282 | -0.5138335 | 1.499032 | 0.732 | 1.114556 |
| REHAB VS TUINA+MOXI | -2.058713 | 0.5621454 | -2.588294 | 1.392102 | 0.5295806 | 1.501303 | 0.724 | 1.114608 |
| TCM VS TCM+MOXI | -1.805186 | 0.7890999 | -1.536925 | 34.76522 | -0.2682618 | 34.77405 | 0.994 | 1.099259 |
| TUINA VS TUINA+MOXI | -1.807146 | 0.6397438 | -2.974443 | 21.93435 | 1.167297 | 21.94366 | 0.958 | 1.099364 |

| **Side** | **Coef.** | **Std.Err.** | **Coef.** | **Std.Err.** | **Coef.** | **Std.Err.** | **P>z** | **tau** |
| --- | --- | --- | --- | --- | --- | --- | --- | --- |
| ACU VS MOXI | 5.180632 | 1.412872 | 2.942216 | 1.672259 | 2.238416 | 2.189826 | 0.307 | 1.502969 |
| ACU VS TCM+MOXI | 2.071814 | 1.182952 | 4.249879 | 1.843389 | -2.178065 | 2.189572 | 0.32 | 1.502846 |
| CT VS CT+MOXI | 11.93 | 1.557059 | 2.00849 | 113.8923 | 9.921511 | 113.9023 | 0.931 | 1.494589 |
| CT VS MOXI | 4.921557 | 0.8521564 | 7.115318 | 2.019426 | -2.193762 | 2.191834 | 0.317 | 1.502965 |
| CT VS TCM+MOXI | 4.027564 | 0.8334618 | 1.844586 | 2.026605 | 2.182978 | 2.191297 | 0.319 | 1.502927 |
| MOXI VS TUINA+MOXI | 2.580007 | 1.562232 | -2.406801 | 23.26045 | 4.986808 | 23.31282 | 0.831 | 1.49502 |
| REHAB VS TUINA+MOXI | 4.782333 | 0.9223253 | 13.60635 | 96.08805 | -8.824019 | 96.09243 | 0.927 | 1.494637 |
| TCM VS TCM+MOXI | 4.246371 | 0.9724875 | 5.40917 | 128.7045 | -1.162799 | 128.7081 | 0.993 | 1.494546 |

**Node-splitting analysis based on the JOA score group in the primary analysis of patients with LDH(Table S9)**

**Node-splitting analysis based on the cure rate group in the primary analysis of patients with LDH (Table S10)**

| **Side** | **Coef.** | **Std.Err.** | **Coef.** | **Std.Err.** | **Coef.** | **Std.Err.** | **P>z** | **tau** |
| --- | --- | --- | --- | --- | --- | --- | --- | --- |
| ACU VS MOXI | 0.4964369 | 0.2547682 | 1.622593 | 0.7637795 | -1.126156 | 0.8051497 | 0.162 | 8.00E-09 |
| ACU VS TCM+MOXI | 1.704748 | 0.7305931 | 0.5673606 | 0.3434595 | 1.137387 | 0.8072984 | 0.159 | 1.58E-10 |
| CT VS CT+MOXI | 0.5108039 | 0.1781734 | 1.373108 | 0.8013779 | -0.8623039 | 0.8209546 | 0.294 | 5.50E-10 |
| CT VS MOXI | 0.492466 | 0.1800441 | -0.5592246 | 0.5618068 | 1.051691 | 0.5899488 | 0.075 | 2.50E-10 |
| CT VS TCM+MOXI | 0.5196577 | 0.1493453 | 1.655481 | 0.7931175 | -1.135824 | 0.8070589 | 0.159 | 1.43E-09 |
| CT+MOXI VS TUINA+MOXI | 0.3084557 | 0.1657301 | 1.172874 | 0.8045426 | -0.8644179 | 0.8214392 | 0.293 | 4.81E-11 |
| MOXI VS TUINA+MOXI | 1.252737 | 0.7653167 | 0.3850032 | 0.2999673 | 0.8677337 | 0.822005 | 0.291 | 5.69E-10 |
| TCM VS TCM+MOXI | 0.2513144 | 0.2005284 | 1.562368 | 59.18242 | -1.311053 | 59.18258 | 0.982 | 2.06E-08 |
| TUINA VS TUINA+MOXI | 0.4522193 | 0.1595045 | 2.256201 | 42.01657 | -1.803982 | 42.01657 | 0.966 | 3.46E-08 |

主分析环不一致性分析Loop-Specific Inconsistency Test for the Primary Analysis

**Loop inconsistency analysis of the ODI score group based on the primary analysis in patients with LDH.**

**(Table S11)**

| **Loop** | **IF** | **seIF** | **z_value** | **p_value** | **CI_95** | **Loop_Heterog_tau2** |
| --- | --- | --- | --- | --- | --- | --- |
| CT-CT+MOXI-MOXI-TUINA+MOXI | 0.27 | 1.453 | 0.186 | 0.853 | (0.00,3.12) | 0 |
| CT-MOXI-TCM+MOXI | 0.146 | 2.041 | 0.072 | 0.943 | (0.00,4.15) | 1.469 |

**Loop inconsistency analysis of the VAS score group based on the primary analysis in patients with LDH.** **(Table S12)**

| **Loop** | **IF** | **seIF** | **z_value** | **p_value** | **CI_95** | **Loop_Heterog_tau2** |
| --- | --- | --- | --- | --- | --- | --- |
| CT-MOXI-TUINA+MOXI | 2.382 | 0.766 | 3.108 | 0.002 | (0.00,3.88) | 0.225 |
| ACU-MOXI-TCM+MOXI | 2.354 | 0.542 | 4.345 | 0 | (0.00,3.42) | 0.054 |
| CT-MOXI-TCM+MOXI | 1.975 | 0.681 | 2.9 | 0.004 | (0.00,3.31) | 0.255 |
| CT-CT+MOXI-TUINA+MOXI | 1.708 | 3.263 | 0.523 | 0.601 | (0.00,8.10) | 4.531 |
| ACU-ACU+MOXI-CT-MOXI | 0.945 | 1.231 | 0.767 | 0.443 | (0.00,3.36) | 1.003 |
| ACU-ACU+MOXI-CT-TCM+MOXI | 0.519 | 1.046 | 0.497 | 0.62 | (0.00,2.57) | 0.829 |
| CT-REHAB-TCM+MOXI-TUINA+MOXI | 0.502 | 1.714 | 0.293 | 0.769 | (0.00,3.86) | 1.166 |
| MOXI-REHAB-TCM+MOXI-TUINA+MOXI | 0.133 | 2.992 | 0.044 | 0.965 | (0.00,6.00) | 2.679 |

**Loop inconsistency analysis of the JOA score group based on the primary analysis in patients with LDH.**

**(Table S13)**

| **Loop** | **IF** | **seIF** | **z_value** | **p_value** | **CI_95** | **Loop_Heterog_tau2** |  |
| --- | --- | --- | --- | --- | --- | --- | --- |
| ACU-CT-MOXI-TCM+MOXI | 2.292 | 2.68 | 0.855 | 0.392 | (0.00,7.55) | 3.826 |  |

**Loop inconsistency analysis of the cure rate group based on the primary analysis in patients with LDH.(Table S14)**

| **Loop** | **IF** | **seIF** | **z_value** | **p_value** | **CI_95** | **Loop_Heterog_tau2** |
| --- | --- | --- | --- | --- | --- | --- |
| ACU-CT-MOXI-TCM+MOXI | 1.181 | 0.808 | 1.461 | 0.144 | (0.00,2.77) | 0 |
| CT-CT+MOXI-MOXI-TUINA+MOXI | 0.926 | 0.823 | 1.125 | 0.261 | (0.00,2.54) | 0 |

SUCRA Primary analysis

**Ranking of treatment effects based on the ODI score group in the primary analysis of patients with LDH (Table S15)**

| **Treatment** | **SUCRA** | **PrBest** | **MeanRank** |
| --- | --- | --- | --- |
| ACU | 67.9 | 0 | 3.6 |
| ACU+MOXI | 99.9 | 99.6 | 1 |
| CT | 15.6 | 0 | 7.8 |
| CT+MOXI | 28.7 | 0 | 6.7 |
| MOXI | 49.6 | 0 | 5 |
| REHAB | 33.5 | 0 | 6.3 |
| TCM | 0.9 | 0 | 8.9 |
| TCM+MOXI | 68.5 | 0 | 3.5 |
| TUINA+MOXI | 85.2 | 0.4 | 2.2 |

**Ranking of treatment effects based on the VAS score group in the primary analysis of patients with LDH (Table S16)**

| **Treatment** | **SUCRA** | **PrBest** | **MeanRank** |
| --- | --- | --- | --- |
| TUINA+MOXI | 87.6 | 41.2 | 2.1 |
| ACU+MOXI | 82.6 | 26.7 | 2.6 |
| CT+MOXI | 81.9 | 27.5 | 2.6 |
| MOXI | 64.9 | 2.8 | 4.2 |
| TCM+MOXI | 64.4 | 1.7 | 4.2 |
| ACU | 37.7 | 0 | 6.6 |
| TUINA | 30.6 | 0.1 | 7.2 |
| REHAB | 20.3 | 0 | 8.2 |
| CT | 17 | 0 | 8.5 |
| TCM | 13.1 | 0.1 | 8.8 |

**Ranking of treatment effects based on the JOA score group in the primary analysis of patients with LDH (Table S17)**

| **Treatment** | **SUCRA** | **PrBest** | **MeanRank** |
| --- | --- | --- | --- |
| ACU | 22 | 0 | 8 |
| ACU+MOXI | 58.6 | 0 | 4.7 |
| CT | 10.3 | 0 | 9.1 |
| CT+MOXI | 99.6 | 96.1 | 1 |
| MOXI | 71.5 | 0 | 3.6 |
| REHAB | 41.2 | 0 | 6.3 |
| TCM | 6.3 | 0 | 9.4 |
| TCM+MOXI | 50.5 | 0 | 5.5 |
| TUINA | 51.8 | 0 | 5.3 |
| TUINA+MOXI | 88.1 | 3.9 | 2.1 |

**Ranking of treatment effects based on the cure rate group in the primary analysis of patients with LDH (Table S18)**

| **Treatment** | **SUCRA** | **PrBest** | **MeanRank** |
| --- | --- | --- | --- |
| ACU | 4.9 | 0 | 8.6 |
| ACU+MOXI | 29.4 | 0.9 | 6.7 |
| CT | 16.7 | 0 | 7.7 |
| CT+MOXI | 70.6 | 0.6 | 3.4 |
| MOXI | 55.5 | 1.7 | 4.6 |
| TCM | 45.3 | 2 | 5.4 |
| TCM+MOXI | 72.7 | 8.6 | 3.2 |
| TUINA | 57.6 | 0.2 | 4.4 |
| TUINA+MOXI | 97.2 | 85.9 | 1.2 |

**Table of P values for the Global Inconsistency Test of Each Outcome in the Subgroup Analysis of Patients with LDH(Table S19)**

| **Outcome** | **P value (Global Inconsistency Test)** |
| --- | --- |
| Subgroup analysis:ODI outcome group | 0.2252 |
| Subgroup analysis:VAS outcome group | 0.7736 |
| Subgroup analysis:JOA outcome group | 0.9879 |
| Subgroup analysis:cured group | 0.4854 |

Node-splitting analysis in the subgroup analysis

**Node-splitting analysis based on the ODI score group in the subgroup analysis of patients with LDH (Table S20)**

| **Side** | **Coef.** | **Std.Err.** | **Coef.** | **Std.Err.** | **Coef.** | **Std.Err.** | **P>z** | **tau** |
| --- | --- | --- | --- | --- | --- | --- | --- | --- |
| ACU VS CM | -5.632167 | 2.467128 | -5.280648 | 5.369972 | -0.3515192 | 5.913386 | 0.953 | 3.107418 |
| ACU VS WN | -5.500001 | 3.159321 | -5.110741 | 5.518782 | -0.3892602 | 6.359109 | 0.951 | 3.127956 |
| CM VS CT | 4.997926 | 1.102702 | -1.091712 | 3.28469 | 6.089638 | 3.46533 | 0.079 | 2.63418 |
| CM VS HM | -4.575898 | 2.1021 | 0.7619536 | 2.630431 | -5.337852 | 3.367214 | 0.113 | 2.730049 |
| CT VS HM | -3.089998 | 2.703926 | -9.354095 | 2.194534 | 6.264097 | 3.482413 | 0.072 | 2.635355 |
| HM VS TCM | 8.22999 | 3.260209 | 7.855959 | 5.465481 | 0.3740308 | 6.363994 | 0.953 | 3.128196 |
| TCM VS WN | -5.339974 | 3.192287 | -5.724119 | 5.506413 | 0.3841448 | 6.364839 | 0.952 | 3.128256 |

**Node-splitting analysis based on the VAS score group in the subgroup analysis of patients with LDH (Table S21)**

| **Side** | **Coef.** | **Std. Err.** | **Coef.** | **Std. Err.** | **Coef.** | **Std. Err.** | **P>z** | **tau** |
| --- | --- | --- | --- | --- | --- | --- | --- | --- |
| ACU VS CM | -0.9173497 | 0.3834341 | -1.14204 | 0.5604205 | 0.2246905 | 0.6790382 | 0.741 | 0.922897 |
| ACU VS HM | -1.327079 | 0.6655343 | -0.7473351 | 0.5041695 | -0.5797436 | 0.8349264 | 0.487 | 0.9172263 |
| ACU VS WN | -1.30672 | 0.473409 | -1.467067 | 0.5938029 | 0.1603469 | 0.7594301 | 0.833 | 0.9240287 |
| CM VS CT | 1.590345 | 0.3409784 | 0.6544263 | 0.5380758 | 0.9359189 | 0.6370105 | 0.142 | 0.8933074 |
| CM VS HM | -0.6549065 | 0.6678985 | 0.3398275 | 0.4482774 | -0.9947339 | 0.8043884 | 0.216 | 0.902015 |
| CM VS TUINA | 1.592306 | 0.6616873 | 1.885827 | 1.022897 | -0.2935211 | 1.218262 | 0.81 | 0.9242195 |
| CT VS HM | -0.8830658 | 0.5377177 | -1.656082 | 0.5086191 | 0.773016 | 0.740158 | 0.296 | 0.908761 |
| CT VS TF | -2.76254 | 0.5324307 | -3.859511 | 0.8885908 | 1.096971 | 1.035886 | 0.29 | 0.908353 |
| CT VS WN | -1.778813 | 0.6646893 | -1.658361 | 0.494378 | -0.1204521 | 0.8283855 | 0.884 | 0.9247957 |
| HM VS REHAB | 1.066493 | 0.6607794 | 1.951073 | 0.7100469 | -0.8845805 | 0.9698958 | 0.362 | 0.9123844 |
| REHAB VS TF | -4.230003 | 0.9335767 | -2.647616 | 0.7200432 | -1.582386 | 1.178994 | 0.18 | 0.8987489 |
| REHAB VS WN | -1.815264 | 0.6580047 | -1.973734 | 0.7161342 | 0.1584704 | 0.9725205 | 0.871 | 0.9247945 |
| TCM VS TF | -2.419985 | 0.9553122 | -2.590636 | 1.109403 | 0.1706516 | 1.464032 | 0.907 | 0.9247236 |
| TCM VS WN | -1.209996 | 0.9339186 | -1.042911 | 1.127226 | -0.1670855 | 1.463844 | 0.909 | 0.9247205 |
| TUINA VS WN | -2.229997 | 0.9321029 | -1.937512 | 0.7853562 | -0.2924843 | 1.21885 | 0.81 | 0.9242349 |

**Node-splitting analysis based on the JOA score group in the subgroup analysis of patients with LDH (Table S22)**

| **Side** | **Coef.** | **Std.Err.** | **Coef.** | **Std.Err.** | **Coef.** | **Std.Err.** | **P>z** | **tau** |
| --- | --- | --- | --- | --- | --- | --- | --- | --- |
| ACU VS CM | 2.73835 | 1.098938 | 3.647258 | 2.416155 | -0.9089088 | 2.654211 | 0.732 | 2.297294 |
| ACU VS TF | 4.1 | 2.634272 | 4.438939 | 1.852398 | -0.3389382 | 3.220367 | 0.916 | 2.300519 |
| ACU VS WN | 4.418815 | 1.489171 | 3.277752 | 2.161141 | 1.141064 | 2.624371 | 0.664 | 2.293026 |
| CM VS CT | -5.007266 | 1.716026 | -4.083497 | 2.03714 | -0.9237691 | 2.663589 | 0.729 | 2.297718 |
| CM VS TUINA | -4.048107 | 1.312517 | -5.799805 | 54.59602 | 1.751698 | 54.61176 | 0.974 | 2.244891 |
| CT VS HM | 4.031917 | 1.69128 | 6.088175 | 2.324374 | -2.056258 | 2.874606 | 0.474 | 2.27286 |
| CT VS TF | 6.327993 | 1.346887 | 5.277519 | 2.281208 | 1.050475 | 2.64916 | 0.692 | 2.295879 |
| CT VS WN | 5.664019 | 1.797072 | 5.884301 | 1.676694 | -0.2202821 | 2.458045 | 0.929 | 2.304243 |
| HM VS REHAB | -4.37 | 2.364505 | -3.855974 | 2.502945 | -0.5140258 | 3.443198 | 0.881 | 2.307968 |
| HM VS TCM | -4.629998 | 2.385309 | -2.341869 | 2.356759 | -2.288129 | 3.353209 | 0.495 | 2.277798 |
| REHAB VS WN | 5.041353 | 1.68429 | 5.558267 | 3.001666 | -0.5169143 | 3.441939 | 0.881 | 2.30792 |
| TCM VS TF | 4.060006 | 2.494463 | 5.403087 | 2.304972 | -1.343082 | 3.396358 | 0.693 | 2.29518 |
| TCM VS WN | 4.029997 | 2.390411 | 4.932555 | 2.244482 | -0.9025582 | 3.278988 | 0.783 | 2.302802 |

**Node-splitting analysis based on the cure rate group in the subgroup analysis of patients with LDH (Table S23)**

| **Side** | **Coef.** | **Std.Err.** | **Coef.** | **Std.Err.** | **Coef.** | **Std.Err.** | **P>z** | **tau** |
| --- | --- | --- | --- | --- | --- | --- | --- | --- |
| ACU VS CM | 0.3295896 | 0.1179528 | 0.5426057 | 0.3182252 | -0.2130161 | 0.339382 | 0.53 | 3.49e-10 |
| ACU VS WN | 0.4964369 | 0.2547682 | 0.282911 | 0.2244121 | 0.2135259 | 0.3395108 | 0.529 | 1.85e-07 |
| CM VS CT | -0.5706925 | 0.1796378 | -0.3159834 | 0.2082982 | -0.2547091 | 0.2750592 | 0.354 | 2.27e-08 |
| CM VS HM | 0.3084688 | 0.1657301 | -0.1398434 | 0.3081002 | 0.4483122 | 0.3498477 | 0.2 | 1.94e-07 |
| CM VS TUINA | -0.549041 | 0.2233275 | -0.2596865 | 0.2914403 | -0.2893546 | 0.3671661 | 0.431 | 7.21e-08 |
| CT VS HM | 0.4054614 | 0.2687416 | 0.8536888 | 0.2240289 | -0.4482274 | 0.349873 | 0.2 | 8.72e-11 |
| CT VS TF | 0.5108256 | 0.1781742 | 0.1489118 | 42.8276 | 0.3619138 | 42.82848 | 0.993 | 8.99e-10 |
| CT VS WN | 0.4924744 | 0.1800441 | 0.462687 | 0.262297 | 0.0297874 | 0.3181441 | 0.925 | 2.37e-06 |
| HM VS REHAB | -0.5108256 | 0.4680252 | -1.190903 | 130.2125 | 0.6800774 | 130.2129 | 0.996 | 1.79e-08 |
| HM VS TCM | -0.2513144 | 0.2005284 | -1.142142 | 59.11851 | 0.8908278 | 59.11868 | 0.988 | 9.53e-10 |
| TUINA VS WN | 0.3513971 | 0.2278875 | 0.6407857 | 0.2878946 | -0.2893886 | 0.3671728 | 0.431 | 1.36e-10 |

Loop-Specific Inconsistency Analysis in the Subgroup Analysis

**Loop inconsistency analysis based on the ODI score group in the subgroup analysis of patients with LDH (Table S24)**

| **Loop** | **IF** | **seIF** | **z_value** | **p_value** | **CI_95** | | **Loop_Heterog_tau2** | |
| --- | --- | --- | --- | --- | --- | --- | --- | --- |
| CM-CT-HM | 6.476 | 3.903 | 1.659 | 0.097 | | (0.00,14.13) | | 8.467 |

**Loop inconsistency analysis based on the VAS score group in the subgroup analysis of patients with LDH (Table S25)**

| **Loop** | **IF** | **seIF** | **z_value** | **p_value** | **CI_95** | **Loop_Heterog_tau2** |
| --- | --- | --- | --- | --- | --- | --- |
| REHAB-TCM-TF-WN | 1.582 | 0.377 | 4.198 | 0 | (0.00,2.32) | 0 |
| CT-REHAB-TF-WN | 1.437 | 1.812 | 0.793 | 0.428 | (0.00,4.99) | 1.368 |
| CM-CT-HM | 1.359 | 1.115 | 1.219 | 0.223 | (0.00,3.54) | 1.217 |
| CT-HM-REHAB-TF | 1.283 | 1.518 | 0.845 | 0.398 | (0.00,4.26) | 1.015 |
| ACU-CT-HM-WN | 0.931 | 0.666 | 1.398 | 0.162 | (0.00,2.24) | 0.243 |
| ACU-HM-REHAB-WN | 0.794 | 0.921 | 0.862 | 0.389 | (0.00,2.60) | 0.452 |
| CM-CT-TUINA-WN | 0.442 | 1.782 | 0.248 | 0.804 | (0.00,3.93) | 1.462 |
| ACU-CM-TUINA-WN | 0.263 | 1.015 | 0.259 | 0.796 | (0.00,2.25) | 0.517 |
| ACU-CM-HM | 0.251 | 0.774 | 0.324 | 0.746 | (0.00,1.77) | 0.457 |
| CT-TCM-TF-WN | 0.236 | 2.117 | 0.111 | 0.911 | (0.00,4.39) | 1.548 |
| ACU-CM-CT-WN | 0.201 | 1.063 | 0.189 | 0.85 | (0.00,2.28) | 1.035 |
| CT-HM-REHAB-WN | 0.127 | 0.923 | 0.137 | 0.891 | (0.00,1.94) | 0.436 |

**Loop inconsistency analysis based on the JOA score group in the subgroup analysis of patients with LDH (Table S26)**

| **Loop** | **IF** | **seIF** | **z_value** | **p_value** | **CI_95** | **Loop_Heterog_tau2** |
| --- | --- | --- | --- | --- | --- | --- |
| CT-HM-TCM-TF | 2.839 | 7.966 | 0.356 | 0.722 | (0.00,18.45) | 21.766 |
| CT-HM-TCM-WN | 2.25 | 3.854 | 0.584 | 0.559 | (0.00,9.80) | 4.364 |
| ACU-CT-TF-WN | 1.096 | 5.778 | 0.19 | 0.85 | (0.00,12.42) | 14.134 |
| CT-HM-REHAB-WN | 0.961 | 3.799 | 0.253 | 0.8 | (0.00,8.41) | 5.258 |
| ACU-CM-CT-WN | 0.839 | 1.89 | 0.444 | 0.657 | (0.00,4.54) | 1.371 |
| CT-TCM-TF-WN | 0.685 | 7.931 | 0.086 | 0.931 | (0.00,16.23) | 21.497 |
| HM-REHAB-TCM-WN | 0.682 | 1.154 | 0.591 | 0.555 | (0.00,2.94) | 0 |
| ACU-TCM-TF-WN | 0.136 | 2.653 | 0.051 | 0.959 | (0.00,5.34) | 0.985 |
| ACU-CM-CT-TF | 0.018 | 4.732 | 0.004 | 0.997 | (0.00,9.29) | 9.948 |

**Loop inconsistency analysis based on the cure rate group in the subgroup analysis of patients with LDH (Table S27)**

| **Loop** | **IF** | **seIF** | **z_value** | **p_value** | **CI_95** | **Loop_Heterog_tau2** |
| --- | --- | --- | --- | --- | --- | --- |
| CM-CT-HM | 0.474 | 0.363 | 1.304 | 0.192 | (0.00,1.19) | 0 |
| ACU-CM-TF-WN | 0.364 | 0.554 | 0.658 | 0.511 | (0.00,1.45) | 0 |
| ACU-CM-CT-WN | 0.245 | 0.433 | 0.567 | 0.571 | (0.00,1.09) | 0 |
| CM-CT-TF-WN | 0.119 | 0.408 | 0.293 | 0.77 | (0.00,0.92) | 0 |

SUCRA Subgroup analysis

**Ranking of treatment effects based on the ODI score group in the subgroup analysis of patients with LDH(Table S28)**

| **Treatm~t** | **SUCRA** | **PrBest** | **MeanRank** |
| --- | --- | --- | --- |
| ACU | 16.9 | 0 | 5.2 |
| CM | 71 | 5 | 2.5 |
| CT | 28.4 | 0 | 4.6 |
| HM | 94.8 | 77 | 1.3 |
| TCM | 17.8 | 0 | 5.1 |
| WN | 71.1 | 18 | 2.4 |

**Ranking of treatment effects based on the VAS score group in the subgroup analysis of patients with LDH(Table S29)**

| **Treatm~t** | **SUCRA** | **PrBest** | **MeanRank** |
| --- | --- | --- | --- |
| ACU | 36.7 | 0 | 6.1 |
| CM | 69.4 | 0 | 3.5 |
| CT | 22.3 | 0 | 7.2 |
| HM | 68.1 | 0.1 | 3.6 |
| REHAB | 15.9 | 0 | 7.7 |
| TCM | 43 | 0 | 5.6 |
| TF | 99.9 | 99.3 | 1 |
| TUINA | 12.1 | 0 | 8 |
| WN | 82.6 | 0.6 | 2.4 |

**Ranking of treatment effects based on the JOA score group in the subgroup analysis of patients with LDH(Table S30)**

| **Treatm~t** | **SUCRA** | **PrBest** | **MeanRank** |
| --- | --- | --- | --- |
| TF | 89.7 | 48.4 | 1.8 |
| WN | 87.3 | 34.1 | 2 |
| HM | 73.7 | 10.2 | 3.1 |
| CM | 72.3 | 7.3 | 3.2 |
| ACU | 38.4 | 0 | 5.9 |
| TCM | 30.8 | 0 | 6.5 |
| REHAB | 22.9 | 0 | 7.2 |
| TUINA | 21.8 | 0 | 7.3 |
| CT | 13.1 | 0 | 7.9 |

**Ranking of treatment effects based on the cure rate group in the subgroup analysis of patients with LDH(Table S31)**

| **Treatm~t** | **SUCRA** | **PrBest** | **MeanRank** |
| --- | --- | --- | --- |
| ACU | 26 | 0 | 6.9 |
| CM | 65.6 | 2.4 | 3.8 |
| CT | 14.4 | 0 | 7.8 |
| HM | 90.7 | 49.9 | 1.7 |
| REHAB | 37.5 | 11.4 | 6 |
| TCM | 59.3 | 7.5 | 4.3 |
| TF | 70.8 | 19.2 | 3.3 |
| TUINA | 17.4 | 0 | 7.6 |
| WN | 68.4 | 9.5 | 3.5 |

Meta-regression in the primary analysis

**Meta-regression analysis of the ODI score group in the primary analysis of LDH: intervention duration(Table S32)**

|  |  | **Coefficient** | **Std. err.** | **z** | **P>z** | **[95% conf. interval]** | |
| --- | --- | --- | --- | --- | --- | --- | --- |
| ACU VS ACU+MOXI | cov1 | 0.2119128 | 0.2149387 | 0.99 | 0.324 | -0.2093593 | 0.633185 |
| ACU VS CT | cov1 | 0.013195 | 1.498459 | 0.01 | 0.993 | -2.923731 | 2.950121 |
| ACU VS CT+MOXI | cov1 | -0.0235227 | 4.61217 | -0.01 | 0.996 | -9.063209 | 9.016164 |
| ACU VSMOXI | cov1 | 0.1715245 | 1.904198 | 0.09 | 0.928 | -3.560636 | 3.903685 |
| ACU VS TCM+MOXI | cov1 | 0.0780284 | 1.500992 | 0.05 | 0.959 | -2.863861 | 3.019918 |
| ACU VS TUINA+MOXI | cov1 | -0.1786174 | 8.971588 | -0.02 | 0.984 | -17.76261 | 17.40537 |

**Meta-regression analysis of the ODI score group in the primary analysis of LDH:age(Table S33)**

|  |  | **Coefficient** | **Std. err.** | **z** | **P>z** | **[95% conf. interval]** | |
| --- | --- | --- | --- | --- | --- | --- | --- |
| ACU VS ACU+MOXI | age | 0.0755328 | 0.1063799 | 0.71 | 0.478 | -0.1329681 | 0.2840336 |
| ACU VS CT | age | 0.1591028 | 2.299371 | 0.07 | 0.945 | -4.347581 | 4.665786 |
| ACU VS CT+MOXI | age | 0.2041817 | 7.150027 | 0.03 | 0.977 | -13.80961 | 14.21798 |
| ACU VS MOXI | age | 0.1629556 | 2.300292 | 0.07 | 0.944 | -4.345535 | 4.671446 |
| ACU VS TCM | age | 0.5934562 | 2.308635 | 0.26 | 0.797 | -3.931385 | 5.118297 |
| ACU VS TCM+MOXI | age | 0.2723612 | 2.300438 | 0.12 | 0.906 | -4.236415 | 4.781137 |
| ACU VS TUINA+MOXI | age | 0.1395468 | 19.01863 | 0.01 | 0.994 | -37.13629 | 37.41538 |

**Meta-regression analysis of the ODI score group in the primary analysis of LDH: disease duration (Table S34)**

|  |  | | **Coefficient** | **Std. err.** | **z** | **P>z** | **[95% conf. interval]** | |
| --- | --- | --- | --- | --- | --- | --- | --- | --- |
| ACU VS ACU+MOXI | time | -0.5265371 | | 21.01092 | -0.03 | 0.98 | -41.70718 | 40.65411 |
| ACU VS CT | time | | 4.797968 | 59.22559 | 0.08 | 0.935 | -111.2821 | 120.878 |
| ACU VS MOXI | time | | 0.9984459 | 1.322985 | 0.75 | 0.45 | -1.594556 | 3.591448 |
| ACU VS REHAB | time | | 0.0352007 | 1.004644 | 0.04 | 0.972 | -1.933866 | 2.004268 |
| ACU VS TCM | time | | -0.1560318 | 20.40657 | -0.01 | 0.994 | -40.15218 | 39.84011 |

**Meta-regression analysis of the VAS score group in the primary analysis of LDH: intervention duration(Table S35)**

|  |  | **Coefficient** | **Std. err.** | **z** | **P>z** | **[95% conf. interval]** | |
| --- | --- | --- | --- | --- | --- | --- | --- |
| ACU VS ACU+MOXI | cov1 | 0.0546465 | 0.0487962 | 1.12 | 0.263 | -0.0409924 | 0.1502853 |
| ACU VS CT | cov1 | -0.0683476 | 0.0588656 | -1.16 | 0.246 | -0.1837221 | 0.047027 |
| ACU VS CT+MOXI | cov1 | 0.1358371 | 0.085668 | 1.59 | 0.113 | -0.0320691 | 0.3037432 |
| ACU VS MOXI | cov1 | 0.0126421 | 0.0698533 | 0.18 | 0.856 | -0.1242678 | 0.149552 |
| ACU VS REHAB | cov1 | -0.0552821 | 0.1009724 | -0.55 | 0.584 | -0.2531845 | 0.1426202 |
| ACU VS TCM | cov1 | -0.1609257 | 0.1070913 | -1.5 | 0.133 | -0.3708208 | 0.0489694 |
| ACU VS TCM+MOXI | cov1 | -0.0745652 | 0.0671394 | -1.11 | 0.267 | -0.206156 | 0.0570256 |
| ACU VS TUINA | cov1 | 0.0647776 | 0.187111 | 0.35 | 0.729 | -0.3019533 | 0.4315085 |
| ACU VS TUINA+MOXI | cov1 | 0.1252334 | 0.0968056 | 1.29 | 0.196 | -0.064502 | 0.3149689 |

**Meta-regression analysis of the VAS score group in the primary analysis of LDH: age(Table S36)**

|  |  | **Coefficient** | **Std. err.** | **z** | **P>z** | **[95% conf. interval]** | |
| --- | --- | --- | --- | --- | --- | --- | --- |
| ACU VS ACU+MOXI | age | -0.0450864 | 0.0580507 | -0.78 | 0.437 | -0.1588636 | 0.0686908 |
| ACU VS CT | age | 0.2280977 | 0.1622306 | 1.41 | 0.16 | -0.0898684 | 0.5460639 |
| ACU VS CT+MOXI | age | -0.029478 | 0.1805444 | -0.16 | 0.87 | -0.3833386 | 0.3243825 |
| ACU VS MOXI | age | 0.1639931 | 0.1754707 | 0.93 | 0.35 | -0.1799232 | 0.5079094 |
| ACU VS REHAB | age | 0.3425427 | 0.23087 | 1.48 | 0.138 | -0.1099542 | 0.7950396 |
| ACU VS TCM | age | -0.9916204 | 1.419946 | -0.7 | 0.485 | -3.774664 | 1.791423 |
| ACU VS TCM+MOXI | age | 0.217093 | 0.1499413 | 1.45 | 0.148 | -0.0767866 | 0.5109726 |
| ACU VS TUINA | age | 0.343702 | 0.2664245 | 1.29 | 0.197 | -0.1784803 | 0.8658844 |
| ACU VS TUINA+MOXI | age | 0.3265596 | 0.2005804 | 1.63 | 0.104 | -0.0665708 | 0.71969 |

**Meta-regression analysis of the VAS score group in the primary analysis of LDH: disease duration (Table S37)**

|  |  | **Coefficient** | **Std. err.** | **z** | **P>z** | **[95% conf. interval]** | |
| --- | --- | --- | --- | --- | --- | --- | --- |
| ACU VS ACU+MOXI | time | -0.1005139 | 0.1265298 | -0.79 | 0.427 | -0.3485077 | 0.1474799 |
| ACU VS CT | time | 0.3763903 | 0.5470307 | 0.69 | 0.491 | -0.6957702 | 1.448551 |
| ACU VS CT+MOXI | time | 1.003637 | 0.5701375 | 1.76 | 0.078 | -0.113812 | 2.121086 |
| ACU VS MOXI | time | 0.0721129 | 0.6601914 | 0.11 | 0.913 | -1.221839 | 1.366064 |
| ACU VS REHAB | time | 0.9417469 | 0.6918863 | 1.36 | 0.173 | -0.4143254 | 2.297819 |
| ACU VS TCM | time | 1.223062 | 0.8674095 | 1.41 | 0.159 | -0.4770297 | 2.923153 |
| ACU VS TCM+MOXI | time | 0.6184316 | 0.6533767 | 0.95 | 0.344 | -0.6621633 | 1.899026 |
| ACU VS TUINA | time | 0.6184316 | 0.6533767 | 0.95 | 0.344 | -0.6621633 | 1.899026 |
| ACU VS TUINA+MOXI | time | -0.06191 | 0.5742799 | -0.11 | 0.914 | -1.187478 | 1.063658 |

**Meta-regression analysis of the JOA score group in the primary analysis of LDH: intervention duration (Table S38)**

|  | |  | **Coefficient** | **Std. err.** | **z** | **P>z** | **[95% conf. interval]** | |
| --- | --- | --- | --- | --- | --- | --- | --- | --- |
| ACU VS ACU+MOXI | cov1 | | -0.0662639 | 0.0762213 | -0.87 | 0.385 | -0.2156549 | 0.0831271 |
| ACU VS CT | cov1 | | -0.1799853 | 0.3599591 | -0.5 | 0.617 | -0.8854921 | 0.5255215 |
| ACU VS MOXI | cov1 | | -0.344123 | 0.366453 | -0.94 | 0.348 | -1.062358 | 0.3741118 |
| ACU VS REHAB | cov1 | | -0.0150519 | 3.490066 | 0 | 0.997 | -6.855456 | 6.825352 |
| ACU VS TCM | cov1 | | -0.1811417 | 0.3044349 | -0.6 | 0.552 | -0.7778232 | 0.4155398 |
| ACU VSTCM+MOXI | cov1 | | -0.1622769 | 0.2681292 | -0.61 | 0.545 | -0.6878005 | 0.3632466 |
| ACU VS TUINA | cov1 | | -0.2337082 | 3.488121 | -0.07 | 0.947 | -7.070299 | 6.602883 |
| ACU VS TUINA+MOXI | cov1 | | -0.4143459 | 3.482878 | -0.12 | 0.905 | -7.240662 | 6.41197 |

**Meta-regression analysis of the JOA score group in the primary analysis of LDH: age(Table S39)**

|  |  | **Coefficient** | **Std. err.** | **z** | **P>z** | **[95% conf. interval]** | |
| --- | --- | --- | --- | --- | --- | --- | --- |
| ACU VS ACU+MOXI | age | -0.0896821 | 0.3179754 | -0.28 | 0.778 | -0.7129025 | 0.5335383 |
| ACU VS CT | age | -0.5181641 | 0.9952473 | -0.52 | 0.603 | -2.468813 | 1.432485 |
| ACU VS MOXI | age | -0.5513415 | 0.9963659 | -0.55 | 0.58 | -2.504183 | 1.4015 |
| ACU VS REHAB | age | 0.2215958 | 3.039259 | 0.07 | 0.942 | -5.735242 | 6.178434 |
| ACU VS TCM | age | -0.5256245 | 0.9818702 | -0.54 | 0.592 | -2.450055 | 1.398806 |
| ACU VS TCM+MOXI | age | -0.4644844 | 0.9521293 | -0.49 | 0.626 | -2.330624 | 1.401655 |
| ACU VS TUINA | age | 0.3397901 | 3.041408 | 0.11 | 0.911 | -5.62126 | 6.30084 |
| ACU VS TUINA+MOXI | age | 0.5297275 | 3.030683 | 0.17 | 0.861 | -5.410301 | 6.469756 |

**Meta-regression analysis of the JOA score group in the primary analysis of LDH: disease duration (Table S40)**

|  |  | **Coefficient** | **Std. err.** | **z** | **P>z** | **[95% conf. interval]** | |
| --- | --- | --- | --- | --- | --- | --- | --- |
| ACU VS ACU+MOXI | time | -2.083717 | 2.950062 | -0.71 | 0.48 | -7.865733 | 3.698299 |
| ACU VS CT | time | -0.791408 | 5.600456 | -0.14 | 0.888 | -11.7681 | 10.18528 |
| ACU VS MOXI | time | 0.6909819 | 5.795713 | 0.12 | 0.905 | -10.66841 | 12.05037 |
| ACU VS TCM | time | -2.397229 | 5.76227 | -0.42 | 0.677 | -13.69107 | 8.896613 |
| ACU VS TCM+MOXI | time | -1.756718 | 5.644457 | -0.31 | 0.756 | -12.81965 | 9.306215 |
| ACU VS TUINA | time | -6.157295 | 66.41223 | -0.09 | 0.926 | -136.3229 | 124.0083 |
| ACU VS TUINA+MOXI | time | -5.079724 | 66.36926 | -0.08 | 0.939 | -135.1611 | 125.0016 |

Meta-regression in the Subgroup analysis

**Meta-regression analysis based on the ODI score group in the subgroup analysis of LDH: intervention duration(Table S41)**

|  |  | **Coefficient** | **Std. err.** | **z** | **P>z** | **[95% conf. interval]** | |
| --- | --- | --- | --- | --- | --- | --- | --- |
| ACU VS CM | cov1 | 0.2084069 | 0.2953723 | 0.71 | 0.48 | -0.3705121 | 0.7873259 |
| ACU VS CT | cov1 | -0.1243358 | 0.3305404 | -0.38 | 0.707 | -0.772183 | 0.5235114 |
| ACU VS HM | cov1 | 0.4702693 | 0.3814926 | 1.23 | 0.218 | -0.2774425 | 1.217981 |
| ACU VS WN | cov1 | 0.5763927 | 1.392655 | 0.41 | 0.679 | -2.153161 | 3.305946 |

**Meta-regression analysis based on the ODI score group in the subgroup analysis of LDH: age(Table S42)**

|  |  | **Coefficient** | **Std. err.** | **z** | **P>z** | **[95% conf. interval]** | |
| --- | --- | --- | --- | --- | --- | --- | --- |
| ACU VS CM | age | 0.2385708 | 0.4381552 | 0.54 | 0.586 | -0.6201976 | 1.097339 |
| ACU VS CT | age | 0.2557004 | 0.4778175 | 0.54 | 0.593 | -0.6808047 | 1.192206 |
| ACU VS HM | age | 0.531352 | 0.5232058 | 1.02 | 0.31 | -0.4941125 | 1.556816 |
| ACU VS TCM | age | -0.1424492 | 10.87087 | -0.01 | 0.99 | -21.44897 | 21.16407 |
| ACU VS WN | age | 1.415006 | 48.87974 | 0.03 | 0.977 | -94.38753 | 97.21754 |

**Meta-regression analysis based on the ODI score group in the subgroup analysis of LDH:disease duration (Table S43)**

|  |  | **Coefficient** | **Std. err.** | **z** | **P>z** | **[95% conf. interval]** | |
| --- | --- | --- | --- | --- | --- | --- | --- |
| ACU VS CM | time | 0.3571345 | 4.086957 | 0.09 | 0.93 | -7.653153 | 8.367422 |
| ACU VS CT | time | 0.1313781 | 4.087342 | 0.03 | 0.974 | -7.879665 | 8.142421 |
| ACU VS HM | time | 0.1526453 | 4.095488 | 0.04 | 0.97 | -7.874364 | 8.179655 |
| ACU VS TCM | time | -0.914812 | 32.9746 | -0.03 | 0.978 | -65.54385 | 63.71422 |
| ACU VS WN | time | -0.9433334 | 32.51585 | -0.03 | 0.977 | -64.67322 | 62.78656 |

**Meta-regression analysis based on the VAS score group in the subgroup analysis of LDH: intervention duration (Table S44)**

|  |  | **Coefficient** | **Std. err.** | **z** | **P>z** | **[95% conf. interval]** | |
| --- | --- | --- | --- | --- | --- | --- | --- |
| ACU VS CM | cov1 | -0.0310709 | 0.0519984 | -0.6 | 0.55 | -0.1329858 | 0.0708441 |
| ACU VS CT | cov1 | -0.1144698 | 0.0623812 | -1.84 | 0.067 | -0.2367346 | 0.0077951 |
| ACU VS HM | cov1 | -0.0721851 | 0.0750442 | -0.96 | 0.336 | -0.2192691 | 0.0748989 |
| ACU VS REHAB | cov1 | -0.1892402 | 0.0824625 | -2.29 | 0.062 | -0.3508637 | 0.0276166 |
| ACU VS TCM | cov1 | -0.1107414 | 0.1245846 | -0.89 | 0.374 | -0.3549228 | 0.13344 |
| ACU VS TF | cov1 | -0.1030042 | 0.0797509 | -1.29 | 0.197 | -0.2593131 | 0.0533046 |
| ACU VS TUINA | cov1 | 0.0327722 | 0.2071943 | 0.16 | 0.874 | -0.3733211 | 0.4388655 |
| ACU VS WN | cov1 | -0.068677 | 0.0660143 | -1.04 | 0.298 | -0.1980627 | 0.0607086 |

**Meta-regression analysis based on the VAS score group in the subgroup analysis of LDH: age(Table S45)**

|  |  | **Coefficient** | **Std. err.** | **z** | **P>z** | **[95% conf. interval]** | |
| --- | --- | --- | --- | --- | --- | --- | --- |
| ACU VS CM | age | -0.0146964 | 0.0567542 | -0.26 | 0.796 | -0.1259326 | 0.0965399 |
| ACU VS CT | age | 0.069715 | 0.0694172 | 1 | 0.315 | -0.0663401 | 0.2057701 |
| ACU VS HM | age | 0.1111934 | 0.1045972 | 1.06 | 0.288 | -0.0938134 | 0.3162002 |
| ACU VS REHAB | age | 0.0845209 | 0.138421 | 0.61 | 0.541 | -0.1867793 | 0.3558211 |
| ACU VS TCM | age | -0.2785111 | 1.54818 | -0.18 | 0.857 | -3.312887 | 2.755865 |
| ACU VS TF | age | -0.0127283 | 0.0966486 | -0.13 | 0.895 | -0.2021562 | 0.1766995 |
| ACU VS TUINA | age | -0.0360107 | 0.1885999 | -0.19 | 0.849 | -0.4056596 | 0.3336383 |
| ACU VS WN | age | 0.021354 | 0.0711981 | 0.3 | 0.764 | -0.1181918 | 0.1608997 |

**Meta-regression analysis based on the VAS score group in the subgroup analysis of LDH: disease duration (Table S46)**

|  |  | **Coefficient** | **Std. err.** | **z** | **P>z** | **[95% conf. interval]** | |
| --- | --- | --- | --- | --- | --- | --- | --- |
| ACU VS CM | time | 0.0067728 | 0.1035293 | 0.07 | 0.948 | -0.1961409 | 0.2096865 |
| ACU VS CT | time | -0.0611352 | 0.1091361 | -0.56 | 0.575 | -0.2750381 | 0.1527677 |
| ACU VS HM | time | -0.1132319 | 0.09637 | -1.17 | 0.24 | -0.3021137 | 0.0756498 |
| ACU VS REHAB | time | -0.779348 | 0.7093824 | -1.1 | 0.272 | -2.169712 | 0.6110159 |
| ACU VS TCM | time | -1.358001 | 0.9218923 | -1.47 | 0.141 | -3.164877 | 0.4488747 |
| ACU VS TF | time | 0.1361444 | 0.3186177 | 0.43 | 0.669 | -0.4883349 | 0.7606237 |
| ACU VS WN | time | -0.2369845 | 0.1620545 | -1.46 | 0.144 | -0.5546055 | 0.0806365 |

**Meta-regression analysis based on the JOA score group in the subgroup analysis of LDH: intervention duration (Table S47)**

|  | |  | **Coefficient** | **Std. err.** | **z** | **P>z** | **[95% conf. interval]** | |
| --- | --- | --- | --- | --- | --- | --- | --- | --- |
| ACU VS CM | cov1 | | -0.0532564 | 0.1286365 | -0.41 | 0.679 | -0.3053793 | 0.1988665 |
| ACU VS CT | cov1 | | 0.1415165 | 0.2928114 | 0.48 | 0.629 | -0.4323832 | 0.7154162 |
| ACU VS HM | cov1 | | -0.0894089 | 0.3561085 | -0.25 | 0.802 | -0.7873687 | 0.608551 |
| ACU VS REHAB | cov1 | | 0.2549889 | 0.5056559 | 0.5 | 0.614 | -0.7360785 | 1.246056 |
| ACU VS TCM | cov1 | | -0.1837855 | 0.3685198 | -0.5 | 0.618 | -0.9060711 | 0.5385 |
| ACU VS TF | cov1 | | 0.1726811 | 0.4073193 | 0.42 | 0.672 | -0.6256502 | 0.9710123 |
| ACU VS TUINA | cov1 | | 0.1269534 | 0.4084711 | 0.31 | 0.756 | -0.6736353 | 0.9275421 |
| ACU VS WN | cov1 | | -0.1904776 | 0.2916259 | -0.65 | 0.514 | -0.7620539 | 0.3810987 |

**Meta-regression analysis based on the JOA score group in the subgroup analysis of LDH: age(Table S48)**

|  | |  | **Coefficient** | **Std. err.** | **z** | **P>z** | **[95% conf. interval]** | |
| --- | --- | --- | --- | --- | --- | --- | --- | --- |
| ACU VS CM | age | | -0.1756717 | 0.2575481 | -0.68 | 0.495 | -0.6804566 | 0.3291133 |
| ACU VS CT | age | | -0.1633719 | 0.3336182 | -0.49 | 0.624 | -0.8172515 | 0.4905077 |
| ACU VS HM | age | | -0.3299162 | 0.4297977 | -0.77 | 0.443 | -1.172304 | 0.5124718 |
| ACU VS REHAB | age | | -0.3361781 | 0.4388177 | -0.77 | 0.444 | -1.196245 | 0.5238888 |
| ACU VS TCM | age | | -0.3015524 | 0.4529735 | -0.67 | 0.506 | -1.189364 | 0.5862593 |
| ACU VS TF | age | | 0.2754846 | 0.3634522 | 0.76 | 0.448 | -0.4368686 | 0.9878379 |
| ACU VS TUINA | age | | -0.365298 | 0.3921076 | -0.93 | 0.352 | -1.133815 | 0.4032187 |
| ACU VS WN | age | | -0.0076447 | 0.3417646 | -0.02 | 0.982 | -0.6774911 | 0.6622017 |

**Meta-regression analysis based on the JOA score group in the subgroup analysis of LDH: disease duration (Table S49)**

|  |  | **Coefficient** | **Std. err.** | **z** | **P>z** | **[95% conf. interval]** | |
| --- | --- | --- | --- | --- | --- | --- | --- |
| ACU VS CM | time | -2.739377 | 3.046755 | -0.9 | 0.369 | -8.710906 | 3.232153 |
| ACU VS CT | time | -0.734631 | 5.665253 | -0.13 | 0.897 | -11.83832 | 10.36906 |
| ACU VS HM | time | -4.116101 | 5.876231 | -0.7 | 0.484 | -15.6333 | 7.401101 |
| ACU VS TCM | time | -5.221475 | 6.017825 | -0.87 | 0.386 | -17.0162 | 6.573245 |
| ACU VS TF | time | 0.4475175 | 5.667599 | 0.08 | 0.937 | -10.66077 | 11.55581 |
| ACU VS TUINA | time | -3.827077 | 4.642759 | -0.82 | 0.41 | -12.92672 | 5.272563 |
| ACU VS WN | time | 1.19728 | 5.885483 | 0.2 | 0.839 | -10.33806 | 12.73262 |

Sensitivity Analysis (Primary analysis)

**Sensitivity analysis based on the ODI score group in the primary analysis of patients with LDH. (Table S50)**

| **dropped_id** | **comparison** | **eff** | **lci** | **uci** | **connected** |
| --- | --- | --- | --- | --- | --- |
| Wang Liya 2024 | CT VS ACU | -4.7378531 | -7.39781 | -2.077896 | 1 |
| Wang Liya 2024 | CT VS ACU+MOXI | -10.107906 | -12.18363 | -8.032186 | 1 |
| Wang Liya 2024 | CT VS CT+MOXI | -0.95280548 | -5.124144 | 3.218533 | 1 |
| Wang Liya 2024 | CT VS MOXI | -3.1643115 | -4.865468 | -1.463155 | 1 |
| Wang Liya 2024 | CT VS REHAB | -1.5627466 | -5.570918 | 2.445425 | 1 |
| Wang Liya 2024 | CT VS TCM | 1.9353434 | -0.2221109 | 4.092798 | 1 |
| Wang Liya 2024 | CT VS TCM+MOXI | -4.6666403 | -5.811548 | -3.521732 | 1 |
| Wang Liya 2024 | CT VS TUINA+MOXI | -6.4429683 | -9.280127 | -3.60581 | 1 |
| Hu Qinqin 2024 | CT VS ACU | -4.745936 | -7.201064 | -2.290808 | 1 |
| Hu Qinqin 2024 | CT VS ACU+MOXI | -10.10826 | -11.99955 | -8.216969 | 1 |
| Hu Qinqin 2024 | CT VS CT+MOXI | -1.0804462 | -2.904017 | 0.7431245 | 1 |
| Hu Qinqin 2024 | CT VS MOXI | -3.0768631 | -4.571263 | -1.582463 | 1 |
| Hu Qinqin 2024 | CT VS REHAB | -1.5484201 | -4.984735 | 1.887894 | 1 |
| Hu Qinqin 2024 | CT VS TCM | 2.1294531 | 0.0290606 | 4.229846 | 1 |
| Hu Qinqin 2024 | CT VS TCM+MOXI | -4.4498575 | -5.624376 | -3.275339 | 1 |
| Hu Qinqin 2024 | CT VS TUINA+MOXI | -6.4286206 | -8.555551 | -4.301691 | 1 |
| Xu Fen 2025 | CT VS ACU | -4.7357331 | -7.442704 | -2.028762 | 1 |
| Xu Fen 2025 | CT VS ACU+MOXI | -10.107819 | -12.22583 | -7.989809 | 1 |
| Xu Fen 2025 | CT VS CT+MOXI | -1.1260582 | -3.156945 | 0.9048279 | 1 |
| Xu Fen 2025 | CT VS MOXI | -3.2683152 | -5.435705 | -1.100925 | 1 |
| Xu Fen 2025 | CT VS REHAB | -1.6915437 | -5.516261 | 2.133174 | 1 |
| Xu Fen 2025 | CT VS TCM | 1.9152685 | -0.2968689 | 4.127406 | 1 |
| Xu Fen 2025 | CT VS TCM+MOXI | -4.6914788 | -5.89988 | -3.483078 | 1 |
| Xu Fen 2025 | CT VS TUINA+MOXI | -6.5717777 | -9.108486 | -4.035069 | 1 |
| Liu Wenlin 2024 | CT VS ACU | -5.0267602 | -7.521332 | -2.532188 | 1 |
| Liu Wenlin 2024 | CT VS ACU+MOXI | -10.108379 | -11.96782 | -8.248936 | 1 |
| Liu Wenlin 2024 | CT VS CT+MOXI | -1.1097849 | -2.907706 | 0.6881367 | 1 |
| Liu Wenlin 2024 | CT VS MOXI | -3.1955572 | -4.649319 | -1.741795 | 1 |
| Liu Wenlin 2024 | CT VS REHAB | -1.6364242 | -5.034525 | 1.761677 | 1 |
| Liu Wenlin 2024 | CT VS TCM | 1.892657 | -0.111667 | 3.896981 | 1 |
| Liu Wenlin 2024 | CT VS TCM+MOXI | -4.6824214 | -5.729175 | -3.635668 | 1 |
| Liu Wenlin 2024 | CT VS TUINA+MOXI | -6.5166258 | -8.609994 | -4.423258 | 1 |
| Yao Dong 2024 | CT VS ACU | -4.7378472 | -7.397851 | -2.077843 | 1 |
| Yao Dong 2024 | CT VS ACU+MOXI | -10.107902 | -12.18367 | -8.03214 | 1 |
| Yao Dong 2024 | CT VS CT+MOXI | -1.1499998 | -3.375961 | 1.075961 | 1 |
| Yao Dong 2024 | CT VS MOXI | -3.1644261 | -4.865633 | -1.463219 | 1 |
| Yao Dong 2024 | CT VS REHAB | -1.56307 | -5.571382 | 2.445242 | 1 |
| Yao Dong 2024 | CT VS TCM | 1.9353204 | -0.2221611 | 4.092802 | 1 |
| Yao Dong 2024 | CT VS TCM+MOXI | -4.6666678 | -5.811594 | -3.521742 | 1 |
| Yao Dong 2024 | CT VS TUINA+MOXI | -6.4432919 | -9.280618 | -3.605966 | 1 |
| Deng Min 2022 | CT VS ACU | -4.7355204 | -7.447111 | -2.02393 | 1 |
| Deng Min 2022 | CT VS ACU+MOXI | -10.10781 | -12.22997 | -7.985651 | 1 |
| Deng Min 2022 | CT VS CT+MOXI | -1.1085925 | -3.110325 | 0.8931403 | 1 |
| Deng Min 2022 | CT VS MOXI | -3.2003761 | -4.849659 | -1.551093 | 1 |
| Deng Min 2022 | CT VS REHAB | -1.6417716 | -5.328868 | 2.045325 | 1 |
| Deng Min 2022 | CT VS TCM | 1.9123813 | -0.3722442 | 4.197007 | 1 |
| Deng Min 2022 | CT VS TCM+MOXI | -4.6948251 | -6.025697 | -3.363954 | 1 |
| Deng Min 2022 | CT VS TUINA+MOXI | -6.5220033 | -8.842216 | -4.20179 | 1 |
| Guo Hui 2023 | CT VS ACU | -4.7536663 | -6.516277 | -2.991055 | 1 |
| Guo Hui 2023 | CT VS ACU+MOXI | -10.109228 | -11.36912 | -8.849339 | 1 |
| Guo Hui 2023 | CT VS CT+MOXI | -1.1225774 | -2.479045 | 0.2338904 | 1 |
| Guo Hui 2023 | CT VS MOXI | -3.221986 | -4.323393 | -2.120579 | 1 |
| Guo Hui 2023 | CT VS REHAB | -1.6511057 | -4.470412 | 1.168201 | 1 |
| Guo Hui 2023 | CT VS TCM | 0.6210676 | -1.317778 | 2.559914 | 1 |
| Guo Hui 2023 | CT VS TCM+MOXI | -4.7189984 | -5.540538 | -3.897459 | 1 |
| Guo Hui 2023 | CT VS TUINA+MOXI | -6.5312545 | -8.160377 | -4.902132 | 1 |
| Zhang Jing 2026 | CT VS ACU | -4.7536664 | -6.516284 | -2.991049 | 1 |
| Zhang Jing 2026 | CT VS ACU+MOXI | -10.109228 | -11.36912 | -8.849333 | 1 |
| Zhang Jing 2026 | CT VS CT+MOXI | -1.1225809 | -2.479053 | 0.2338907 | 1 |
| Zhang Jing 2026 | CT VS MOXI | -3.2220038 | -4.323413 | -2.120595 | 1 |
| Zhang Jing 2026 | CT VS REHAB | -1.6511198 | -4.470431 | 1.168192 | 1 |
| Zhang Jing 2026 | CT VS TCM | 3.5109886 | 1.177718 | 5.844259 | 1 |
| Zhang Jing 2026 | CT VS TCM+MOXI | -4.7190294 | -5.540567 | -3.897492 | 1 |
| Zhang Jing 2026 | CT VS TUINA+MOXI | -6.5312686 | -8.160395 | -4.902142 | 1 |
| Yin Shaojian 2022 | CT VS ACU | -4.7348081 | -7.461721 | -2.007895 | 1 |
| Yin Shaojian 2022 | CT VS ACU+MOXI | -10.107782 | -12.24372 | -7.97184 | 1 |
| Yin Shaojian 2022 | CT VS CT+MOXI | -1.0953838 | -3.137573 | 0.9468055 | 1 |
| Yin Shaojian 2022 | CT VS MOXI | -3.1495038 | -5.288107 | -1.010901 | 1 |
| Yin Shaojian 2022 | CT VS REHAB | -1.6046032 | -5.436806 | 2.2276 | 1 |
| Yin Shaojian 2022 | CT VS TCM | 1.9228049 | -0.3229219 | 4.168532 | 1 |
| Yin Shaojian 2022 | CT VS TCM+MOXI | -4.685915 | -5.939733 | -3.432097 | 1 |
| Yin Shaojian 2022 | CT VS TUINA+MOXI | -6.4848345 | -9.0178 | -3.951869 | 1 |
| Jiang Yunqing 2023 | CT VS ACU | -4.7494632 | -6.338055 | -3.160872 | 1 |
| Jiang Yunqing 2023 | CT VS ACU+MOXI | -10.109415 | -11.20639 | -9.012438 | 1 |
| Jiang Yunqing 2023 | CT VS CT+MOXI | -1.168564 | -2.412025 | 0.0748974 | 1 |
| Jiang Yunqing 2023 | CT VS MOXI | -3.4625761 | -4.488279 | -2.436873 | 1 |
| Jiang Yunqing 2023 | CT VS REHAB | -1.8412474 | -4.528096 | 0.8456009 | 1 |
| Jiang Yunqing 2023 | CT VS TCM | 1.3330231 | -0.210836 | 2.876882 | 1 |
| Jiang Yunqing 2023 | CT VS TCM+MOXI | -5.1083811 | -5.929207 | -4.287555 | 1 |
| Jiang Yunqing 2023 | CT VS TUINA+MOXI | -6.7213916 | -8.240826 | -5.201957 | 1 |
| Wang Jianying 2025 | _ | _ | _ | _ | disconnected |
| Gao Yuanguanyu 2021 | CT VS ACU | -4.7483791 | -7.128134 | -2.368624 | 1 |
| Gao Yuanguanyu 2021 | CT VS ACU+MOXI | -10.108383 | -11.93152 | -8.28525 | 1 |
| Gao Yuanguanyu 2021 | CT VS CT+MOXI | -1.0719041 | -2.843906 | 0.700098 | 1 |
| Gao Yuanguanyu 2021 | CT VS MOXI | -3.0364231 | -4.500114 | -1.572732 | 1 |
| Gao Yuanguanyu 2021 | CT VS REHAB | -1.5172555 | -4.885635 | 1.851124 | 1 |
| Gao Yuanguanyu 2021 | CT VS TCM | 2.197302 | 0.1281246 | 4.266479 | 1 |
| Gao Yuanguanyu 2021 | CT VS TCM+MOXI | -4.3728007 | -5.565222 | -3.180379 | 1 |
| Gao Yuanguanyu 2021 | CT VS TUINA+MOXI | -6.3974475 | -8.474661 | -4.320234 | 1 |
| Xu Mengxia 2019 | CT VS ACU | -4.909548 | -8.104357 | -1.714739 | 1 |
| Xu Mengxia 2019 | CT VS ACU+MOXI | -10.10964 | -12.13185 | -8.087426 | 1 |
| Xu Mengxia 2019 | CT VS CT+MOXI | -1.1071067 | -3.029586 | 0.8153728 | 1 |
| Xu Mengxia 2019 | CT VS MOXI | -3.1911106 | -4.74492 | -1.637301 | 1 |
| Xu Mengxia 2019 | CT VS REHAB | -1.6343223 | -5.204798 | 1.936153 | 1 |
| Xu Mengxia 2019 | CT VS TCM | 1.9204497 | -0.1949697 | 4.035869 | 1 |
| Xu Mengxia 2019 | CT VS TCM+MOXI | -4.6752577 | -5.787718 | -3.562797 | 1 |
| Xu Mengxia 2019 | CT VS TUINA+MOXI | -6.5145418 | -8.739905 | -4.289178 | 1 |
| Pan Meng 2025 | CT VS ACU | -4.2678434 | -6.996661 | -1.539025 | 1 |
| Pan Meng 2025 | CT VS ACU+MOXI | -10.108466 | -12.03815 | -8.178779 | 1 |
| Pan Meng 2025 | CT VS CT+MOXI | -1.1085902 | -2.960135 | 0.742955 | 1 |
| Pan Meng 2025 | CT VS MOXI | -3.1935379 | -4.690372 | -1.696703 | 1 |
| Pan Meng 2025 | CT VS REHAB | -1.6354566 | -5.107369 | 1.836456 | 1 |
| Pan Meng 2025 | CT VS TCM | 1.9050982 | -0.1471162 | 3.957313 | 1 |
| Pan Meng 2025 | CT VS TCM+MOXI | -4.6792033 | -5.754239 | -3.604167 | 1 |
| Pan Meng 2025 | CT VS TUINA+MOXI | -6.5156657 | -8.665808 | -4.365523 | 1 |
| Li Ju 2024 | CT VS ACU | -4.737842 | -7.397952 | -2.077732 | 1 |
| Li Ju 2024 | CT VS ACU+MOXI | -10.107902 | -12.18376 | -8.032044 | 1 |
| Li Ju 2024 | CT VS CT+MOXI | -1.1494952 | -3.375406 | 1.076415 | 1 |
| Li Ju 2024 | CT VS MOXI | -3.1655162 | -4.866936 | -1.464097 | 1 |
| Li Ju 2024 | CT VS REHAB | -1.7584825 | -6.482794 | 2.965829 | 1 |
| Li Ju 2024 | CT VS TCM | 1.935058 | -0.222461 | 4.092577 | 1 |
| Li Ju 2024 | CT VS TCM+MOXI | -4.6669411 | -5.811906 | -3.521976 | 1 |
| Li Ju 2024 | CT VS TUINA+MOXI | -6.6387153 | -10.42068 | -2.856745 | 1 |
| Zhou Huaming 2024 | CT VS ACU | -4.7485657 | -7.121936 | -2.375195 | 1 |
| Zhou Huaming 2024 | CT VS ACU+MOXI | -10.108393 | -11.92591 | -8.290872 | 1 |
| Zhou Huaming 2024 | CT VS CT+MOXI | -1.1106038 | -2.876683 | 0.6554749 | 1 |
| Zhou Huaming 2024 | CT VS MOXI | -3.1969279 | -4.625139 | -1.768716 | 1 |
| Zhou Huaming 2024 | CT VS REHAB | 1.88488 | -0.0918047 | 3.861565 | 1 |
| Zhou Huaming 2024 | CT VS TCM | -4.6844597 | -5.714525 | -3.654394 | 1 |
| Zhou Huaming 2024 | CT VS TCM+MOXI | -6.51749 | -8.577229 | -4.45775 | 1 |

**Sensitivity analysis based on the VAS score group in the primary analysis of patients with LDH.(Table S51)**

| **dropped_id** | **comparison** | **eff** | **lci** | **uci** | **connected** |
| --- | --- | --- | --- | --- | --- |
| Chunyue Cai 2018 | CT VS ACU | -0.62989373 | -1.76548 | 0.5056927 | 1 |
| Chunyue Cai 2018 | CT VS ACU+MOXI | -1.924973 | -3.069277 | -0.7806687 | 1 |
| Chunyue Cai 2018 | CT VS CT+MOXI | -2.0144799 | -3.16261 | -0.8663501 | 1 |
| Chunyue Cai 2018 | CT VS MOXI | -1.4741623 | -2.418322 | -0.5300032 | 1 |
| Chunyue Cai 2018 | CT VS REHAB | -0.05458796 | -1.479329 | 1.370153 | 1 |
| Chunyue Cai 2018 | CT VS TCM | 0.30893203 | -1.44827 | 2.066134 | 1 |
| Chunyue Cai 2018 | CT VS TCM+MOXI | -1.4971919 | -2.284687 | -0.7096967 | 1 |
| Chunyue Cai 2018 | CT VS TUINA | -0.37552385 | -2.140135 | 1.389088 | 1 |
| Chunyue Cai 2018 | CT VS TUINA+MOXI | -2.1850177 | -3.408336 | -0.9616991 | 1 |
| Xu Minmin 2016 | CT VS ACU | -0.65165306 | -1.718338 | 0.4150323 | 1 |
| Xu Minmin 2016 | CT VS ACU+MOXI | -1.9423141 | -3.043069 | -0.8415589 | 1 |
| Xu Minmin 2016 | CT VS CT+MOXI | -2.0067975 | -3.15362 | -0.8599749 | 1 |
| Xu Minmin 2016 | CT VS MOXI | -1.3945837 | -2.430763 | -0.3584042 | 1 |
| Xu Minmin 2016 | CT VS REHAB | -0.02092253 | -1.449132 | 1.407287 | 1 |
| Xu Minmin 2016 | CT VS TCM | 0.35243539 | -1.396967 | 2.101838 | 1 |
| Xu Minmin 2016 | CT VS TCM+MOXI | -1.4536104 | -2.228579 | -0.6786422 | 1 |
| Xu Minmin 2016 | CT VS TUINA | -0.34402703 | -2.111587 | 1.423533 | 1 |
| Xu Minmin 2016 | CT VS TUINA+MOXI | -2.1534493 | -3.383156 | -0.9237426 | 1 |
| Wang Liya 2024 | CT VS ACU | -0.71627772 | -1.701247 | 0.2686912 | 1 |
| Wang Liya 2024 | CT VS ACU+MOXI | -1.9944405 | -3.017038 | -0.9718426 | 1 |
| Wang Liya 2024 | CT VS CT+MOXI | -2.7080744 | -3.969206 | -1.446943 | 1 |
| Wang Liya 2024 | CT VS MOXI | -1.5260566 | -2.406183 | -0.6459301 | 1 |
| Wang Liya 2024 | CT VS REHAB | -0.21270534 | -1.556267 | 1.130857 | 1 |
| Wang Liya 2024 | CT VS TCM | 0.30686093 | -1.331563 | 1.945284 | 1 |
| Wang Liya 2024 | CT VS TCM+MOXI | -1.4978499 | -2.219561 | -0.7761387 | 1 |
| Wang Liya 2024 | CT VS TUINA | -0.56731464 | -2.229582 | 1.094952 | 1 |
| Wang Liya 2024 | CT VS TUINA+MOXI | -2.3764274 | -3.537218 | -1.215637 | 1 |
| Hu Qinqin 2024 | CT VS ACU | -0.68995684 | -1.751346 | 0.3714323 | 1 |
| Hu Qinqin 2024 | CT VS ACU+MOXI | -1.9727849 | -3.071431 | -0.8741382 | 1 |
| Hu Qinqin 2024 | CT VS CT+MOXI | -2.0139688 | -3.163378 | -0.8645591 | 1 |
| Hu Qinqin 2024 | CT VS MOXI | -1.487099 | -2.431515 | -0.5426826 | 1 |
| Hu Qinqin 2024 | CT VS REHAB | -0.04788558 | -1.478872 | 1.383101 | 1 |
| Hu Qinqin 2024 | CT VS TCM | 0.33496838 | -1.43937 | 2.109306 | 1 |
| Hu Qinqin 2024 | CT VS TCM+MOXI | -1.471147 | -2.293418 | -0.6488761 | 1 |
| Hu Qinqin 2024 | CT VS TUINA | -0.37347275 | -2.141843 | 1.394897 | 1 |
| Hu Qinqin 2024 | CT VS TUINA+MOXI | -2.1829725 | -3.410466 | -0.9554793 | 1 |
| Ding Wenjie 2025 | CT VS ACU | -0.75727325 | -1.796281 | 0.281734 | 1 |
| Ding Wenjie 2025 | CT VS ACU+MOXI | -2.0265789 | -3.102784 | -0.9503735 | 1 |
| Ding Wenjie 2025 | CT VS CT+MOXI | -1.9231102 | -3.061817 | -0.7844036 | 1 |
| Ding Wenjie 2025 | CT VS MOXI | -1.6735295 | -2.652869 | -0.69419 | 1 |
| Ding Wenjie 2025 | CT VS REHAB | 0.25063638 | -1.248085 | 1.749358 | 1 |
| Ding Wenjie 2025 | CT VS TCM | 0.32961209 | -1.38795 | 2.047174 | 1 |
| Ding Wenjie 2025 | CT VS TCM+MOXI | -1.4761088 | -2.232461 | -0.7197565 | 1 |
| Ding Wenjie 2025 | CT VS TUINA | -0.0005882 | -1.857809 | 1.856632 | 1 |
| Ding Wenjie 2025 | CT VS TUINA+MOXI | -1.8092554 | -3.185091 | -0.4334201 | 1 |
| Zhong Qunhua 2024 | CT VS ACU | -0.67121569 | -1.606453 | 0.2640217 | 1 |
| Zhong Qunhua 2024 | CT VS ACU+MOXI | -1.9591563 | -2.929933 | -0.98838 | 1 |
| Zhong Qunhua 2024 | CT VS CT+MOXI | -1.1097531 | -2.30729 | 0.0877832 | 1 |
| Zhong Qunhua 2024 | CT VS MOXI | -1.4427726 | -2.278605 | -0.6069407 | 1 |
| Zhong Qunhua 2024 | CT VS REHAB | 0.15022481 | -1.126681 | 1.42713 | 1 |
| Zhong Qunhua 2024 | CT VS TCM | 0.35778586 | -1.198186 | 1.913757 | 1 |
| Zhong Qunhua 2024 | CT VS TCM+MOXI | -1.4456482 | -2.130974 | -0.7603221 | 1 |
| Zhong Qunhua 2024 | CT VS TUINA | -0.12646905 | -1.704924 | 1.451985 | 1 |
| Zhong Qunhua 2024 | CT VS TUINA+MOXI | -1.934392 | -3.037366 | -0.8314183 | 1 |
| Liu Wenlin 2024 | CT VS ACU | -0.65853451 | -1.709965 | 0.392896 | 1 |
| Liu Wenlin 2024 | CT VS ACU+MOXI | -2.0201668 | -3.116289 | -0.9240444 | 1 |
| Liu Wenlin 2024 | CT VS CT+MOXI | -2.013055 | -3.154855 | -0.8712556 | 1 |
| Liu Wenlin 2024 | CT VS MOXI | -1.4768612 | -2.411618 | -0.5421041 | 1 |
| Liu Wenlin 2024 | CT VS REHAB | -0.04382783 | -1.460497 | 1.372842 | 1 |
| Liu Wenlin 2024 | CT VS TCM | 0.34074299 | -1.399548 | 2.081033 | 1 |
| Liu Wenlin 2024 | CT VS TCM+MOXI | -1.4652357 | -2.231968 | -0.6985032 | 1 |
| Liu Wenlin 2024 | CT VS TUINA | -0.36946166 | -2.124462 | 1.385538 | 1 |
| Liu Wenlin 2024 | CT VS TUINA+MOXI | -2.1788889 | -3.395659 | -0.9621186 | 1 |
| Wu Yusheng 2025 | CT VS ACU | -0.72269027 | -1.779189 | 0.3338088 | 1 |
| Wu Yusheng 2025 | CT VS ACU+MOXI | -1.9988898 | -3.09264 | -0.9051391 | 1 |
| Wu Yusheng 2025 | CT VS CT+MOXI | -2.0191657 | -3.163708 | -0.8746237 | 1 |
| Wu Yusheng 2025 | CT VS MOXI | -1.5090158 | -2.449253 | -0.5687789 | 1 |
| Wu Yusheng 2025 | CT VS REHAB | -0.0783406 | -1.503139 | 1.346458 | 1 |
| Wu Yusheng 2025 | CT VS TCM | 0.26588821 | -1.499857 | 2.031634 | 1 |
| Wu Yusheng 2025 | CT VS TCM+MOXI | -1.5402146 | -2.35655 | -0.7238792 | 1 |
| Wu Yusheng 2025 | CT VS TUINA | -0.39455896 | -2.155409 | 1.366291 | 1 |
| Wu Yusheng 2025 | CT VS TUINA+MOXI | -2.2040529 | -3.426308 | -0.9817982 | 1 |
| Yao Dong 2024 | CT VS ACU | -0.67114055 | -1.721269 | 0.3789882 | 1 |
| Yao Dong 2024 | CT VS ACU+MOXI | -1.9578549 | -3.047371 | -0.8683391 | 1 |
| Yao Dong 2024 | CT VS CT+MOXI | -2.1697605 | -3.439775 | -0.8997459 | 1 |
| Yao Dong 2024 | CT VS MOXI | -1.4528162 | -2.396641 | -0.5089914 | 1 |
| Yao Dong 2024 | CT VS REHAB | 0.10270231 | -1.41617 | 1.621575 | 1 |
| Yao Dong 2024 | CT VS TCM | 0.35466465 | -1.389786 | 2.099115 | 1 |
| Yao Dong 2024 | CT VS TCM+MOXI | -1.4513295 | -2.22281 | -0.679849 | 1 |
| Yao Dong 2024 | CT VS TUINA | -0.1909298 | -2.066515 | 1.684656 | 1 |
| Yao Dong 2024 | CT VS TUINA+MOXI | -2.0000595 | -3.383916 | -0.6162034 | 1 |
| Liang Yuanyuan 2014 | CT VS ACU | -0.76083737 | -1.804219 | 0.2825446 | 1 |
| Liang Yuanyuan 2014 | CT VS ACU+MOXI | -2.0293917 | -3.109354 | -0.9494298 | 1 |
| Liang Yuanyuan 2014 | CT VS CT+MOXI | -2.0252696 | -3.155103 | -0.8954365 | 1 |
| Liang Yuanyuan 2014 | CT VS MOXI | -1.5343921 | -2.462822 | -0.6059622 | 1 |
| Liang Yuanyuan 2014 | CT VS REHAB | -0.11393045 | -1.521078 | 1.293218 | 1 |
| Liang Yuanyuan 2014 | CT VS TCM | 0.18614109 | -1.558081 | 1.930363 | 1 |
| Liang Yuanyuan 2014 | CT VS TCM+MOXI | -1.6197773 | -2.427853 | -0.8117011 | 1 |
| Liang Yuanyuan 2014 | CT VS TUINA | -0.41901203 | -2.157495 | 1.319471 | 1 |
| Liang Yuanyuan 2014 | CT VS TUINA+MOXI | -2.2284157 | -3.435402 | -1.021429 | 1 |
| Deng Min 2022 | CT VS ACU | -0.69785446 | -1.759623 | 0.3639141 | 1 |
| Deng Min 2022 | CT VS ACU+MOXI | -1.9790715 | -3.078013 | -0.8801295 | 1 |
| Deng Min 2022 | CT VS CT+MOXI | -2.0152145 | -3.16479 | -0.8656389 | 1 |
| Deng Min 2022 | CT VS MOXI | -1.4924078 | -2.437072 | -0.5477436 | 1 |
| Deng Min 2022 | CT VS REHAB | -0.05521492 | -1.486544 | 1.376114 | 1 |
| Deng Min 2022 | CT VS TCM | 0.31819538 | -1.457009 | 2.0934 | 1 |
| Deng Min 2022 | CT VS TCM+MOXI | -1.4879406 | -2.311666 | -0.6642151 | 1 |
| Deng Min 2022 | CT VS TUINA | -0.37857405 | -2.147247 | 1.390099 | 1 |
| Deng Min 2022 | CT VS TUINA+MOXI | -2.1880843 | -3.415829 | -0.9603395 | 1 |
| Li Shengqiang 2015 | CT VS ACU | -0.75293322 | -1.790695 | 0.2848282 | 1 |
| Li Shengqiang 2015 | CT VS ACU+MOXI | -1.8922186 | -2.974242 | -0.8101954 | 1 |
| Li Shengqiang 2015 | CT VS CT+MOXI | -2.0166779 | -3.143173 | -0.8901835 | 1 |
| Li Shengqiang 2015 | CT VS MOXI | -1.5087844 | -2.431153 | -0.5864156 | 1 |
| Li Shengqiang 2015 | CT VS REHAB | -0.0605534 | -1.45869 | 1.337583 | 1 |
| Li Shengqiang 2015 | CT VS TCM | 0.3165814 | -1.400576 | 2.033739 | 1 |
| Li Shengqiang 2015 | CT VS TCM+MOXI | -1.4891453 | -2.245699 | -0.7325918 | 1 |
| Li Shengqiang 2015 | CT VS TUINA | -0.38371521 | -2.115383 | 1.347952 | 1 |
| Li Shengqiang 2015 | CT VS TUINA+MOXI | -2.1930301 | -3.393823 | -0.9922373 | 1 |
| Wen Qi 2023 | CT VS ACU | -0.75304222 | -1.870311 | 0.3642262 | 1 |
| Wen Qi 2023 | CT VS ACU+MOXI | -2.0230323 | -3.155412 | -0.8906529 | 1 |
| Wen Qi 2023 | CT VS CT+MOXI | -2.0116925 | -3.158775 | -0.86461 | 1 |
| Wen Qi 2023 | CT VS MOXI | -1.4452997 | -2.419854 | -0.4707454 | 1 |
| Wen Qi 2023 | CT VS REHAB | -0.04235481 | -1.465799 | 1.381089 | 1 |
| Wen Qi 2023 | CT VS TCM | 0.32483452 | -1.423745 | 2.073415 | 1 |
| Wen Qi 2023 | CT VS TCM+MOXI | -1.4812525 | -2.252454 | -0.7100507 | 1 |
| Wen Qi 2023 | CT VS TUINA | -0.36407879 | -2.128038 | 1.39988 | 1 |
| Wen Qi 2023 | CT VS TUINA+MOXI | -2.1735423 | -3.397252 | -0.9498329 | 1 |
| Lin Mingqin 2021 | CT VS ACU | -0.71040285 | -1.778967 | 0.3581609 | 1 |
| Lin Mingqin 2021 | CT VS ACU+MOXI | -1.9890707 | -3.091814 | -0.8863274 | 1 |
| Lin Mingqin 2021 | CT VS CT+MOXI | -2.0177609 | -3.166725 | -0.8687969 | 1 |
| Lin Mingqin 2021 | CT VS MOXI | -1.5334686 | -2.570969 | -0.4959678 | 1 |
| Lin Mingqin 2021 | CT VS REHAB | -0.06269285 | -1.493477 | 1.368091 | 1 |
| Lin Mingqin 2021 | CT VS TCM | 0.32297238 | -1.429661 | 2.075606 | 1 |
| Lin Mingqin 2021 | CT VS TCM+MOXI | -1.4831421 | -2.259514 | -0.7067701 | 1 |
| Lin Mingqin 2021 | CT VS TUINA | -0.3889571 | -2.15976 | 1.381846 | 1 |
| Lin Mingqin 2021 | CT VS TUINA+MOXI | -2.1984774 | -3.430385 | -0.9665694 | 1 |
| Xie Wenhui 2023 | CT VS ACU | -1.0250427 | -2.132989 | 0.0829038 | 1 |
| Xie Wenhui 2023 | CT VS ACU+MOXI | -2.4252129 | -3.638233 | -1.212193 | 1 |
| Xie Wenhui 2023 | CT VS CT+MOXI | -2.0267679 | -3.13555 | -0.9179857 | 1 |
| Xie Wenhui 2023 | CT VS MOXI | -1.6006705 | -2.520047 | -0.6812934 | 1 |
| Xie Wenhui 2023 | CT VS REHAB | -0.10730747 | -1.48575 | 1.271135 | 1 |
| Xie Wenhui 2023 | CT VS TCM | 0.24746067 | -1.446226 | 1.941147 | 1 |
| Xie Wenhui 2023 | CT VS TCM+MOXI | -1.5579981 | -2.310529 | -0.8054668 | 1 |
| Xie Wenhui 2023 | CT VS TUINA | -0.42436939 | -2.130048 | 1.281309 | 1 |
| Xie Wenhui 2023 | CT VS TUINA+MOXI | -2.2335883 | -3.417484 | -1.049692 | 1 |
| Zhang Jing 2026 | CT VS ACU | -0.6917177 | -1.734843 | 0.3514081 | 1 |
| Zhang Jing 2026 | CT VS ACU+MOXI | -1.9742827 | -3.057562 | -0.8910035 | 1 |
| Zhang Jing 2026 | CT VS CT+MOXI | -2.0143159 | -3.152818 | -0.875814 | 1 |
| Zhang Jing 2026 | CT VS MOXI | -1.4881194 | -2.419533 | -0.5567057 | 1 |
| Zhang Jing 2026 | CT VS REHAB | -0.04967839 | -1.462263 | 1.362906 | 1 |
| Zhang Jing 2026 | CT VS TCM | 0.94630786 | -1.402966 | 3.295582 | 1 |
| Zhang Jing 2026 | CT VS TCM+MOXI | -1.4738506 | -2.23806 | -0.7096413 | 1 |
| Zhang Jing 2026 | CT VS TUINA | -0.37449886 | -2.124416 | 1.375418 | 1 |
| Zhang Jing 2026 | CT VS TUINA+MOXI | -2.1839057 | -3.397146 | -0.9706658 | 1 |
| Zhu Bin 2021 | CT VS ACU | -0.90352544 | -1.996945 | 0.1898938 | 1 |
| Zhu Bin 2021 | CT VS ACU+MOXI | -2.1430267 | -3.252305 | -1.033749 | 1 |
| Zhu Bin 2021 | CT VS CT+MOXI | -2.0059532 | -3.131462 | -0.8804445 | 1 |
| Zhu Bin 2021 | CT VS MOXI | -1.3418655 | -2.29666 | -0.3870705 | 1 |
| Zhu Bin 2021 | CT VS REHAB | -0.02733028 | -1.424615 | 1.369954 | 1 |
| Zhu Bin 2021 | CT VS TCM | 0.30538028 | -1.410542 | 2.021303 | 1 |
| Zhu Bin 2021 | CT VS TCM+MOXI | -1.5003379 | -2.257066 | -0.7436103 | 1 |
| Zhu Bin 2021 | CT VS TUINA | -0.33975518 | -2.070761 | 1.391251 | 1 |
| Zhu Bin 2021 | CT VS TUINA+MOXI | -2.1489858 | -3.350072 | -0.9479001 | 1 |
| Yu Ansheng 2017 | CT VS ACU | -0.70410273 | -1.760915 | 0.3527099 | 1 |
| Yu Ansheng 2017 | CT VS ACU+MOXI | -1.9564241 | -3.058218 | -0.8546298 | 1 |
| Yu Ansheng 2017 | CT VS CT+MOXI | -2.0146321 | -3.162295 | -0.8669696 | 1 |
| Yu Ansheng 2017 | CT VS MOXI | -1.4922396 | -2.43176 | -0.552719 | 1 |
| Yu Ansheng 2017 | CT VS REHAB | -0.05116163 | -1.474931 | 1.372608 | 1 |
| Yu Ansheng 2017 | CT VS TCM | 0.32936896 | -1.41979 | 2.078528 | 1 |
| Yu Ansheng 2017 | CT VS TCM+MOXI | -1.4767251 | -2.24737 | -0.7060799 | 1 |
| Yu Ansheng 2017 | CT VS TUINA | -0.37612963 | -2.140068 | 1.387809 | 1 |
| Yu Ansheng 2017 | CT VS TUINA+MOXI | -2.1856204 | -3.408513 | -0.9627282 | 1 |
| Yin Shaojian 2022 | CT VS ACU | -0.727886 | -1.746296 | 0.2905239 | 1 |
| Yin Shaojian 2022 | CT VS ACU+MOXI | -2.0033357 | -3.060435 | -0.9462366 | 1 |
| Yin Shaojian 2022 | CT VS CT+MOXI | -2.0242397 | -3.134602 | -0.9138774 | 1 |
| Yin Shaojian 2022 | CT VS MOXI | -1.7453122 | -2.714455 | -0.7761699 | 1 |
| Yin Shaojian 2022 | CT VS REHAB | -0.05411564 | -1.432537 | 1.324306 | 1 |
| Yin Shaojian 2022 | CT VS TCM | 0.48102597 | -1.222875 | 2.184927 | 1 |
| Yin Shaojian 2022 | CT VS TCM+MOXI | -1.3242331 | -2.095182 | -0.5532844 | 1 |
| Yin Shaojian 2022 | CT VS TUINA | -0.41407055 | -2.121735 | 1.293594 | 1 |
| Yin Shaojian 2022 | CT VS TUINA+MOXI | -2.2232877 | -3.40817 | -1.038406 | 1 |
| Xu Zhenkai 2018 | CT VS ACU | -0.69113463 | -1.742388 | 0.3601187 | 1 |
| Xu Zhenkai 2018 | CT VS ACU+MOXI | -1.9737383 | -3.065495 | -0.8819819 | 1 |
| Xu Zhenkai 2018 | CT VS CT+MOXI | -2.0141984 | -3.161657 | -0.8667397 | 1 |
| Xu Zhenkai 2018 | CT VS MOXI | -1.4878805 | -2.426552 | -0.5492089 | 1 |
| Xu Zhenkai 2018 | CT VS REHAB | -0.04910833 | -1.472613 | 1.374396 | 1 |
| Xu Zhenkai 2018 | CT VS TCM | 0.3326237 | -1.416016 | 2.081263 | 1 |
| Xu Zhenkai 2018 | CT VS TCM+MOXI | -1.4734633 | -2.243505 | -0.703422 | 1 |
| Xu Zhenkai 2018 | CT VS TUINA | -0.51702857 | -2.492669 | 1.458612 | 1 |
| Xu Zhenkai 2018 | CT VS TUINA+MOXI | -2.1838335 | -3.406575 | -0.9610914 | 1 |
| Feng Minhe 2021 | CT VS ACU | -0.68438105 | -1.743309 | 0.3745469 | 1 |
| Feng Minhe 2021 | CT VS ACU+MOXI | -1.9861233 | -3.090384 | -0.8818622 | 1 |
| Feng Minhe 2021 | CT VS CT+MOXI | -2.0138963 | -3.163121 | -0.8646721 | 1 |
| Feng Minhe 2021 | CT VS MOXI | -1.4855717 | -2.426448 | -0.544695 | 1 |
| Feng Minhe 2021 | CT VS REHAB | -0.04775998 | -1.473433 | 1.377913 | 1 |
| Feng Minhe 2021 | CT VS TCM | 0.33437878 | -1.417167 | 2.085924 | 1 |
| Feng Minhe 2021 | CT VS TCM+MOXI | -1.4717373 | -2.243484 | -0.699991 | 1 |
| Feng Minhe 2021 | CT VS TUINA | -0.37317602 | -2.139503 | 1.39315 | 1 |
| Feng Minhe 2021 | CT VS TUINA+MOXI | -2.1826753 | -3.40721 | -0.9581406 | 1 |
| Wang Jianying 2025 | CT VS ACU | -0.14399816 | -1.187012 | 0.8990161 | 1 |
| Wang Jianying 2025 | CT VS ACU+MOXI | -1.2302898 | -2.373393 | -0.0871868 | 1 |
| Wang Jianying 2025 | CT VS CT+MOXI | -1.9958501 | -3.036983 | -0.9547169 | 1 |
| Wang Jianying 2025 | CT VS MOXI | -1.3037451 | -2.16778 | -0.4397097 | 1 |
| Wang Jianying 2025 | CT VS REHAB | 0.0368237 | -1.259523 | 1.33317 | 1 |
| Wang Jianying 2025 | CT VS TCM | 0.4685088 | -1.122791 | 2.059809 | 1 |
| Wang Jianying 2025 | CT VS TCM+MOXI | -1.3353264 | -2.042481 | -0.6281716 | 1 |
| Wang Jianying 2025 | CT VS TUINA | -0.29433409 | -1.896724 | 1.308056 | 1 |
| Wang Jianying 2025 | CT VS TUINA+MOXI | -2.1027031 | -3.2158 | -0.9896058 | 1 |
| Sun Jian 2009 | CT VS ACU | -0.6360029 | -1.676316 | 0.40431 | 1 |
| Sun Jian 2009 | CT VS ACU+MOXI | -2.0546542 | -3.139429 | -0.9698792 | 1 |
| Sun Jian 2009 | CT VS CT+MOXI | -2.0124047 | -3.141488 | -0.8833217 | 1 |
| Sun Jian 2009 | CT VS MOXI | -1.4692889 | -2.393785 | -0.5447934 | 1 |
| Sun Jian 2009 | CT VS REHAB | -0.04075134 | -1.442025 | 1.360522 | 1 |
| Sun Jian 2009 | CT VS TCM | 0.346156 | -1.37492 | 2.067232 | 1 |
| Sun Jian 2009 | CT VS TCM+MOXI | -1.4595881 | -2.217883 | -0.7012935 | 1 |
| Sun Jian 2009 | CT VS TUINA | -0.36631722 | -2.101934 | 1.369299 | 1 |
| Sun Jian 2009 | CT VS TUINA+MOXI | -2.1756258 | -3.379125 | -0.9721264 | 1 |
| Sun Jianbing 2025 | CT VS ACU | -0.65517695 | -1.712469 | 0.4021153 | 1 |
| Sun Jianbing 2025 | CT VS ACU+MOXI | -1.9451357 | -3.039437 | -0.8508345 | 1 |
| Sun Jianbing 2025 | CT VS CT+MOXI | -2.0085107 | -3.153232 | -0.8637896 | 1 |
| Sun Jianbing 2025 | CT VS MOXI | -1.4636636 | -2.404363 | -0.5229645 | 1 |
| Sun Jianbing 2025 | CT VS REHAB | -0.01567288 | -1.44109 | 1.409744 | 1 |
| Sun Jianbing 2025 | CT VS TCM | 0.4091431 | -1.358588 | 2.176874 | 1 |
| Sun Jianbing 2025 | CT VS TCM+MOXI | -1.396812 | -2.217015 | -0.5766094 | 1 |
| Sun Jianbing 2025 | CT VS TUINA | -0.3509695 | -2.112231 | 1.410292 | 1 |
| Sun Jianbing 2025 | CT VS TUINA+MOXI | -2.1603884 | -3.383048 | -0.937729 | 1 |
| Gao Yuanguanyu 2021 | CT VS ACU | -0.69781162 | -1.759427 | 0.3638035 | 1 |
| Gao Yuanguanyu 2021 | CT VS ACU+MOXI | -1.9790382 | -3.077847 | -0.8802292 | 1 |
| Gao Yuanguanyu 2021 | CT VS CT+MOXI | -2.0152083 | -3.164683 | -0.8657338 | 1 |
| Gao Yuanguanyu 2021 | CT VS MOXI | -1.4923775 | -2.436929 | -0.5478259 | 1 |
| Gao Yuanguanyu 2021 | CT VS REHAB | -0.05517661 | -1.486345 | 1.375992 | 1 |
| Gao Yuanguanyu 2021 | CT VS TCM | 0.31829413 | -1.456592 | 2.09318 | 1 |
| Gao Yuanguanyu 2021 | CT VS TCM+MOXI | -1.48784 | -2.311136 | -0.664544 | 1 |
| Gao Yuanguanyu 2021 | CT VS TUINA | -0.37854541 | -2.147051 | 1.38996 | 1 |
| Gao Yuanguanyu 2021 | CT VS TUINA+MOXI | -2.1880547 | -3.415673 | -0.9604366 | 1 |
| Jiang Yuqin 2025 | CT VS ACU | -0.6841271 | -1.743287 | 0.3750324 | 1 |
| Jiang Yuqin 2025 | CT VS ACU+MOXI | -1.9884245 | -3.092936 | -0.8839134 | 1 |
| Jiang Yuqin 2025 | CT VS CT+MOXI | -2.0138877 | -3.163061 | -0.8647144 | 1 |
| Jiang Yuqin 2025 | CT VS MOXI | -1.4854861 | -2.426357 | -0.5446153 | 1 |
| Jiang Yuqin 2025 | CT VS REHAB | -0.04772013 | -1.473337 | 1.377896 | 1 |
| Jiang Yuqin 2025 | CT VS TCM | 0.33444177 | -1.417037 | 2.08592 | 1 |
| Jiang Yuqin 2025 | CT VS TCM+MOXI | -1.4716733 | -2.24341 | -0.699937 | 1 |
| Jiang Yuqin 2025 | CT VS TUINA | -0.37313918 | -2.139391 | 1.393113 | 1 |
| Jiang Yuqin 2025 | CT VS TUINA+MOXI | -2.1826379 | -3.407124 | -0.9581515 | 1 |
| Liu Chunyu 2021 | CT VS ACU | -0.69218029 | -1.744925 | 0.360565 | 1 |
| Liu Chunyu 2021 | CT VS ACU+MOXI | -1.9745583 | -3.06778 | -0.8813364 | 1 |
| Liu Chunyu 2021 | CT VS CT+MOXI | -2.0514726 | -3.3905 | -0.7124456 | 1 |
| Liu Chunyu 2021 | CT VS MOXI | -1.4898009 | -2.430316 | -0.5492854 | 1 |
| Liu Chunyu 2021 | CT VS REHAB | -0.05745245 | -1.491258 | 1.376353 | 1 |
| Liu Chunyu 2021 | CT VS TCM | 0.33144222 | -1.419482 | 2.082366 | 1 |
| Liu Chunyu 2021 | CT VS TCM+MOXI | -1.4746707 | -2.245993 | -0.7033485 | 1 |
| Liu Chunyu 2021 | CT VS TUINA | -0.38449345 | -2.160411 | 1.391424 | 1 |
| Liu Chunyu 2021 | CT VS TUINA+MOXI | -2.1940096 | -3.432797 | -0.9552218 | 1 |
| Yin Zhao 2023 | CT VS ACU | -0.6915909 | -1.734671 | 0.3514893 | 1 |
| Yin Zhao 2023 | CT VS ACU+MOXI | -1.9741821 | -3.057419 | -0.8909449 | 1 |
| Yin Zhao 2023 | CT VS CT+MOXI | -2.0142961 | -3.152761 | -0.8758309 | 1 |
| Yin Zhao 2023 | CT VS MOXI | -1.4880337 | -2.419411 | -0.5566559 | 1 |
| Yin Zhao 2023 | CT VS REHAB | -0.04956126 | -1.462094 | 1.362971 | 1 |
| Yin Zhao 2023 | CT VS TCM | -0.26230573 | -2.577546 | 2.052934 | 1 |
| Yin Zhao 2023 | CT VS TCM+MOXI | -1.4735778 | -2.237682 | -0.7094737 | 1 |
| Yin Zhao 2023 | CT VS TUINA | -0.3744166 | -2.124275 | 1.375442 | 1 |
| Yin Zhao 2023 | CT VS TUINA+MOXI | -2.183823 | -3.39702 | -0.9706258 | 1 |
| Zhang Aili 2023 | CT VS ACU | -0.74554913 | -1.778003 | 0.2869049 | 1 |
| Zhang Aili 2023 | CT VS ACU+MOXI | -2.0172835 | -3.087819 | -0.946748 | 1 |
| Zhang Aili 2023 | CT VS CT+MOXI | -2.1323112 | -3.271885 | -0.9927378 | 1 |
| Zhang Aili 2023 | CT VS MOXI | -1.5807366 | -2.512016 | -0.6494573 | 1 |
| Zhang Aili 2023 | CT VS REHAB | -0.45044891 | -1.994261 | 1.093364 | 1 |
| Zhang Aili 2023 | CT VS TCM | 0.27350453 | -1.440367 | 1.987376 | 1 |
| Zhang Aili 2023 | CT VS TCM+MOXI | -1.5321945 | -2.291906 | -0.7724833 | 1 |
| Zhang Aili 2023 | CT VS TUINA | -0.85715815 | -2.759947 | 1.04563 | 1 |
| Zhang Aili 2023 | CT VS TUINA+MOXI | -2.667237 | -4.108261 | -1.226213 | 1 |
| Hua Yuqi 2015 | CT VS ACU | -0.68202835 | -1.733678 | 0.3696209 | 1 |
| Hua Yuqi 2015 | CT VS ACU+MOXI | -1.9664976 | -3.058074 | -0.8749213 | 1 |
| Hua Yuqi 2015 | CT VS CT+MOXI | -2.0395165 | -3.19546 | -0.8835728 | 1 |
| Hua Yuqi 2015 | CT VS MOXI | -1.4955947 | -2.434478 | -0.5567111 | 1 |
| Hua Yuqi 2015 | CT VS REHAB | -0.22281774 | -1.971655 | 1.52602 | 1 |
| Hua Yuqi 2015 | CT VS TCM | 0.36532298 | -1.392221 | 2.122867 | 1 |
| Hua Yuqi 2015 | CT VS TCM+MOXI | -1.4407107 | -2.233705 | -0.6477165 | 1 |
| Hua Yuqi 2015 | CT VS TUINA | -0.4778902 | -2.341498 | 1.385718 | 1 |
| Hua Yuqi 2015 | CT VS TUINA+MOXI | -2.2875487 | -3.652068 | -0.9230295 | 1 |
| Wu Zongxiang 2017 | CT VS ACU | -0.69135041 | -1.740768 | 0.3580671 | 1 |
| Wu Zongxiang 2017 | CT VS ACU+MOXI | -1.9739282 | -3.063769 | -0.8840873 | 1 |
| Wu Zongxiang 2017 | CT VS CT+MOXI | -2.014467 | -3.159933 | -0.8690014 | 1 |
| Wu Zongxiang 2017 | CT VS MOXI | -1.4881097 | -2.425162 | -0.5510576 | 1 |
| Wu Zongxiang 2017 | CT VS REHAB | -0.05004729 | -1.471377 | 1.371282 | 1 |
| Wu Zongxiang 2017 | CT VS TCM | 0.33243521 | -1.41315 | 2.07802 | 1 |
| Wu Zongxiang 2017 | CT VS TCM+MOXI | -1.4736164 | -2.24232 | -0.704913 | 1 |
| Wu Zongxiang 2017 | CT VS TUINA | -0.58770332 | -2.565737 | 1.39033 | 1 |
| Wu Zongxiang 2017 | CT VS TUINA+MOXI | -2.1848426 | -3.405946 | -0.9637389 | 1 |
| Chen Jing 2016 | CT VS ACU | -0.69191185 | -1.734685 | 0.3508616 | 1 |
| Chen Jing 2016 | CT VS ACU+MOXI | -1.9744407 | -3.057356 | -0.8915256 | 1 |
| Chen Jing 2016 | CT VS CT+MOXI | -2.0149484 | -3.153142 | -0.8767545 | 1 |
| Chen Jing 2016 | CT VS MOXI | -1.4885545 | -2.419703 | -0.5574063 | 1 |
| Chen Jing 2016 | CT VS REHAB | -0.05178302 | -1.464553 | 1.360987 | 1 |
| Chen Jing 2016 | CT VS TCM | 0.33199451 | -1.402552 | 2.066541 | 1 |
| Chen Jing 2016 | CT VS TCM+MOXI | -1.4739279 | -2.237774 | -0.7100821 | 1 |
| Chen Jing 2016 | CT VS TUINA | -0.01976708 | -1.987883 | 1.948349 | 1 |
| Chen Jing 2016 | CT VS TUINA+MOXI | -2.1864814 | -3.400456 | -0.9725071 | 1 |
| Wang Hongfei 2020 | CT VS ACU | -0.69317177 | -1.744067 | 0.3577234 | 1 |
| Wang Hongfei 2020 | CT VS ACU+MOXI | -1.9753644 | -3.066719 | -0.8840097 | 1 |
| Wang Hongfei 2020 | CT VS CT+MOXI | -2.0088874 | -3.156356 | -0.8614185 | 1 |
| Wang Hongfei 2020 | CT VS MOXI | -1.4863246 | -2.424693 | -0.5479559 | 1 |
| Wang Hongfei 2020 | CT VS REHAB | -0.11986136 | -1.599747 | 1.360025 | 1 |
| Wang Hongfei 2020 | CT VS TCM | 0.32553721 | -1.422869 | 2.073943 | 1 |
| Wang Hongfei 2020 | CT VS TCM+MOXI | -1.4805491 | -2.251351 | -0.7097475 | 1 |
| Wang Hongfei 2020 | CT VS TUINA | -0.35260291 | -2.120744 | 1.415538 | 1 |
| Wang Hongfei 2020 | CT VS TUINA+MOXI | -2.1620463 | -3.391796 | -0.9322965 | 1 |
| Zhao Huayun 2023 | CT VS ACU | -0.68688122 | -1.724473 | 0.3507104 | 1 |
| Zhao Huayun 2023 | CT VS ACU+MOXI | -1.970488 | -3.047971 | -0.8930047 | 1 |
| Zhao Huayun 2023 | CT VS CT+MOXI | -2.0313571 | -3.164269 | -0.8984451 | 1 |
| Zhao Huayun 2023 | CT VS MOXI | -1.4939813 | -2.42054 | -0.5674224 | 1 |
| Zhao Huayun 2023 | CT VS REHAB | 0.14830358 | -1.31541 | 1.612017 | 1 |
| Zhao Huayun 2023 | CT VS TCM | 0.35165693 | -1.374646 | 2.07796 | 1 |
| Zhao Huayun 2023 | CT VS TCM+MOXI | -1.4541411 | -2.215241 | -0.6930413 | 1 |
| Zhao Huayun 2023 | CT VS TUINA | -0.44403031 | -2.190698 | 1.302638 | 1 |
| Zhao Huayun 2023 | CT VS TUINA+MOXI | -2.2535016 | -3.469177 | -1.037826 | 1 |
| Li Xiuxia 2019 | CT VS ACU | -0.6182341 | -1.67732 | 0.440852 | 1 |
| Li Xiuxia 2019 | CT VS ACU+MOXI | -1.9157802 | -3.008739 | -0.8228214 | 1 |
| Li Xiuxia 2019 | CT VS CT+MOXI | -2.0005946 | -3.139374 | -0.8618153 | 1 |
| Li Xiuxia 2019 | CT VS MOXI | -1.3148177 | -2.342834 | -0.2868016 | 1 |
| Li Xiuxia 2019 | CT VS REHAB | 0.00267885 | -1.415694 | 1.421052 | 1 |
| Li Xiuxia 2019 | CT VS TCM | 0.36914231 | -1.368067 | 2.106352 | 1 |
| Li Xiuxia 2019 | CT VS TCM+MOXI | -1.4367439 | -2.206257 | -0.6672304 | 1 |
| Li Xiuxia 2019 | CT VS TUINA | -0.31833283 | -2.073547 | 1.436881 | 1 |
| Li Xiuxia 2019 | CT VS TUINA+MOXI | -2.1276401 | -3.348816 | -0.9064637 | 1 |
| Xu Mengxia 2019 | CT VS ACU | -0.71294865 | -1.768781 | 0.3428838 | 1 |
| Xu Mengxia 2019 | CT VS ACU+MOXI | -1.9447675 | -3.045675 | -0.8438605 | 1 |
| Xu Mengxia 2019 | CT VS CT+MOXI | -2.0149704 | -3.161296 | -0.8686447 | 1 |
| Xu Mengxia 2019 | CT VS MOXI | -1.4952319 | -2.433698 | -0.5567661 | 1 |
| Xu Mengxia 2019 | CT VS REHAB | -0.05272283 | -1.474879 | 1.369433 | 1 |
| Xu Mengxia 2019 | CT VS TCM | 0.32710807 | -1.420038 | 2.074254 | 1 |
| Xu Mengxia 2019 | CT VS TCM+MOXI | -1.4789649 | -2.24874 | -0.7091902 | 1 |
| Xu Mengxia 2019 | CT VS TUINA | -0.37746496 | -2.139368 | 1.384438 | 1 |
| Xu Mengxia 2019 | CT VS TUINA+MOXI | -2.1869463 | -3.408447 | -0.9654453 | 1 |
| Pan Meng 2025 | CT VS ACU | -0.65862537 | -1.711044 | 0.393793 | 1 |
| Pan Meng 2025 | CT VS ACU+MOXI | -2.0206862 | -3.118088 | -0.9232848 | 1 |
| Pan Meng 2025 | CT VS CT+MOXI | -2.0130494 | -3.155534 | -0.8705649 | 1 |
| Pan Meng 2025 | CT VS MOXI | -1.4768899 | -2.412249 | -0.5415305 | 1 |
| Pan Meng 2025 | CT VS REHAB | -0.04380498 | -1.46131 | 1.3737 | 1 |
| Pan Meng 2025 | CT VS TCM | 0.34073469 | -1.400604 | 2.082074 | 1 |
| Pan Meng 2025 | CT VS TCM+MOXI | -1.4652562 | -2.232477 | -0.6980356 | 1 |
| Pan Meng 2025 | CT VS TUINA | -0.36946403 | -2.125512 | 1.386584 | 1 |
| Pan Meng 2025 | CT VS TUINA+MOXI | -2.1788974 | -3.396389 | -0.9614055 | 1 |
| Zhou Huaming 2024 | CT VS ACU | -0.70697509 | -1.685016 | 0.2710658 | 1 |
| Zhou Huaming 2024 | CT VS ACU+MOXI | -1.9871109 | -3.002555 | -0.9716665 | 1 |
| Zhou Huaming 2024 | CT VS CT+MOXI | -1.9827645 | -3.050195 | -0.9153342 | 1 |
| Zhou Huaming 2024 | CT VS MOXI | -1.4792303 | -2.352688 | -0.6057725 | 1 |
| Zhou Huaming 2024 | CT VS REHAB | -0.46867634 | -1.844655 | 0.9073026 | 1 |
| Zhou Huaming 2024 | CT VS TCM | 0.28864006 | -1.338801 | 1.916081 | 1 |
| Zhou Huaming 2024 | CT VS TCM+MOXI | -1.5159295 | -2.23327 | -0.7985885 | 1 |
| Zhou Huaming 2024 | CT VS TUINA | -0.24194727 | -1.887242 | 1.403348 | 1 |
| Zhou Huaming 2024 | CT VS TUINA+MOXI | -2.0504883 | -3.195406 | -0.9055701 | 1 |
| Li Jia 2024 | CT VS ACU | -0.64194785 | -1.779997 | 0.4961008 | 1 |
| Li Jia 2024 | CT VS ACU+MOXI | -1.9345639 | -3.080688 | -0.7884397 | 1 |
| Li Jia 2024 | CT VS CT+MOXI | -2.0144405 | -3.163295 | -0.8655864 | 1 |
| Li Jia 2024 | CT VS MOXI | -1.4769487 | -2.421823 | -0.5320744 | 1 |
| Li Jia 2024 | CT VS REHAB | -0.05365131 | -1.47929 | 1.371987 | 1 |
| Li Jia 2024 | CT VS TCM | 0.31309488 | -1.445386 | 2.071576 | 1 |
| Li Jia 2024 | CT VS TCM+MOXI | -1.4930374 | -2.281428 | -0.7046469 | 1 |
| Li Jia 2024 | CT VS TUINA | -0.37538921 | -2.141109 | 1.390331 | 1 |
| Li Jia 2024 | CT VS TUINA+MOXI | -2.1848892 | -3.408971 | -0.9608071 | 1 |
| Xiong Qi 2024 | CT VS ACU | -0.68519575 | -1.689324 | 0.3189321 | 1 |
| Xiong Qi 2024 | CT VS ACU+MOXI | -1.9694919 | -3.012097 | -0.9268868 | 1 |
| Xiong Qi 2024 | CT VS CT+MOXI | -2.0441201 | -3.140329 | -0.9479113 | 1 |
| Xiong Qi 2024 | CT VS MOXI | -1.4989214 | -2.39571 | -0.6021324 | 1 |
| Xiong Qi 2024 | CT VS REHAB | 0.29139681 | -1.12764 | 1.710434 | 1 |
| Xiong Qi 2024 | CT VS TCM | 0.36460036 | -1.306131 | 2.035332 | 1 |
| Xiong Qi 2024 | CT VS TCM+MOXI | -1.4404877 | -2.177098 | -0.7038773 | 1 |
| Xiong Qi 2024 | CT VS TUINA | -0.49502124 | -2.186255 | 1.196213 | 1 |
| Xiong Qi 2024 | CT VS TUINA+MOXI | -2.3042304 | -3.482408 | -1.126053 | 1 |

**Sensitivity analysis based on the JOA score group in the primary analysis of patients with LDH. (Table S52)**

| **dropped_id** | **comparison** | **eff** | **lci** | **uci** | **connected** |
| --- | --- | --- | --- | --- | --- |
| Chunyue Cai 2018 | CT VS ACU | 0.27137416 | -2.164659 | 2.707407 | 1 |
| Chunyue Cai 2018 | CT VS ACU+MOXI | 3.5422889 | 0.6482174 | 6.43636 | 1 |
| Chunyue Cai 2018 | CT VS CT+MOXI | 11.930001 | 8.892393 | 14.96761 | 1 |
| Chunyue Cai 2018 | CT VS MOXI | 5.0560061 | 3.49697 | 6.615042 | 1 |
| Chunyue Cai 2018 | CT VS REHAB | 2.8273451 | -1.030639 | 6.685329 | 1 |
| Chunyue Cai 2018 | CT VS TCM | -0.35909906 | -2.796345 | 2.078147 | 1 |
| Chunyue Cai 2018 | CT VS TCM+MOXI | 3.8875147 | 2.359088 | 5.415941 | 1 |
| Chunyue Cai 2018 | CT VS TUINA | 3.5729827 | -0.2520401 | 7.398005 | 1 |
| Chunyue Cai 2018 | CT VS TUINA+MOXI | 7.6100941 | 4.196851 | 11.02334 | 1 |
| Xu Minmin 2016 | CT VS ACU | 0.83839898 | -1.361499 | 3.038297 | 1 |
| Xu Minmin 2016 | CT VS ACU+MOXI | 4.1124482 | 1.399351 | 6.825546 | 1 |
| Xu Minmin 2016 | CT VS CT+MOXI | 11.930001 | 8.82661 | 15.03339 | 1 |
| Xu Minmin 2016 | CT VS MOXI | 4.9612235 | 3.161799 | 6.760648 | 1 |
| Xu Minmin 2016 | CT VS REHAB | 2.7294827 | -1.296498 | 6.755464 | 1 |
| Xu Minmin 2016 | CT VS TCM | -0.58566887 | -3.052979 | 1.881641 | 1 |
| Xu Minmin 2016 | CT VS TCM+MOXI | 3.6607285 | 2.12835 | 5.193107 | 1 |
| Xu Minmin 2016 | CT VS TUINA | 3.4763825 | -0.5182373 | 7.471003 | 1 |
| Xu Minmin 2016 | CT VS TUINA+MOXI | 7.5146503 | 3.930469 | 11.09883 | 1 |
| Zhang Qun 2023 | CT VS ACU | 1.6304771 | -0.3647917 | 3.625746 | 1 |
| Zhang Qun 2023 | CT VS ACU+MOXI | 4.8803381 | 2.435958 | 7.324718 | 1 |
| Zhang Qun 2023 | CT VS CT+MOXI | 11.930001 | 9.253184 | 14.60682 | 1 |
| Zhang Qun 2023 | CT VS MOXI | 5.3889805 | 4.008932 | 6.769029 | 1 |
| Zhang Qun 2023 | CT VS REHAB | 3.1810435 | -0.2296654 | 6.591753 | 1 |
| Zhang Qun 2023 | CT VS TCM | 0.35172258 | -1.956495 | 2.65994 | 1 |
| Zhang Qun 2023 | CT VS TCM+MOXI | 4.5995934 | 3.044921 | 6.154265 | 1 |
| Zhang Qun 2023 | CT VS TUINA | 3.9183836 | 0.5444813 | 7.292286 | 1 |
| Zhang Qun 2023 | CT VS TUINA+MOXI | 7.9475849 | 4.933123 | 10.96205 | 1 |
| Hu Qinqin 2024 | CT VS ACU | 0.93847042 | -1.294915 | 3.171855 | 1 |
| Hu Qinqin 2024 | CT VS ACU+MOXI | 4.214226 | 1.465403 | 6.963049 | 1 |
| Hu Qinqin 2024 | CT VS CT+MOXI | 11.930001 | 8.789923 | 15.07008 | 1 |
| Hu Qinqin 2024 | CT VS MOXI | 5.2380354 | 3.662436 | 6.813635 | 1 |
| Hu Qinqin 2024 | CT VS REHAB | 3.002948 | -0.9682302 | 6.974126 | 1 |
| Hu Qinqin 2024 | CT VS TCM | -0.60381137 | -3.216332 | 2.008709 | 1 |
| Hu Qinqin 2024 | CT VS TCM+MOXI | 3.6424809 | 1.907789 | 5.377173 | 1 |
| Hu Qinqin 2024 | CT VS TUINA | 3.7504024 | -0.1886891 | 7.689494 | 1 |
| Hu Qinqin 2024 | CT VS TUINA+MOXI | 7.7894691 | 4.278522 | 11.30042 | 1 |
| Xu Fen 2025 | CT VS ACU | 1.4419482 | -0.5442241 | 3.428121 | 1 |
| Xu Fen 2025 | CT VS ACU+MOXI | 4.6957438 | 2.240843 | 7.150644 | 1 |
| Xu Fen 2025 | CT VS CT+MOXI | 11.930001 | 9.193083 | 14.66692 | 1 |
| Xu Fen 2025 | CT VS MOXI | 6.1136615 | 4.513987 | 7.713336 | 1 |
| Xu Fen 2025 | CT VS REHAB | 3.8988248 | 0.3322582 | 7.465392 | 1 |
| Xu Fen 2025 | CT VS TCM | -0.42193689 | -2.638978 | 1.795104 | 1 |
| Xu Fen 2025 | CT VS TCM+MOXI | 3.8256456 | 2.449454 | 5.201837 | 1 |
| Xu Fen 2025 | CT VS TUINA | 4.6375274 | 1.106616 | 8.168439 | 1 |
| Xu Fen 2025 | CT VS TUINA+MOXI | 8.6685767 | 5.496706 | 11.84045 | 1 |
| Zhong Qunhua 2024 | CT VS ACU | 0.98876715 | -1.133744 | 3.111278 | 1 |
| Zhong Qunhua 2024 | CT VS ACU+MOXI | 4.2603124 | 1.622092 | 6.898532 | 1 |
| Zhong Qunhua 2024 | CT VS CT+MOXI | 5.2472751 | 3.715357 | 6.779193 | 1 |
| Zhong Qunhua 2024 | CT VS MOXI | 3.0168882 | -0.8453216 | 6.879098 | 1 |
| Zhong Qunhua 2024 | CT VS REHAB | -0.54022852 | -2.967425 | 1.886968 | 1 |
| Zhong Qunhua 2024 | CT VS TCM | 3.7063219 | 2.203788 | 5.208856 | 1 |
| Zhong Qunhua 2024 | CT VS TCM+MOXI | 3.7627382 | -0.0666035 | 7.59208 | 1 |
| Zhong Qunhua 2024 | CT VS TUINA | 7.8002229 | 4.38638 | 11.21407 | 1 |
| Liu Wenlin 2024 | CT VS ACU | 0.98844792 | -1.140014 | 3.11691 | 1 |
| Liu Wenlin 2024 | CT VS ACU+MOXI | 3.907445 | 1.14643 | 6.66846 | 1 |
| Liu Wenlin 2024 | CT VS CT+MOXI | 11.930001 | 8.867319 | 14.99268 | 1 |
| Liu Wenlin 2024 | CT VS MOXI | 5.2477126 | 3.711301 | 6.784124 | 1 |
| Liu Wenlin 2024 | CT VS REHAB | 3.0167447 | -0.8582191 | 6.891708 | 1 |
| Liu Wenlin 2024 | CT VS TCM | -0.53944179 | -2.973913 | 1.89503 | 1 |
| Liu Wenlin 2024 | CT VS TCM+MOXI | 3.707078 | 2.199969 | 5.214188 | 1 |
| Liu Wenlin 2024 | CT VS TUINA | 3.7627929 | -0.0793931 | 7.604979 | 1 |
| Liu Wenlin 2024 | CT VS TUINA+MOXI | 7.8004739 | 4.375337 | 11.22561 | 1 |
| Wu Yusheng 2025 | CT VS ACU | 0.65531217 | -1.474021 | 2.784646 | 1 |
| Wu Yusheng 2025 | CT VS ACU+MOXI | 3.9224489 | 1.298926 | 6.545971 | 1 |
| Wu Yusheng 2025 | CT VS CT+MOXI | 11.930001 | 8.964823 | 14.89518 | 1 |
| Wu Yusheng 2025 | CT VS MOXI | 5.1550359 | 3.654741 | 6.655331 | 1 |
| Wu Yusheng 2025 | CT VS REHAB | 2.9299745 | -0.8290945 | 6.689044 | 1 |
| Wu Yusheng 2025 | CT VS TCM | -1.0564253 | -3.53302 | 1.42017 | 1 |
| Wu Yusheng 2025 | CT VS TCM+MOXI | 3.1903311 | 1.55465 | 4.826012 | 1 |
| Wu Yusheng 2025 | CT VS TUINA | 3.6741406 | -0.0512225 | 7.399504 | 1 |
| Wu Yusheng 2025 | CT VS TUINA+MOXI | 7.7098907 | 4.386953 | 11.03283 | 1 |
| Xu Shihai 2019 | CT VS ACU | 1.0993817 | -1.335507 | 3.534271 | 1 |
| Xu Shihai 2019 | CT VS ACU+MOXI | 4.3746811 | 1.463086 | 7.286276 | 1 |
| Xu Shihai 2019 | CT VS CT+MOXI | 11.930001 | 8.799287 | 15.06071 | 1 |
| Xu Shihai 2019 | CT VS MOXI | 5.2807915 | 3.687742 | 6.87384 | 1 |
| Xu Shihai 2019 | CT VS REHAB | 3.045982 | -0.9225053 | 7.014469 | 1 |
| Xu Shihai 2019 | CT VS TCM | -0.56451499 | -3.06214 | 1.93311 | 1 |
| Xu Shihai 2019 | CT VS TCM+MOXI | 3.681808 | 2.119239 | 5.244377 | 1 |
| Xu Shihai 2019 | CT VS TUINA | 3.7932564 | -0.1430673 | 7.72958 | 1 |
| Xu Shihai 2019 | CT VS TUINA+MOXI | 7.832183 | 4.321632 | 11.34273 | 1 |
| Liang Yuanyuan 2014 | CT VS ACU | 0.60177447 | -1.493875 | 2.697423 | 1 |
| Liang Yuanyuan 2014 | CT VS ACU+MOXI | 3.8656089 | 1.283464 | 6.447754 | 1 |
| Liang Yuanyuan 2014 | CT VS CT+MOXI | 11.930001 | 9.025824 | 14.83418 | 1 |
| Liang Yuanyuan 2014 | CT VS MOXI | 5.1384797 | 3.664057 | 6.612902 | 1 |
| Liang Yuanyuan 2014 | CT VS REHAB | 2.9170485 | -0.7681674 | 6.602264 | 1 |
| Liang Yuanyuan 2014 | CT VS TCM | -1.1452414 | -3.580013 | 1.28953 | 1 |
| Liang Yuanyuan 2014 | CT VS TCM+MOXI | 3.1017 | 1.492314 | 4.711086 | 1 |
| Liang Yuanyuan 2014 | CT VS TUINA | 3.6599334 | 0.0090297 | 7.310837 | 1 |
| Liang Yuanyuan 2014 | CT VS TUINA+MOXI | 7.6944148 | 4.436908 | 10.95192 | 1 |
| Liu Yiyi 2022 | CT VS ACU | 0.98610288 | -1.165486 | 3.137691 | 1 |
| Liu Yiyi 2022 | CT VS ACU+MOXI | 4.0960367 | 1.329692 | 6.862382 | 1 |
| Liu Yiyi 2022 | CT VS CT+MOXI | 11.930001 | 8.82559 | 15.03441 | 1 |
| Liu Yiyi 2022 | CT VS MOXI | 5.2491269 | 3.695026 | 6.803228 | 1 |
| Liu Yiyi 2022 | CT VS REHAB | 3.0158854 | -0.909625 | 6.941396 | 1 |
| Liu Yiyi 2022 | CT VS TCM | -0.53676016 | -2.999836 | 1.926316 | 1 |
| Liu Yiyi 2022 | CT VS TCM+MOXI | 3.7096401 | 2.184771 | 5.234509 | 1 |
| Liu Yiyi 2022 | CT VS TUINA | 3.7627015 | -0.1303931 | 7.655796 | 1 |
| Liu Yiyi 2022 | CT VS TUINA+MOXI | 7.8011441 | 4.331257 | 11.27103 | 1 |
| Guo Hui 2023 | CT VS ACU | 0.98421469 | -1.185709 | 3.154139 | 1 |
| Guo Hui 2023 | CT VS ACU+MOXI | 4.2598435 | 1.563213 | 6.956474 | 1 |
| Guo Hui 2023 | CT VS CT+MOXI | 11.930001 | 8.792527 | 15.06748 | 1 |
| Guo Hui 2023 | CT VS MOXI | 5.2502021 | 3.682071 | 6.818333 | 1 |
| Guo Hui 2023 | CT VS REHAB | 3.0151901 | -0.9503737 | 6.980754 | 1 |
| Guo Hui 2023 | CT VS TCM | -0.33222855 | -3.193769 | 2.529312 | 1 |
| Guo Hui 2023 | CT VS TCM+MOXI | 3.7116336 | 2.172621 | 5.250646 | 1 |
| Guo Hui 2023 | CT VS TUINA | 3.7625944 | -0.1708313 | 7.69602 | 1 |
| Guo Hui 2023 | CT VS TUINA+MOXI | 7.8016225 | 4.296278 | 11.30697 | 1 |
| Wen Qi 2023 | CT VS ACU | 1.0162481 | -1.423626 | 3.456122 | 1 |
| Wen Qi 2023 | CT VS ACU+MOXI | 4.2913647 | 1.37622 | 7.206509 | 1 |
| Wen Qi 2023 | CT VS CT+MOXI | 11.930001 | 8.80345 | 15.05655 | 1 |
| Wen Qi 2023 | CT VS MOXI | 5.2389904 | 3.627163 | 6.850817 | 1 |
| Wen Qi 2023 | CT VS REHAB | 3.0046159 | -0.9666948 | 6.975926 | 1 |
| Wen Qi 2023 | CT VS TCM | -0.52501099 | -3.030722 | 1.9807 | 1 |
| Wen Qi 2023 | CT VS TCM+MOXI | 3.7213283 | 2.143052 | 5.299604 | 1 |
| Wen Qi 2023 | CT VS TUINA | 3.7518317 | -0.1874558 | 7.691119 | 1 |
| Wen Qi 2023 | CT VS TUINA+MOXI | 7.790662 | 4.275422 | 11.3059 | 1 |
| Lin Mingqin 2021 | CT VS ACU | 1.1053627 | -1.073603 | 3.284328 | 1 |
| Lin Mingqin 2021 | CT VS ACU+MOXI | 4.379295 | 1.683191 | 7.075399 | 1 |
| Lin Mingqin 2021 | CT VS CT+MOXI | 11.930001 | 8.82832 | 15.03168 | 1 |
| Lin Mingqin 2021 | CT VS MOXI | 5.4816214 | 3.777258 | 7.185985 | 1 |
| Lin Mingqin 2021 | CT VS REHAB | 3.2473606 | -0.7359338 | 7.230655 | 1 |
| Lin Mingqin 2021 | CT VS TCM | -0.49755962 | -2.961947 | 1.966828 | 1 |
| Lin Mingqin 2021 | CT VS TCM+MOXI | 3.7488529 | 2.22003 | 5.277676 | 1 |
| Lin Mingqin 2021 | CT VS TUINA | 3.9940448 | 0.0425104 | 7.94558 | 1 |
| Lin Mingqin 2021 | CT VS TUINA+MOXI | 8.0325647 | 4.49612 | 11.56901 | 1 |
| Zhang Jing 2026 | CT VS ACU | 0.98390448 | -1.189406 | 3.157215 | 1 |
| Zhang Jing 2026 | CT VS ACU+MOXI | 4.2598147 | 1.559008 | 6.960622 | 1 |
| Zhang Jing 2026 | CT VS CT+MOXI | 11.930001 | 8.786439 | 15.07356 | 1 |
| Zhang Jing 2026 | CT VS MOXI | 5.2504066 | 3.67969 | 6.821123 | 1 |
| Zhang Jing 2026 | CT VS REHAB | 3.0150713 | -0.9578692 | 6.988012 | 1 |
| Zhang Jing 2026 | CT VS TCM | -0.65392456 | -3.538164 | 2.230315 | 1 |
| Zhang Jing 2026 | CT VS TCM+MOXI | 3.7120126 | 2.170396 | 5.25363 | 1 |
| Zhang Jing 2026 | CT VS TUINA | 3.7625821 | -0.1782707 | 7.703435 | 1 |
| Zhang Jing 2026 | CT VS TUINA+MOXI | 7.8017164 | 4.289842 | 11.31359 | 1 |
| Zhu Bin 2021 | CT VS ACU | 1.4931841 | -0.8084964 | 3.794865 | 1 |
| Zhu Bin 2021 | CT VS ACU+MOXI | 4.7641027 | 1.980814 | 7.547391 | 1 |
| Zhu Bin 2021 | CT VS CT+MOXI | 11.930001 | 8.889156 | 14.97085 | 1 |
| Zhu Bin 2021 | CT VS MOXI | 5.0718931 | 3.513003 | 6.630783 | 1 |
| Zhu Bin 2021 | CT VS REHAB | 2.842978 | -1.018484 | 6.70444 | 1 |
| Zhu Bin 2021 | CT VS TCM | -0.3745102 | -2.813405 | 2.064384 | 1 |
| Zhu Bin 2021 | CT VS TCM+MOXI | 3.8720922 | 2.34321 | 5.400974 | 1 |
| Zhu Bin 2021 | CT VS TUINA | 3.5886726 | -0.2397965 | 7.417142 | 1 |
| Zhu Bin 2021 | CT VS TUINA+MOXI | 7.6258535 | 4.209768 | 11.04194 | 1 |
| Xu Zhenkai 2018 | CT VS ACU | 0.98558056 | -1.177335 | 3.148496 | 1 |
| Xu Zhenkai 2018 | CT VS ACU+MOXI | 4.2606215 | 1.572633 | 6.94861 | 1 |
| Xu Zhenkai 2018 | CT VS CT+MOXI | 11.930001 | 8.805157 | 15.05485 | 1 |
| Xu Zhenkai 2018 | CT VS MOXI | 5.2512093 | 3.68824 | 6.814178 | 1 |
| Xu Zhenkai 2018 | CT VS REHAB | 3.0226564 | -0.9293457 | 6.974658 | 1 |
| Xu Zhenkai 2018 | CT VS TCM | -0.5352861 | -3.012401 | 1.941828 | 1 |
| Xu Zhenkai 2018 | CT VS TCM+MOXI | 3.7110568 | 2.177458 | 5.244656 | 1 |
| Xu Zhenkai 2018 | CT VS TUINA | 3.3834121 | -0.7404364 | 7.50726 | 1 |
| Xu Zhenkai 2018 | CT VS TUINA+MOXI | 7.8086457 | 4.314819 | 11.30247 | 1 |
| Wang Guoshu 2017 | CT VS ACU | 0.98518623 | -1.175381 | 3.145753 | 1 |
| Wang Guoshu 2017 | CT VS ACU+MOXI | 4.2822112 | 1.507222 | 7.057201 | 1 |
| Wang Guoshu 2017 | CT VS CT+MOXI | 11.930001 | 8.809422 | 15.05058 | 1 |
| Wang Guoshu 2017 | CT VS MOXI | 5.2496585 | 3.688697 | 6.810619 | 1 |
| Wang Guoshu 2017 | CT VS REHAB | 3.015548 | -0.9295481 | 6.960644 | 1 |
| Wang Guoshu 2017 | CT VS TCM | -0.53575782 | -3.009932 | 1.938416 | 1 |
| Wang Guoshu 2017 | CT VS TCM+MOXI | 3.7105969 | 2.178834 | 5.24236 | 1 |
| Wang Guoshu 2017 | CT VS TUINA | 3.7626539 | -0.1501632 | 7.675471 | 1 |
| Wang Guoshu 2017 | CT VS TUINA+MOXI | 7.8013847 | 4.314159 | 11.28861 | 1 |
| Sun Jian 2009 | CT VS ACU | 0.98428713 | -1.186964 | 3.155539 | 1 |
| Sun Jian 2009 | CT VS ACU+MOXI | 4.3535428 | 1.517838 | 7.189248 | 1 |
| Sun Jian 2009 | CT VS CT+MOXI | 11.930001 | 8.7903 | 15.0697 | 1 |
| Sun Jian 2009 | CT VS MOXI | 5.2503269 | 3.681242 | 6.819412 | 1 |
| Sun Jian 2009 | CT VS REHAB | 3.0151964 | -0.9530688 | 6.983461 | 1 |
| Sun Jian 2009 | CT VS TCM | -0.53453473 | -3.021851 | 1.952782 | 1 |
| Sun Jian 2009 | CT VS TCM+MOXI | 3.7117669 | 2.171833 | 5.251701 | 1 |
| Sun Jian 2009 | CT VS TUINA | 3.7626397 | -0.1735061 | 7.698786 | 1 |
| Sun Jian 2009 | CT VS TUINA+MOXI | 7.8017067 | 4.29397 | 11.30944 | 1 |
| Xu Chenghua 2018 | CT VS ACU | 0.98638629 | -1.164188 | 3.136961 | 1 |
| Xu Chenghua 2018 | CT VS ACU+MOXI | 4.2603796 | 1.587618 | 6.933141 | 1 |
| Xu Chenghua 2018 | CT VS CT+MOXI | 11.930001 | 8.827372 | 15.03263 | 1 |
| Xu Chenghua 2018 | CT VS MOXI | 5.2497065 | 3.696239 | 6.803174 | 1 |
| Xu Chenghua 2018 | CT VS REHAB | 3.0193035 | -0.905136 | 6.943743 | 1 |
| Xu Chenghua 2018 | CT VS TCM | -0.53681146 | -2.998677 | 1.925054 | 1 |
| Xu Chenghua 2018 | CT VS TCM+MOXI | 3.7095938 | 2.185466 | 5.233722 | 1 |
| Xu Chenghua 2018 | CT VS TUINA | 4.2045388 | 0.132274 | 8.276804 | 1 |
| Xu Chenghua 2018 | CT VS TUINA+MOXI | 7.8044986 | 4.335264 | 11.27373 | 1 |
| Jiang Yuqin 2025 | CT VS ACU | 0.98871087 | -1.139429 | 3.11685 | 1 |
| Jiang Yuqin 2025 | CT VS ACU+MOXI | 4.6953056 | 1.903706 | 7.486906 | 1 |
| Jiang Yuqin 2025 | CT VS CT+MOXI | 11.930001 | 8.868137 | 14.99187 | 1 |
| Jiang Yuqin 2025 | CT VS MOXI | 5.2477421 | 3.711663 | 6.783822 | 1 |
| Jiang Yuqin 2025 | CT VS REHAB | 3.0168189 | -0.8571588 | 6.890797 | 1 |
| Jiang Yuqin 2025 | CT VS TCM | -0.53942394 | -2.973349 | 1.894502 | 1 |
| Jiang Yuqin 2025 | CT VS TCM+MOXI | 3.7070982 | 2.200314 | 5.213882 | 1 |
| Jiang Yuqin 2025 | CT VS TUINA | 3.7628518 | -0.0783413 | 7.604045 | 1 |
| Jiang Yuqin 2025 | CT VS TUINA+MOXI | 7.8005176 | 4.376252 | 11.22478 | 1 |
| Yin Zhao 2023 | CT VS ACU | 0.98425989 | -1.185063 | 3.153583 | 1 |
| Yin Zhao 2023 | CT VS ACU+MOXI | 4.259839 | 1.563947 | 6.95573 | 1 |
| Yin Zhao 2023 | CT VS CT+MOXI | 11.930001 | 8.7936 | 15.0664 | 1 |
| Yin Zhao 2023 | CT VS MOXI | 5.2501634 | 3.682489 | 6.817838 | 1 |
| Yin Zhao 2023 | CT VS REHAB | 3.0152085 | -0.9490557 | 6.979473 | 1 |
| Yin Zhao 2023 | CT VS TCM | -0.61386358 | -3.405463 | 2.177735 | 1 |
| Yin Zhao 2023 | CT VS TCM+MOXI | 3.7115522 | 2.173014 | 5.25009 | 1 |
| Yin Zhao 2023 | CT VS TUINA | 3.762594 | -0.1695232 | 7.694711 | 1 |
| Yin Zhao 2023 | CT VS TUINA+MOXI | 7.8016033 | 4.29741 | 11.3058 | 1 |
| Chen Jing 2016 | CT VS ACU | 0.98429665 | -1.199494 | 3.168088 | 1 |
| Chen Jing 2016 | CT VS ACU+MOXI | 4.261067 | 1.547324 | 6.97481 | 1 |
| Chen Jing 2016 | CT VS CT+MOXI | 11.930001 | 8.76765 | 15.09235 | 1 |
| Chen Jing 2016 | CT VS MOXI | 5.2538383 | 3.674718 | 6.832958 | 1 |
| Chen Jing 2016 | CT VS REHAB | 3.0289525 | -0.9705392 | 7.028444 | 1 |
| Chen Jing 2016 | CT VS TCM | -0.53276275 | -3.035656 | 1.970131 | 1 |
| Chen Jing 2016 | CT VS TCM+MOXI | 3.7134767 | 2.163862 | 5.263092 | 1 |
| Chen Jing 2016 | CT VS TUINA | 3.7067296 | -0.4700769 | 7.883536 | 1 |
| Chen Jing 2016 | CT VS TUINA+MOXI | 7.8162498 | 4.279826 | 11.35267 | 1 |
| Wang Hongfei 2020 | CT VS ACU | 0.99790441 | -1.03356 | 3.029369 | 1 |
| Wang Hongfei 2020 | CT VS ACU+MOXI | 4.2607436 | 1.73481 | 6.786677 | 1 |
| Wang Hongfei 2020 | CT VS CT+MOXI | 11.930001 | 9.042767 | 14.81724 | 1 |
| Wang Hongfei 2020 | CT VS MOXI | 5.2412067 | 3.778941 | 6.703473 | 1 |
| Wang Hongfei 2020 | CT VS REHAB | 4.0205807 | 0.1792907 | 7.861871 | 1 |
| Wang Hongfei 2020 | CT VS TCM | -0.55236563 | -2.867141 | 1.76241 | 1 |
| Wang Hongfei 2020 | CT VS TCM+MOXI | 3.6946927 | 2.261866 | 5.12752 | 1 |
| Wang Hongfei 2020 | CT VS TUINA | 3.7634548 | 0.1350124 | 7.391897 | 1 |
| Wang Hongfei 2020 | CT VS TUINA+MOXI | 7.7976221 | 4.560207 | 11.03504 | 1 |
| Zhao Huayun 2023 | CT VS ACU | 0.9920006 | -1.100965 | 3.084966 | 1 |
| Zhao Huayun 2023 | CT VS ACU+MOXI | 4.2608452 | 1.659115 | 6.862576 | 1 |
| Zhao Huayun 2023 | CT VS CT+MOXI | 11.930001 | 8.931324 | 14.92868 | 1 |
| Zhao Huayun 2023 | CT VS MOXI | 5.245966 | 3.73657 | 6.755362 | 1 |
| Zhao Huayun 2023 | CT VS REHAB | 2.1834951 | -1.827769 | 6.194759 | 1 |
| Zhao Huayun 2023 | CT VS TCM | -0.54382701 | -2.934525 | 1.846871 | 1 |
| Zhao Huayun 2023 | CT VS TCM+MOXI | 3.7028824 | 2.222944 | 5.182821 | 1 |
| Zhao Huayun 2023 | CT VS TUINA | 3.765858 | 0.0009989 | 7.530717 | 1 |
| Zhao Huayun 2023 | CT VS TUINA+MOXI | 7.8023198 | 4.444942 | 11.1597 | 1 |
| Li Xiuxia 2019 | CT VS ACU | 0.76203519 | -1.376291 | 2.900361 | 1 |
| Li Xiuxia 2019 | CT VS ACU+MOXI | 4.0318667 | 1.389814 | 6.673919 | 1 |
| Li Xiuxia 2019 | CT VS CT+MOXI | 11.930001 | 8.912614 | 14.94739 | 1 |
| Li Xiuxia 2019 | CT VS MOXI | 4.7970045 | 3.107381 | 6.486628 | 1 |
| Li Xiuxia 2019 | CT VS REHAB | 2.5706902 | -1.320751 | 6.462132 | 1 |
| Li Xiuxia 2019 | CT VS TCM | -0.61815091 | -3.024532 | 1.78823 | 1 |
| Li Xiuxia 2019 | CT VS TCM+MOXI | 3.6284938 | 2.135914 | 5.121073 | 1 |
| Li Xiuxia 2019 | CT VS TUINA | 3.3160219 | -0.5426921 | 7.174736 | 1 |
| Li Xiuxia 2019 | CT VS TUINA+MOXI | 7.3526153 | 3.895764 | 10.80947 | 1 |
| Li Ju 2024 | _ | _ | _ | _ | disconnected |
| Xiong Qi 2024 | CT VS ACU | 0.98399384 | -1.190975 | 3.158963 | 1 |
| Xiong Qi 2024 | CT VS ACU+MOXI | 4.2600418 | 1.557181 | 6.962903 | 1 |
| Xiong Qi 2024 | CT VS CT+MOXI | 11.930001 | 8.783446 | 15.07656 | 1 |
| Xiong Qi 2024 | CT VS MOXI | 5.2511003 | 3.679021 | 6.823179 | 1 |
| Xiong Qi 2024 | CT VS REHAB | 2.804061 | -1.391705 | 6.999827 | 1 |
| Xiong Qi 2024 | CT VS TCM | -0.53409286 | -3.026112 | 1.957926 | 1 |
| Xiong Qi 2024 | CT VS TCM+MOXI | 3.7121898 | 2.169343 | 5.255037 | 1 |
| Xiong Qi 2024 | CT VS TUINA | 3.7656388 | -0.1797287 | 7.711006 | 1 |
| Xiong Qi 2024 | CT VS TUINA+MOXI | 7.8048267 | 4.288759 | 11.32089 | 1 |

**Sensitivity analysis based on the cure rate group in the primary analysis of patients with LDH. (Table S53)**

| **dropped_id** | **comparison** | **log_eff** | **log_lci** | **log_uci** | **OR** | **OR_lci** | **OR_uci** | **connected** |
| --- | --- | --- | --- | --- | --- | --- | --- | --- |
| Chunyue Cai 2018 | CT VS ACU | -0.04833789 | -0.6547628 | 0.5580871 | 0.95281179 | 0.5195653 | 1.747327 | 1 |
| Chunyue Cai 2018 | CT VS ACU+MOXI | 0.24444371 | -0.4056303 | 0.8945177 | 1.2769108 | 0.6665565 | 2.446156 | 1 |
| Chunyue Cai 2018 | CT VS CT+MOXI | 0.55412693 | 0.2132093 | 0.8950445 | 1.7404208 | 1.237644 | 2.447445 | 1 |
| Chunyue Cai 2018 | CT VS MOXI | 0.44813329 | 0.1038223 | 0.7924443 | 1.5653873 | 1.109403 | 2.208789 | 1 |
| Chunyue Cai 2018 | CT VS TCM | 0.26833602 | -0.2217194 | 0.7583914 | 1.3077865 | 0.8011401 | 2.134839 | 1 |
| Chunyue Cai 2018 | CT VS TCM+MOXI | 0.51965725 | 0.2269439 | 0.8123706 | 1.6814512 | 1.254759 | 2.253243 | 1 |
| Chunyue Cai 2018 | CT VS TUINA | 0.44784707 | -0.1046663 | 1.00036 | 1.5649393 | 0.9006251 | 2.719262 | 1 |
| Chunyue Cai 2018 | CT VS TUINA+MOXI | 0.90007626 | 0.4445101 | 1.355642 | 2.4597907 | 1.559726 | 3.879252 | 1 |
| Hu Qinqin 2024 | CT VS ACU | -0.21931358 | -0.7798656 | 0.3412384 | 0.80306985 | 0.4584677 | 1.406689 | 1 |
| Hu Qinqin 2024 | CT VS ACU+MOXI | 0.07347535 | -0.5340344 | 0.6809851 | 1.076242 | 0.5862351 | 1.975823 | 1 |
| Hu Qinqin 2024 | CT VS CT+MOXI | 0.55141535 | 0.2105175 | 0.8923132 | 1.7357079 | 1.234317 | 2.440769 | 1 |
| Hu Qinqin 2024 | CT VS MOXI | 0.39303348 | 0.0568931 | 0.7291739 | 1.481468 | 1.058543 | 2.073367 | 1 |
| Hu Qinqin 2024 | CT VS TCM | 0.28078414 | -0.2270675 | 0.7886358 | 1.3241677 | 0.796867 | 2.200392 | 1 |
| Hu Qinqin 2024 | CT VS TCM+MOXI | 0.53210559 | 0.2104836 | 0.8537276 | 1.7025133 | 1.234275 | 2.348384 | 1 |
| Hu Qinqin 2024 | CT VS TUINA | 0.44278942 | -0.1096815 | 0.9952604 | 1.5570444 | 0.8961195 | 2.705429 | 1 |
| Hu Qinqin 2024 | CT VS TUINA+MOXI | 0.89501854 | 0.4395038 | 1.350533 | 2.4473812 | 1.551937 | 3.859483 | 1 |
| Ding Wenjie 2025 | CT VS ACU | -0.17988777 | -0.7438588 | 0.3840833 | 0.83536396 | 0.4752764 | 1.468268 | 1 |
| Ding Wenjie 2025 | CT VS ACU+MOXI | 0.11289948 | -0.4977663 | 0.7235652 | 1.1195194 | 0.607887 | 2.061771 | 1 |
| Ding Wenjie 2025 | CT VS CT+MOXI | 0.51073508 | 0.1615384 | 0.8599318 | 1.6665158 | 1.175318 | 2.362999 | 1 |
| Ding Wenjie 2025 | CT VS MOXI | 0.43387669 | 0.0898778 | 0.7778755 | 1.5432286 | 1.094041 | 2.176843 | 1 |
| Ding Wenjie 2025 | CT VS TCM | 0.30865256 | -0.1784092 | 0.7957143 | 1.3615892 | 0.8366001 | 2.216023 | 1 |
| Ding Wenjie 2025 | CT VS TCM+MOXI | 0.55997448 | 0.2723013 | 0.8476477 | 1.7506278 | 1.312983 | 2.33415 | 1 |
| Ding Wenjie 2025 | CT VS TUINA | 0.36691273 | -0.2033049 | 0.9371303 | 1.443272 | 0.8160294 | 2.552646 | 1 |
| Ding Wenjie 2025 | CT VS TUINA+MOXI | 0.81914077 | 0.3422569 | 1.296025 | 2.2685498 | 1.408122 | 3.654739 | 1 |
| Zhong Qunhua 2024 | CT VS ACU | -0.18007737 | -0.7440366 | 0.3838818 | 0.83520559 | 0.4751919 | 1.467972 | 1 |
| Zhong Qunhua 2024 | CT VS ACU+MOXI | 0.11270988 | -0.4979449 | 0.7233647 | 1.1193072 | 0.6077784 | 2.061357 | 1 |
| Zhong Qunhua 2024 | CT VS CT+MOXI | 1.3739233 | -0.1968192 | 2.944666 | 3.9508207 | 0.8213391 | 19.00431 | 1 |
| Zhong Qunhua 2024 | CT VS MOXI | 0.4336649 | 0.0896903 | 0.7776395 | 1.5429018 | 1.093835 | 2.176329 | 1 |
| Zhong Qunhua 2024 | CT VS TCM | 0.30864496 | -0.1784168 | 0.7957067 | 1.3615789 | 0.8365937 | 2.216007 | 1 |
| Zhong Qunhua 2024 | CT VS TCM+MOXI | 0.55996687 | 0.2722937 | 0.84764 | 1.7506145 | 1.312973 | 2.334132 | 1 |
| Zhong Qunhua 2024 | CT VS TUINA | 1.2301814 | -0.3380867 | 2.79845 | 3.4218501 | 0.7131335 | 16.41917 | 1 |
| Zhong Qunhua 2024 | CT VS TUINA+MOXI | 1.6824217 | 0.1456083 | 3.219235 | 5.3785655 | 1.156743 | 25.00899 | 1 |
| Liu Wenlin 2024 | CT VS ACU | -0.2153066 | -0.7754763 | 0.3448631 | 0.80629419 | 0.4604844 | 1.411797 | 1 |
| Liu Wenlin 2024 | CT VS ACU+MOXI | 0.11457147 | -0.5317382 | 0.7608811 | 1.1213928 | 0.5875828 | 2.140161 | 1 |
| Liu Wenlin 2024 | CT VS CT+MOXI | 0.5514789 | 0.2105812 | 0.8923766 | 1.7358182 | 1.234395 | 2.440924 | 1 |
| Liu Wenlin 2024 | CT VS MOXI | 0.3943248 | 0.0582506 | 0.7303991 | 1.4833823 | 1.059981 | 2.075909 | 1 |
| Liu Wenlin 2024 | CT VS TCM | 0.30723199 | -0.1798227 | 0.7942867 | 1.3596564 | 0.8354183 | 2.212862 | 1 |
| Liu Wenlin 2024 | CT VS TCM+MOXI | 0.55855388 | 0.2708926 | 0.8462151 | 1.7481426 | 1.311134 | 2.330808 | 1 |
| Liu Wenlin 2024 | CT VS TUINA | 0.44290796 | -0.1095626 | 0.9953786 | 1.557229 | 0.896226 | 2.705748 | 1 |
| Liu Wenlin 2024 | CT VS TUINA+MOXI | 0.89513708 | 0.4396228 | 1.350651 | 2.4476713 | 1.552122 | 3.859939 | 1 |
| Wu Yusheng 2025 | CT VS ACU | -0.20539719 | -0.7662477 | 0.3554533 | 0.81432381 | 0.4647537 | 1.426827 | 1 |
| Wu Yusheng 2025 | CT VS ACU+MOXI | 0.08739115 | -0.520394 | 0.6951764 | 1.0913235 | 0.5942863 | 2.004062 | 1 |
| Wu Yusheng 2025 | CT VS CT+MOXI | 0.55163605 | 0.2107381 | 0.892534 | 1.736091 | 1.234589 | 2.441308 | 1 |
| Wu Yusheng 2025 | CT VS MOXI | 0.39751827 | 0.0613261 | 0.7337104 | 1.488127 | 1.063246 | 2.082794 | 1 |
| Wu Yusheng 2025 | CT VS TCM | 0.3722956 | -0.1496142 | 0.8942055 | 1.4510618 | 0.8610401 | 2.445392 | 1 |
| Wu Yusheng 2025 | CT VS TCM+MOXI | 0.62361859 | 0.280227 | 0.9670102 | 1.8656669 | 1.32343 | 2.630069 | 1 |
| Wu Yusheng 2025 | CT VS TUINA | 0.44320109 | -0.1092701 | 0.9956723 | 1.5576855 | 0.8964882 | 2.706543 | 1 |
| Wu Yusheng 2025 | CT VS TUINA+MOXI | 0.89543021 | 0.4399152 | 1.350945 | 2.4483889 | 1.552575 | 3.861073 | 1 |
| Yao Dong 2024 | CT VS ACU | -0.17998511 | -0.7439499 | 0.3839797 | 0.83528265 | 0.475233 | 1.468116 | 1 |
| Yao Dong 2024 | CT VS ACU+MOXI | 0.11280214 | -0.4978578 | 0.7234621 | 1.1194104 | 0.6078314 | 2.061558 | 1 |
| Yao Dong 2024 | CT VS CT+MOXI | 0.51082562 | 0.1616043 | 0.860047 | 1.6666667 | 1.175395 | 2.363272 | 1 |
| Yao Dong 2024 | CT VS MOXI | 0.43376796 | 0.0897818 | 0.7777541 | 1.5430608 | 1.093936 | 2.176578 | 1 |
| Yao Dong 2024 | CT VS TCM | 0.30864866 | -0.1784131 | 0.7957104 | 1.3615839 | 0.8365968 | 2.216015 | 1 |
| Yao Dong 2024 | CT VS TCM+MOXI | 0.55997057 | 0.2722974 | 0.8476437 | 1.750621 | 1.312977 | 2.334141 | 1 |
| Yao Dong 2024 | CT VS TUINA | 1.232244 | -0.3370349 | 2.801523 | 3.4289152 | 0.7138839 | 16.46971 | 1 |
| Yao Dong 2024 | CT VS TUINA+MOXI | 1.6844843 | 0.1466394 | 3.222329 | 5.3896709 | 1.157936 | 25.08648 | 1 |
| Guo Hui 2023 | CT VS ACU | -0.21528985 | -0.7754149 | 0.3448352 | 0.8063077 | 0.4605127 | 1.411757 | 1 |
| Guo Hui 2023 | CT VS ACU+MOXI | 0.07749891 | -0.529617 | 0.6846148 | 1.0805811 | 0.5888305 | 1.983008 | 1 |
| Guo Hui 2023 | CT VS CT+MOXI | 0.55147916 | 0.2105815 | 0.8923768 | 1.7358187 | 1.234396 | 2.440924 | 1 |
| Guo Hui 2023 | CT VS MOXI | 0.3943302 | 0.0582637 | 0.7303967 | 1.4833903 | 1.059994 | 2.075904 | 1 |
| Guo Hui 2023 | CT VS TCM | 0.55856534 | 0.2709007 | 0.84623 | 1.7481627 | 1.311145 | 2.330843 | 1 |
| Guo Hui 2023 | CT VS TCM+MOXI | 0.44290846 | -0.1095621 | 0.995379 | 1.5572298 | 0.8962265 | 2.70575 | 1 |
| Guo Hui 2023 | CT VS TUINA | 0.89513757 | 0.4396233 | 1.350652 | 2.4476725 | 1.552122 | 3.859941 | 1 |
| Wen Qi 2023 | CT VS ACU | -1.1825543 | -2.642326 | 0.2772172 | 0.30649486 | 0.0711955 | 1.319453 | 1 |
| Wen Qi 2023 | CT VS ACU+MOXI | -0.8897241 | -2.368111 | 0.5886624 | 0.41076907 | 0.0936575 | 1.801577 | 1 |
| Wen Qi 2023 | CT VS CT+MOXI | 0.55412793 | 0.2132103 | 0.8950456 | 1.7404226 | 1.237645 | 2.447447 | 1 |
| Wen Qi 2023 | CT VS MOXI | 0.448154 | 0.1038148 | 0.7924932 | 1.5654198 | 1.109395 | 2.208897 | 1 |
| Wen Qi 2023 | CT VS TCM | 0.26843778 | -0.2216085 | 0.7584841 | 1.3079196 | 0.8012289 | 2.135037 | 1 |
| Wen Qi 2023 | CT VS TCM+MOXI | 0.51975901 | 0.2270608 | 0.8124572 | 1.6816223 | 1.254906 | 2.253438 | 1 |
| Wen Qi 2023 | CT VS TUINA | 0.44784892 | -0.1046646 | 1.000362 | 1.5649422 | 0.9006266 | 2.719267 | 1 |
| Wen Qi 2023 | CT VS TUINA+MOXI | 0.90007811 | 0.4445118 | 1.355644 | 2.4597952 | 1.559729 | 3.87926 | 1 |
| Wang Guoshu 2017 | CT VS ACU | -0.21530543 | -0.7754798 | 0.3448689 | 0.80629514 | 0.4604828 | 1.411805 | 1 |
| Wang Guoshu 2017 | CT VS ACU+MOXI | 0.11513808 | -0.5267355 | 0.7570117 | 1.1220284 | 0.5905296 | 2.131896 | 1 |
| Wang Guoshu 2017 | CT VS CT+MOXI | 0.55147891 | 0.2105812 | 0.8923766 | 1.7358182 | 1.234395 | 2.440924 | 1 |
| Wang Guoshu 2017 | CT VS MOXI | 0.39432518 | 0.0582501 | 0.7304002 | 1.4833828 | 1.05998 | 2.075911 | 1 |
| Wang Guoshu 2017 | CT VS TCM | 0.30723204 | -0.1798227 | 0.7942867 | 1.3596564 | 0.8354183 | 2.212862 | 1 |
| Wang Guoshu 2017 | CT VS TCM+MOXI | 0.55855393 | 0.2708927 | 0.8462152 | 1.7481427 | 1.311134 | 2.330808 | 1 |
| Wang Guoshu 2017 | CT VS TUINA | 0.44290799 | -0.1095626 | 0.9953786 | 1.5572291 | 0.896226 | 2.705749 | 1 |
| Wang Guoshu 2017 | CT VS TUINA+MOXI | 0.89513711 | 0.4396228 | 1.350651 | 2.4476714 | 1.552122 | 3.859939 | 1 |
| Sun Jian 2009 | CT VS ACU | -0.21528427 | -0.7754167 | 0.3448481 | 0.8063122 | 0.4605119 | 1.411775 | 1 |
| Sun Jian 2009 | CT VS ACU+MOXI | 0.0330422 | -0.5783467 | 0.6444311 | 1.0335942 | 0.5608248 | 1.904903 | 1 |
| Sun Jian 2009 | CT VS CT+MOXI | 0.55147925 | 0.2105816 | 0.8923769 | 1.7358188 | 1.234396 | 2.440925 | 1 |
| Sun Jian 2009 | CT VS MOXI | 0.39433199 | 0.0582642 | 0.7303998 | 1.4833929 | 1.059995 | 2.07591 | 1 |
| Sun Jian 2009 | CT VS TCM | 0.30723289 | -0.1798217 | 0.7942875 | 1.3596576 | 0.8354191 | 2.212864 | 1 |
| Sun Jian 2009 | CT VS TCM+MOXI | 0.55855478 | 0.2708937 | 0.8462159 | 1.7481442 | 1.311136 | 2.33081 | 1 |
| Sun Jian 2009 | CT VS TUINA | 0.44290862 | -0.1095619 | 0.9953792 | 1.55723 | 0.8962266 | 2.70575 | 1 |
| Sun Jian 2009 | CT VS TUINA+MOXI | 0.89513774 | 0.4396235 | 1.350652 | 2.4476729 | 1.552123 | 3.859941 | 1 |
| Sun Jianbing 2025 | CT VS ACU | -0.2115636 | -0.7731861 | 0.3500589 | 0.8093178 | 0.4615402 | 1.419151 | 1 |
| Sun Jianbing 2025 | CT VS ACU+MOXI | 0.081225 | -0.5272726 | 0.6897225 | 1.0846149 | 0.5902125 | 1.993163 | 1 |
| Sun Jianbing 2025 | CT VS CT+MOXI | 0.55153826 | 0.21064 | 0.8924366 | 1.7359213 | 1.234468 | 2.44107 | 1 |
| Sun Jianbing 2025 | CT VS MOXI | 0.39553104 | 0.0592051 | 0.731857 | 1.4851727 | 1.060993 | 2.078938 | 1 |
| Sun Jianbing 2025 | CT VS TCM | 0.33174649 | -0.224903 | 0.888396 | 1.3933996 | 0.7985936 | 2.431227 | 1 |
| Sun Jianbing 2025 | CT VS TCM+MOXI | 0.5830688 | 0.1888761 | 0.9772615 | 1.7915278 | 1.207891 | 2.65717 | 1 |
| Sun Jianbing 2025 | CT VS TUINA | 0.44301868 | -0.1094532 | 0.9954906 | 1.5574014 | 0.8963241 | 2.706052 | 1 |
| Sun Jianbing 2025 | CT VS TUINA+MOXI | 0.8952478 | 0.4397319 | 1.350764 | 2.4479423 | 1.552291 | 3.860373 | 1 |
| Xu Chenghua 2018 | CT VS ACU | -0.2152891 | -0.7754141 | 0.3448359 | 0.80630831 | 0.460513 | 1.411758 | 1 |
| Xu Chenghua 2018 | CT VS ACU+MOXI | 0.07749967 | -0.5296162 | 0.6846156 | 1.0805819 | 0.5888309 | 1.983009 | 1 |
| Xu Chenghua 2018 | CT VS CT+MOXI | 0.55150792 | 0.2106032 | 0.8924127 | 1.7358686 | 1.234422 | 2.441012 | 1 |
| Xu Chenghua 2018 | CT VS MOXI | 0.39433235 | 0.0582658 | 0.7303989 | 1.4833935 | 1.059997 | 2.075909 | 1 |
| Xu Chenghua 2018 | CT VS TCM | 0.30723269 | -0.1798219 | 0.7942873 | 1.3596573 | 0.835419 | 2.212863 | 1 |
| Xu Chenghua 2018 | CT VS TCM+MOXI | 0.55855458 | 0.2708935 | 0.8462157 | 1.7481439 | 1.311135 | 2.33081 | 1 |
| Xu Chenghua 2018 | CT VS TUINA | 0.5437831 | -0.0941889 | 1.181755 | 1.722511 | 0.9101109 | 3.260091 | 1 |
| Xu Chenghua 2018 | CT VS TUINA+MOXI | 0.89519122 | 0.4396585 | 1.350724 | 2.4478038 | 1.552177 | 3.860219 | 1 |
| Wu Zongxiang 2017 | CT VS ACU | -0.21529008 | -0.7754151 | 0.3448349 | 0.80630751 | 0.4605126 | 1.411757 | 1 |
| Wu Zongxiang 2017 | CT VS ACU+MOXI | 0.07749867 | -0.5296172 | 0.6846145 | 1.0805808 | 0.5888304 | 1.983007 | 1 |
| Wu Zongxiang 2017 | CT VS CT+MOXI | 0.55149547 | 0.2105942 | 0.8923967 | 1.735847 | 1.234411 | 2.440973 | 1 |
| Wu Zongxiang 2017 | CT VS MOXI | 0.39433123 | 0.0582647 | 0.7303978 | 1.4833918 | 1.059996 | 2.075906 | 1 |
| Wu Zongxiang 2017 | CT VS TCM | 0.30723264 | -0.179822 | 0.7942873 | 1.3596572 | 0.8354189 | 2.212863 | 1 |
| Wu Zongxiang 2017 | CT VS TCM+MOXI | 0.55855453 | 0.2708934 | 0.8462157 | 1.7481438 | 1.311135 | 2.33081 | 1 |
| Wu Zongxiang 2017 | CT VS TUINA | 0.34611152 | -0.2856298 | 0.9778529 | 1.4135602 | 0.7515408 | 2.658741 | 1 |
| Wu Zongxiang 2017 | CT VS TUINA+MOXI | 0.89516799 | 0.4396443 | 1.350692 | 2.4477469 | 1.552155 | 3.860095 | 1 |
| Li Xiuxia 2019 | CT VS ACU | -1.0716013 | -2.165167 | 0.0219643 | 0.34245969 | 0.1147308 | 1.022207 | 1 |
| Li Xiuxia 2019 | CT VS ACU+MOXI | -0.77877592 | -1.897105 | 0.3395531 | 0.45896748 | 0.1500023 | 1.40432 | 1 |
| Li Xiuxia 2019 | CT VS CT+MOXI | 0.50440649 | 0.1596211 | 0.8491918 | 1.6560024 | 1.173066 | 2.337757 | 1 |
| Li Xiuxia 2019 | CT VS MOXI | -0.562194 | -1.663849 | 0.5394607 | 0.5699572 | 0.1894086 | 1.715082 | 1 |
| Li Xiuxia 2019 | CT VS TCM | 0.27288785 | -0.2156214 | 0.7613971 | 1.3137529 | 0.8060405 | 2.141266 | 1 |
| Li Xiuxia 2019 | CT VS TCM+MOXI | 0.52420916 | 0.2340919 | 0.8143264 | 1.6891225 | 1.263761 | 2.257654 | 1 |
| Li Xiuxia 2019 | CT VS TUINA | 0.35510867 | -0.2056923 | 0.9159096 | 1.4263357 | 0.8140836 | 2.499047 | 1 |
| Li Xiuxia 2019 | CT VS TUINA+MOXI | 0.80733654 | 0.3417536 | 1.27292 | 2.2419287 | 1.407413 | 3.571264 | 1 |

Sensitivity Analysis(Subgroup Analysis)

**Sensitivity analysis based on the ODI score group in the subgroup analysis of patients with LDH (Table S54)**

| **dropped_id** | **comparison** | **eff** | **lci** | **uci** | **connected** |
| --- | --- | --- | --- | --- | --- |
| Wang Liya 2024 | CT VS ACU | 0.74973541 | -4.020382 | 5.519853 | 1 |
| Wang Liya 2024 | CT VS CM | -4.9406363 | -7.366438 | -2.514835 | 1 |
| Wang Liya 2024 | CT VS HM | -7.2560116 | -10.93466 | -3.577361 | 1 |
| Wang Liya 2024 | CT VS TCM | 0.84260175 | -4.982378 | 6.667582 | 1 |
| Wang Liya 2024 | CT VS WN | -4.6240563 | -10.65293 | 1.404818 | 1 |
| Hu Qinqin 2024 | CT VS ACU | 1.5486302 | -3.525369 | 6.62263 | 1 |
| Hu Qinqin 2024 | CT VS CM | -4.1109647 | -6.700248 | -1.521681 | 1 |
| Hu Qinqin 2024 | CT VS HM | -6.7114093 | -10.6455 | -2.777322 | 1 |
| Hu Qinqin 2024 | CT VS TCM | 1.4752541 | -4.757641 | 7.708149 | 1 |
| Hu Qinqin 2024 | CT VS WN | -3.9075816 | -10.35793 | 2.542769 | 1 |
| Xu Fen 2025 | CT VS ACU | 0.32225084 | -4.165902 | 4.810404 | 1 |
| Xu Fen 2025 | CT VS CM | -4.9952899 | -7.163875 | -2.826705 | 1 |
| Xu Fen 2025 | CT VS HM | -9.3320127 | -13.64829 | -5.015732 | 1 |
| Xu Fen 2025 | CT VS TCM | -0.65761856 | -6.413809 | 5.098572 | 1 |
| Xu Fen 2025 | CT VS WN | -5.5802451 | -11.32805 | 0.1675602 | 1 |
| Liu Wenlin 2024 | CT VS ACU | 0.334434 | -5.610422 | 6.27929 | 1 |
| Liu Wenlin 2024 | CT VS CM | -4.3398086 | -6.706581 | -1.973036 | 1 |
| Liu Wenlin 2024 | CT VS HM | -6.9678242 | -10.81239 | -3.123255 | 1 |
| Liu Wenlin 2024 | CT VS TCM | 0.88861321 | -5.371873 | 7.1491 | 1 |
| Liu Wenlin 2024 | CT VS WN | -4.8106762 | -11.54965 | 1.928297 | 1 |
| Yao Dong 2024 | CT VS ACU | 1.3334736 | -3.380302 | 6.04725 | 1 |
| Yao Dong 2024 | CT VS CM | -4.5508168 | -6.822275 | -2.279359 | 1 |
| Yao Dong 2024 | CT VS HM | -5.7278091 | -9.861805 | -1.593813 | 1 |
| Yao Dong 2024 | CT VS TCM | 2.0423661 | -3.918209 | 8.002941 | 1 |
| Yao Dong 2024 | CT VS WN | -3.7365047 | -9.78819 | 2.315181 | 1 |
| Chen Weiyi 2021 | CT VS ACU | 1.3294671 | -3.655102 | 6.314036 | 1 |
| Chen Weiyi 2021 | CT VS CM | -4.4262698 | -6.844918 | -2.007622 | 1 |
| Chen Weiyi 2021 | CT VS HM | -6.4497163 | -10.8356 | -2.063828 | 1 |
| Chen Weiyi 2021 | CT VS TCM | 1.5709206 | -4.7599 | 7.901742 | 1 |
| Chen Weiyi 2021 | CT VS WN | -3.9709124 | -10.39949 | 2.457669 | 1 |
| Deng Min 2022 | CT VS ACU | 1.3646287 | -3.786017 | 6.515274 | 1 |
| Deng Min 2022 | CT VS CM | -4.3092788 | -6.951448 | -1.66711 | 1 |
| Deng Min 2022 | CT VS HM | -6.8482941 | -10.84594 | -2.850648 | 1 |
| Deng Min 2022 | CT VS TCM | 1.3223927 | -5.007958 | 7.652743 | 1 |
| Deng Min 2022 | CT VS WN | -4.07599 | -10.62754 | 2.475563 | 1 |
| Guo Hui 2023 | CT VS ACU | 1.2815341 | -4.165071 | 6.728139 | 1 |
| Guo Hui 2023 | CT VS CM | -4.3569857 | -6.788841 | -1.92513 | 1 |
| Guo Hui 2023 | CT VS HM | -6.8585152 | -10.9109 | -2.80613 | 1 |
| Guo Hui 2023 | CT VS TCM | 1.125517 | -9.246582 | 11.49762 | 1 |
| Guo Hui 2023 | CT VS WN | -4.2153013 | -12.47201 | 4.041412 | 1 |
| Zhang Jing 2026 | CT VS ACU | 1.2804768 | -4.168874 | 6.729828 | 1 |
| Zhang Jing 2026 | CT VS CM | -4.3570794 | -6.789392 | -1.924766 | 1 |
| Zhang Jing 2026 | CT VS HM | -6.8580787 | -10.91053 | -2.805624 | 1 |
| Zhang Jing 2026 | CT VS TCM | 1.372691 | -6.216215 | 8.961597 | 1 |
| Zhang Jing 2026 | CT VS WN | -4.2179899 | -12.48332 | 4.047339 | 1 |
| Jiang Yunqing 2023 | CT VS ACU | 1.0164044 | -4.005248 | 6.038056 | 1 |
| Jiang Yunqing 2023 | CT VS CM | -4.6716983 | -7.231997 | -2.111399 | 1 |
| Jiang Yunqing 2023 | CT VS HM | -7.0879756 | -10.97925 | -3.1967 | 1 |
| Jiang Yunqing 2023 | CT VS TCM | 1.045314 | -5.118812 | 7.20944 | 1 |
| Jiang Yunqing 2023 | CT VS WN | -4.3888708 | -10.76787 | 1.990124 | 1 |
| Wang Jianying 2025 | CT VS ACU | 2.2134231 | -1.48597 | 5.912817 | 1 |
| Wang Jianying 2025 | CT VS CM | -3.3021053 | -5.112164 | -1.492047 | 1 |
| Wang Jianying 2025 | CT VS HM | -6.0830945 | -8.864219 | -3.30197 | 1 |
| Wang Jianying 2025 | CT VS TCM | 2.1138537 | -2.284539 | 6.512247 | 1 |
| Wang Jianying 2025 | CT VS WN | -3.2568257 | -7.806276 | 1.292625 | 1 |
| Gao Yuanguanyu 2021 | CT VS ACU | 1.5301207 | -3.572214 | 6.632456 | 1 |
| Gao Yuanguanyu 2021 | CT VS CM | -4.1317379 | -6.746278 | -1.517198 | 1 |
| Gao Yuanguanyu 2021 | CT VS HM | -6.7263617 | -10.68364 | -2.769085 | 1 |
| Gao Yuanguanyu 2021 | CT VS TCM | 1.459123 | -4.807365 | 7.725611 | 1 |
| Gao Yuanguanyu 2021 | CT VS WN | -3.9248863 | -10.41036 | 2.560591 | 1 |
| Xu Mengxia 2019 | CT VS ACU | 1.2782881 | -4.17554 | 6.732116 | 1 |
| Xu Mengxia 2019 | CT VS CM | -4.3574065 | -6.790137 | -1.924676 | 1 |
| Xu Mengxia 2019 | CT VS HM | -6.8578072 | -10.90963 | -2.805979 | 1 |
| Xu Mengxia 2019 | CT VS TCM | 1.3744203 | -6.208909 | 8.957749 | 1 |
| Xu Mengxia 2019 | CT VS WN | -3.9648677 | -13.81045 | 5.880713 | 1 |
| Pan Meng 2025 | CT VS ACU | 2.5607699 | -3.879713 | 9.001253 | 1 |
| Pan Meng 2025 | CT VS CM | -4.3759706 | -6.747069 | -2.004872 | 1 |
| Pan Meng 2025 | CT VS HM | -6.7559842 | -10.61392 | -2.89805 | 1 |
| Pan Meng 2025 | CT VS TCM | 1.7951593 | -4.554569 | 8.144888 | 1 |
| Pan Meng 2025 | CT VS WN | -3.2384416 | -10.20728 | 3.730396 | 1 |

**Sensitivity analysis based on the VAS score group in the subgroup analysis of patients with LDH (Table S55)**

| **dropped_id** | **comparison** | **eff** | **lci** | **Uci** | **connected** |
| --- | --- | --- | --- | --- | --- |
| Chunyue Cai 2018 | CT VS ACU | -0.2720311 | -1.034629 | 0.490567 | 1 |
| Chunyue Cai 2018 | CT VS CM | -1.3306708 | -1.914084 | -0.7472576 | 1 |
| Chunyue Cai 2018 | CT VS HM | -1.274547 | -2.008499 | -0.5405953 | 1 |
| Chunyue Cai 2018 | CT VS REHAB | 0.2076631 | -0.7864532 | 1.201779 | 1 |
| Chunyue Cai 2018 | CT VS TCM | -0.54028642 | -1.997952 | 0.9173791 | 1 |
| Chunyue Cai 2018 | CT VS TF | -3.0458708 | -3.953358 | -2.138383 | 1 |
| Chunyue Cai 2018 | CT VS TUINA | 0.36210814 | -0.821318 | 1.545534 | 1 |
| Chunyue Cai 2018 | CT VS WN | -1.6703664 | -2.448688 | -0.8920451 | 1 |
| Xu Minmin 2016 | CT VS ACU | -0.36163649 | -1.084476 | 0.3612029 | 1 |
| Xu Minmin 2016 | CT VS CM | -1.3396155 | -1.904688 | -0.7745427 | 1 |
| Xu Minmin 2016 | CT VS HM | -1.3250029 | -2.038862 | -0.6111442 | 1 |
| Xu Minmin 2016 | CT VS REHAB | 0.0766985 | -0.8994423 | 1.052839 | 1 |
| Xu Minmin 2016 | CT VS TCM | -0.77167368 | -2.215782 | 0.6724347 | 1 |
| Xu Minmin 2016 | CT VS TF | -3.4275393 | -4.442866 | -2.412213 | 1 |
| Xu Minmin 2016 | CT VS TUINA | 0.32618455 | -0.8235943 | 1.475963 | 1 |
| Xu Minmin 2016 | CT VS WN | -1.7589569 | -2.5152 | -1.002714 | 1 |
| Wang Liya 2024 | CT VS ACU | -0.42000977 | -1.1586 | 0.3185804 | 1 |
| Wang Liya 2024 | CT VS CM | -1.4477753 | -2.046606 | -0.8489445 | 1 |
| Wang Liya 2024 | CT VS HM | -1.3481114 | -2.069335 | -0.6268876 | 1 |
| Wang Liya 2024 | CT VS REHAB | 0.13610995 | -0.836059 | 1.108279 | 1 |
| Wang Liya 2024 | CT VS TCM | -0.5982987 | -2.02134 | 0.824743 | 1 |
| Wang Liya 2024 | CT VS TF | -3.0713333 | -3.957121 | -2.185546 | 1 |
| Wang Liya 2024 | CT VS TUINA | 0.25420726 | -0.9113047 | 1.419719 | 1 |
| Wang Liya 2024 | CT VS WN | -1.7595878 | -2.520634 | -0.998542 | 1 |
| Hu Qinqin 2024 | CT VS ACU | -0.30705258 | -1.066592 | 0.4524871 | 1 |
| Hu Qinqin 2024 | CT VS CM | -1.2968151 | -1.912015 | -0.6816155 | 1 |
| Hu Qinqin 2024 | CT VS HM | -1.276812 | -2.018552 | -0.5350723 | 1 |
| Hu Qinqin 2024 | CT VS REHAB | 0.20046473 | -0.7997637 | 1.200693 | 1 |
| Hu Qinqin 2024 | CT VS TCM | -0.54915565 | -2.01353 | 0.9152188 | 1 |
| Hu Qinqin 2024 | CT VS TF | -3.0489724 | -3.960616 | -2.137329 | 1 |
| Hu Qinqin 2024 | CT VS TUINA | 0.37982553 | -0.8196662 | 1.579317 | 1 |
| Hu Qinqin 2024 | CT VS WN | -1.6847468 | -2.467747 | -0.9017469 | 1 |
| Ding Wenjie 2025 | CT VS ACU | -0.27649689 | -0.9886034 | 0.4356096 | 1 |
| Ding Wenjie 2025 | CT VS CM | -1.2909735 | -1.847568 | -0.7343795 | 1 |
| Ding Wenjie 2025 | CT VS HM | -1.2387995 | -1.942213 | -0.5353855 | 1 |
| Ding Wenjie 2025 | CT VS REHAB | 0.32778424 | -0.6341212 | 1.28969 | 1 |
| Ding Wenjie 2025 | CT VS TCM | -0.29453598 | -1.717808 | 1.128736 | 1 |
| Ding Wenjie 2025 | CT VS TF | -2.5970038 | -3.600033 | -1.593975 | 1 |
| Ding Wenjie 2025 | CT VS TUINA | 0.4055894 | -0.7268381 | 1.538017 | 1 |
| Ding Wenjie 2025 | CT VS WN | -1.61777 | -2.362837 | -0.8727034 | 1 |
| Liu Wenlin 2024 | CT VS ACU | -0.28544832 | -1.047691 | 0.4767938 | 1 |
| Liu Wenlin 2024 | CT VS CM | -1.3265777 | -1.910657 | -0.7424989 | 1 |
| Liu Wenlin 2024 | CT VS HM | -1.2776248 | -2.012544 | -0.5427054 | 1 |
| Liu Wenlin 2024 | CT VS REHAB | 0.2035468 | -0.7919092 | 1.199003 | 1 |
| Liu Wenlin 2024 | CT VS TCM | -0.54437882 | -2.004102 | 0.9153447 | 1 |
| Liu Wenlin 2024 | CT VS TF | -3.0474609 | -3.956263 | -2.138659 | 1 |
| Liu Wenlin 2024 | CT VS TUINA | 0.36267168 | -0.8224944 | 1.547838 | 1 |
| Liu Wenlin 2024 | CT VS WN | -1.676851 | -2.455968 | -0.8977337 | 1 |
| Wu Yusheng 2025 | CT VS ACU | -0.34578572 | -1.101099 | 0.4095281 | 1 |
| Wu Yusheng 2025 | CT VS CM | -1.3331044 | -1.922619 | -0.7435895 | 1 |
| Wu Yusheng 2025 | CT VS HM | -1.3427271 | -2.13834 | -0.5471146 | 1 |
| Wu Yusheng 2025 | CT VS REHAB | 0.16020248 | -0.8492982 | 1.169703 | 1 |
| Wu Yusheng 2025 | CT VS TCM | -0.56971793 | -2.031285 | 0.8918487 | 1 |
| Wu Yusheng 2025 | CT VS TF | -3.060853 | -3.971183 | -2.150523 | 1 |
| Wu Yusheng 2025 | CT VS TUINA | 0.34602839 | -0.8435708 | 1.535628 | 1 |
| Wu Yusheng 2025 | CT VS WN | -1.713644 | -2.49585 | -0.9314382 | 1 |
| Yao Dong 2024 | CT VS ACU | -0.32693113 | -1.065358 | 0.4114959 | 1 |
| Yao Dong 2024 | CT VS CM | -1.3574683 | -1.944525 | -0.770412 | 1 |
| Yao Dong 2024 | CT VS HM | -1.1990222 | -1.959887 | -0.4381576 | 1 |
| Yao Dong 2024 | CT VS REHAB | 0.22931206 | -0.7623366 | 1.220961 | 1 |
| Yao Dong 2024 | CT VS TCM | -0.54964228 | -1.998402 | 0.8991179 | 1 |
| Yao Dong 2024 | CT VS TF | -3.0435398 | -3.946029 | -2.141051 | 1 |
| Yao Dong 2024 | CT VS TUINA | 0.3373736 | -0.8412308 | 1.515978 | 1 |
| Yao Dong 2024 | CT VS WN | -1.6909189 | -2.460422 | -0.9214156 | 1 |
| Chen Weiyi 2021 | CT VS ACU | -0.32676107 | -1.065245 | 0.4117229 | 1 |
| Chen Weiyi 2021 | CT VS CM | -1.3562476 | -1.942951 | -0.7695439 | 1 |
| Chen Weiyi 2021 | CT VS HM | -1.201541 | -1.961112 | -0.4419704 | 1 |
| Chen Weiyi 2021 | CT VS REHAB | 0.2282372 | -0.7633153 | 1.21979 | 1 |
| Chen Weiyi 2021 | CT VS TCM | -0.54980597 | -1.998691 | 0.8990793 | 1 |
| Chen Weiyi 2021 | CT VS TF | -3.0437801 | -3.94634 | -2.141221 | 1 |
| Chen Weiyi 2021 | CT VS TUINA | 0.33815493 | -0.8404714 | 1.516781 | 1 |
| Chen Weiyi 2021 | CT VS WN | -1.6910094 | -2.460578 | -0.9214408 | 1 |
| Liang Yuanyuan 2014 | CT VS ACU | -0.39700429 | -1.1435 | 0.3494911 | 1 |
| Liang Yuanyuan 2014 | CT VS CM | -1.4171564 | -2.021523 | -0.8127893 | 1 |
| Liang Yuanyuan 2014 | CT VS HM | -1.3337503 | -2.062982 | -0.604519 | 1 |
| Liang Yuanyuan 2014 | CT VS REHAB | 0.14941266 | -0.8338398 | 1.132665 | 1 |
| Liang Yuanyuan 2014 | CT VS TCM | -0.58826747 | -2.027762 | 0.8512267 | 1 |
| Liang Yuanyuan 2014 | CT VS TF | -3.0666968 | -3.962789 | -2.170605 | 1 |
| Liang Yuanyuan 2014 | CT VS TUINA | 0.27975981 | -0.8989593 | 1.458479 | 1 |
| Liang Yuanyuan 2014 | CT VS WN | -1.7443825 | -2.513999 | -0.9747665 | 1 |
| Deng Min 2022 | CT VS ACU | -0.31671998 | -1.077505 | 0.4440656 | 1 |
| Deng Min 2022 | CT VS CM | -1.3098064 | -1.926769 | -0.6928436 | 1 |
| Deng Min 2022 | CT VS HM | -1.2830011 | -2.025653 | -0.5403496 | 1 |
| Deng Min 2022 | CT VS REHAB | 0.19506958 | -0.8062242 | 1.196363 | 1 |
| Deng Min 2022 | CT VS TCM | -0.55334948 | -2.019154 | 0.9124551 | 1 |
| Deng Min 2022 | CT VS TF | -3.0508361 | -3.963358 | -2.138314 | 1 |
| Deng Min 2022 | CT VS TUINA | 0.36905785 | -0.8319955 | 1.570111 | 1 |
| Deng Min 2022 | CT VS WN | -1.6911718 | -2.475137 | -0.9072063 | 1 |
| Li Shengqiang 2015 | CT VS ACU | -0.46915981 | -1.201183 | 0.2628636 | 1 |
| Li Shengqiang 2015 | CT VS CM | -1.2761862 | -1.836136 | -0.7162365 | 1 |
| Li Shengqiang 2015 | CT VS HM | -1.3199941 | -2.02511 | -0.6148782 | 1 |
| Li Shengqiang 2015 | CT VS REHAB | 0.14401604 | -0.8106955 | 1.098728 | 1 |
| Li Shengqiang 2015 | CT VS TCM | -0.60116039 | -2.000903 | 0.7985827 | 1 |
| Li Shengqiang 2015 | CT VS TF | -3.070484 | -3.941764 | -2.199204 | 1 |
| Li Shengqiang 2015 | CT VS TUINA | 0.36581003 | -0.7698776 | 1.501498 | 1 |
| Li Shengqiang 2015 | CT VS WN | -1.7660048 | -2.513262 | -1.018748 | 1 |
| Wen Qi 20Xie Wenhui 2023 | CT VS ACU | -0.29991202 | -1.058026 | 0.4582021 | 1 |
| Wen Qi 20Xie Wenhui 2023 | CT VS CM | -1.3084785 | -1.893812 | -0.7231446 | 1 |
| Wen Qi 20Xie Wenhui 2023 | CT VS HM | -1.2817537 | -2.017551 | -0.5459567 | 1 |
| Wen Qi 20Xie Wenhui 2023 | CT VS REHAB | 0.18236998 | -0.8156633 | 1.180403 | 1 |
| Wen Qi 20Xie Wenhui 2023 | CT VS TCM | -0.57119998 | -2.036355 | 0.8939553 | 1 |
| Wen Qi 20Xie Wenhui 2023 | CT VS TF | -3.0568196 | -3.96771 | -2.145929 | 1 |
| Wen Qi 20Xie Wenhui 2023 | CT VS TUINA | 0.36013755 | -0.8275434 | 1.547818 | 1 |
| Wen Qi 20Xie Wenhui 2023 | CT VS WN | -1.7203975 | -2.514877 | -0.9259181 | 1 |
| Lin Mingqin 2021 | CT VS ACU | -0.36840307 | -1.134892 | 0.3980858 | 1 |
| Lin Mingqin 2021 | CT VS CM | -1.3412608 | -1.931637 | -0.7508844 | 1 |
| Lin Mingqin 2021 | CT VS HM | -1.3134018 | -2.055072 | -0.5717314 | 1 |
| Lin Mingqin 2021 | CT VS REHAB | 0.14055656 | -0.874338 | 1.155451 | 1 |
| Lin Mingqin 2021 | CT VS TCM | -0.61163666 | -2.086913 | 0.8636392 | 1 |
| Lin Mingqin 2021 | CT VS TF | -3.0728944 | -3.984846 | -2.160943 | 1 |
| Lin Mingqin 2021 | CT VS TUINA | 0.31699195 | -0.8822007 | 1.516185 | 1 |
| Lin Mingqin 2021 | CT VS WN | -1.7841797 | -2.638393 | -0.929966 | 1 |
| Xie Wenhui 2023 | CT VS ACU | -0.37934785 | -1.129574 | 0.3708782 | 1 |
| Xie Wenhui 2023 | CT VS CM | -1.3566001 | -1.941933 | -0.7712672 | 1 |
| Xie Wenhui 2023 | CT VS HM | -1.4203457 | -2.214979 | -0.6257126 | 1 |
| Xie Wenhui 2023 | CT VS REHAB | 0.11588219 | -0.8868251 | 1.11859 | 1 |
| Xie Wenhui 2023 | CT VS TCM | -0.58942769 | -2.039301 | 0.8604452 | 1 |
| Xie Wenhui 2023 | CT VS TF | -3.0733944 | -3.976414 | -2.170375 | 1 |
| Xie Wenhui 2023 | CT VS TUINA | 0.32143113 | -0.858712 | 1.501574 | 1 |
| Xie Wenhui 2023 | CT VS WN | -1.740247 | -2.516725 | -0.9637691 | 1 |
| Zhang Jing 2026 | CT VS ACU | -0.31566694 | -1.067636 | 0.4363025 | 1 |
| Zhang Jing 2026 | CT VS CM | -1.3130662 | -1.899112 | -0.72702 | 1 |
| Zhang Jing 2026 | CT VS HM | -1.2835414 | -2.02084 | -0.546243 | 1 |
| Zhang Jing 2026 | CT VS REHAB | 0.19465385 | -0.8033133 | 1.192621 | 1 |
| Zhang Jing 2026 | CT VS TCM | -0.64884255 | -2.751855 | 1.45417 | 1 |
| Zhang Jing 2026 | CT VS TF | -3.0696311 | -4.025521 | -2.113741 | 1 |
| Zhang Jing 2026 | CT VS TUINA | 0.36971098 | -0.8232263 | 1.562648 | 1 |
| Zhang Jing 2026 | CT VS WN | -1.6827285 | -2.483807 | -0.8816499 | 1 |
| Zhu Bin 2021 | CT VS ACU | -0.36426854 | -1.117736 | 0.3891985 | 1 |
| Zhu Bin 2021 | CT VS CM | -1.3313803 | -1.913557 | -0.7492037 | 1 |
| Zhu Bin 2021 | CT VS HM | -1.2943907 | -2.026433 | -0.5623479 | 1 |
| Zhu Bin 2021 | CT VS REHAB | 0.21150089 | -0.7813241 | 1.204326 | 1 |
| Zhu Bin 2021 | CT VS TCM | -0.52467284 | -1.982137 | 0.9327917 | 1 |
| Zhu Bin 2021 | CT VS TF | -3.0420812 | -3.948264 | -2.135898 | 1 |
| Zhu Bin 2021 | CT VS TUINA | 0.37063062 | -0.810869 | 1.55213 | 1 |
| Zhu Bin 2021 | CT VS WN | -1.6434393 | -2.432842 | -0.8540368 | 1 |
| Yu Ansheng 2017 | CT VS ACU | -0.38467823 | -1.148164 | 0.3788077 | 1 |
| Yu Ansheng 2017 | CT VS CM | -1.3342166 | -1.916182 | -0.7522509 | 1 |
| Yu Ansheng 2017 | CT VS HM | -1.2258703 | -1.978176 | -0.473565 | 1 |
| Yu Ansheng 2017 | CT VS REHAB | 0.20537404 | -0.785216 | 1.195964 | 1 |
| Yu Ansheng 2017 | CT VS TCM | -0.56848774 | -2.021515 | 0.8845391 | 1 |
| Yu Ansheng 2017 | CT VS TF | -3.0518636 | -3.956502 | -2.147225 | 1 |
| Yu Ansheng 2017 | CT VS TUINA | 0.34316452 | -0.8383467 | 1.524676 | 1 |
| Yu Ansheng 2017 | CT VS WN | -1.7198364 | -2.494997 | -0.9446762 | 1 |
| Xu Zhenkai 2018 | CT VS ACU | -0.32538715 | -1.067943 | 0.4171684 | 1 |
| Xu Zhenkai 2018 | CT VS CM | -1.3053384 | -1.887876 | -0.7228006 | 1 |
| Xu Zhenkai 2018 | CT VS HM | -1.2873633 | -2.020238 | -0.554489 | 1 |
| Xu Zhenkai 2018 | CT VS REHAB | 0.17930561 | -0.8143949 | 1.173006 | 1 |
| Xu Zhenkai 2018 | CT VS TCM | -0.57228743 | -2.030245 | 0.8856701 | 1 |
| Xu Zhenkai 2018 | CT VS TF | -3.057655 | -3.965165 | -2.150145 | 1 |
| Xu Zhenkai 2018 | CT VS TUINA | 0.15120209 | -1.251281 | 1.553685 | 1 |
| Xu Zhenkai 2018 | CT VS WN | -1.7217333 | -2.501521 | -0.9419458 | 1 |
| Feng Minhe 2021 | CT VS ACU | -0.34156206 | -1.112953 | 0.4298294 | 1 |
| Feng Minhe 2021 | CT VS CM | -1.3218476 | -1.908341 | -0.7353539 | 1 |
| Feng Minhe 2021 | CT VS HM | -1.2672784 | -2.02713 | -0.5074272 | 1 |
| Feng Minhe 2021 | CT VS REHAB | 0.19643316 | -0.8014035 | 1.19427 | 1 |
| Feng Minhe 2021 | CT VS TCM | -0.55959332 | -2.023187 | 0.9040005 | 1 |
| Feng Minhe 2021 | CT VS TF | -3.0518018 | -3.963012 | -2.140591 | 1 |
| Feng Minhe 2021 | CT VS TUINA | 0.35726857 | -0.83309 | 1.547627 | 1 |
| Feng Minhe 2021 | CT VS WN | -1.7024734 | -2.483657 | -0.9212901 | 1 |
| Wang Jianying 2025 | CT VS ACU | -0.07920774 | -0.6833174 | 0.5249019 | 1 |
| Wang Jianying 2025 | CT VS CM | -0.98627477 | -1.475226 | -0.4973239 | 1 |
| Wang Jianying 2025 | CT VS HM | -1.1252627 | -1.717019 | -0.5335066 | 1 |
| Wang Jianying 2025 | CT VS REHAB | 0.31952813 | -0.4762639 | 1.11532 | 1 |
| Wang Jianying 2025 | CT VS TCM | -0.45027228 | -1.614781 | 0.7142366 | 1 |
| Wang Jianying 2025 | CT VS TF | -3.0094892 | -3.733604 | -2.285374 | 1 |
| Wang Jianying 2025 | CT VS TUINA | 0.63421192 | -0.31593 | 1.584354 | 1 |
| Wang Jianying 2025 | CT VS WN | -1.5317218 | -2.153993 | -0.9094502 | 1 |
| Sun Jian 2009 | CT VS ACU | -0.24008206 | -0.9961817 | 0.5160176 | 1 |
| Sun Jian 2009 | CT VS CM | -1.3403064 | -1.918602 | -0.7620112 | 1 |
| Sun Jian 2009 | CT VS HM | -1.2670798 | -1.994718 | -0.5394412 | 1 |
| Sun Jian 2009 | CT VS REHAB | 0.21729642 | -0.768186 | 1.202779 | 1 |
| Sun Jian 2009 | CT VS TCM | -0.53055503 | -1.975522 | 0.914412 | 1 |
| Sun Jian 2009 | CT VS TF | -3.042167 | -3.941713 | -2.142621 | 1 |
| Sun Jian 2009 | CT VS TUINA | 0.36079235 | -0.8121638 | 1.533749 | 1 |
| Sun Jian 2009 | CT VS WN | -1.6548985 | -2.426455 | -0.8833418 | 1 |
| Sun Jianbing 2025 | CT VS ACU | -0.26168797 | -1.014753 | 0.4913775 | 1 |
| Sun Jianbing 2025 | CT VS CM | -1.23576 | -1.846372 | -0.6251482 | 1 |
| Sun Jianbing 2025 | CT VS HM | -1.2476579 | -1.982888 | -0.5124282 | 1 |
| Sun Jianbing 2025 | CT VS REHAB | 0.22562563 | -0.7655784 | 1.21683 | 1 |
| Sun Jianbing 2025 | CT VS TCM | -0.52948688 | -1.980497 | 0.9215228 | 1 |
| Sun Jianbing 2025 | CT VS TF | -3.0403039 | -3.943578 | -2.137029 | 1 |
| Sun Jianbing 2025 | CT VS TUINA | 0.43037175 | -0.7583593 | 1.619103 | 1 |
| Sun Jianbing 2025 | CT VS WN | -1.6545713 | -2.430594 | -0.8785487 | 1 |
| Gao Yuanguanyu 2021 | CT VS ACU | -0.31677248 | -1.077315 | 0.44377 | 1 |
| Gao Yuanguanyu 2021 | CT VS CM | -1.3098742 | -1.926437 | -0.6933111 | 1 |
| Gao Yuanguanyu 2021 | CT VS HM | -1.2830314 | -2.025527 | -0.5405361 | 1 |
| Gao Yuanguanyu 2021 | CT VS REHAB | 0.19503586 | -0.8060927 | 1.196164 | 1 |
| Gao Yuanguanyu 2021 | CT VS TCM | -0.55337269 | -2.018971 | 0.9122255 | 1 |
| Gao Yuanguanyu 2021 | CT VS TF | -3.0508483 | -3.963245 | -2.138451 | 1 |
| Gao Yuanguanyu 2021 | CT VS TUINA | 0.36899998 | -0.8317811 | 1.569781 | 1 |
| Gao Yuanguanyu 2021 | CT VS WN | -1.6912059 | -2.475007 | -0.9074048 | 1 |
| Jiang Yuqin 2025 | CT VS ACU | -0.33375025 | -1.100549 | 0.4330487 | 1 |
| Jiang Yuqin 2025 | CT VS CM | -1.3130055 | -1.899452 | -0.7265587 | 1 |
| Jiang Yuqin 2025 | CT VS HM | -1.2889574 | -2.026561 | -0.5513534 | 1 |
| Jiang Yuqin 2025 | CT VS REHAB | 0.18849092 | -0.8106039 | 1.187586 | 1 |
| Jiang Yuqin 2025 | CT VS TCM | -0.55920924 | -2.024153 | 0.9057347 | 1 |
| Jiang Yuqin 2025 | CT VS TF | -3.0532668 | -3.965296 | -2.141238 | 1 |
| Jiang Yuqin 2025 | CT VS TUINA | 0.36386834 | -0.8255671 | 1.553304 | 1 |
| Jiang Yuqin 2025 | CT VS WN | -1.7003194 | -2.482628 | -0.9180104 | 1 |
| Liu Chunyu 2021 | CT VS ACU | -0.35960543 | -1.084465 | 0.3652539 | 1 |
| Liu Chunyu 2021 | CT VS CM | -1.3384089 | -1.905111 | -0.7717065 | 1 |
| Liu Chunyu 2021 | CT VS HM | -1.323087 | -2.038923 | -0.607251 | 1 |
| Liu Chunyu 2021 | CT VS REHAB | 0.08243968 | -0.8959358 | 1.060815 | 1 |
| Liu Chunyu 2021 | CT VS TCM | -0.76105735 | -2.207967 | 0.6858523 | 1 |
| Liu Chunyu 2021 | CT VS TF | -3.4090761 | -4.421153 | -2.396999 | 1 |
| Liu Chunyu 2021 | CT VS TUINA | 0.32809169 | -0.8250588 | 1.481242 | 1 |
| Liu Chunyu 2021 | CT VS WN | -1.7557555 | -2.513974 | -0.9975365 | 1 |
| Yin Zhao 2023 | CT VS ACU | -0.31547372 | -1.067384 | 0.4364361 | 1 |
| Yin Zhao 2023 | CT VS CM | -1.3129651 | -1.898983 | -0.726947 | 1 |
| Yin Zhao 2023 | CT VS HM | -1.2834483 | -2.020717 | -0.5461799 | 1 |
| Yin Zhao 2023 | CT VS REHAB | 0.19477687 | -0.8031382 | 1.192692 | 1 |
| Yin Zhao 2023 | CT VS TCM | -0.471237 | -2.469603 | 1.527129 | 1 |
| Yin Zhao 2023 | CT VS TF | -3.0699955 | -4.02597 | -2.114021 | 1 |
| Yin Zhao 2023 | CT VS TUINA | 0.36990672 | -0.8229773 | 1.562791 | 1 |
| Yin Zhao 2023 | CT VS WN | -1.6823436 | -2.48325 | -0.8814375 | 1 |
| Zhang Aili 2023 | CT VS ACU | -0.3360596 | -1.094628 | 0.4225087 | 1 |
| Zhang Aili 2023 | CT VS CM | -1.3262926 | -1.918151 | -0.7344346 | 1 |
| Zhang Aili 2023 | CT VS HM | -1.3202495 | -2.124842 | -0.5156568 | 1 |
| Zhang Aili 2023 | CT VS REHAB | 0.17304299 | -0.8407506 | 1.186837 | 1 |
| Zhang Aili 2023 | CT VS TCM | -0.56400731 | -2.029445 | 0.9014304 | 1 |
| Zhang Aili 2023 | CT VS TF | -3.0572168 | -3.969974 | -2.14446 | 1 |
| Zhang Aili 2023 | CT VS TUINA | 0.35315865 | -0.8399041 | 1.546221 | 1 |
| Zhang Aili 2023 | CT VS WN | -1.7059364 | -2.490925 | -0.9209477 | 1 |
| Hua Yuqi 2015 | CT VS ACU | -0.32194294 | -1.066369 | 0.4224831 | 1 |
| Hua Yuqi 2015 | CT VS CM | -1.3143649 | -1.8973 | -0.7314302 | 1 |
| Hua Yuqi 2015 | CT VS HM | -1.2647668 | -2.01347 | -0.5160636 | 1 |
| Hua Yuqi 2015 | CT VS REHAB | 0.13031471 | -0.9496724 | 1.210302 | 1 |
| Hua Yuqi 2015 | CT VS TCM | -0.57023159 | -2.034297 | 0.8938337 | 1 |
| Hua Yuqi 2015 | CT VS TF | -3.0667627 | -3.982085 | -2.15144 | 1 |
| Hua Yuqi 2015 | CT VS TUINA | 0.36003629 | -0.8268937 | 1.546966 | 1 |
| Hua Yuqi 2015 | CT VS WN | -1.7089986 | -2.491145 | -0.9268521 | 1 |
| Wu Zongxiang 2017 | CT VS ACU | -0.31793376 | -1.063963 | 0.4280952 | 1 |
| Wu Zongxiang 2017 | CT VS CM | -1.3262012 | -1.915518 | -0.736884 | 1 |
| Wu Zongxiang 2017 | CT VS HM | -1.2847082 | -2.020573 | -0.5488436 | 1 |
| Wu Zongxiang 2017 | CT VS REHAB | 0.2042682 | -0.7971383 | 1.205675 | 1 |
| Wu Zongxiang 2017 | CT VS TCM | -0.53986225 | -2.008005 | 0.9282809 | 1 |
| Wu Zongxiang 2017 | CT VS TF | -3.0464141 | -3.958419 | -2.134409 | 1 |
| Wu Zongxiang 2017 | CT VS TUINA | 0.26676961 | -1.157922 | 1.691462 | 1 |
| Wu Zongxiang 2017 | CT VS WN | -1.6690021 | -2.473067 | -0.8649378 | 1 |
| Chen Jing 2016 | CT VS ACU | -0.31641192 | -1.055379 | 0.4225548 | 1 |
| Chen Jing 2016 | CT VS CM | -1.3323133 | -1.912111 | -0.7525152 | 1 |
| Chen Jing 2016 | CT VS HM | -1.2840563 | -2.013402 | -0.5547109 | 1 |
| Chen Jing 2016 | CT VS REHAB | 0.21043152 | -0.778472 | 1.199335 | 1 |
| Chen Jing 2016 | CT VS TCM | -0.53132002 | -1.982202 | 0.9195623 | 1 |
| Chen Jing 2016 | CT VS TF | -3.0436179 | -3.946676 | -2.140559 | 1 |
| Chen Jing 2016 | CT VS TUINA | 0.67054091 | -0.735303 | 2.076385 | 1 |
| Chen Jing 2016 | CT VS WN | -1.6549925 | -2.431293 | -0.8786919 | 1 |
| Wang Hongfei 2020 | CT VS ACU | -0.30335399 | -1.044273 | 0.4375648 | 1 |
| Wang Hongfei 2020 | CT VS CM | -1.3102442 | -1.88922 | -0.7312686 | 1 |
| Wang Hongfei 2020 | CT VS HM | -1.3151382 | -2.04899 | -0.5812866 | 1 |
| Wang Hongfei 2020 | CT VS REHAB | 0.03752729 | -1.032561 | 1.107615 | 1 |
| Wang Hongfei 2020 | CT VS TCM | -0.5373884 | -1.988737 | 0.9139605 | 1 |
| Wang Hongfei 2020 | CT VS TF | -3.0784275 | -3.984536 | -2.172319 | 1 |
| Wang Hongfei 2020 | CT VS TUINA | 0.38793024 | -0.7921984 | 1.568059 | 1 |
| Wang Hongfei 2020 | CT VS WN | -1.6336105 | -2.421058 | -0.8461629 | 1 |
| Zhao Huayun 2023 | CT VS ACU | -0.34561864 | -1.083894 | 0.3926569 | 1 |
| Zhao Huayun 2023 | CT VS CM | -1.3245666 | -1.901408 | -0.7477251 | 1 |
| Zhao Huayun 2023 | CT VS HM | -1.2500643 | -1.98138 | -0.5187491 | 1 |
| Zhao Huayun 2023 | CT VS REHAB | 0.38346554 | -0.6846058 | 1.451537 | 1 |
| Zhao Huayun 2023 | CT VS TCM | -0.57922062 | -2.025267 | 0.8668259 | 1 |
| Zhao Huayun 2023 | CT VS TF | -3.0193794 | -3.922204 | -2.116555 | 1 |
| Zhao Huayun 2023 | CT VS TUINA | 0.33210134 | -0.8436648 | 1.507868 | 1 |
| Zhao Huayun 2023 | CT VS WN | -1.7718636 | -2.556944 | -0.9867835 | 1 |
| Li Xiuxia 2019 | CT VS ACU | -0.25582526 | -1.019643 | 0.5079921 | 1 |
| Li Xiuxia 2019 | CT VS CM | -1.2810609 | -1.869418 | -0.6927041 | 1 |
| Li Xiuxia 2019 | CT VS HM | -1.2474227 | -1.986623 | -0.5082225 | 1 |
| Li Xiuxia 2019 | CT VS REHAB | 0.26463907 | -0.746773 | 1.276051 | 1 |
| Li Xiuxia 2019 | CT VS TCM | -0.47657578 | -1.946875 | 0.9937237 | 1 |
| Li Xiuxia 2019 | CT VS TF | -3.0223317 | -3.931235 | -2.113429 | 1 |
| Li Xiuxia 2019 | CT VS TUINA | 0.42922561 | -0.7658589 | 1.62431 | 1 |
| Li Xiuxia 2019 | CT VS WN | -1.568193 | -2.419136 | -0.7172498 | 1 |
| Xu Mengxia 2019 | CT VS ACU | -0.34864057 | -1.106013 | 0.4087319 | 1 |
| Xu Mengxia 2019 | CT VS CM | -1.3257628 | -1.910495 | -0.7410308 | 1 |
| Xu Mengxia 2019 | CT VS HM | -1.2913586 | -2.026411 | -0.5563061 | 1 |
| Xu Mengxia 2019 | CT VS REHAB | 0.20456075 | -0.7924548 | 1.201576 | 1 |
| Xu Mengxia 2019 | CT VS TCM | -0.53592285 | -1.999585 | 0.927739 | 1 |
| Xu Mengxia 2019 | CT VS TF | -3.0455999 | -3.955554 | -2.135646 | 1 |
| Xu Mengxia 2019 | CT VS TUINA | 0.36816719 | -0.8182778 | 1.554612 | 1 |
| Xu Mengxia 2019 | CT VS WN | -1.6620762 | -2.455794 | -0.868358 | 1 |
| Pan Meng 2025 | CT VS ACU | -0.28578714 | -1.050154 | 0.4785794 | 1 |
| Pan Meng 2025 | CT VS CM | -1.3265086 | -1.911319 | -0.7416981 | 1 |
| Pan Meng 2025 | CT VS HM | -1.2777289 | -2.01342 | -0.5420381 | 1 |
| Pan Meng 2025 | CT VS REHAB | 0.20346986 | -0.7930298 | 1.19997 | 1 |
| Pan Meng 2025 | CT VS TCM | -0.54448065 | -2.005656 | 0.9166948 | 1 |
| Pan Meng 2025 | CT VS TF | -3.047488 | -3.957171 | -2.137805 | 1 |
| Pan Meng 2025 | CT VS TUINA | 0.36267078 | -0.8236428 | 1.548984 | 1 |
| Pan Meng 2025 | CT VS WN | -1.6770213 | -2.457203 | -0.8968391 | 1 |
| Zhou Huaming 2024 | CT VS ACU | -0.38995258 | -1.122264 | 0.3423585 | 1 |
| Zhou Huaming 2024 | CT VS CM | -1.35859 | -1.929793 | -0.7873873 | 1 |
| Zhou Huaming 2024 | CT VS HM | -1.383974 | -2.114732 | -0.6532162 | 1 |
| Zhou Huaming 2024 | CT VS REHAB | -0.13920463 | -1.223754 | 0.9453445 | 1 |
| Zhou Huaming 2024 | CT VS TCM | -0.47756762 | -1.906049 | 0.950914 | 1 |
| Zhou Huaming 2024 | CT VS TF | -2.7954955 | -3.75931 | -1.831681 | 1 |
| Zhou Huaming 2024 | CT VS TUINA | 0.30440258 | -0.8550208 | 1.463826 | 1 |
| Zhou Huaming 2024 | CT VS WN | -1.7866042 | -2.554689 | -1.018519 | 1 |
| Li Jia 2024 | CT VS ACU | -0.25179526 | -1.000756 | 0.4971657 | 1 |
| Li Jia 2024 | CT VS CM | -1.291807 | -1.869511 | -0.7141027 | 1 |
| Li Jia 2024 | CT VS HM | -1.2723288 | -1.998619 | -0.5460383 | 1 |
| Li Jia 2024 | CT VS REHAB | 0.15883883 | -0.8262831 | 1.143961 | 1 |
| Li Jia 2024 | CT VS TCM | -0.60742496 | -2.053555 | 0.8387055 | 1 |
| Li Jia 2024 | CT VS TF | -3.068593 | -3.967502 | -2.169684 | 1 |
| Li Jia 2024 | CT VS TUINA | 0.35109645 | -0.8207873 | 1.52298 | 1 |
| Li Jia 2024 | CT VS WN | -1.7800438 | -2.565204 | -0.9948833 | 1 |
| Xiong Qi 2024 | CT VS ACU | -0.32073994 | -1.047344 | 0.4058646 | 1 |
| Xiong Qi 2024 | CT VS CM | -1.325583 | -1.894411 | -0.7567552 | 1 |
| Xiong Qi 2024 | CT VS HM | -1.3870322 | -2.12013 | -0.6539341 | 1 |
| Xiong Qi 2024 | CT VS REHAB | 0.48182711 | -0.5835189 | 1.547173 | 1 |
| Xiong Qi 2024 | CT VS TCM | -0.48658407 | -1.915887 | 0.9427192 | 1 |
| Xiong Qi 2024 | CT VS TF | -2.9822816 | -3.87624 | -2.088323 | 1 |
| Xiong Qi 2024 | CT VS TUINA | 0.38006846 | -0.7779232 | 1.53806 | 1 |
| Xiong Qi 2024 | CT VS WN | -1.6261046 | -2.390378 | -0.8618315 | 1 |

**Sensitivity analysis based on the JOA score group in the subgroup analysis of patients with LDH (Table S56)**

| **dropped_id** | **comparison** | **eff** | **lci** | **uci** | **connected** |
| --- | --- | --- | --- | --- | --- |
| Chunyue Cai 2018 | CT VS ACU | 1.5408668 | -1.047814 | 4.129547 | 1 |
| Chunyue Cai 2018 | CT VS CM | 4.7911463 | 2.206392 | 7.375901 | 1 |
| Chunyue Cai 2018 | CT VS HM | 4.7094616 | 2.028988 | 7.389935 | 1 |
| Chunyue Cai 2018 | CT VS REHAB | 0.5414084 | -2.79998 | 3.882797 | 1 |
| Chunyue Cai 2018 | CT VS TCM | 1.2132549 | -1.990045 | 4.416554 | 1 |
| Chunyue Cai 2018 | CT VS TF | 6.014457 | 3.760975 | 8.26794 | 1 |
| Chunyue Cai 2018 | CT VS TUINA | 0.73948592 | -2.926083 | 4.405055 | 1 |
| Chunyue Cai 2018 | CT VS WN | 5.6858592 | 3.301988 | 8.06973 | 1 |
| Xu Minmin 2016 | CT VS ACU | 1.7120987 | -0.9018576 | 4.326055 | 1 |
| Xu Minmin 2016 | CT VS CM | 4.6091256 | 2.011435 | 7.206816 | 1 |
| Xu Minmin 2016 | CT VS HM | 4.7388879 | 2.008739 | 7.469036 | 1 |
| Xu Minmin 2016 | CT VS REHAB | 0.60730184 | -2.792736 | 4.00734 | 1 |
| Xu Minmin 2016 | CT VS TCM | 1.2607783 | -2.041587 | 4.563144 | 1 |
| Xu Minmin 2016 | CT VS TF | 6.0383108 | 3.401274 | 8.675347 | 1 |
| Xu Minmin 2016 | CT VS TUINA | 0.55719059 | -3.149208 | 4.263589 | 1 |
| Xu Minmin 2016 | CT VS WN | 5.7715858 | 3.346513 | 8.196658 | 1 |
| Zhang Qun 2023 | CT VS ACU | 2.2398711 | 0.1375153 | 4.342227 | 1 |
| Zhang Qun 2023 | CT VS CM | 4.9609677 | 2.884895 | 7.03704 | 1 |
| Zhang Qun 2023 | CT VS HM | 5.0016183 | 2.842985 | 7.160252 | 1 |
| Zhang Qun 2023 | CT VS REHAB | 0.94440977 | -1.738096 | 3.626916 | 1 |
| Zhang Qun 2023 | CT VS TCM | 2.0033362 | -0.6162442 | 4.622917 | 1 |
| Zhang Qun 2023 | CT VS TF | 7.7969987 | 5.71846 | 9.875538 | 1 |
| Zhang Qun 2023 | CT VS TUINA | 0.91747584 | -1.995204 | 3.830156 | 1 |
| Zhang Qun 2023 | CT VS WN | 6.1228779 | 4.173729 | 8.072027 | 1 |
| Hu Qinqin 2024 | CT VS ACU | 1.9083582 | -0.9251961 | 4.741912 | 1 |
| Hu Qinqin 2024 | CT VS CM | 4.8818187 | 1.817317 | 7.946321 | 1 |
| Hu Qinqin 2024 | CT VS HM | 4.7746624 | 2.057542 | 7.491783 | 1 |
| Hu Qinqin 2024 | CT VS REHAB | 0.68443111 | -2.7208 | 4.089663 | 1 |
| Hu Qinqin 2024 | CT VS TCM | 1.32501 | -1.930194 | 4.580214 | 1 |
| Hu Qinqin 2024 | CT VS TF | 6.0969147 | 3.804351 | 8.389478 | 1 |
| Hu Qinqin 2024 | CT VS TUINA | 0.82969764 | -3.208149 | 4.867545 | 1 |
| Hu Qinqin 2024 | CT VS WN | 5.8693692 | 3.400834 | 8.337905 | 1 |
| Xu Fen 2025 | CT VS ACU | 1.9126007 | -0.5939253 | 4.419127 | 1 |
| Xu Fen 2025 | CT VS CM | 4.748108 | 2.255438 | 7.240777 | 1 |
| Xu Fen 2025 | CT VS HM | 5.9320063 | 2.724736 | 9.139277 | 1 |
| Xu Fen 2025 | CT VS REHAB | 1.217242 | -2.170456 | 4.60494 | 1 |
| Xu Fen 2025 | CT VS TCM | 1.820875 | -1.418371 | 5.060121 | 1 |
| Xu Fen 2025 | CT VS TF | 6.1911371 | 3.987965 | 8.394309 | 1 |
| Xu Fen 2025 | CT VS TUINA | 0.69731565 | -2.853689 | 4.248321 | 1 |
| Xu Fen 2025 | CT VS WN | 6.0821944 | 3.715116 | 8.449273 | 1 |
| Zhong Qunhua 2024 | CT VS ACU | 1.2351574 | -0.6931795 | 3.163494 | 1 |
| Zhong Qunhua 2024 | CT VS CM | 4.2393374 | 2.341637 | 6.137038 | 1 |
| Zhong Qunhua 2024 | CT VS HM | 4.4095552 | 2.447155 | 6.371955 | 1 |
| Zhong Qunhua 2024 | CT VS REHAB | 0.23354438 | -2.201706 | 2.668794 | 1 |
| Zhong Qunhua 2024 | CT VS TCM | 0.39542694 | -1.989639 | 2.780493 | 1 |
| Zhong Qunhua 2024 | CT VS TF | 4.0834428 | 2.207304 | 5.959581 | 1 |
| Zhong Qunhua 2024 | CT VS TUINA | 0.20109552 | -2.437017 | 2.839208 | 1 |
| Zhong Qunhua 2024 | CT VS WN | 5.3371525 | 3.550061 | 7.124243 | 1 |
| Liu Wenlin 2024 | CT VS ACU | 1.8909248 | -0.6864012 | 4.468251 | 1 |
| Liu Wenlin 2024 | CT VS CM | 4.4436692 | 1.870633 | 7.016705 | 1 |
| Liu Wenlin 2024 | CT VS HM | 4.7705995 | 2.097908 | 7.443291 | 1 |
| Liu Wenlin 2024 | CT VS REHAB | 0.67684219 | -2.654414 | 4.008099 | 1 |
| Liu Wenlin 2024 | CT VS TCM | 1.318445 | -1.875502 | 4.512392 | 1 |
| Liu Wenlin 2024 | CT VS TF | 6.0931313 | 3.846572 | 8.33969 | 1 |
| Liu Wenlin 2024 | CT VS TUINA | 0.39250818 | -3.259163 | 4.044179 | 1 |
| Liu Wenlin 2024 | CT VS WN | 5.8588507 | 3.482643 | 8.235059 | 1 |
| Wu Yusheng 2025 | CT VS ACU | 1.6419743 | -0.9403802 | 4.224329 | 1 |
| Wu Yusheng 2025 | CT VS CM | 4.5588776 | 1.987817 | 7.129939 | 1 |
| Wu Yusheng 2025 | CT VS HM | 4.2618539 | 0.9946118 | 7.529096 | 1 |
| Wu Yusheng 2025 | CT VS REHAB | 0.36717576 | -3.121598 | 3.85595 | 1 |
| Wu Yusheng 2025 | CT VS TCM | 1.0446111 | -2.290393 | 4.379615 | 1 |
| Wu Yusheng 2025 | CT VS TF | 5.9995244 | 3.721788 | 8.277261 | 1 |
| Wu Yusheng 2025 | CT VS TUINA | 0.50726637 | -3.163245 | 4.177777 | 1 |
| Wu Yusheng 2025 | CT VS WN | 5.6508498 | 3.214636 | 8.087064 | 1 |
| Xu Shihai 2019 | CT VS ACU | 1.5188979 | -1.170477 | 4.208273 | 1 |
| Xu Shihai 2019 | CT VS CM | 4.4717803 | 1.84529 | 7.098271 | 1 |
| Xu Shihai 2019 | CT VS HM | 4.7846147 | 2.077957 | 7.491272 | 1 |
| Xu Shihai 2019 | CT VS REHAB | 0.72256315 | -2.671348 | 4.116475 | 1 |
| Xu Shihai 2019 | CT VS TCM | 1.3257084 | -1.908755 | 4.560172 | 1 |
| Xu Shihai 2019 | CT VS TF | 6.0314282 | 3.759615 | 8.303242 | 1 |
| Xu Shihai 2019 | CT VS TUINA | 0.42024871 | -3.290841 | 4.131339 | 1 |
| Xu Shihai 2019 | CT VS WN | 5.9215808 | 3.458744 | 8.384418 | 1 |
| Liang Yuanyuan 2014 | CT VS ACU | 1.2996137 | -1.499221 | 4.098449 | 1 |
| Liang Yuanyuan 2014 | CT VS CM | 4.0221905 | 1.00186 | 7.042521 | 1 |
| Liang Yuanyuan 2014 | CT VS HM | 4.6672614 | 1.977308 | 7.357215 | 1 |
| Liang Yuanyuan 2014 | CT VS REHAB | 0.44800216 | -2.922252 | 3.818257 | 1 |
| Liang Yuanyuan 2014 | CT VS TCM | 1.1406615 | -2.082058 | 4.363381 | 1 |
| Liang Yuanyuan 2014 | CT VS TF | 5.9601969 | 3.691496 | 8.228898 | 1 |
| Liang Yuanyuan 2014 | CT VS TUINA | -0.02859467 | -4.014672 | 3.957483 | 1 |
| Liang Yuanyuan 2014 | CT VS WN | 5.5665024 | 3.1233 | 8.009705 | 1 |
| Liu Yiyi 2022 | CT VS ACU | 1.6492212 | -1.199629 | 4.498071 | 1 |
| Liu Yiyi 2022 | CT VS CM | 4.5642953 | 1.850918 | 7.277672 | 1 |
| Liu Yiyi 2022 | CT VS HM | 4.7368735 | 2.025785 | 7.447962 | 1 |
| Liu Yiyi 2022 | CT VS REHAB | 0.58983391 | -2.805335 | 3.985003 | 1 |
| Liu Yiyi 2022 | CT VS TCM | 1.271482 | -1.965876 | 4.50884 | 1 |
| Liu Yiyi 2022 | CT VS TF | 6.1029393 | 3.663225 | 8.542653 | 1 |
| Liu Yiyi 2022 | CT VS TUINA | 0.51255023 | -3.266927 | 4.292027 | 1 |
| Liu Yiyi 2022 | CT VS WN | 5.7459119 | 3.292639 | 8.199184 | 1 |
| Guo Hui 2023 | CT VS ACU | 1.8047097 | -0.7614077 | 4.370827 | 1 |
| Guo Hui 2023 | CT VS CM | 4.6737698 | 2.116959 | 7.23058 | 1 |
| Guo Hui 2023 | CT VS HM | 4.3376026 | 1.411683 | 7.263522 | 1 |
| Guo Hui 2023 | CT VS REHAB | 0.5488282 | -2.800733 | 3.898389 | 1 |
| Guo Hui 2023 | CT VS TCM | 1.9928187 | -1.833962 | 5.8196 | 1 |
| Guo Hui 2023 | CT VS TF | 6.2048092 | 3.90706 | 8.502559 | 1 |
| Guo Hui 2023 | CT VS TUINA | 0.6221728 | -3.028596 | 4.272941 | 1 |
| Guo Hui 2023 | CT VS WN | 5.8859839 | 3.485918 | 8.28605 | 1 |
| Wen Qi 2023 | CT VS ACU | 1.7680437 | -0.9166665 | 4.452754 | 1 |
| Wen Qi 2023 | CT VS CM | 4.6484063 | 2.02087 | 7.275942 | 1 |
| Wen Qi 2023 | CT VS HM | 4.7301621 | 2.01636 | 7.443964 | 1 |
| Wen Qi 2023 | CT VS REHAB | 0.58203855 | -2.818569 | 3.982646 | 1 |
| Wen Qi 2023 | CT VS TCM | 1.2526586 | -1.990044 | 4.495361 | 1 |
| Wen Qi 2023 | CT VS TF | 6.0596565 | 3.781405 | 8.337909 | 1 |
| Wen Qi 2023 | CT VS TUINA | 0.59657394 | -3.120953 | 4.314101 | 1 |
| Wen Qi 2023 | CT VS WN | 5.7375236 | 3.275187 | 8.19986 | 1 |
| Lin Mingqin 2021 | CT VS ACU | 1.9933327 | -0.6562091 | 4.642874 | 1 |
| Lin Mingqin 2021 | CT VS CM | 4.8071618 | 2.210785 | 7.403538 | 1 |
| Lin Mingqin 2021 | CT VS HM | 4.8988576 | 2.186934 | 7.610781 | 1 |
| Lin Mingqin 2021 | CT VS REHAB | 0.98219619 | -2.496723 | 4.461116 | 1 |
| Lin Mingqin 2021 | CT VS TCM | 1.5157524 | -1.751836 | 4.783341 | 1 |
| Lin Mingqin 2021 | CT VS TF | 6.1472313 | 3.882735 | 8.411728 | 1 |
| Lin Mingqin 2021 | CT VS TUINA | 0.75548131 | -2.918298 | 4.429261 | 1 |
| Lin Mingqin 2021 | CT VS WN | 6.2545154 | 3.561869 | 8.947162 | 1 |
| Zhang Jing 2026 | CT VS ACU | 1.7700045 | -0.8355021 | 4.375511 | 1 |
| Zhang Jing 2026 | CT VS CM | 4.6498898 | 2.06089 | 7.238889 | 1 |
| Zhang Jing 2026 | CT VS HM | 4.6792494 | 1.930509 | 7.42799 | 1 |
| Zhang Jing 2026 | CT VS REHAB | 0.66508842 | -2.73563 | 4.065807 | 1 |
| Zhang Jing 2026 | CT VS TCM | 0.96095279 | -2.954221 | 4.876127 | 1 |
| Zhang Jing 2026 | CT VS TF | 6.0052109 | 3.698874 | 8.311548 | 1 |
| Zhang Jing 2026 | CT VS TUINA | 0.59801337 | -3.095186 | 4.291213 | 1 |
| Zhang Jing 2026 | CT VS WN | 5.8887065 | 3.353836 | 8.423576 | 1 |
| Zhu Bin 2021 | CT VS ACU | 2.0329196 | -0.5846679 | 4.650507 | 1 |
| Zhu Bin 2021 | CT VS CM | 4.8346014 | 2.264322 | 7.404881 | 1 |
| Zhu Bin 2021 | CT VS HM | 4.671983 | 2.009941 | 7.334025 | 1 |
| Zhu Bin 2021 | CT VS REHAB | 0.43476732 | -2.896957 | 3.766492 | 1 |
| Zhu Bin 2021 | CT VS TCM | 1.1748843 | -2.006512 | 4.356281 | 1 |
| Zhu Bin 2021 | CT VS TF | 6.0902471 | 3.856315 | 8.32418 | 1 |
| Zhu Bin 2021 | CT VS TUINA | 0.78314597 | -2.856359 | 4.422651 | 1 |
| Zhu Bin 2021 | CT VS WN | 5.5433729 | 3.136014 | 7.950732 | 1 |
| Xu Zhenkai 2018 | CT VS ACU | 1.7184614 | -0.8594359 | 4.296359 | 1 |
| Xu Zhenkai 2018 | CT VS CM | 4.6143405 | 2.038841 | 7.189839 | 1 |
| Xu Zhenkai 2018 | CT VS HM | 4.7414356 | 2.029497 | 7.453374 | 1 |
| Xu Zhenkai 2018 | CT VS REHAB | 0.61086449 | -2.766848 | 3.988577 | 1 |
| Xu Zhenkai 2018 | CT VS TCM | 1.2678223 | -1.971043 | 4.506688 | 1 |
| Xu Zhenkai 2018 | CT VS TF | 6.0540831 | 3.774677 | 8.333489 | 1 |
| Xu Zhenkai 2018 | CT VS TUINA | 0.17679113 | -3.958177 | 4.311759 | 1 |
| Xu Zhenkai 2018 | CT VS WN | 5.7753693 | 3.37494 | 8.175798 | 1 |
| Wang Guoshu 2017 | CT VS ACU | 1.7396518 | -0.8642676 | 4.343571 | 1 |
| Wang Guoshu 2017 | CT VS CM | 4.5907438 | 1.99014 | 7.191348 | 1 |
| Wang Guoshu 2017 | CT VS HM | 4.7450136 | 2.037515 | 7.452512 | 1 |
| Wang Guoshu 2017 | CT VS REHAB | 0.61897251 | -2.755587 | 3.993532 | 1 |
| Wang Guoshu 2017 | CT VS TCM | 1.2740413 | -1.960763 | 4.508846 | 1 |
| Wang Guoshu 2017 | CT VS TF | 6.0589028 | 3.78234 | 8.335465 | 1 |
| Wang Guoshu 2017 | CT VS TUINA | 0.53900541 | -3.157983 | 4.235994 | 1 |
| Wang Guoshu 2017 | CT VS WN | 5.7856218 | 3.381501 | 8.189742 | 1 |
| Sun Jian 2009 | CT VS ACU | 1.7238465 | -0.8975341 | 4.345227 | 1 |
| Sun Jian 2009 | CT VS CM | 4.6060361 | 1.987855 | 7.224217 | 1 |
| Sun Jian 2009 | CT VS HM | 4.7425178 | 2.024053 | 7.460982 | 1 |
| Sun Jian 2009 | CT VS REHAB | 0.61305986 | -2.775876 | 4.001996 | 1 |
| Sun Jian 2009 | CT VS TCM | 1.2696194 | -1.978415 | 4.517653 | 1 |
| Sun Jian 2009 | CT VS TF | 6.055254 | 3.768938 | 8.34157 | 1 |
| Sun Jian 2009 | CT VS TUINA | 0.55415725 | -3.16314 | 4.271454 | 1 |
| Sun Jian 2009 | CT VS WN | 5.7782906 | 3.363142 | 8.193439 | 1 |
| Xu Chenghua 2018 | CT VS ACU | 1.7179328 | -0.8535917 | 4.289457 | 1 |
| Xu Chenghua 2018 | CT VS CM | 4.6131237 | 2.044303 | 7.181945 | 1 |
| Xu Chenghua 2018 | CT VS HM | 4.7411626 | 2.036236 | 7.446089 | 1 |
| Xu Chenghua 2018 | CT VS REHAB | 0.61051955 | -2.758389 | 3.979429 | 1 |
| Xu Chenghua 2018 | CT VS TCM | 1.2674376 | -1.963156 | 4.498032 | 1 |
| Xu Chenghua 2018 | CT VS TF | 6.0540218 | 3.780683 | 8.327361 | 1 |
| Xu Chenghua 2018 | CT VS TUINA | 1.013509 | -3.087271 | 5.114289 | 1 |
| Xu Chenghua 2018 | CT VS WN | 5.7747787 | 3.380224 | 8.169333 | 1 |
| Jiang Yuqin 2025 | CT VS ACU | 1.6133593 | -0.9990498 | 4.225769 | 1 |
| Jiang Yuqin 2025 | CT VS CM | 4.7174837 | 2.10842 | 7.326547 | 1 |
| Jiang Yuqin 2025 | CT VS HM | 4.7228539 | 2.015739 | 7.429968 | 1 |
| Jiang Yuqin 2025 | CT VS REHAB | 0.5700014 | -2.804797 | 3.9448 | 1 |
| Jiang Yuqin 2025 | CT VS TCM | 1.2359337 | -1.998769 | 4.470636 | 1 |
| Jiang Yuqin 2025 | CT VS TF | 6.0304478 | 3.753908 | 8.306988 | 1 |
| Jiang Yuqin 2025 | CT VS TUINA | 0.6655997 | -3.036837 | 4.368036 | 1 |
| Jiang Yuqin 2025 | CT VS WN | 5.7229963 | 3.316972 | 8.12902 | 1 |
| Yin Zhao 2023 | CT VS ACU | 1.6892611 | -0.8851378 | 4.26366 | 1 |
| Yin Zhao 2023 | CT VS CM | 4.5925022 | 2.022903 | 7.162101 | 1 |
| Yin Zhao 2023 | CT VS HM | 4.5967116 | 1.79974 | 7.393683 | 1 |
| Yin Zhao 2023 | CT VS REHAB | 0.49242218 | -2.925779 | 3.910623 | 1 |
| Yin Zhao 2023 | CT VS TCM | 0.80913472 | -3.136595 | 4.754865 | 1 |
| Yin Zhao 2023 | CT VS TF | 6.2159501 | 3.80655 | 8.62535 | 1 |
| Yin Zhao 2023 | CT VS TUINA | 0.54078853 | -3.132741 | 4.214318 | 1 |
| Yin Zhao 2023 | CT VS WN | 5.6699619 | 3.220664 | 8.11926 | 1 |
| Chen Jing 2016 | CT VS ACU | 1.7188195 | -0.8701598 | 4.307799 | 1 |
| Chen Jing 2016 | CT VS CM | 4.6156435 | 2.02835 | 7.202937 | 1 |
| Chen Jing 2016 | CT VS HM | 4.7418029 | 2.017893 | 7.465713 | 1 |
| Chen Jing 2016 | CT VS REHAB | 0.61124028 | -2.781517 | 4.003998 | 1 |
| Chen Jing 2016 | CT VS TCM | 1.268311 | -1.984684 | 4.521306 | 1 |
| Chen Jing 2016 | CT VS TF | 6.0540663 | 3.764295 | 8.343838 | 1 |
| Chen Jing 2016 | CT VS TUINA | 0.4888265 | -3.668503 | 4.646156 | 1 |
| Chen Jing 2016 | CT VS WN | 5.7761025 | 3.365595 | 8.18661 | 1 |
| Wang Hongfei 2020 | CT VS ACU | 1.6274852 | -0.9051703 | 4.160141 | 1 |
| Wang Hongfei 2020 | CT VS CM | 4.5474221 | 2.022307 | 7.072538 | 1 |
| Wang Hongfei 2020 | CT VS HM | 4.9789599 | 2.281957 | 7.675962 | 1 |
| Wang Hongfei 2020 | CT VS REHAB | 1.5126614 | -2.260266 | 5.285589 | 1 |
| Wang Hongfei 2020 | CT VS TCM | 1.2853389 | -1.885069 | 4.455747 | 1 |
| Wang Hongfei 2020 | CT VS TF | 6.0429554 | 3.813854 | 8.272058 | 1 |
| Wang Hongfei 2020 | CT VS TUINA | 0.4963518 | -3.108294 | 4.100998 | 1 |
| Wang Hongfei 2020 | CT VS WN | 5.6042202 | 3.227374 | 7.981066 | 1 |
| Zhao Huayun 2023 | CT VS ACU | 1.8314974 | -0.6854949 | 4.34849 | 1 |
| Zhao Huayun 2023 | CT VS CM | 4.6913534 | 2.182719 | 7.199988 | 1 |
| Zhao Huayun 2023 | CT VS HM | 4.4611015 | 1.779621 | 7.142582 | 1 |
| Zhao Huayun 2023 | CT VS REHAB | -0.44352356 | -4.221356 | 3.334309 | 1 |
| Zhao Huayun 2023 | CT VS TCM | 1.2449657 | -1.90378 | 4.393711 | 1 |
| Zhao Huayun 2023 | CT VS TF | 6.0698546 | 3.856676 | 8.283033 | 1 |
| Zhao Huayun 2023 | CT VS TUINA | 0.64033969 | -2.939046 | 4.219725 | 1 |
| Zhao Huayun 2023 | CT VS WN | 5.9729154 | 3.609751 | 8.33608 | 1 |
| Li Xiuxia 2019 | CT VS ACU | 1.4755075 | -1.193225 | 4.14424 | 1 |
| Li Xiuxia 2019 | CT VS CM | 4.4407164 | 1.83083 | 7.050602 | 1 |
| Li Xiuxia 2019 | CT VS HM | 4.6007317 | 1.876772 | 7.324692 | 1 |
| Li Xiuxia 2019 | CT VS REHAB | 0.28108711 | -3.224053 | 3.786227 | 1 |
| Li Xiuxia 2019 | CT VS TCM | 1.0466651 | -2.238278 | 4.331608 | 1 |
| Li Xiuxia 2019 | CT VS TF | 5.9720749 | 3.69864 | 8.245509 | 1 |
| Li Xiuxia 2019 | CT VS TUINA | 0.38937267 | -3.300218 | 4.078963 | 1 |
| Li Xiuxia 2019 | CT VS WN | 5.3487063 | 2.615462 | 8.08195 | 1 |
| Xiong Qi 2024 | CT VS ACU | 1.7504127 | -0.8706324 | 4.371458 | 1 |
| Xiong Qi 2024 | CT VS CM | 4.636157 | 2.036752 | 7.235562 | 1 |
| Xiong Qi 2024 | CT VS HM | 4.656018 | 1.707152 | 7.604885 | 1 |
| Xiong Qi 2024 | CT VS REHAB | 0.79336784 | -3.370023 | 4.956759 | 1 |
| Xiong Qi 2024 | CT VS TCM | 1.2610455 | -1.986237 | 4.508327 | 1 |
| Xiong Qi 2024 | CT VS TF | 6.0581268 | 3.772785 | 8.343469 | 1 |
| Xiong Qi 2024 | CT VS TUINA | 0.58423431 | -3.120379 | 4.288848 | 1 |
| Xiong Qi 2024 | CT VS WN | 5.8359571 | 3.298295 | 8.373619 | 1 |

**Sensitivity analysis based on the cure rate group in the subgroup analysis of patients with LDH. (Table S57)**

| **dropped_id** | **comparison** | **log_eff** | **log_lci** | **log_uci** | **OR** | **OR_lci** | **OR_uci** | **connected** |
| --- | --- | --- | --- | --- | --- | --- | --- | --- |
| Chunyue Cai 2018 | CT VS ACU | 0.13338521 | -0.1896612 | 0.4564317 | 1.1426901 | 0.8272393 | 1.578432 | 1 |
| Chunyue Cai 2018 | CT VS CM | 0.45708731 | 0.1904191 | 0.7237555 | 1.5794668 | 1.209756 | 2.062163 | 1 |
| Chunyue Cai 2018 | CT VS HM | 0.666314 | 0.3290316 | 1.003596 | 1.9470473 | 1.389622 | 2.728075 | 1 |
| Chunyue Cai 2018 | CT VS REHAB | 0.15548043 | -0.8218851 | 1.132846 | 1.1682191 | 0.4396022 | 3.104479 | 1 |
| Chunyue Cai 2018 | CT VS TCM | 0.41499029 | -0.1029205 | 0.9329011 | 1.514356 | 0.9021987 | 2.541873 | 1 |
| Chunyue Cai 2018 | CT VS TF | 0.51082562 | 0.1616043 | 0.860047 | 1.6666667 | 1.175395 | 2.363272 | 1 |
| Chunyue Cai 2018 | CT VS TUINA | 0.02068684 | -0.3685866 | 0.4099602 | 1.0209023 | 0.6917113 | 1.506758 | 1 |
| Chunyue Cai 2018 | CT VS WN | 0.48938517 | 0.1983691 | 0.7804012 | 1.6313129 | 1.219412 | 2.182348 | 1 |
| Hu Qinqin 2024 | CT VS ACU | 0.06891714 | -0.2708813 | 0.4087155 | 1.0713474 | 0.7627071 | 1.504884 | 1 |
| Hu Qinqin 2024 | CT VS CM | 0.41998635 | 0.1270096 | 0.712963 | 1.5219408 | 1.135428 | 2.040027 | 1 |
| Hu Qinqin 2024 | CT VS HM | 0.63943687 | 0.2908881 | 0.9879857 | 1.8954132 | 1.337615 | 2.685819 | 1 |
| Hu Qinqin 2024 | CT VS REHAB | 0.12860378 | -0.8527066 | 1.109914 | 1.1372394 | 0.4262597 | 3.034098 | 1 |
| Hu Qinqin 2024 | CT VS TCM | 0.38811361 | -0.1372036 | 0.9134309 | 1.4741973 | 0.8717927 | 2.492861 | 1 |
| Hu Qinqin 2024 | CT VS TF | 0.51082562 | 0.1616043 | 0.860047 | 1.6666667 | 1.175395 | 2.363272 | 1 |
| Hu Qinqin 2024 | CT VS TUINA | -0.01009815 | -0.4089848 | 0.3887885 | 0.98995267 | 0.6643243 | 1.475193 | 1 |
| Hu Qinqin 2024 | CT VS WN | 0.46517576 | 0.1697633 | 0.7605882 | 1.592294 | 1.185024 | 2.139534 | 1 |
| Zhong Qunhua 2024 | CT VS ACU | 0.10661893 | -0.2152047 | 0.4284426 | 1.1125102 | 0.8063763 | 1.534865 | 1 |
| Zhong Qunhua 2024 | CT VS CM | 0.46197382 | 0.1953554 | 0.7285923 | 1.5872038 | 1.215743 | 2.072161 | 1 |
| Zhong Qunhua 2024 | CT VS HM | 0.66985401 | 0.3325923 | 1.007116 | 1.953952 | 1.394579 | 2.737693 | 1 |
| Zhong Qunhua 2024 | CT VS REHAB | 0.15902038 | -0.818338 | 1.136379 | 1.1723618 | 0.4411643 | 3.115466 | 1 |
| Zhong Qunhua 2024 | CT VS TCM | 0.41853024 | -0.0993671 | 0.9364276 | 1.5197263 | 0.9054102 | 2.550853 | 1 |
| Zhong Qunhua 2024 | CT VS TF | 0.01999631 | -0.3692762 | 0.4092689 | 1.0201976 | 0.6912345 | 1.505716 | 1 |
| Zhong Qunhua 2024 | CT VS TUINA | 0.48288756 | 0.1919514 | 0.7738237 | 1.6207477 | 1.211612 | 2.16804 | 1 |
| Liu Wenlin 2024 | CT VS ACU | 0.04691796 | -0.3085858 | 0.4024217 | 1.048036 | 0.7344849 | 1.495442 | 1 |
| Liu Wenlin 2024 | CT VS CM | 0.4728861 | 0.2048412 | 0.740931 | 1.6046186 | 1.22733 | 2.097888 | 1 |
| Liu Wenlin 2024 | CT VS HM | 0.67775934 | 0.3399048 | 1.015614 | 1.9694599 | 1.404814 | 2.761058 | 1 |
| Liu Wenlin 2024 | CT VS REHAB | 0.16692557 | -0.8106376 | 1.144489 | 1.1816663 | 0.4445745 | 3.140835 | 1 |
| Liu Wenlin 2024 | CT VS TCM | 0.42643543 | -0.0918482 | 0.9447191 | 1.5317876 | 0.9122436 | 2.572091 | 1 |
| Liu Wenlin 2024 | CT VS TF | 0.51082563 | 0.1616043 | 0.860047 | 1.6666667 | 1.175395 | 2.363272 | 1 |
| Liu Wenlin 2024 | CT VS TUINA | 0.01846395 | -0.3708298 | 0.4077577 | 1.0186355 | 0.6901614 | 1.503443 | 1 |
| Liu Wenlin 2024 | CT VS WN | 0.46839726 | 0.1751593 | 0.7616352 | 1.5974319 | 1.191436 | 2.141776 | 1 |
| Wu Yusheng 2025 | CT VS ACU | 0.18129893 | -0.1602147 | 0.5228126 | 1.1987735 | 0.8519608 | 1.686765 | 1 |
| Wu Yusheng 2025 | CT VS CM | 0.54514319 | 0.2497032 | 0.8405831 | 1.7248553 | 1.283644 | 2.317718 | 1 |
| Wu Yusheng 2025 | CT VS HM | 0.85355826 | 0.4144925 | 1.292624 | 2.3479868 | 1.513602 | 3.642332 | 1 |
| Wu Yusheng 2025 | CT VS REHAB | 0.34272133 | -0.6742644 | 1.359707 | 1.4087761 | 0.5095311 | 3.895052 | 1 |
| Wu Yusheng 2025 | CT VS TCM | 0.60223137 | 0.0129536 | 1.191509 | 1.8261892 | 1.013038 | 3.292046 | 1 |
| Wu Yusheng 2025 | CT VS TF | 0.51082562 | 0.1616043 | 0.860047 | 1.6666667 | 1.175395 | 2.363272 | 1 |
| Wu Yusheng 2025 | CT VS TUINA | 0.07960779 | -0.3202111 | 0.4794267 | 1.0828623 | 0.7259957 | 1.615148 | 1 |
| Wu Yusheng 2025 | CT VS WN | 0.51797127 | 0.2221226 | 0.8138199 | 1.6786187 | 1.248724 | 2.256511 | 1 |
| Yao Dong 2024 | CT VS ACU | 0.18135054 | -0.1601714 | 0.5228725 | 1.1988353 | 0.8519977 | 1.686866 | 1 |
| Yao Dong 2024 | CT VS CM | 0.54520072 | 0.2497488 | 0.8406526 | 1.7249546 | 1.283703 | 2.317879 | 1 |
| Yao Dong 2024 | CT VS HM | 0.40543827 | -0.1212697 | 0.9321463 | 1.4999597 | 0.8857951 | 2.539955 | 1 |
| Yao Dong 2024 | CT VS REHAB | -0.10539062 | -1.163171 | 0.9523898 | 0.8999729 | 0.3124937 | 2.591896 | 1 |
| Yao Dong 2024 | CT VS TCM | 0.15411897 | -0.5030628 | 0.8113008 | 1.1666297 | 0.6046758 | 2.250834 | 1 |
| Yao Dong 2024 | CT VS TF | 0.51082562 | 0.1616043 | 0.860047 | 1.6666667 | 1.175395 | 2.363272 | 1 |
| Yao Dong 2024 | CT VS TUINA | 0.07964903 | -0.3201744 | 0.4794725 | 1.0829069 | 0.7260224 | 1.615222 | 1 |
| Yao Dong 2024 | CT VS WN | 0.51799553 | 0.2221448 | 0.8138463 | 1.6786595 | 1.248752 | 2.256571 | 1 |
| Guo Hui 2023 | CT VS ACU | 0.10662646 | -0.2151982 | 0.4284511 | 1.1125186 | 0.8063816 | 1.534878 | 1 |
| Guo Hui 2023 | CT VS CM | 0.4619823 | 0.1953624 | 0.7286022 | 1.5872172 | 1.215752 | 2.072182 | 1 |
| Guo Hui 2023 | CT VS HM | 0.66987264 | 0.3326052 | 1.00714 | 1.9539884 | 1.394597 | 2.73776 | 1 |
| Guo Hui 2023 | CT VS REHAB | 0.15903901 | -0.8183213 | 1.136399 | 1.1723837 | 0.4411716 | 3.11553 | 1 |
| Guo Hui 2023 | CT VS TCM | 0.51082562 | 0.1616043 | 0.860047 | 1.6666667 | 1.175395 | 2.363272 | 1 |
| Guo Hui 2023 | CT VS TF | 0.02000238 | -0.3692707 | 0.4092754 | 1.0202038 | 0.6912383 | 1.505726 | 1 |
| Guo Hui 2023 | CT VS TUINA | 0.48289111 | 0.1919547 | 0.7738275 | 1.6207534 | 1.211616 | 2.168049 | 1 |
| Wen Qi 2023 | CT VS ACU | 0.16134634 | -0.2029169 | 0.5256096 | 1.1750919 | 0.816346 | 1.69149 | 1 |
| Wen Qi 2023 | CT VS CM | 0.49094284 | 0.2094404 | 0.7724453 | 1.633856 | 1.232988 | 2.165054 | 1 |
| Wen Qi 2023 | CT VS HM | 0.69084003 | 0.3472896 | 1.03439 | 1.995391 | 1.415226 | 2.813391 | 1 |
| Wen Qi 2023 | CT VS REHAB | 0.18000602 | -0.7995402 | 1.159552 | 1.1972246 | 0.4495356 | 3.188505 | 1 |
| Wen Qi 2023 | CT VS TCM | 0.4395159 | -0.0824985 | 0.9615303 | 1.5519557 | 0.9208128 | 2.615696 | 1 |
| Wen Qi 2023 | CT VS TF | 0.51082562 | 0.1616043 | 0.860047 | 1.6666667 | 1.175395 | 2.363272 | 1 |
| Wen Qi 2023 | CT VS TUINA | 0.01590278 | -0.3735802 | 0.4053858 | 1.0160299 | 0.6882657 | 1.499881 | 1 |
| Wen Qi 2023 | CT VS WN | 0.44436772 | 0.1296059 | 0.7591296 | 1.5595038 | 1.13838 | 2.136416 | 1 |
| Wang Guoshu 2017 | CT VS ACU | 0.04999339 | -0.3021338 | 0.4021206 | 1.0512641 | 0.7392392 | 1.494992 | 1 |
| Wang Guoshu 2017 | CT VS CM | 0.4723265 | 0.2044297 | 0.7402233 | 1.6037209 | 1.226825 | 2.096404 | 1 |
| Wang Guoshu 2017 | CT VS HM | 0.67735383 | 0.3395609 | 1.015147 | 1.9686614 | 1.404331 | 2.759768 | 1 |
| Wang Guoshu 2017 | CT VS REHAB | 0.16652006 | -0.8110218 | 1.144062 | 1.1811872 | 0.4444038 | 3.139495 | 1 |
| Wang Guoshu 2017 | CT VS TCM | 0.42602992 | -0.0922135 | 0.9442734 | 1.5311666 | 0.9119104 | 2.570945 | 1 |
| Wang Guoshu 2017 | CT VS TF | 0.51082562 | 0.1616043 | 0.860047 | 1.6666667 | 1.175395 | 2.363272 | 1 |
| Wang Guoshu 2017 | CT VS TUINA | 0.0185444 | -0.3707475 | 0.4078363 | 1.0187174 | 0.6902182 | 1.503561 | 1 |
| Wang Guoshu 2017 | CT VS WN | 0.46914415 | 0.1761463 | 0.762142 | 1.5986254 | 1.192613 | 2.142861 | 1 |
| Sun Jian 2009 | CT VS ACU | 0.13415254 | -0.191824 | 0.460129 | 1.1435672 | 0.8254521 | 1.584278 | 1 |
| Sun Jian 2009 | CT VS CM | 0.45694861 | 0.1901613 | 0.7237359 | 1.5792477 | 1.209445 | 2.062123 | 1 |
| Sun Jian 2009 | CT VS HM | 0.66621352 | 0.3288817 | 1.003545 | 1.9468516 | 1.389414 | 2.727936 | 1 |
| Sun Jian 2009 | CT VS REHAB | 0.15537996 | -0.8220026 | 1.132763 | 1.1681017 | 0.4395505 | 3.10422 | 1 |
| Sun Jian 2009 | CT VS TCM | 0.41488981 | -0.1030532 | 0.9328328 | 1.5142039 | 0.902079 | 2.541699 | 1 |
| Sun Jian 2009 | CT VS TF | 0.51082562 | 0.1616043 | 0.860047 | 1.6666667 | 1.175395 | 2.363272 | 1 |
| Sun Jian 2009 | CT VS TUINA | 0.02070746 | -0.3685677 | 0.4099826 | 1.0209233 | 0.6917244 | 1.506792 | 1 |
| Sun Jian 2009 | CT VS WN | 0.48957168 | 0.1983631 | 0.7807803 | 1.6316172 | 1.219405 | 2.183175 | 1 |
| Sun Jianbing 2025 | CT VS ACU | 0.06525006 | -0.3116212 | 0.4421213 | 1.0674259 | 0.7322589 | 1.556005 | 1 |
| Sun Jianbing 2025 | CT VS CM | 0.41590241 | 0.0712458 | 0.760559 | 1.5157379 | 1.073845 | 2.139472 | 1 |
| Sun Jianbing 2025 | CT VS HM | 0.63647833 | 0.2639465 | 1.00901 | 1.8898139 | 1.302059 | 2.742885 | 1 |
| Sun Jianbing 2025 | CT VS REHAB | 0.1256453 | -0.864437 | 1.115728 | 1.1338799 | 0.4212886 | 3.051788 | 1 |
| Sun Jianbing 2025 | CT VS TCM | 0.38515512 | -0.1563717 | 0.926682 | 1.4698423 | 0.8552412 | 2.526114 | 1 |
| Sun Jianbing 2025 | CT VS TF | 0.51082562 | 0.1616043 | 0.860047 | 1.6666667 | 1.175395 | 2.363272 | 1 |
| Sun Jianbing 2025 | CT VS TUINA | -0.01302531 | -0.4325958 | 0.4065452 | 0.98705916 | 0.6488227 | 1.501621 | 1 |
| Sun Jianbing 2025 | CT VS WN | 0.46345301 | 0.1582772 | 0.7686288 | 1.5895533 | 1.171491 | 2.156807 | 1 |
| Xu Chenghua 2018 | CT VS ACU | 0.08871905 | -0.2361723 | 0.4136104 | 1.0927736 | 0.7896446 | 1.512268 | 1 |
| Xu Chenghua 2018 | CT VS CM | 0.43156675 | 0.1544267 | 0.7087068 | 1.5396679 | 1.166989 | 2.031363 | 1 |
| Xu Chenghua 2018 | CT VS HM | 0.647826 | 0.306142 | 0.98951 | 1.911381 | 1.358175 | 2.689916 | 1 |
| Xu Chenghua 2018 | CT VS REHAB | 0.13699277 | -0.8419004 | 1.115886 | 1.1468199 | 0.4308909 | 3.052271 | 1 |
| Xu Chenghua 2018 | CT VS TCM | 0.3965026 | -0.1242853 | 0.9172905 | 1.4866163 | 0.8831278 | 2.502501 | 1 |
| Xu Chenghua 2018 | CT VS TF | 0.51082562 | 0.1616043 | 0.860047 | 1.6666667 | 1.175395 | 2.363272 | 1 |
| Xu Chenghua 2018 | CT VS TUINA | 0.17193264 | -0.3705247 | 0.71439 | 1.1875978 | 0.690372 | 2.04294 | 1 |
| Xu Chenghua 2018 | CT VS WN | 0.52333558 | 0.2155067 | 0.8311645 | 1.6876476 | 1.24049 | 2.295991 | 1 |
| Hua Yuqi 2015 | CT VS ACU | 0.10662013 | -0.2152037 | 0.428444 | 1.1125116 | 0.8063772 | 1.534867 | 1 |
| Hua Yuqi 2015 | CT VS CM | 0.46197526 | 0.1953565 | 0.728594 | 1.587206 | 1.215744 | 2.072165 | 1 |
| Hua Yuqi 2015 | CT VS HM | 0.66985707 | 0.3325942 | 1.00712 | 1.953958 | 1.394581 | 2.737705 | 1 |
| Hua Yuqi 2015 | CT VS REHAB | 0.4185333 | -0.0993648 | 0.9364314 | 1.5197309 | 0.9054123 | 2.550862 | 1 |
| Hua Yuqi 2015 | CT VS TCM | 0.51082562 | 0.1616043 | 0.860047 | 1.6666667 | 1.175395 | 2.363272 | 1 |
| Hua Yuqi 2015 | CT VS TF | 0.01999733 | -0.3692753 | 0.40927 | 1.0201986 | 0.6912351 | 1.505718 | 1 |
| Hua Yuqi 2015 | CT VS TUINA | 0.48288814 | 0.191952 | 0.7738243 | 1.6207486 | 1.211612 | 2.168042 | 1 |
| Wu Zongxiang 2017 | CT VS ACU | 0.08871646 | -0.2361725 | 0.4136055 | 1.0927708 | 0.7896444 | 1.51226 | 1 |
| Wu Zongxiang 2017 | CT VS CM | 0.43156238 | 0.1544263 | 0.7086985 | 1.5396612 | 1.166988 | 2.031346 | 1 |
| Wu Zongxiang 2017 | CT VS HM | 0.64782293 | 0.3061406 | 0.9895052 | 1.9113751 | 1.358173 | 2.689903 | 1 |
| Wu Zongxiang 2017 | CT VS REHAB | 0.13698969 | -0.8419029 | 1.115882 | 1.1468163 | 0.4308898 | 3.05226 | 1 |
| Wu Zongxiang 2017 | CT VS TCM | 0.39649952 | -0.1242873 | 0.9172863 | 1.4866117 | 0.8831261 | 2.50249 | 1 |
| Wu Zongxiang 2017 | CT VS TF | 0.51082562 | 0.1616043 | 0.860047 | 1.6666667 | 1.175395 | 2.363272 | 1 |
| Wu Zongxiang 2017 | CT VS TUINA | -0.11748734 | -0.6355641 | 0.4005894 | 0.88915176 | 0.5296367 | 1.492704 | 1 |
| Wu Zongxiang 2017 | CT VS WN | 0.52334071 | 0.2155092 | 0.8311722 | 1.6876562 | 1.240493 | 2.296009 | 1 |
| Li Xiuxia 2019 | CT VS ACU | 0.09709046 | -0.2810013 | 0.4751823 | 1.1019601 | 0.7550273 | 1.608307 | 1 |
| Li Xiuxia 2019 | CT VS CM | 0.4547648 | 0.1487756 | 0.760754 | 1.5758027 | 1.160413 | 2.139889 | 1 |
| Li Xiuxia 2019 | CT VS HM | 0.66463144 | 0.3102636 | 1.018999 | 1.943774 | 1.363785 | 2.770421 | 1 |
| Li Xiuxia 2019 | CT VS REHAB | 0.15379791 | -0.8295943 | 1.13719 | 1.1662552 | 0.4362262 | 3.117995 | 1 |
| Li Xiuxia 2019 | CT VS TCM | 0.41330776 | -0.1158882 | 0.9425038 | 1.5118102 | 0.8905748 | 2.566399 | 1 |
| Li Xiuxia 2019 | CT VS TF | 0.51082562 | 0.1616043 | 0.860047 | 1.6666667 | 1.175395 | 2.363272 | 1 |
| Li Xiuxia 2019 | CT VS TUINA | 0.00635004 | -0.4756382 | 0.4883383 | 1.0063702 | 0.6214883 | 1.629606 | 1 |
| Li Xiuxia 2019 | CT VS WN | 0.46253815 | -0.0515364 | 0.9766126 | 1.5880997 | 0.9497691 | 2.655446 | 1 |

Grade assessment

**GRADE assessment based on the ODI score group in the primary analysis of patients with LDH. (Table S58)**

| **Comparison** | **Number of studies** | **Within-study bias** | **Reporting bias** | **Indirectness** | **Imprecision** | **Heterogeneity** | **Incoherence** | **Confidence rating** | **Reason(s) for downgrading** |
| --- | --- | --- | --- | --- | --- | --- | --- | --- | --- |
| ACU:ACU+MOXI | 3 | Major concerns | Low risk | No concerns | No concerns | No concerns | No concerns | Low | ["Within-study bias"] |
| ACU+MOXI:CT | 1 | Some concerns | Low risk | No concerns | No concerns | No concerns | No concerns | Moderate | ["Within-study bias"] |
| CT:CT+MOXI | 1 | Major concerns | Low risk | No concerns | No concerns | No concerns | No concerns | Low | ["Within-study bias"] |
| CT:MOXI | 1 | Major concerns | Low risk | No concerns | No concerns | No concerns | No concerns | Low | ["Within-study bias"] |
| CT:TCM+MOXI | 4 | Some concerns | Low risk | No concerns | No concerns | No concerns | No concerns | Moderate | ["Within-study bias"] |
| CT+MOXI:TUINA+MOXI | 1 | Some concerns | Low risk | No concerns | No concerns | No concerns | No concerns | Moderate | ["Within-study bias"] |
| MOXI:TCM+MOXI | 1 | Some concerns | Low risk | No concerns | No concerns | No concerns | No concerns | Moderate | ["Within-study bias"] |
| MOXI:TUINA+MOXI | 1 | Major concerns | Low risk | No concerns | No concerns | No concerns | No concerns | Low | ["Within-study bias"] |
| REHAB:TUINA+MOXI | 1 | Major concerns | Low risk | No concerns | No concerns | No concerns | No concerns | Low | ["Within-study bias"] |
| TCM:TCM+MOXI | 2 | No concerns | Low risk | No concerns | No concerns | No concerns | No concerns | High | [] |
| ACU:CT | 0 | Some concerns | Low risk | No concerns | No concerns | No concerns | No concerns | Moderate | ["Within-study bias"] |
| ACU:CT+MOXI | 0 | Major concerns | Low risk | No concerns | No concerns | No concerns | No concerns | Low | ["Within-study bias"] |
| ACU:MOXI | 0 | Some concerns | Low risk | No concerns | No concerns | No concerns | No concerns | Moderate | ["Within-study bias"] |
| ACU:REHAB | 0 | Major concerns | Low risk | No concerns | No concerns | No concerns | No concerns | Low | ["Within-study bias"] |
| ACU:TCM | 0 | Some concerns | Low risk | No concerns | No concerns | Some concerns | No concerns | Low | ["Within-study bias","Heterogeneity"] |
| ACU:TCM+MOXI | 0 | Some concerns | Low risk | No concerns | No concerns | No concerns | No concerns | Moderate | ["Within-study bias"] |
| ACU:TUINA+MOXI | 0 | Major concerns | Low risk | No concerns | No concerns | No concerns | No concerns | Low | ["Within-study bias"] |
| ACU+MOXI:CT+MOXI | 0 | Some concerns | Low risk | No concerns | No concerns | No concerns | No concerns | Moderate | ["Within-study bias"] |
| ACU+MOXI:MOXI | 0 | Some concerns | Low risk | No concerns | No concerns | Some concerns | No concerns | Low | ["Within-study bias","Heterogeneity"] |
| ACU+MOXI:REHAB | 0 | Major concerns | Low risk | No concerns | No concerns | No concerns | No concerns | Low | ["Within-study bias"] |
| ACU+MOXI:TCM | 0 | Some concerns | Low risk | No concerns | No concerns | No concerns | No concerns | Moderate | ["Within-study bias"] |
| ACU+MOXI:TCM+MOXI | 0 | Some concerns | Low risk | No concerns | No concerns | No concerns | No concerns | Moderate | ["Within-study bias"] |
| ACU+MOXI:TUINA+MOXI | 0 | Some concerns | Low risk | No concerns | No concerns | No concerns | No concerns | Moderate | ["Within-study bias"] |
| CT:REHAB | 0 | Major concerns | Low risk | No concerns | No concerns | No concerns | No concerns | Low | ["Within-study bias"] |
| CT:TCM | 0 | Some concerns | Low risk | No concerns | No concerns | No concerns | No concerns | Moderate | ["Within-study bias"] |
| CT:TUINA+MOXI | 0 | Major concerns | Low risk | No concerns | No concerns | No concerns | No concerns | Low | ["Within-study bias"] |
| CT+MOXI:MOXI | 0 | Major concerns | Low risk | No concerns | No concerns | No concerns | No concerns | Low | ["Within-study bias"] |
| CT+MOXI:REHAB | 0 | Major concerns | Low risk | No concerns | No concerns | No concerns | No concerns | Low | ["Within-study bias"] |
| CT+MOXI:TCM | 0 | Some concerns | Low risk | No concerns | No concerns | No concerns | No concerns | Moderate | ["Within-study bias"] |
| CT+MOXI:TCM+MOXI | 0 | Some concerns | Low risk | No concerns | No concerns | No concerns | No concerns | Moderate | ["Within-study bias"] |
| MOXI:REHAB | 0 | Major concerns | Low risk | No concerns | No concerns | No concerns | No concerns | Low | ["Within-study bias"] |
| MOXI:TCM | 0 | Some concerns | Low risk | No concerns | No concerns | No concerns | No concerns | Moderate | ["Within-study bias"] |
| REHAB:TCM | 0 | Major concerns | Low risk | No concerns | No concerns | No concerns | No concerns | Low | ["Within-study bias"] |
| REHAB:TCM+MOXI | 0 | Major concerns | Low risk | No concerns | No concerns | No concerns | No concerns | Low | ["Within-study bias"] |
| TCM:TUINA+MOXI | 0 | Some concerns | Low risk | No concerns | No concerns | No concerns | No concerns | Moderate | ["Within-study bias"] |
| TCM+MOXI:TUINA+MOXI | 0 | Some concerns | Low risk | No concerns | No concerns | No concerns | No concerns | Moderate | ["Within-study bias"] |

**GRADE assessment based on the VAS score group in the primary analysis of patients with LDH.(Table S59)**

| **Comparison** | **Number of studies** | **Within-study bias** | **Reporting bias** | **Indirectness** | **Imprecision** | **Heterogeneity** | **Incoherence** | **Confidence rating** | | **Reason(s) for downgrading** |
| --- | --- | --- | --- | --- | --- | --- | --- | --- | --- | --- |
| ACU:ACU+MOXI | 8 | Some concerns | Low risk | No concerns | No concerns | Some concerns | No concerns | Low | ["Within-study bias","Heterogeneity"] | |
| ACU:MOXI | 2 | Some concerns | Low risk | No concerns | Some concerns | Some concerns | No concerns | Very low | ["Within-study bias","Imprecision","Heterogeneity"] | |
| ACU:TCM+MOXI | 2 | Some concerns | Low risk | No concerns | No concerns | Some concerns | No concerns | Low | ["Within-study bias","Heterogeneity"] | |
| ACU+MOXI:CT | 2 | Some concerns | Low risk | No concerns | No concerns | Some concerns | No concerns | Low | ["Within-study bias","Heterogeneity"] | |
| CT:CT+MOXI | 3 | Some concerns | Low risk | No concerns | No concerns | Some concerns | No concerns | Low | ["Within-study bias","Heterogeneity"] | |
| CT:MOXI | 3 | Some concerns | Low risk | No concerns | No concerns | Some concerns | No concerns | Low | ["Within-study bias","Heterogeneity"] | |
| CT:TCM+MOXI | 6 | Some concerns | Low risk | No concerns | No concerns | Some concerns | No concerns | Low | ["Within-study bias","Heterogeneity"] | |
| CT:TUINA+MOXI | 1 | Major concerns | Low risk | No concerns | No concerns | Some concerns | No concerns | Very low | ["Within-study bias","Heterogeneity"] | |
| CT+MOXI:TUINA+MOXI | 1 | Some concerns | Low risk | No concerns | No concerns | Some concerns | No concerns | Low | ["Within-study bias","Heterogeneity"] | |
| MOXI:TCM+MOXI | 1 | Some concerns | Low risk | No concerns | No concerns | Some concerns | No concerns | Low | ["Within-study bias","Heterogeneity"] | |
| MOXI:TUINA+MOXI | 1 | Some concerns | Low risk | No concerns | Some concerns | Some concerns | No concerns | Very low | ["Within-study bias","Imprecision","Heterogeneity"] | |
| REHAB:TCM+MOXI | 1 | Major concerns | Low risk | No concerns | Some concerns | No concerns | No concerns | Very low | ["Within-study bias","Imprecision"] | |
| REHAB:TUINA+MOXI | 4 | Some concerns | Low risk | No concerns | No concerns | Some concerns | No concerns | Low | ["Within-study bias","Heterogeneity"] | |
| TCM:TCM+MOXI | 2 | No concerns | Low risk | No concerns | No concerns | Some concerns | No concerns | Moderate | ["Heterogeneity"] | |
| TUINA:TUINA+MOXI | 3 | Major concerns | Low risk | No concerns | No concerns | Some concerns | No concerns | Very low | ["Within-study bias","Heterogeneity"] | |
| ACU:CT | 0 | Some concerns | Low risk | No concerns | No concerns | Some concerns | No concerns | Low | ["Within-study bias","Heterogeneity"] | |
| ACU:CT+MOXI | 0 | Some concerns | Low risk | No concerns | Some concerns | No concerns | No concerns | Low | ["Within-study bias","Imprecision"] | |
| ACU:REHAB | 0 | Some concerns | Low risk | No concerns | Some concerns | Some concerns | No concerns | Very low | ["Within-study bias","Imprecision","Heterogeneity"] | |
| ACU:TCM | 0 | Some concerns | Low risk | No concerns | Some concerns | Some concerns | No concerns | Very low | ["Within-study bias","Imprecision","Heterogeneity"] | |
| ACU:TUINA | 0 | Some concerns | Low risk | No concerns | Some concerns | Some concerns | No concerns | Very low | ["Within-study bias","Imprecision","Heterogeneity"] | |
| ACU:TUINA+MOXI | 0 | Some concerns | Low risk | No concerns | Some concerns | No concerns | No concerns | Low | ["Within-study bias","Imprecision"] | |
| ACU+MOXI:CT+MOXI | 0 | Some concerns | Low risk | No concerns | No concerns | Some concerns | No concerns | Low | ["Within-study bias","Heterogeneity"] | |
| ACU+MOXI:MOXI | 0 | Some concerns | Low risk | No concerns | No concerns | Some concerns | No concerns | Low | ["Within-study bias","Heterogeneity"] | |
| ACU+MOXI:REHAB | 0 | Some concerns | Low risk | No concerns | Some concerns | No concerns | No concerns | Low | ["Within-study bias","Imprecision"] | |
| ACU+MOXI:TCM | 0 | Some concerns | Low risk | No concerns | No concerns | Some concerns | No concerns | Low | ["Within-study bias","Heterogeneity"] | |
| ACU+MOXI:TCM+MOXI | 0 | Some concerns | Low risk | No concerns | No concerns | Major concerns | No concerns | Very low | ["Within-study bias","Heterogeneity"] | |
| ACU+MOXI:TUINA | 0 | Some concerns | Low risk | No concerns | Some concerns | No concerns | No concerns | Low | ["Within-study bias","Imprecision"] | |
| ACU+MOXI:TUINA+MOXI | 0 | Some concerns | Low risk | No concerns | No concerns | Some concerns | No concerns | Low | ["Within-study bias","Heterogeneity"] | |
| CT:REHAB | 0 | Some concerns | Low risk | No concerns | No concerns | Some concerns | No concerns | Low | ["Within-study bias","Heterogeneity"] | |
| CT:TCM | 0 | Some concerns | Low risk | No concerns | Some concerns | Some concerns | No concerns | Very low | ["Within-study bias","Imprecision","Heterogeneity"] | |
| CT:TUINA | 0 | Major concerns | Low risk | No concerns | Some concerns | Some concerns | No concerns | Very low | ["Within-study bias","Imprecision","Heterogeneity"] | |
| CT+MOXI:MOXI | 0 | Some concerns | Low risk | No concerns | Some concerns | Some concerns | No concerns | Very low | ["Within-study bias","Imprecision","Heterogeneity"] | |
| CT+MOXI:REHAB | 0 | Some concerns | Low risk | No concerns | No concerns | Some concerns | No concerns | Low | ["Within-study bias","Heterogeneity"] | |
| CT+MOXI:TCM | 0 | Some concerns | Low risk | No concerns | No concerns | Some concerns | No concerns | Low | ["Within-study bias","Heterogeneity"] | |
| CT+MOXI:TCM+MOXI | 0 | Some concerns | Low risk | No concerns | Some concerns | Some concerns | No concerns | Very low | ["Within-study bias","Imprecision","Heterogeneity"] | |
| CT+MOXI:TUINA | 0 | Some concerns | Low risk | No concerns | Some concerns | No concerns | No concerns | Low | ["Within-study bias","Imprecision"] | |
| MOXI:REHAB | 0 | Some concerns | Low risk | No concerns | Some concerns | No concerns | No concerns | Low | ["Within-study bias","Imprecision"] | |
| MOXI:TCM | 0 | Some concerns | Low risk | No concerns | Some concerns | No concerns | No concerns | Low | ["Within-study bias","Imprecision"] | |
| MOXI:TUINA | 0 | Some concerns | Low risk | No concerns | Some concerns | Some concerns | No concerns | Very low | ["Within-study bias","Imprecision","Heterogeneity"] | |
| REHAB:TCM | 0 | Some concerns | Low risk | No concerns | Major concerns | No concerns | No concerns | Very low | ["Within-study bias","Imprecision"] | |
| REHAB:TUINA | 0 | Major concerns | Low risk | No concerns | Some concerns | Some concerns | No concerns | Very low | ["Within-study bias","Imprecision","Heterogeneity"] | |
| TCM:TUINA | 0 | Some concerns | Low risk | No concerns | Some concerns | Some concerns | No concerns | Very low | ["Within-study bias","Imprecision","Heterogeneity"] | |
| TCM:TUINA+MOXI | 0 | Some concerns | Low risk | No concerns | No concerns | Some concerns | No concerns | Low | ["Within-study bias","Heterogeneity"] | |
| TCM+MOXI:TUINA | 0 | Some concerns | Low risk | No concerns | Some concerns | Some concerns | No concerns | Very low | ["Within-study bias","Imprecision","Heterogeneity"] | |
| TCM+MOXI:TUINA+MOXI | 0 | Some concerns | Low risk | No concerns | Some concerns | Some concerns | No concerns | Very low | ["Within-study bias","Imprecision","Heterogeneity"] | |

**GRADE assessment based on the JOA score group in the primary analysis of patients with LDH. (Table S60)**

| **Comparison** | **Number of studies** | **Within-study bias** | **Reporting bias** | **Indirectness** | **Imprecision** | **Heterogeneity** | **Incoherence** | **Confidence rating** | **Reason(s) for downgrading** |
| --- | --- | --- | --- | --- | --- | --- | --- | --- | --- |
| ACU:ACU+MOXI | 5 | Some concerns | Low risk | No concerns | No concerns | No concerns | No concerns | Moderate | ["Within-study bias"] |
| ACU:MOXI | 2 | Some concerns | Low risk | No concerns | No concerns | No concerns | No concerns | Moderate | ["Within-study bias"] |
| ACU:TCM+MOXI | 2 | Major concerns | Low risk | No concerns | No concerns | No concerns | No concerns | Low | ["Within-study bias"] |
| CT:CT+MOXI | 1 | Some concerns | Low risk | No concerns | No concerns | No concerns | No concerns | Moderate | ["Within-study bias"] |
| CT:MOXI | 4 | Major concerns | Low risk | No concerns | No concerns | No concerns | No concerns | Low | ["Within-study bias"] |
| CT:TCM+MOXI | 4 | Major concerns | Low risk | No concerns | No concerns | No concerns | No concerns | Low | ["Within-study bias"] |
| MOXI:TUINA+MOXI | 1 | Major concerns | Low risk | No concerns | No concerns | No concerns | No concerns | Low | ["Within-study bias"] |
| REHAB:TUINA+MOXI | 3 | Some concerns | Low risk | No concerns | No concerns | No concerns | No concerns | Moderate | ["Within-study bias"] |
| TCM:TCM+MOXI | 3 | Some concerns | Low risk | No concerns | No concerns | No concerns | No concerns | Moderate | ["Within-study bias"] |
| TUINA:TUINA+MOXI | 3 | Some concerns | Low risk | No concerns | No concerns | No concerns | No concerns | Moderate | ["Within-study bias"] |
| ACU:CT | 0 | Major concerns | Low risk | No concerns | No concerns | No concerns | No concerns | Low | ["Within-study bias"] |
| ACU:CT+MOXI | 0 | Some concerns | Low risk | No concerns | No concerns | No concerns | No concerns | Moderate | ["Within-study bias"] |
| ACU:REHAB | 0 | Major concerns | Low risk | No concerns | No concerns | No concerns | No concerns | Low | ["Within-study bias"] |
| ACU:TCM | 0 | Major concerns | Low risk | No concerns | No concerns | No concerns | No concerns | Low | ["Within-study bias"] |
| ACU:TUINA | 0 | Major concerns | Low risk | No concerns | No concerns | No concerns | No concerns | Low | ["Within-study bias"] |
| ACU:TUINA+MOXI | 0 | Major concerns | Low risk | No concerns | No concerns | No concerns | No concerns | Low | ["Within-study bias"] |
| ACU+MOXI:CT | 0 | Some concerns | Low risk | No concerns | No concerns | No concerns | No concerns | Moderate | ["Within-study bias"] |
| ACU+MOXI:CT+MOXI | 0 | Some concerns | Low risk | No concerns | No concerns | No concerns | No concerns | Moderate | ["Within-study bias"] |
| ACU+MOXI:MOXI | 0 | Some concerns | Low risk | No concerns | No concerns | No concerns | No concerns | Moderate | ["Within-study bias"] |
| ACU+MOXI:REHAB | 0 | Major concerns | Low risk | No concerns | No concerns | No concerns | No concerns | Low | ["Within-study bias"] |
| ACU+MOXI:TCM | 0 | Some concerns | Low risk | No concerns | No concerns | No concerns | No concerns | Moderate | ["Within-study bias"] |
| ACU+MOXI:TCM+MOXI | 0 | Major concerns | Low risk | No concerns | No concerns | No concerns | No concerns | Low | ["Within-study bias"] |
| ACU+MOXI:TUINA | 0 | Major concerns | Low risk | No concerns | No concerns | No concerns | No concerns | Low | ["Within-study bias"] |
| ACU+MOXI:TUINA+MOXI | 0 | Major concerns | Low risk | No concerns | No concerns | No concerns | No concerns | Low | ["Within-study bias"] |
| CT:REHAB | 0 | Major concerns | Low risk | No concerns | No concerns | No concerns | No concerns | Low | ["Within-study bias"] |
| CT:TCM | 0 | Some concerns | Low risk | No concerns | No concerns | No concerns | No concerns | Moderate | ["Within-study bias"] |
| CT:TUINA | 0 | Major concerns | Low risk | No concerns | No concerns | No concerns | No concerns | Low | ["Within-study bias"] |
| CT:TUINA+MOXI | 0 | Major concerns | Low risk | No concerns | No concerns | No concerns | No concerns | Low | ["Within-study bias"] |
| CT+MOXI:MOXI | 0 | Some concerns | Low risk | No concerns | No concerns | No concerns | No concerns | Moderate | ["Within-study bias"] |
| CT+MOXI:REHAB | 0 | Some concerns | Low risk | No concerns | No concerns | No concerns | No concerns | Moderate | ["Within-study bias"] |
| CT+MOXI:TCM | 0 | Some concerns | Low risk | No concerns | No concerns | No concerns | No concerns | Moderate | ["Within-study bias"] |
| CT+MOXI:TCM+MOXI | 0 | Some concerns | Low risk | No concerns | No concerns | No concerns | No concerns | Moderate | ["Within-study bias"] |
| CT+MOXI:TUINA | 0 | Some concerns | Low risk | No concerns | No concerns | Some concerns | No concerns | Low | ["Within-study bias","Heterogeneity"] |
| CT+MOXI:TUINA+MOXI | 0 | Major concerns | Low risk | No concerns | No concerns | No concerns | No concerns | Low | ["Within-study bias"] |
| MOXI:REHAB | 0 | Major concerns | Low risk | No concerns | No concerns | No concerns | No concerns | Low | ["Within-study bias"] |
| MOXI:TCM | 0 | Some concerns | Low risk | No concerns | No concerns | No concerns | No concerns | Moderate | ["Within-study bias"] |
| MOXI:TCM+MOXI | 0 | Major concerns | Low risk | No concerns | No concerns | No concerns | No concerns | Low | ["Within-study bias"] |
| MOXI:TUINA | 0 | Major concerns | Low risk | No concerns | No concerns | No concerns | No concerns | Low | ["Within-study bias"] |
| REHAB:TCM | 0 | Major concerns | Low risk | No concerns | No concerns | No concerns | No concerns | Low | ["Within-study bias"] |
| REHAB:TCM+MOXI | 0 | Major concerns | Low risk | No concerns | No concerns | No concerns | No concerns | Low | ["Within-study bias"] |
| REHAB:TUINA | 0 | Some concerns | Low risk | No concerns | No concerns | No concerns | No concerns | Moderate | ["Within-study bias"] |
| TCM:TUINA | 0 | Major concerns | Low risk | No concerns | No concerns | No concerns | No concerns | Low | ["Within-study bias"] |
| TCM:TUINA+MOXI | 0 | Major concerns | Low risk | No concerns | No concerns | Some concerns | No concerns | Very low | ["Within-study bias","Heterogeneity"] |
| TCM+MOXI:TUINA | 0 | Major concerns | Low risk | No concerns | No concerns | No concerns | No concerns | Low | ["Within-study bias"] |
| TCM+MOXI:TUINA+MOXI | 0 | Major concerns | Low risk | No concerns | No concerns | No concerns | No concerns | Low | ["Within-study bias"] |

**GRADE assessment based on the cure rate group in the primary analysis of patients with LDH.(Table S61)**

| **Comparison** | **Number of studies** | **Within-study bias** | **Reporting bias** | **Indirectness** | **Imprecision** | **Heterogeneity** | **Incoherence** | **Confidence rating** | **Reason(s) for downgrading** |
| --- | --- | --- | --- | --- | --- | --- | --- | --- | --- |
| ACU:ACU+MOXI | 3 | Some concerns | Low risk | No concerns | No concerns | Some concerns | No concerns | Low | ["Within-study bias","Heterogeneity"] |
| ACU:MOXI | 1 | Some concerns | Low risk | No concerns | No concerns | No concerns | No concerns | Moderate | ["Within-study bias"] |
| ACU:TCM+MOXI | 1 | Some concerns | Low risk | No concerns | No concerns | No concerns | No concerns | Moderate | ["Within-study bias"] |
| CT:CT+MOXI | 1 | Some concerns | Low risk | No concerns | No concerns | No concerns | No concerns | Moderate | ["Within-study bias"] |
| CT:MOXI | 1 | Some concerns | Low risk | No concerns | No concerns | Some concerns | Some concerns | Very low | ["Within-study bias","Heterogeneity","Incoherence"] |
| CT:TCM+MOXI | 3 | Some concerns | Low risk | No concerns | No concerns | No concerns | No concerns | Moderate | ["Within-study bias"] |
| CT+MOXI:TUINA+MOXI | 1 | Some concerns | Low risk | No concerns | No concerns | Some concerns | No concerns | Low | ["Within-study bias","Heterogeneity"] |
| MOXI:TUINA+MOXI | 1 | Some concerns | Low risk | No concerns | Some concerns | No concerns | No concerns | Low | ["Within-study bias","Imprecision"] |
| TCM:TCM+MOXI | 1 | Some concerns | Low risk | No concerns | Some concerns | Some concerns | No concerns | Very low | ["Within-study bias","Imprecision","Heterogeneity"] |
| TUINA:TUINA+MOXI | 2 | Some concerns | Low risk | No concerns | No concerns | No concerns | No concerns | Moderate | ["Within-study bias"] |
| ACU:CT | 0 | Some concerns | Low risk | No concerns | Major concerns | No concerns | No concerns | Very low | ["Within-study bias","Imprecision"] |
| ACU:CT+MOXI | 0 | Some concerns | Low risk | No concerns | No concerns | Some concerns | No concerns | Low | ["Within-study bias","Heterogeneity"] |
| ACU:TCM | 0 | Some concerns | Low risk | No concerns | Some concerns | Some concerns | No concerns | Very low | ["Within-study bias","Imprecision","Heterogeneity"] |
| ACU:TUINA | 0 | Some concerns | Low risk | No concerns | Some concerns | Some concerns | No concerns | Very low | ["Within-study bias","Imprecision","Heterogeneity"] |
| ACU:TUINA+MOXI | 0 | Some concerns | Low risk | No concerns | No concerns | No concerns | No concerns | Moderate | ["Within-study bias"] |
| ACU+MOXI:CT | 0 | Some concerns | Low risk | No concerns | Major concerns | No concerns | No concerns | Very low | ["Within-study bias","Imprecision"] |
| ACU+MOXI:CT+MOXI | 0 | Some concerns | Low risk | No concerns | Some concerns | Some concerns | No concerns | Very low | ["Within-study bias","Imprecision","Heterogeneity"] |
| ACU+MOXI:MOXI | 0 | Some concerns | Low risk | No concerns | Some concerns | Some concerns | No concerns | Very low | ["Within-study bias","Imprecision","Heterogeneity"] |
| ACU+MOXI:TCM | 0 | Some concerns | Low risk | No concerns | Major concerns | No concerns | No concerns | Very low | ["Within-study bias","Imprecision"] |
| ACU+MOXI:TCM+MOXI | 0 | Some concerns | Low risk | No concerns | Some concerns | Some concerns | No concerns | Very low | ["Within-study bias","Imprecision","Heterogeneity"] |
| ACU+MOXI:TUINA | 0 | Some concerns | Low risk | No concerns | Major concerns | No concerns | No concerns | Very low | ["Within-study bias","Imprecision"] |
| ACU+MOXI:TUINA+MOXI | 0 | Some concerns | Low risk | No concerns | No concerns | Some concerns | No concerns | Low | ["Within-study bias","Heterogeneity"] |
| CT:TCM | 0 | Some concerns | Low risk | No concerns | Some concerns | Some concerns | No concerns | Very low | ["Within-study bias","Imprecision","Heterogeneity"] |
| CT:TUINA | 0 | Some concerns | Low risk | No concerns | Some concerns | Some concerns | No concerns | Very low | ["Within-study bias","Imprecision","Heterogeneity"] |
| CT:TUINA+MOXI | 0 | Some concerns | Low risk | No concerns | No concerns | No concerns | No concerns | Moderate | ["Within-study bias"] |
| CT+MOXI:MOXI | 0 | Some concerns | Low risk | No concerns | Major concerns | No concerns | No concerns | Very low | ["Within-study bias","Imprecision"] |
| CT+MOXI:TCM | 0 | Some concerns | Low risk | No concerns | Major concerns | No concerns | No concerns | Very low | ["Within-study bias","Imprecision"] |
| CT+MOXI:TCM+MOXI | 0 | Some concerns | Low risk | No concerns | Major concerns | No concerns | No concerns | Very low | ["Within-study bias","Imprecision"] |
| CT+MOXI:TUINA | 0 | Some concerns | Low risk | No concerns | Major concerns | No concerns | No concerns | Very low | ["Within-study bias","Imprecision"] |
| MOXI:TCM | 0 | Some concerns | Low risk | No concerns | Major concerns | No concerns | No concerns | Very low | ["Within-study bias","Imprecision"] |
| MOXI:TCM+MOXI | 0 | Some concerns | Low risk | No concerns | Major concerns | No concerns | No concerns | Very low | ["Within-study bias","Imprecision"] |
| MOXI:TUINA | 0 | Some concerns | Low risk | No concerns | Major concerns | No concerns | No concerns | Very low | ["Within-study bias","Imprecision"] |
| TCM:TUINA | 0 | Some concerns | Low risk | No concerns | Major concerns | No concerns | No concerns | Very low | ["Within-study bias","Imprecision"] |
| TCM:TUINA+MOXI | 0 | Some concerns | Low risk | No concerns | Some concerns | Some concerns | No concerns | Very low | ["Within-study bias","Imprecision","Heterogeneity"] |
| TCM+MOXI:TUINA | 0 | Some concerns | Low risk | No concerns | Major concerns | No concerns | No concerns | Very low | ["Within-study bias","Imprecision"] |
| TCM+MOXI:TUINA+MOXI | 0 | Some concerns | Low risk | No concerns | Some concerns | Some concerns | No concerns | Very low | ["Within-study bias","Imprecision","Heterogeneity"] |

**
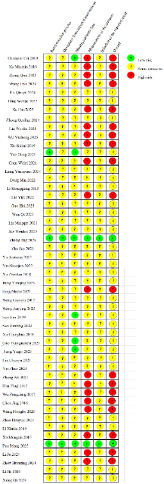
**

Figure1: Result of the risk of bias assessment using the RoB 2 tool**.
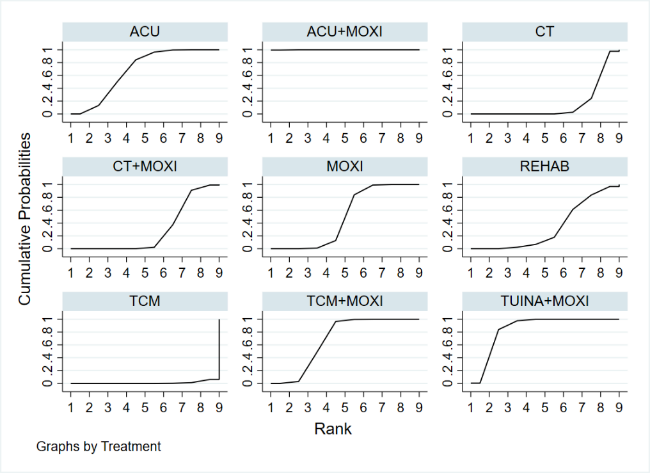
**

Figure2: Rank plot based on the ODI score group in the primary analysis of patients with LDH.


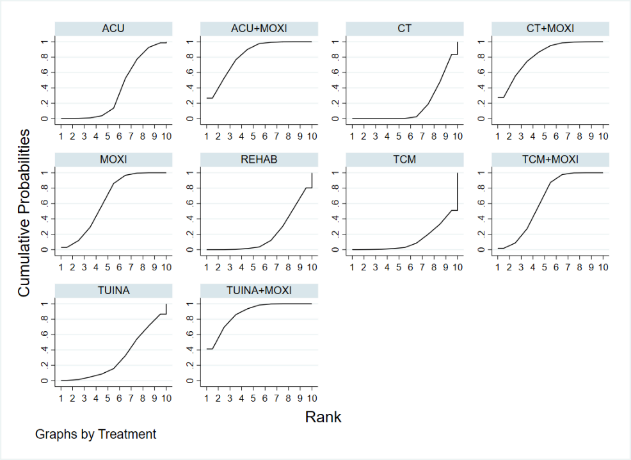


Figure3: Rank plot based on the VAS score group in the primary analysis of patients with LDH.


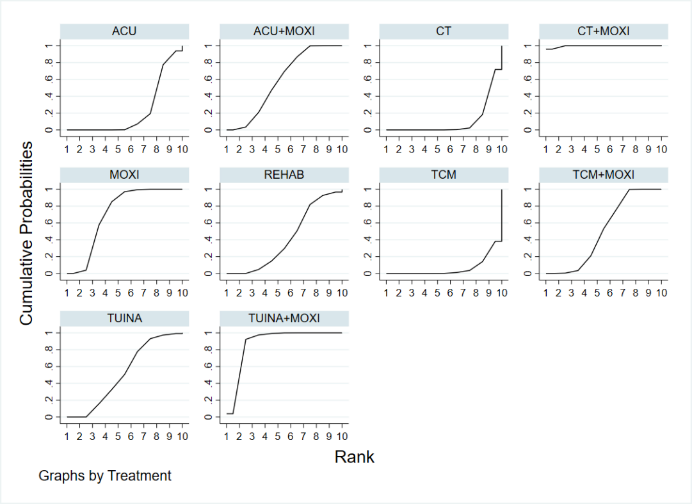


Figure4: Rank plot based on the JOA score group in the primary analysis of patients with LDH.

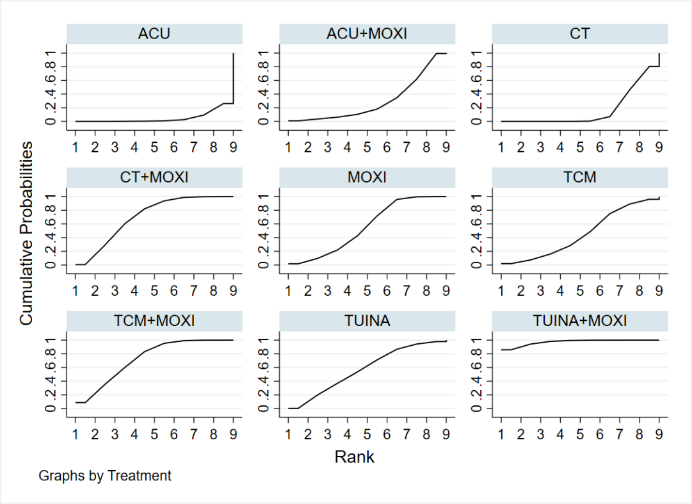


Figure5: Rank plot based on the cure rate group in the primary analysis of patients with LDH.


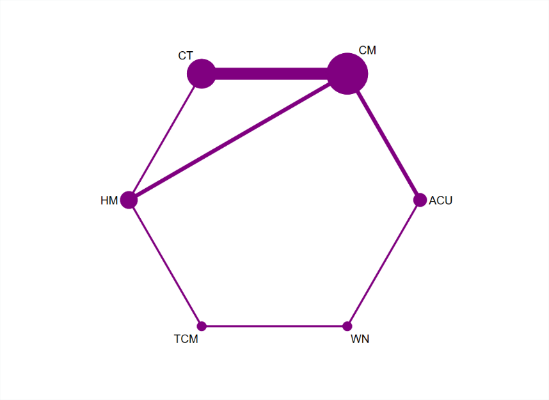


Figure6:

Network plot based on the ODI score group in the subgroup analysis of patients with LDH.


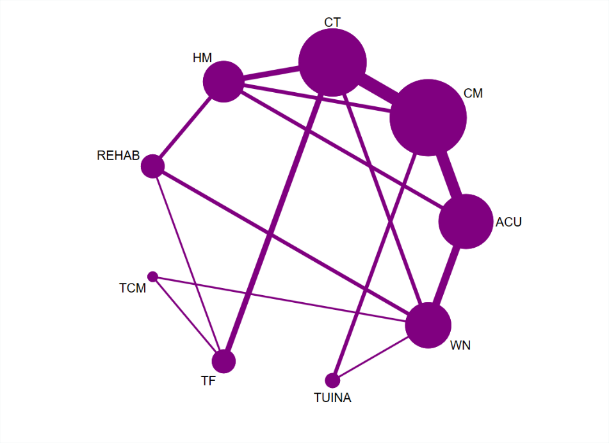


Figure7：

Network plot based on the VAS score group in the subgroup analysis of patients with LDH.


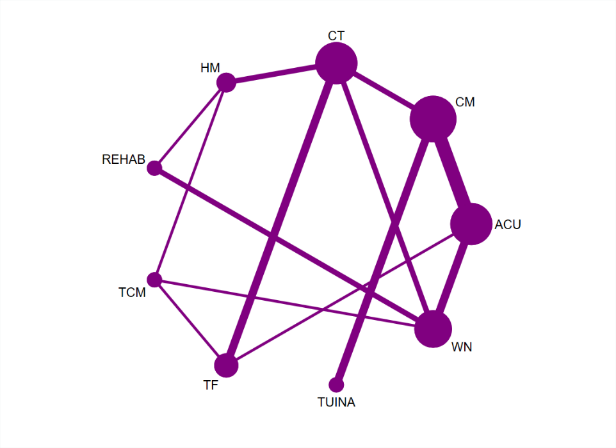


Figure8：

Network plot based on the JOA score group in the subgroup analysis of patients with LDH.


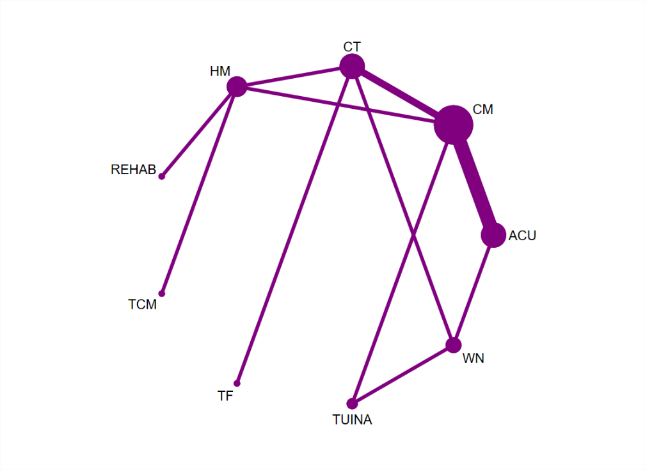


Figure9：

Network plot based on the cure rate group in the subgroup analysis of patients with LDH.


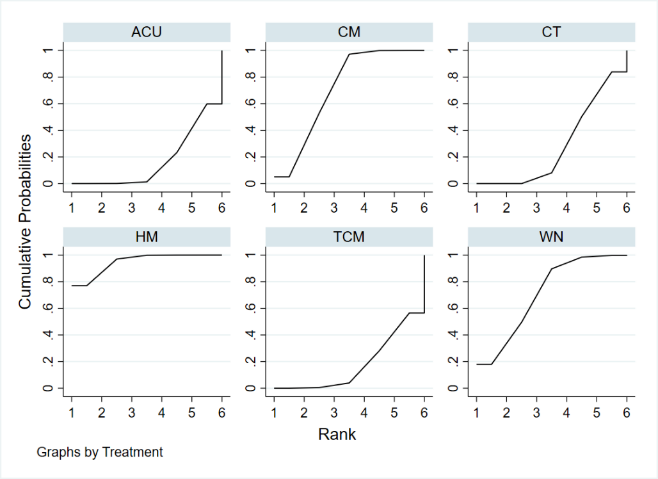


Figure10: Rank plot based on the ODI score group in the subgroup analysis of patients with LDH.


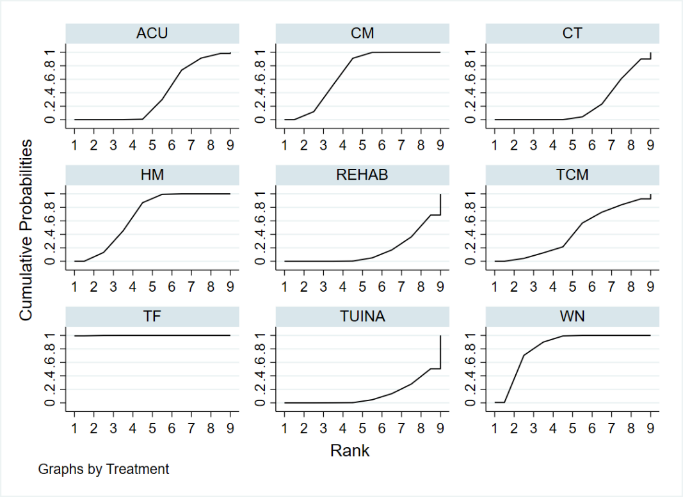


Figure11: Rank plot based on the VAS score group in the subgroup analysis of patients with LDH.


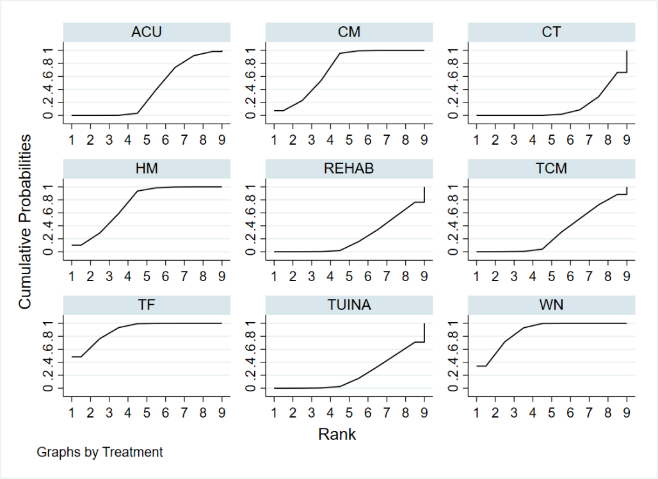


Figure12: Rank plot based on the JOA score group in the subgroup analysis of patients with LDH.


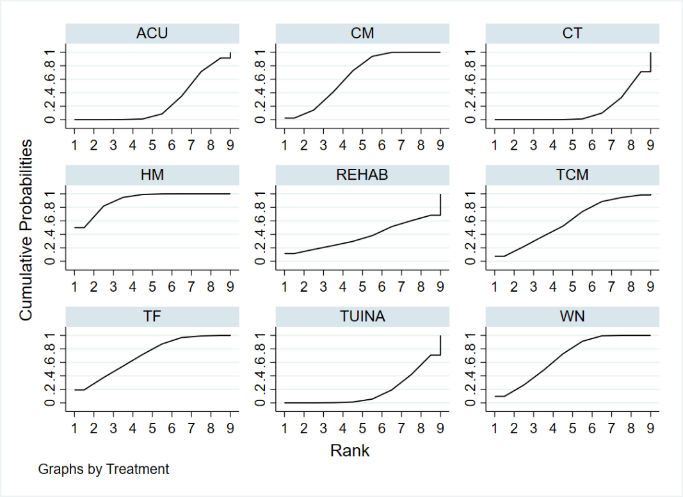


Figure13: Rank plot based on the cure rate group in the subgroup analysis of patients with LDH.


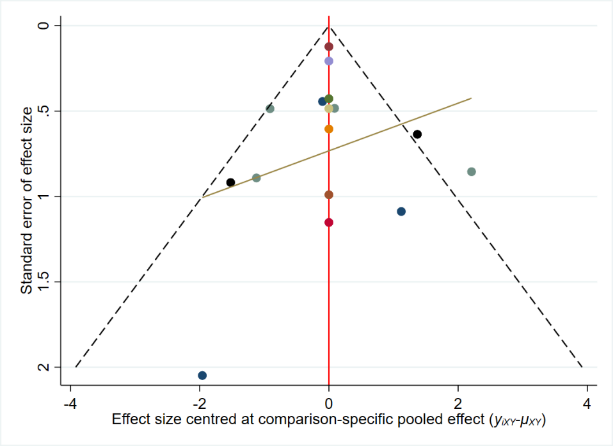


Figure14: Funnel plot based on the ODI score group in the primary analysis of patients with LDH.


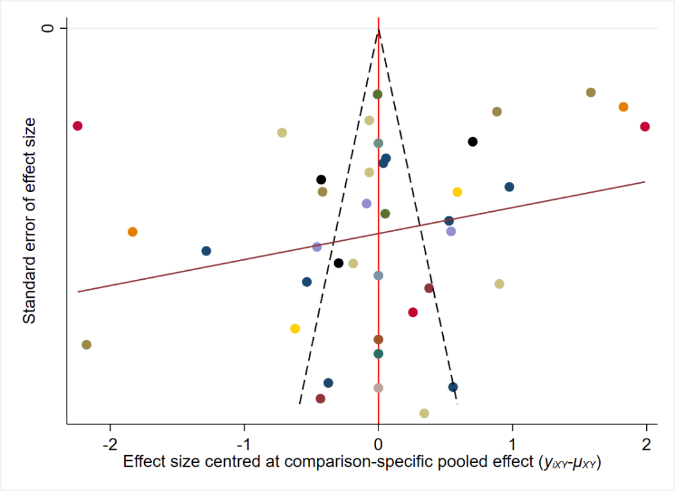


Figure15: Funnel plot based on the VAS score group in the primary analysis of patients with LDH.


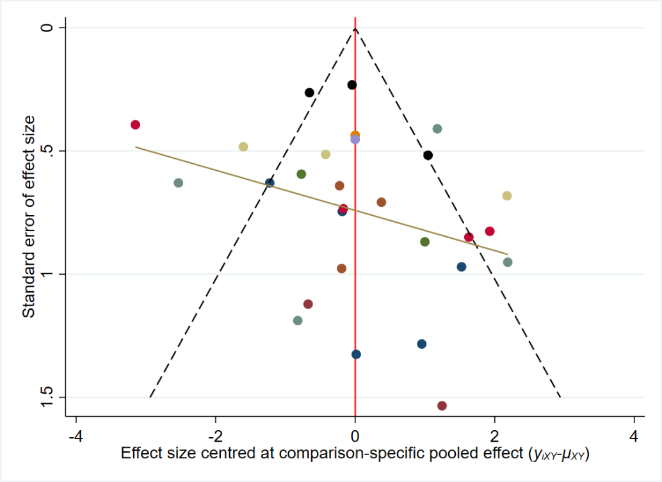


Figure16: Funnel plot based on the JOA score group in the primary analysis of patients with LDH.


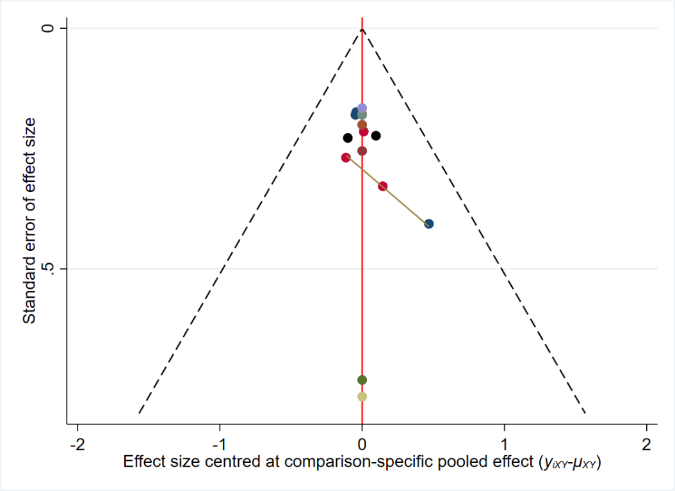


Figure17: Funnel plot based on the cure rate group in the primary analysis of patients with LDH.


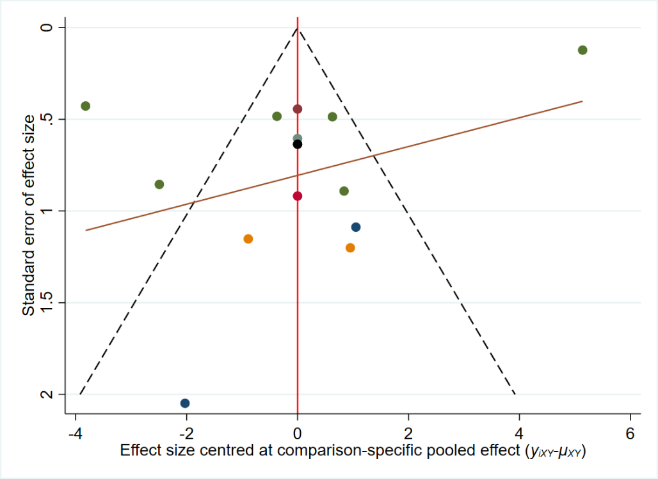


Figure18:Funnel plot based on the ODI score group in the subgroup analysis of patients with LDH.


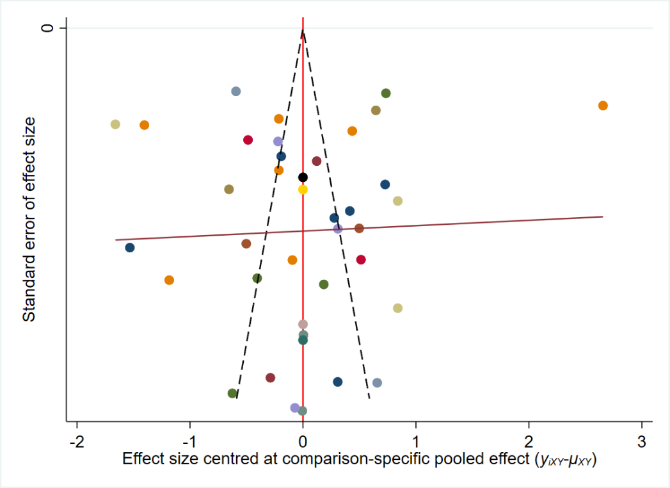


Figure29: Funnel plot based on the VAS score group in the subgroup analysis of patients with LDH.


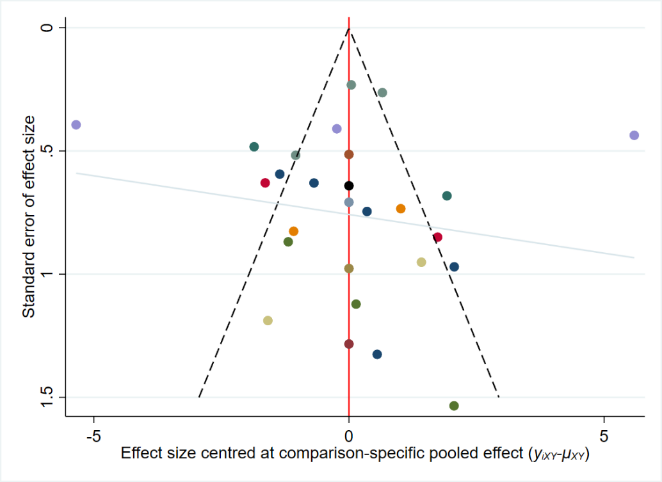


Figure20:Funnel plot based on the JOA score group in the subgroup analysis of patients with LDH.


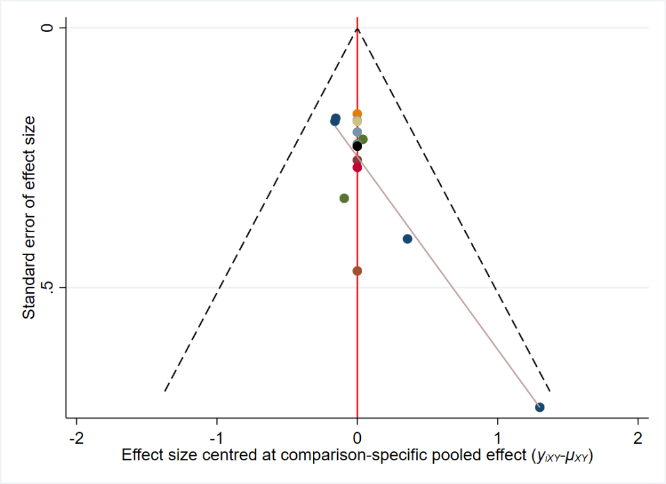


Figure21: Funnel plot based on the cure rate group in the subgroup analysis of patients with LDH.
